# Tumour-specific STING agonist synthesis via a two-component prodrug system

**DOI:** 10.1038/s41557-025-01930-9

**Published:** 2025-09-16

**Authors:** Nai-Shu Hsu, Cong Tang, Raquel V. Mendes, Carlos Labão-Almeida, Caio V. dos Reis, Ana R. Coelho, Marta C. Marques, Mar Cabeza Cabrerizo, Roman Misteli, Timothy P. C. Rooney, Marko Hyvönen, Francisco Corzana, Rita Fior, Gonçalo J. L. Bernardes

**Affiliations:** 1 Yusuf Hamied Department of Chemistry, University of Cambridge, Cambridge, UK; 2 GIMM - Gulbenkian Institute for Molecular Medicine, Lisbon, Portugal; 3 Xi’an Fengcheng Hospital, Xi’an, China; 4 Champalimaud Research, Champalimaud Foundation, Lisbon, Portugal; 5 The ALBORADA Drug Discovery Institute, University of Cambridge, Cambridge, UK; 6 Department of Biochemistry, University of Cambridge, Cambridge, UK; 7 Departamento de Química and Instituto de Investigación en Química de la Universidad de La Rioja (IQUR), Universidad de La Rioja, Logroño, Spain; 8 Translational Chemical Biology Group, Spanish National Cancer Research Centre, Madrid, Spain; 9 Present Address: The Discovery Centre, Cambridge, UK

**Keywords:** Small molecules, Drug delivery

## Abstract

Pharmacological activation of STING holds promise in cancer treatment. A recent trend is the development of tumour-specific or conditionally activated STING agonists for enhanced safety and efficacy. Here we explore an unconventional prodrug activation strategy for on-tumour synthesis of a potent agonist. Leveraging the unique mechanism of MSA2, a small-molecule agonist that dimerizes non-covalently before binding to STING, we showed that its analogues bearing reactive functional groups readily and selectively form covalent dimers under mild conditions and in complex environments. We identified a reacting pair that led to a thioether-linked dimer with submicromolar potency in cell-based assays. Caging one of the reactants with a self-immolative β-glucuronide moiety resulted in a two-component prodrug system that near-exclusively formed the active compounds in tumours overexpressing β-glucuronidase. These results exemplify the use of small-molecule recognition for on-site generation of active compounds from benign precursors.

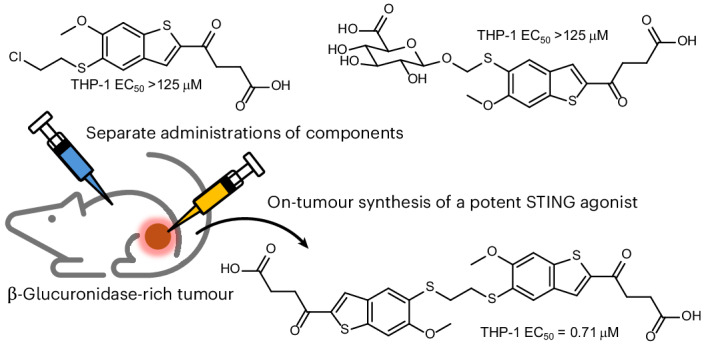

## Abstract (web summary)

STING is a promising drug target, but selective activation is necessary for safety and efficacy. Researchers have developed a two-component prodrug system for potent pharmacological activation of STING that offers excellent tumour targeting.

## Main

Misplaced cytosolic DNA in mammalian cells is commonly related to pathogen invasion, mitochondrial leakage and chromatin damage^1^. Their presence is thus closely monitored by cyclic GMP–AMP synthase (cGAS), a pattern recognition receptor that binds to double-stranded DNA in a sequence-independent manner and subsequently activates the Stimulator of Interferon Genes (STING) through the synthesis of the cyclic dinucleotide (CDN) messenger 2′,3′-cGAMP^2–5^. As an adaptor protein, STING scaffolds the phosphorylation of downstream effectors, including TANK-binding kinase 1 (TBK-1) and interferon regulatory factor 3 (IRF3), which induce the secretion of type-I interferons and other proinflammatory cytokines to elicit immune responses against the infected or damaged cells^1,6,7^.

Recently, pharmacological activation of STING has emerged as a promising strategy for cancer immunotherapy^8–10^. In animal models, STING agonists could induce tumour regression, promote macrophage repolarization and establish long-term protection by tumour-specific memory T cells^8–10^. Despite their antitumour effects, first-generation STING agonists based on modified CDNs have demonstrated moderate success in clinical trials, and their administration route is limited solely to intratumoural injections^11,12^. Efforts to discover non-CDN STING agonists suitable for systemic administration have led to several novel small molecules potentially applicable to a broader range of malignancies^13–15^. However, possible side effects of indiscriminate STING activation in both healthy and cancerous tissues remain a potential risk that limits their therapeutic index, as mutations that hyperactivate the cGAS–STING pathway are known to cause severe autoimmune diseases^16,17^. STING agonist overdosing could also lead to the apoptosis of T cells, which probably contributed to the failure to establish durable anticancer immunity in some studies^18–21^. Moreover, instead of promoting immunity against tumours, STING activation in B cells induced the secretion of anti-inflammatory cytokine IL-35 that impeded the actions of NK cells and led to poorer outcomes in mouse models^22^. These reports suggest that strategies for minimizing off-target STING activation could be vital in further developing STING-based therapeutics.

Several methods for tumour- or cell-type-specific delivery of STING agonists have been reported, including virus-like particles, HEK293-derived extracellular vesicles and antibody–STING agonist conjugates^23–26^. Of note, STING agonists combined with these delivery systems compared with the free drugs generally showed superior survival benefits in animal models while requiring lower doses, reinforcing the notion that non-specific activation could be the major obstacle in STING-based therapies. As an alternative to these macromolecule-based strategies, we seek to develop a small-molecule-based prodrug responsive to enzymes overexpressed in the tumour microenvironment (TME), thereby enabling a preferential activation of the STING agonist in the tumour.

**Fig. 1:**
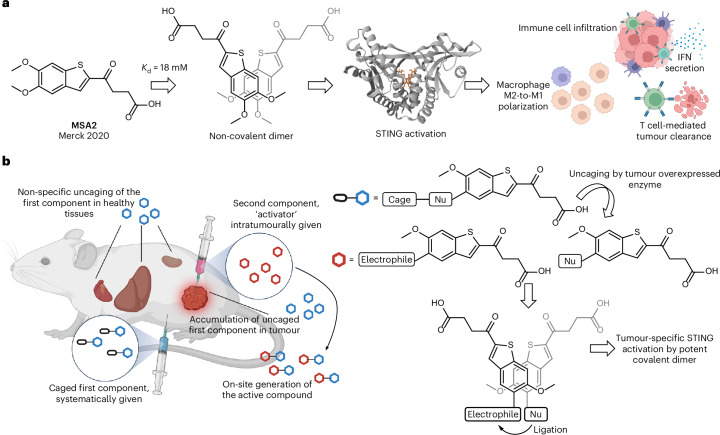
Activation of a two-component prodrug system based on the unique mechanism of MSA2. **a**, The unique predimerization mechanism of STING agonist MSA2 discovered by Merck. **b**, Our two-component prodrug strategy with enhanced tumour specificity for on-site synthesis of a potent STING agonist. Nu, nucleophile. Panels **a** and **b** created with BioRender.com.

MSA2 is a small-molecule non-CDN STING agonist discovered by scientists at Merck^15^. Under physiological conditions, it forms a weakly associated (*K*_D_ = 18 mM) non-covalent dimer by stacking the aromatic ring. The non-covalent dimer then binds to and activates STING (Fig. 1a). Inspired by this unique mechanism of action, we designed a two-component prodrug system for tumour-specific, on-site synthesis of a potent STING agonist (Fig. 1b). An MSA2 analogue bearing a caged nucleophile would be administered systematically and uncaged by enzymes overexpressed in the TME, leading to a preferential accumulation of the free nucleophile in the tumour. Another MSA2 analogue bearing an electrophile would be administered intratumourally, which would dimerize non-covalently with its nucleophilic counterpart and facilitate the ligation between them, forming a covalently linked, active dimer in situ. Conventional enzyme-responsive prodrugs for cancer treatment face the hurdle of inadequate tumour specificity due to expressions of the triggering enzymes in healthy tissues^27,28^. The administration site of the second component, therefore, serves to further confine drug activation to the tumour. Moreover, individual components could be rendered benign compared with the parent MSA2 by their nucleophilic or electrophilic modifications, thus further increasing the safety profile of the two-component system.

## Results

### MSA2 analogues readily form covalent dimers under mild conditions

**Fig. 2:**
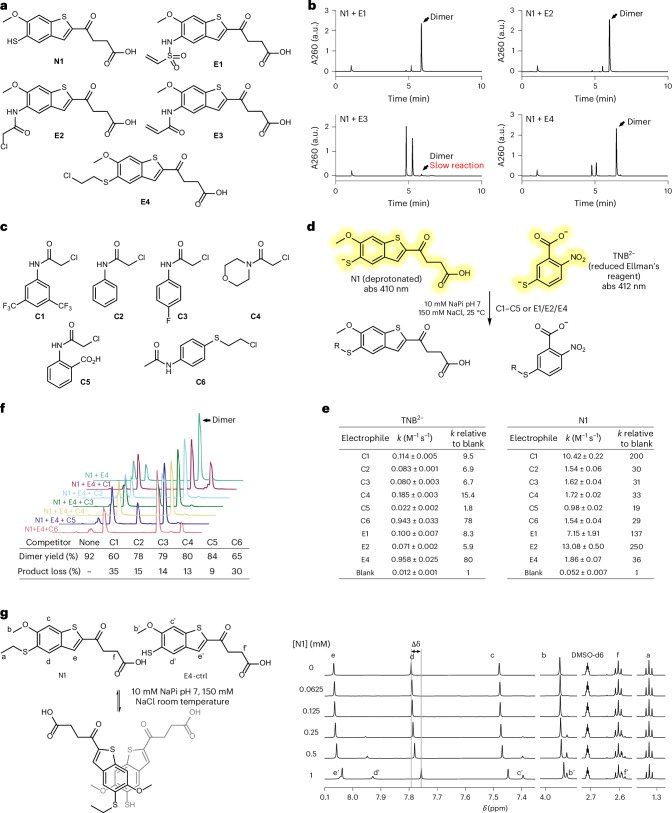
Facile formation of covalent dimers from MSA2 analogues. **a**, Structures of reactive MSA2 analogues. **b**, HPLC traces of the dimerization reactions. Each reacting pair (50 μM) was allowed to react for 2 h under physiological conditions (37 °C, 10 mM NaPi pH 7, 150 mM NaCl). Reactions were quenched with iodoacetamide (500 μM) to block the remaining free thiol. A260, absorbance at 260 nm; a.u., arbitrary unit; abs, absorbance. See Supplementary Fig. 1 for a detailed analysis of all identified species. **c**, Structures of electrophiles used as non-specific competitors. **d**, A colorimetric assay for rate constant measurement. **e**, A summary of calculated rate constants (mean ± s.d.). Data were averaged from three independent experiments performed on freshly prepared compound dilutions. See Extended Data Fig. 1 for the original plots. **f**, Top: HPLC traces of the N1–E4 dimerization reaction (50 μM each, 37 °C, 10 mM NaPi pH 7, 150 mM NaCl, 50 µM TCEP) in the presence of competitors. Reactions were quenched with iodoacetamide after 2 h. Right: yields calculated by comparing the integration value of the dimer peak using a calibration curve constructed with pure standards. See Supplementary Figs. 3 and 4 for details. **g**, Left: non-covalent dimerization between the non-reactive E4 analogue (E4-ctrl) and N1. Right: dose-dependent ^1^H NMR chemical shift perturbations of E4-ctrl by N1. [E4-ctrl] = 2 mM; 10 mM NaPi pH 7, 150 mM NaCl, 100 μM TCEP in D_2_O; 25 °C. Source data

We first synthesized several MSA2 analogues having either a thiol (N1), a vinyl sulfonamide (E1), a chloroacetamide (E2), an acrylamide (E3) or a half-mustard (E4) installed at the 5-methoxy group of MSA2 (Fig. 2a). As the 5-methoxy groups of the monomers are facing each other in the crystal structure of MSA2-bound STING (PDB: 6UKM), their positions are a natural choice of sites for linker attachment. The ligation reactions between nucleophile N1 and electrophile E1, E2 and E4 near-quantitatively afforded the desired dimers even at a low concentration (50 µM) and under mild conditions (pH 7, 37 °C) (Fig. 2b). Because MSA2 is known to dimerize in aqueous solutions, we hypothesized that, through proximity effects, such non-covalent interactions could lead to enhanced reaction rates between the analogues compared with those between N1 and a panel of non-specific electrophiles (Fig. 2c).

Conveniently, N1 exists primarily in its deprotonated form at neutral pH with strong absorption at 410 nm that diminishes after alkylation, allowing straightforward measurements of the rate constants using a published colorimetric assay^29^ (Fig. 2d,e). 2-Nitro-5-thiobenzoate (TNB^2−^) was used as a non-specific thiol to benchmark the intrinsic reactivities of the electrophiles. E4 and C6 demonstrated much faster alkylation rates of TNB^2−^, reflecting the higher reactivities expected for half-mustards. Likewise, similar alkylation rates were observed for the milder chloroacetamides, differing in most cases by no larger than twofold. These benchmarking results suggested that the intrinsic reactivities of the electrophiles were not substantially impacted by the MSA2 scaffold. While the same trend of reactivities was preserved for the alkylation of N1 by C2 to C5, clear rate enhancements were observed for the analogues E1 and particularly E2, which we believe could be attributed to the proximity effects induced by the dimerizing interactions. Among the non-specific electrophiles, C1 also showed an increased alkylation rate, albeit to a lesser extent. It remained possible that its rather hydrophobic aromatic ring could stack with the MSA2 scaffold of N1 as well and thus benefit from similar rate acceleration. Curiously, despite having the highest reactivities as indicated by TNB^2−^, alkylation rates of N1 by E4 or C6 were mediocre compared with the rest of the electrophiles.

To further investigate the eccentric behaviour of E4, we carried out a competition assay where equimolar mixtures of E4 and C1–6 (50 μM) competed for one equivalent of N1 (50 μM) (Fig. 2f). Surprisingly, in all cases, E4 prevailed, forming the covalent dimer as the major product. Such product distributions could not be explained in terms of their apparent second-order rate constants, as even the most reactive competitor, C1, merely reduced the amount of dimer by 35%. These results hinted that even weak interactions between the reactants could profoundly impact their selectivity. To ascertain that the thiol-modified structures of N1 and E4 were still capable of engaging in the same stacking interactions of MSA2, we incubated E4-ctrl, a non-reactive analogue of E4, with increasing concentrations of N1 and observed the resulting perturbations of proton nuclear magnetic resonance (NMR) chemical shifts (Fig. 2g). Indeed, protons close to the thiophene moiety (b, c, d and e) experienced dose-dependent shielding by the addition of N1, while the remote protons (a and f) showed no such effects, in agreement with the proposed spatial arrangement of the stacking monomers. Overall, the model reactions of the MSA2 analogues showed that they could efficiently and selectively form the desired covalent dimer under physiologically relevant conditions, providing the basis for further developing our two-component prodrug.

### Thioether-linked covalent dimers are highly potent STING agonists

**Fig. 3:**
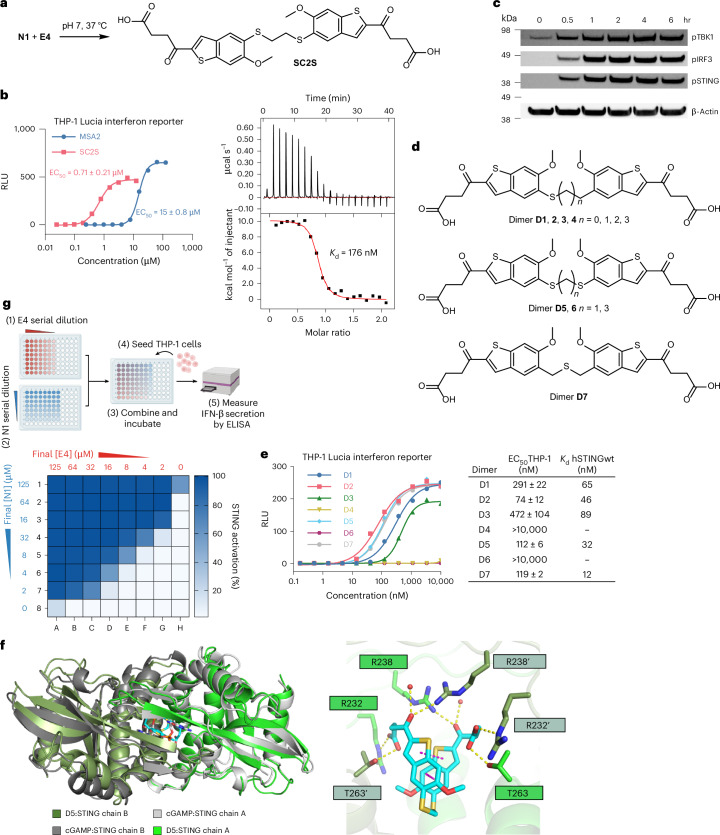
In situ formation of a potent thioether-linked dimer from MSA2 analogues. **a**, The structure of SC2S dimer formed from N1 and E4 under mild conditions. **b**, Left: dose–response curves of purified SC2S (red) and MSA2 (blue) on THP-1 Lucia ISG reporter cells. RLU, relative luminescence unit, reported as folds compared with DMSO-treated controls. Curves shown are averaged from three biological replicates, with cells that were split and passaged at least once. EC_50_ values are reported as mean ± s.d. Right: representative ITC data for binding constant determination of SC2S and purified LBD of wild-type human STING (hSTINGwt, Supplementary Fig. 5). **c**, Phosphorylation of major STING pathway effectors in THP-1 Lucia ISG cells treated with purified SC2S dimer (5 μM). The experiment was repeated once with similar results. **d**, Structures of thioether-containing dimers D1–7 with systematically explored linker lengths. **e**, Left: dose–response curves of dimers D1–7 on THP-1 Lucia ISG reporter cells. Right: THP-1 EC_50_ values and binding constants with hSTINGwt of D1–7. Cellular EC_50_ values were calculated from three biological replicates and reported as mean ± s.d. Binding constants were averaged from duplicate measurements. See Extended Data Fig. 2 for all ITC data. **f**, Binding mode of D5 with STING LBD. Left: crystal structure of D5-bound STING (PDB: 9QVT; dark and light green) superimposed on 2′,3′-cGAMP-bound STING (PDB:4KSY; dark and light grey). Right: key interacting residues with D5. All structural representations were generated using PyMOL. See Extended Data Fig. 4 for more structural analysis. **g**, Top: workflow to test the in situ formation of SC2S on THP-1 cells. Analogues N1 and E4 were serially diluted in two separate 96-well plates and mixed to generate an 8 × 8 matrix of various concentration combinations. The crude mixtures were then seeded with THP-1 cells. IFN-β concentrations in the supernatants were then measured by ELISA. Bottom: IFN-β secretion levels of THP-1 cells treated with N1 and E4 combined at different concentrations. The response from cells treated with 25 μM of purified SC2S dimer was defined as 100%. Panel **g** created with BioRender.com. Source data

We next tested purified covalent dimers of the reacting analogues on THP-1 Lucia ISG cells, a commercially available human monocyte cell line engineered for monitoring the interferon pathway. While other dimers were inactive, a thioether-containing covalent compound SCS2 (Fig. 3a), formed from N1 and E4, induced the interferon pathway with an half maximal effective concentration (EC_50_) of 0.71 µM; by comparison, the MSA2 parent molecule had an EC_50_ of 15 μM (Fig. 3b, left). The binding constant between SC2S and purified human wild-type STING was determined by isothermal titration calorimetry (ITC) to be 176 nM (Fig. 3b, right). Similar to the endogenous ligand 2′,3′-cGAMP, the reaction was highly endothermic, suggesting that both ligands induced substantial conformational change typical of STING activation^3^. Western blotting of THP-1 Lucia ISG treated with SC2S confirmed the phosphorylation of major STING pathway proteins (Fig. 3c).

Because thioether linkages represent a previously unexplored chemical space of MSA2, we synthesized a series of sulfur-containing dimers D1–7 with systematically varied lengths to gain further insight into their structure–activity relationship (SAR) (Fig. 3d,e). Three-atom linkages appeared to be optimal, with D2, D5 and D7 being the most active compounds in the THP-1 assay. Either shortening (D1) or lengthening the linker (D3) by one atom led to impaired cellular activities. Interestingly, their binding constants to purified STING ligand-binding domain (LBD) did not seem to correlate completely with their cellular EC_50_, a phenomenon reported previously^15^. Further lengthening of the linker (D4 and D6) completely abolished their activities. One-microsecond molecular dynamics (MD) simulations indicated that thio-containing linkers with four or fewer atoms benefit from more favourable interactions (for example, CH-π with Y167) and greater numbers of hydrogen bonds (Extended Data Fig. 3). Co-crystal structure of D5 and STING LBD (Fig. 3f, left, PDB: 9QVT) revealed a closed-lid conformation similar to that induced by 2′,3′-cGAMP (PDB: 4KSY). Well-preserved interactions with residues such as R232, R238 and T263 were observed (Fig. 3f, right). In comparison with STING bound by MSA2 (Extended Data Fig. 4, PDB: 6UKM), the covalent linkage in D5 seems to allow for slightly more intimate packing between the benzothiophene rings, thereby promoting STING closure.

A prerequisite of our prodrug strategy is the efficient formation of the active dimer in more complex environments. We first conducted the dimerization reaction in the presence of excessive glutathione, an endogenous thiol that could potentially compete for E4 (Extended Data Fig. 5). No glutathione–E4 adduct was detected, while the formation of SC2S remained largely unaffected. We next tested the dilution limit of the N1–E4 reaction that could still form effective levels of SC2S (Fig. 3g). Components N1 and E4 serially diluted in foetal bovine serum (FBS)-containing growth medium were combined at various concentrations and seeded with THP-1 cells to stimulate IFN-β secretions, the levels of which corresponded to the strengths of STING activation. Strong responses were observed even when N1 and E4 were combined at single-digit micromolar concentrations, demonstrating the robustness of the reaction. Importantly, neither N1 nor E4 alone showed any STING activation up to 64 μM, confirming that the individual components of our prodrug system were rendered benign by the introduction of the nucleophilic or electrophilic substituent. Together, these results provided in vitro evidence that N1 and E4 efficiently reacted at low micromolar concentrations and under physiological conditions to form the active SC2S dimer.

### Caged N1 and E4 enable β-glucuronidase-specific STING activation

**Fig. 4:**
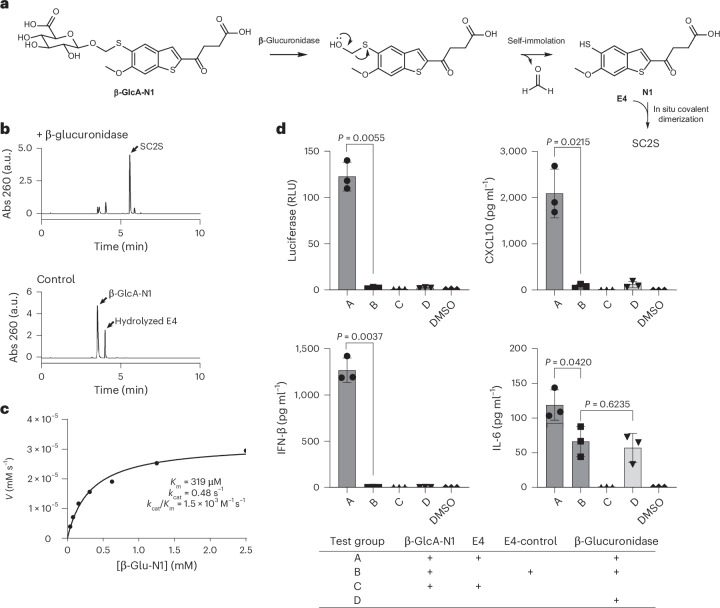
β-Glucuronidase-specific N1 decaging and subsequent in situ STING agonist formation. **a**, Cleavage of the glycosidic bond of β-GlcA-N1 (caged N1) releases a hemithioacetal, which collapses in aqueous environments and liberate the free N1. Liberated N1 could then react with E4 to form the active SC2S dimer. **b**, HPLC traces of reactions between β-GlcA-N1 and E4 (250 µM each) in the absence or presence of *E. coli* β-glucuronidase (250 Sigma units as defined by the manufacturer). 37 °C, 75 mM potassium phosphate pH 6.8, 1-h incubation. See Supplementary Fig. 6 for a detailed analysis of all identified species. **c**, Kinetics of β-GlcA-N1 cleavage mediated by human β-glucuronidase (5 μg ml^−1^, 67 nM). 37 °C, 0.2 M sodium acetate pH 4.5. *V*, velocity of the reaction. Each data point was averaged from duplicate measurements. **d**, Cytokine secretion assays for β-glucuronidase-specific STING activation of THP-1 Lucia ISG cells. Luciferase: reporter gene triggered by interferon pathway; E4-control: E4 substitute without the reactive chloride (see Fig. 2g for structure). The final concentration of each compound was 25 μM. The final concentration of β-glucuronidase was 250 units ml^−1^. Data are represented as mean ± s.d. from three biological replicates with cells that had been split and passaged at least once. Significance was analysed by two-tailed Welch’s *t*-test. Source data

β-Glucuronidase is an enzyme overexpressed in various cancers, particularly in the necrotic areas of solid tumours^30,31^. Its activity to cleave β-glucuronides has led to the design of numerous cancer-specific prodrugs and turn-on imaging probes^32–35^. Following a published procedure on synthesizing chloromethyl glycosides, we masked the free thiol of N1 with a β glucuronidase-cleavable moiety^36^. Upon cleavage of the glycosidic bond of the pronucleophile β-GlcA-N1, a hemithioacetal intermediate is released, which would spontaneously collapse in aqueous environments, generating in the process one molecule each of formaldehyde and N1. The uncaged N1 could then react with E4 to form the active SC2S dimer (Fig. 4a). We hypothesized that N1 would preferentially accumulate in TMEs overexpressing β-glucuronidase, while intratumoural administration of E4 would further enhance the tumour specificity by localizing the formation of the active STING agonist.

The specificity of the β-GlcA-N1/E4 prodrug system was first confirmed by reaction assays in a phosphate buffer (Fig. 4b). In the presence of β-glucuronidase, clean and near-quantitative formation of the active SC2S agonist was observed within 2 h. By contrast, β-GlcA-N1 remained stable under the same conditions without the added enzyme, whereas most E4 underwent hydrolysis. No hemithioacetal intermediate was found in the reaction mixture, suggesting it rapidly degraded to release N1 under physiological conditions. The stability of β-GlcA-N1 was further assessed in human plasma (Extended Data Fig. 6). After 48 h of incubation at 37 °C, more than 90% of β-GlcA-N1 remained intact. The Michaelis–Menten kinetics of the glycosidic bond-cleavage reaction determined with purified human β-glucuronidase yielded *K*_m_ and *k*_cat_ values close to those of previously reported substrates, indicating that the hemithioacetal spacer and the N1 moiety were well accommodated by the enzyme^37^ (Fig. 4c). The β-GlcA-N1/E4 system was then tested on THP-1 Lucia cells (Fig. 4d). The addition of β-glucuronidase to the culture medium resulted in significantly increased secretions of cytokines downstream of the STING pathway, including interferon-β, CXCL-10 and IL-6 (test group A). Swapping E4 for E4-ctrl (see Fig. 2g for the structure of E4-ctrl) diminished the cytokine inductions, supporting the idea that the linked dimer SC2S was the active compound (test group B). Neither β-GlcA-N1 nor E4 alone activated STING (test group C), although the commercial enzyme used in our study mildly stimulated the secretion of IL-6, which we attributed to the signalling of immune pathways other than STING (test group D). These results provide evidence of the in vitro functionality of the β-GlcA-N1/E4 two-component prodrug system.

### The β-GlcA-N1/E4 prodrug system shows efficacy in a zebrafish xenograft model

**Fig. 5:**
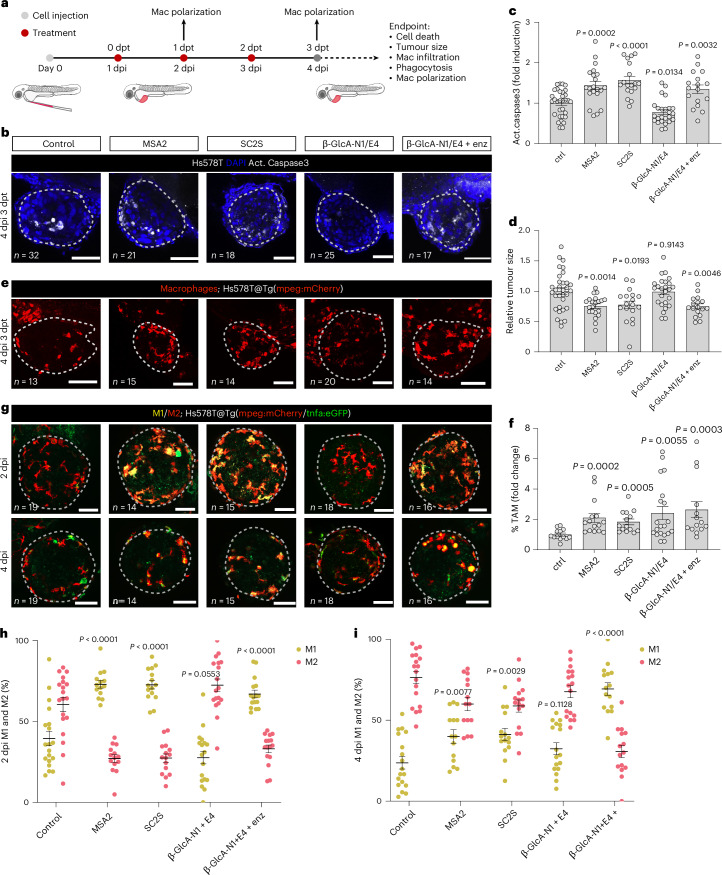
β-GlcA-N1/E4 showed efficacy in the Hs578T zebrafish xenograft model in the presence of β-glucuronidase. **a**, Experimental design. Hs578T cells were injected into the PVS of 2 dpf zebrafish embryos. At 1 dpi, xenografts were randomly distributed into five treatment groups: DMSO, MSA2 (15 μM), SC2S (0.62 μM), β-GlcA-N1/E4 (20 μM each) and β-GlcA-N1/E4 + enzyme (20 μM each + 250 units ml^−1^ of β-glucuronidase) with daily renewal of the compounds. At 4 dpi (3 dpt), xenografts were fixed and analysed for apoptosis, tumour size, phagocytosis and macrophage infiltration. Macrophage polarization analysis was performed at 2 dpi (1 dpt) and 4 dpi (3 dpt) by live imaging. All compounds were administrated at concentrations below their MTCs (Extended Data Fig. 7a). mac, macrophages; enz, enzyme (β-glucuronidase). **b**, Treated xenografts at 4 dpi. Blue, DAPI; white, activated Caspase-3. **c**, Quantification of Caspase-3 activation. Act., activated. **d**, Quantification of tumour sizes (number of tumour cells). For **c** and **d**, the results of two independent experiments were averaged and are presented as mean ± s.e.m. Each dot represents one xenograft. **e**, Representative confocal projection images of macrophages (red) in Hs578T xenografts in Tg(mpeg1:mcherry-F) at 4 dpi. **f**, Fold induction of TAM (defined by the ratio of the total number of macrophages and that of tumour cells) normalized to control treatments at 4 dpi. Data were averaged from two independent experiments and are presented as mean ± s.e.m. **g**, Representative confocal images of Hs578T xenografts injected in Tg(mpeg1:mcherry-F, tnfa:GFP-F) at 2 and 4 dpi. Red, macrophages; green, tnfa-positive cells; yellow, macrophages expressing TNF (that is, M1-like macrophages). **h**, Ratios of M1- to M2-like macrophages in the TME at 2 dpi. **i**, Ratios of M1-to M2-like macrophages at 4 dpi. For **h** and **i**, data are presented as mean ± s.e.m. Images are maximum intensity projections. All images are anterior to the left, posterior to right, dorsal up and ventral down. Scale bars, 50 μm. Dashed lines delineate the tumours. All datasets were challenged by D’Agostino and Pearson and Shapiro–Wilk normality tests. Those with a Gaussian distribution were analysed by parametric unpaired *t*-test, and those that did not pass the normality tests were analysed by non-parametric unpaired Mann–Whitney test. All tests were two-sided. Source data

We next tested the in vivo efficacy of the β-GlcA-N1/E4 prodrug system on a zebrafish xenograft model^38,39^. Compounds were first assessed for their maximum tolerated concentrations (MTC) on non-tumour zebrafish larvae to determine the treatment dosages (Extended Data Fig. 7a). Fluorescently labelled Hs578T cells (a triple-negative breast cancer cell line) were xenotransplanted into the perivitelline space (PVS) of 2 days postfertilization (dpf) zebrafish embryos, which after 24 h were treated with various compounds and analysed at 4 days postinjection (dpi) by confocal immunofluorescence microscopy (Fig. 5a).

Treatments of xenografts with either MSA2 or purified SC2S dimer significantly induced apoptosis, as indicated by elevated levels of activated Caspase 3 (Fig. 5b,c). Unexpectedly, the β-GlcA-N1/E4 combination failed to induce apoptosis, although adding β-glucuronidase to the medium restored their activity. It was likely that insufficient expression levels of the enzyme in the tumours limited the activation of the prodrug system. Reduction in tumour size was observed across all treatment groups that induced apoptosis, consistent with the tumour-ablating effect of previously reported STING agonists when given at high doses^14,15,21^ (Fig. 5d). A key feature of STING agonist-based treatments is the inflammation of the TME, which promotes macrophage infiltration and their repolarization from the immunosuppressive M2 to the proinflammatory M1 phenotype^9^. To study the immunomodulatory effects of our prodrug system, a transgenic zebrafish line with mCherry-expressing macrophages (Tg(mpeg1:mcherry-F)) was xenotransplanted with Hs578T cells and treated with the β-GlcA-N1/E4 prodrug system or control compounds (Fig. 5e,f). All treatments significantly increased tumour-associated macrophages (TAMs), although for β-GlcA-N1/E4 the effect was more pronounced in the presence of added β-glucuronidase. A distinguishing feature of M1-like macrophages is the expression of tumour necrosis factor (TNF)^40,41^. Therefore, we utilized the double-transgenic zebrafish line Tg(mpeg1:mCherry-F; tnfa:eGFP-F) expressing mCherry in macrophages and eGFP under the control of the *tnfa* promoter to quantify the M1-like subpopulation of the macrophages within the xenograft tumours (Fig. 5g–i). In the presence of added β-glucuronidase, the β-GlcA-N1/E4 combination potently polarized the macrophages towards the M1-like phenotype (mpeg and TNF positive, yellow) at 1 day posttreatment (dpt); the effect remained unabated even at 3 dpt compared with that of the control compounds MSA2 and SC2S dimer. In addition, the macrophages showed increased phagocytic activities accompanying their M2-to-M1 repolarizations (Extended Data Fig. 7b).

Together, these results suggest that, contrary to our expectations, the expression levels of β-glucuronidase in Hs578T xenografts were insufficient to trigger the β-GlcA-N1/E4 combination. A possibility was that the zebrafish xenograft lacked the necrotic areas of larger, more developed tumours, which contained much higher amounts of extracellular β-glucuronidase, as indicated by some previous studies^32,33^. With added β-glucuronidase, the prodrug system elicited tumour apoptosis and durable modulation of macrophages, lending further evidence for the in vivo feasibility of our prodrug system.

### Treating a mouse tumour model with the β-GlcA-N1/E4 combination leads to tumour-specific, in situ formation of the active dimer and confers survival benefits

We next evaluated the therapeutic effects of our prodrug system on an MC38 syngeneic mouse model (Extended Data Fig. 8a). Consistent with our earlier zebrafish experiments, the combination of β-GlcA-N1/E4 did not elicit an appreciable antitumour response, a result that could probably be attributed to the lack of β-glucuronidase in the TME. Indeed, β-GlcA-N1/E4 preincubated with β-glucuronidase showed tumour-attenuating effects and conferred similar survival benefits compared with the benchmark MSA2 (Extended Data Fig. 8b,c). None of the treatment groups induced significant body weight loss (Extended Data Fig. 8d). Secretome profiling of the sera indicated that both MSA2 and preincubated β-GlcA-N1/E4 activated highly overlapping sets of cytokines, suggesting the similarity of their mechanisms (Extended Data Fig. 8e).

**Fig. 6:**
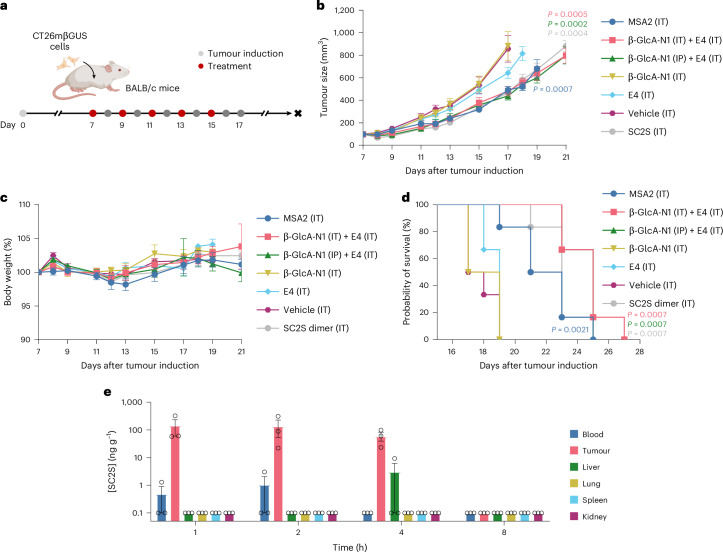
In vivo proof of concept of the two-component prodrug on a β-glucuronidase-overexpressing mouse tumour model. **a**, Experimental design. CT26 cells overexpressing the murine β-glucuronidase (CT26mβGUS) were inoculated into the right flank of BALB/c mice. Overexpression of β-glucuronidase was confirmed by western blotting (Extended Data Fig. 9a). Treatments began once the tumour volume reached about 90 mm^3^. MSA2: 5 mg kg^−1^ (IT); β-GlcA-N1: 12.5 mg kg^−1^ (IT or IP); E4: 5 mg kg^−1^ (IT); SC2S dimer: 8.6 mg kg^−1^ (IT, matching molar concentration of the β-GlcA-N1/E4 treatment group). *n* = 6 for each treatment group. IP, intraperitoneal; IT, intratumoral. **b**, Tumour volume curves. Data were analysed by one-way ANOVA, followed by multiple comparisons adjusted with the Šidák–Holm correction. **c**, Body weight monitoring. **d**, Survival curves. Differences between the compound-treated groups and the vehicle-treated control group were analysed by the log-rank test. **e**, Tissue distribution of SC2S dimer formed after the β-GlcA-N1 (IP) + E4 (IT) treatment. Here, 0.1 ng g^−1^ indicates that [SC2S] was below the detection limit. All data points are presented as mean ± s.e.m. *n* = 3 for each timepoint. Panel **a** created with BioRender.com. Source data

To demonstrate the in situ formation of the SC2S dimer in a β-glucuronidase-rich TME, we turned to a CT26 syngeneic tumour model (CT26mβGUS) where the inoculated cell line was genetically engineered to express a membrane-anchored mouse β-glucuronidase for applications in gene-directed enzyme prodrug therapy^42^ (Fig. 6a). At comparable doses, both MSA2 and the β-GlcA-N1/E4 treatments attenuated tumour growth to similar extents, while the individual components did not show significant effects (Fig. 6b). The benign nature of either β-GlcA-N1 or E4 alone was further supported by body weight monitoring, as no significant losses were observed (Fig. 6c). The β-GlcA-N1/E4 combination conferred slightly better survival benefits compared with MSA2 (Fig. 6d). It is worth noting that the effectiveness of β-GlcA-N1/E4 was indistinguishable from the SC2S control treatment group, where a matching concentration of the purified dimer was given intratumourally, from which one could infer that the efficiency of dimer formation from the prodrug system was not the limiting factor of its efficacy. Such results may represent the inherent limitations of STING-based therapy on our tumour model.

The tissue distribution of the active SC2S dimer resulted from the β-GlcA-N1/E4 combination (β-GlcA-N1 was given intraperitoneally (IP); E4 was given intratumourally (IT)) was analysed with mass spectrometry (Fig. 6e). For up to 4 h after the treatment, the SC2S dimer was detected at a stable level almost exclusively in the tumour. Apart from traces of SC2S detected in the blood and the liver, the concentrations of the agonist were below the detection limit across all other organs analysed. By comparison, the reported pharmacokinetic profile of intratumourally injected MSA2 indicated that its concentration in the tumour decreased rapidly past the 2-h timepoint, and substantial leakage into the plasma occurred^15^. Haematotoxicity (Extended Data Fig. 9b) and histopathology analyses (Extended Data Fig. 10) suggested that the treatments were generally well tolerated, although modest decreases in the levels of red blood cells, haemoglobin and haematocrit were observed for the MSA2 treatment group. Overall, these results provide an in vivo proof of concept of the two-component prodrug system and show that the active dimer could indeed be preferentially formed in a β-glucuronidase-rich TME, with minimal off-target accumulation relative to MSA2.

## Discussion

To summarize, we report a two-component prodrug system for STING activation utilizing the unique dimerization mechanism of the non-CDN STING agonist MSA2. Based on structural information of the non-covalent dimer in complex with STING, we synthesized MSA2 analogues with either an electrophile or a nucleophile that formed covalent dimers efficiently at low micromolar concentrations and under physiological conditions. One of the dimers with a thioether linkage activated THP-1 reporter cells at submicromolar concentrations. Caging the nucleophilic analogue with a self-immolative β-glucuronide moiety allowed for β-glucuronidase-specific STING agonist formation in cell-based assays and zebrafish xenograft models. Using a mouse tumour model overexpressing β-glucuronidase, we showed that separate administration of the two components resulted in near-exclusive formation of the active dimer in the tumour, thus providing an in vivo proof of concept of our prodrug system. Considering that β-glucuronidase-responsive prodrugs or imaging probes are known to be uncaged and to accumulate significantly in normal organs such as the liver, the kidney and the heart, the specificity achieved by the two-component system is remarkable^32,43–46^.

In recent years, a plethora of strategies for ‘on-tumour’ generation of therapeutics has been reported^47,48^. Conceptually related to our two-component prodrug, these systems likewise involve separate administration of two elements: a ‘trigger’ that preferentially accumulate in tumours, and a latent effector to be rendered active by the trigger through specific bond formation or cleavage. Triggers are commonly directed to tumours through the enhanced permeability and retention effects of nanomaterial, conjugations to antibodies or ligands, or even direct surgical implantations. An assumption that has been made throughout, however, is that the trigger must be a bioorthogonal reaction, such as palladium-catalysed depropargylation, *trans*-cyclooctene-tetrazine cycloaddition or azide–phosphine reduction^49–51^. As an alternative to this established paradigm, we showed that even a plain nucleophilic substitution reaction could efficiently and tumour-selectively form the active compound by utilizing the non-covalent interactions between the reactants. Compared with the rate constants of most bioorthogonal reactions, which fell in the range of 10^−1^ to 10^−3^ M^−1^ s^−1^, the dimerization rates of the MSA2 analogues reported herein were at least ten- to a hundred-fold faster, showing a potential benefit of moving beyond the constraints of biorthogonality. The reaction between the constituent components of our prodrug system also brings about important structural changes in these molecules, resulting in drastically different bioactivities between the reactants and the product. As demonstrated in our cell-based assays, neither β-GlcA-N1 nor E4 appreciably activates STING, while their product is a potent agonist at submicromolar concentrations. Therefore, toxicities stemming from non-specific decaging could be rendered less detrimental, as only a relatively benign precursor rather than a fully active compound would be released. A similar concept has also been explored in a palladium-based prodrug strategy, where targeted delivery of palladium nanoparticles catalysed an in-cell Suzuki–Miyaura coupling to synthesize a cytotoxic compound from two innocuous precursors^52^.

A question that remained unanswered is the exact nature of the dimerizing interactions between the MSA2 molecules. Indeed, at first glance, the benzo[*b*]thiophene core of MSA2 seems to be a rather inconspicuous moiety that is commonly used in medicinal chemistry. Yet, its stacking is strong enough to hold two monomers together with sufficient lifetime to bind to STING and, as leveraged in this work, to efficiently and specifically form a new covalent bond. It is conceivable that the discovery of more molecular scaffolds with such self-recognizing properties could open up new possibilities in prodrug design and beyond.

## Methods

### General material and instrumentation

Most chemicals and solvents were purchased from Acros, Alfa Aesar, Sigma-Aldrich, MedChemExpress, Fluorochem, Biosynth Carbosynth and Cayman Chemical. Milli-Q ultrapure water was used in all relevant experiments. Thin-layer chromatography was performed with Supelco silica gel 60 F254 plates. Flash column chromatography was performed with Millipore silica gel 60 (0.040–0.063 mm, 230–400 mesh). NMR spectra were recorded on a Bruker Avance III 500 MHz spectrometer equipped with a BBO smart probe or on a Bruker Avance 700 MHz spectrometer equipped with a TXO cryoprobe. Spectra were processed with MestreNova. High-resolution mass spectra of small molecules were recorded on a Waters LCT Premier mass analyser equipped with an Agilent 1100 high-performance liquid chromatography (HPLC) system or on a Waters Xevo G2-S bench top QTOF. Liquid chromatography–mass spectrometry (LC–MS) analysis of reaction mixtures was performed on a Waters SQD2 mass analyser coupled with a Waters H-class UPLC system (Waters Acquity UPLC BEH C18 reversed-phase column). Preparative HPLC was performed on an Agilent 1260 Infinity II chromatography system (ZORBAX 5 Eclipse Plus C8 21.2 × 150 mm reversed-phase column).

### Alkylation rate constant measurements

Electrophiles C1–C5 and E1, E2 and E4 were prepared as 50 mM dimethyl sulfoxide (DMSO) stock solutions, diluted in the reaction buffer (10 mM NaPi pH 7, 150 mM NaCl) to 500 µM, and dispensed (100 μl each well) into an ultraviolet-transparent 96-well plate (Corning 3635). Before the start of each assay, thiol N1 (or Ellman’s reagent) was freshly weighed and prepared as a 50 mM DMSO stock solution. N1 was diluted in the reaction buffer containing 200 µM Tris(2-carboxyethyl)phosphine) (TCEP) (400 μM for Ellman’s reagent) to prepare a 200 µM working solution, which was then added (100 μl each well) to the electrophiles, resulting in the following initial condition: 250 μM of the electrophile, 100 μM of the nucleophile, 10 mM NaPi pH 7, 150 mM NaCl and 100 µM TCEP (assuming complete reduction of the Ellman’s reagent). The absorbance at 410 nm (412 nm for Ellman’s reagent) was monitored at 25 °C with a plate reader (Molecular Devices SpectraMax i3x). The concentration of the remaining nucleophile at each timepoint was determined by comparing the background-subtracted absorbance with a calibration curve obtained by serial dilutions of the 200 µM working solution. The concentration of the remaining electrophile at each timepoint was determined by calculating the amount of nucleophile consumed. Rate constants were obtained by fitting the data with the standard second-order rate law (GraphPad Prism).

### Michaelis–Menten kinetics of β-GlcA-N1 cleavage by human β-glucuronidase

Human β-glucuronidase (AcroBiosystems BEB-H52H3) was reconstituted in deionized water to a concentration of 1 mg ml^−1^. The enzyme stock solution was further diluted in assay buffer (0.2 M acetate pH 4.5) to 10 μg ml^−1^ (134 nM). β-GlcA-N1 (50 mM DMSO stock) was twofold serially diluted in assay buffer from 5 to 0.078 mM. The enzyme solution and diluted β-GlcA-N1 were incubated in a transparent 96-well plate and incubated at 37 °C until thermo equilibrium was reached. Then, 100 µl of β-GlcA-N1 at various concentrations was mixed with 100 µl of β-glucuronidase using a multichannel pipette, resulting in the following initial condition: [β-GlcA-N1] = 2.5, 1.25, 0.62, 0.31, 0.16, 0.08 and 0.04 mM; [β-glucuronidase] = 67 nM; 5% DMSO (v/v). The reactions were incubated at 37 °C. At 30-min and 60-min timepoints, a 50-µl portion was withdrawn from each reaction mixture and mixed with 50 µl of quenching buffer (1 M sodium carbonate, 1 mM TCEP). The concentration of liberated N1 was determined by comparing the absorbance of each quenched reaction at 410 nm to a calibration curve of purified N1. Reaction velocities were calculated from the slopes of [N1] versus time plots. The velocity versus [β-GlcA-N1] plot was then fitted with the standard Michaelis–Menten kinetic model (GraphPad Prism).

### Purification of human STING wild-type 138-379

*Escherichia coli* BL21 (DE3) (Thermo Scientific EC0114) was transformed with a pET28a plasmid (Genscript) harbouring the codon-optimized sequence of SUMO-hSTINGwt (aa138-379). Transformed BL21 cells were inoculated into LB medium (50 µg ml^−1^ kanamycin) and incubated at 180 rpm, 37 °C overnight. The overnight culture was then diluted (1:100 dilution) in 2× YT medium (50 µg ml^−1^ kanamycin) and incubated at 180 rpm, 37 °C until optical density at 600 nm (OD_600_) reached 0.6 (approximately 3 h). The bacterial culture was then cooled to 16 °C, induced with 0.5 mM isopropyl 1-thio-d-galactopyranoside and incubated overnight at 180 rpm, 16 °C. The bacterial cells were pelleted at 4,000*g* for 30 min, resuspended in 20 ml of lysis buffer (25 mM HEPES pH 7.5, 150 mM NaCl, 20 mM imidazole and 2 mM TCEP supplied with 2 tablets of cOmplete mini EDTA-free protease inhibitors) and disrupted by sonication on ice. The total lysate was clarified by centrifugation at 22,000*g* for 30 min. The supernatant was collected and filtered through a 0.45-µm filter (Millipore SLHAR33SS). The clarified lysate was then incubated on a roller with 10 ml bed volume of Ni-NTA resin (Thermo Scientific 88223) at 4 °C for 1 h. The Ni-NTA resin was then packed in a column, flushed with 20 ml of wash buffer (25 mM HEPES pH 7.5, 150 mM NaCl and 20 mM imidazole) and eluted with 40 ml of elution buffer (25 mM HEPES pH 8.0, 150 mM NaCl and 250 mM imidazole). The eluted protein was concentrated to approximately 5 ml (Millipore Amicon 10 kDa molecular weight cut-off (MWCO)) and buffer-exchanged into storage buffer (25 mM HEPES pH 7.5 and 150 mM NaCl). TCEP was then added to a final concentration of 2 mM, and 250 units of SUMO protease (Merck SAE0067) were added. The cleavage reaction mixture was left to stand at 4 °C overnight until LC–MS analysis indicated the completion of the cleavage reaction. The cleavage mixture was incubated with 10 ml bed volume of Ni-NTA resin on a roller at 4 °C for 1 h and packed in a disposable column. The flowthrough was collected, and the resin was further flushed with 20 ml of wash buffer. The eluted fractions were combined, concentrated and further purified by size-exclusion chromatography (Cytiva, Superdex 200 Increase 10/300 GL). The purified protein in storage buffer was concentrated to 330 µM (dimer concentration) as determined by bicinchoninic acid (BCA) assay, aliquoted, flash-frozen in liquid nitrogen and stored at −80 °C for future use.

### ITC

The binding constants of purified dimers were measured by ITC (Malvern Panalytical AutoITC200) in a reverse-titration format. Stock DMSO solutions of each dimer were dissolved in titration buffer (25 mM HEPES pH 7.5 and 150 mM NaCl) to a final concentration of 25 µM and added to the titration cell. The syringe was filled with 250 µM of purified STING in a matching buffer. Titration was performed at 25 °C with the following parameters: 750 rpm stirring, 0.2 µl initial injection followed by 2-µl injections of the dimer solution every 120 s. Titration of STING protein into blank buffer was separately performed and subtracted from the raw data before the analysis. The background-subtracted titration curves were fitted with the built-in Origin software using the standard one-site model.

### MD simulations

The crystal structure of MSA2 in complex with STING (PDB entry: 6UKM)^15^ was used as initial structure for all simulations. MD simulations were performed with the AMBER 22 package^53^, implemented with ff14SB^54^ and gaff2^55^ to correctly reproduce the conformational behaviour of the protein and the ligands, respectively. The LEaP module of AMBER 22 was used to generate the topology and coordinate files for the MD simulations, which were carried out using the CUDA version of the PMEMD module of the AMBER simulation package. Each complex was immersed in a water box with a 10 Å buffer of TIP3P water molecules^56^, and the system was neutralized by adding explicit counter ions (Na^+^). A two-stage geometry optimization approach was performed with the PMEMD module. The first stage minimizes only the positions of solvent molecules and ions, using a 50 kcal mol^−1^ Å^−2^ harmonic potential, and the second stage is an unrestrained minimization of all the atoms in the simulation cell. In both stages, 2,500 steps of steepest descent minimization were followed by 2,500 steps of conjugate gradient minimization. The systems were then heated by incrementing the temperature from 0 to 300 K under constant pressure of 1 atm and periodic boundary conditions for 2 ns. Harmonic restraints of 10 kcal mol^−1^ were applied to the solute, and the Andersen temperature coupling scheme^57^ was used to control and equalize the temperature. The time step was kept at 1 fs during the heating stages. The SHAKE algorithm was applied to constrain all bonds involving hydrogen atoms^58^. Long-range electrostatic effects were modelled using the particle-mesh-Ewald method^59^. A sharp cut-off of 8 Å was applied to Lennard–Jones interactions. Each system was equilibrated for 2 ns with a 2-fs time step at a constant volume and temperature of 300 K. Production trajectories were then run for an additional microsecond under the same simulation conditions. Binding free energy calculations were performed using the MM-GBSA method^60^. This approach combines molecular mechanics energies with solvation terms estimated using the generalized Born implicit solvent model. Representative snapshots were extracted from the equilibrated MD trajectories for postprocessing. Entropic contributions were not considered in this study.

### Potency test of purified dimers on THP-1 cells

THP-1 Lucia ISG cells (InvivoGen thpl-isg) were maintained in the growth medium (RPMI 1640, 2 mM GlutaMAX, 25 mM HEPES, 10% FBS and Pen-Strep/Normocin) at 37 °C in a humidified atmosphere containing 5% CO_2_. Cells were pelleted (150*g*, 10 min) and resuspended in the growth medium (40% conditioned) at a density of 1 × 10^6^ cells ml^−1^. The cell suspension was dispensed (50 µl each well) into a lidded white 96-well plate (BrandTech 781665). Test compounds or DMSO control were diluted in the growth medium and sterilized by filtering through 0.22-µm polyethersulfone (PES) membranes (Merck SLGP033RS). Compounds serially diluted in the growth medium were then added to (50 µl each well) the cell suspension, resulting in a final density of 5 × 10^5^ cells ml^−1^ with 20% conditioned medium. In all cases, the DMSO content did not exceed 0.5%. Treated cells were incubated at 37 °C for 24 h. Upon cooling down to room temperature, 50 µl of reconstituted luciferase substrate (InvivoGen QUANTI-Luc) was added to each well via the injector of the plate reader (Molecular Devices SpectraMax i3x), and the luminescence output was recorded (4 s delay, 0.1 s integration). Raw signals were divided by the averaged signal of the DMSO-treated control wells and reported in relative luminescence unit (RLU). The dose–response curve of each replicate was fitted individually using the standard 4PL model (GraphPad Prism). The EC_50_ value of each compound was averaged from three independent experiments and reported as mean ± s.e.m.

### Testing the dilution limits of N1/E4 combination to activate THP-1 cells

THP-1 cells (Cell Line Service) were maintained in the growth medium (RPMI 1640, 2 mM GlutaMAX, 1 mM sodium pyruvate, 1× non-essential amino acids, 10% FBS and Pen-Strep) at 37 °C in a humidified atmosphere containing 5% CO_2_. On the day before the experiment, the cells were resuspended in fresh growth medium at a density of 5 × 10^5^ cells ml^−1^, dispensed (75 µl per well) to a round-bottom 96-well plate (Sarstedt 83.3925.500), and incubated at 37 °C for 24 h. DMSO stocks of Compound N1 and E4 were serially diluted in the growth medium in two separate 96-well plates (Sarstedt 83.3925.500). The contents of the two plates were mixed and incubated briefly (5 min) at 37 °C before being transferred to the cells (75 µl per well), resulting in a final density of 2.5 × 10^5^ cells ml^−1^ per well. Cells treated with purified SC2S dimers (25 µM) served as the positive control. All test compounds diluted in the growth medium were sterilized by filtering through 0.22 µm PES membranes (Merck SLGP033RS). In all cases, the final concentration of DMSO did not exceed 0.5% (v/v). Treated cells were incubated at 37 °C for 6 h and pelleted by centrifugation (120*g*, 10 min). The supernatants were collected and immediately analysed for interferon-β concentrations by enzyme-linked immunosorbent assay (ELISA; PBL assay science 41435) following the manufacturer’s instructions. The signal strength of cells treated with 25 µM of purified SC2S dimer was defined as 100% response. All other wells were reported as percentage activation and plotted on a heatmap (GraphPad Prism) accordingly.

### β-Glucuronidase-specific STING activation by the β-GlcA-N1/E4 pair

THP-1 Lucia ISG cells were maintained as described above. On the day of the experiment, cells were pelleted (150*g*, 10 min) and resuspended in the growth medium (40% conditioned) at a density of 1 × 10^6^ cells ml^−1^. Compound β-GlcA-N1, E4 and E4-ctrl were prepared as DMSO stock solutions. β-Glucuronidase (Sigma-Aldrich G8295) was reconstituted in PBS (25,000 units ml^−1^). Various test groups (group A: 50 µM β-GlcA-N1, 50 µM E4, 500 units ml^−1^ β-glucuronidase; group B: 50 µM β-GlcA-N1, 50 µM E4-control, 500 units ml^−1^ β-glucuronidase; group C: 50 µM β-GlcA-N1, 50 µM E4-ctrl; group D: 500 units ml^−1^ β-glucuronidase) were prepared, incubated at 37 °C for 1 h, sterile-filtered through 0.22-µm PES membranes (Merck SLGP033RS) and combined with the cell suspension at an equal volume (1 + 1 ml) on a 48-well plate (Greiner 677102). The final DMSO content of each well did not exceed 0.5%. Treated cells were incubated at 37 °C for 24 h and pelleted by centrifugation (300*g*, 10 min). The supernatants were collected, aliquoted and frozen at −20 °C for future analysis. The concentrations of cytokines from three biological replicates were quantified by ELISA (CXCL-10: Bio-Techne QK266; IL-6: Bio-Techne D6050B; IFN-β: PBL assay science 41435). Data were analysed by two-tailed Welch’s *t*-test (GraphPad Prism).

### Western blots of STING pathway activation in THP-1 Lucia ISG cells

THP-1 Lucia ISG cells were maintained as described above. On the day of the experiment, cells were collected by centrifugation (150*g*, 10 min) and resuspended in fresh growth medium at a density of 2 × 10^6^ cells ml^−1^. Cells were treated with cycloheximide (50 µg ml^−1^) and incubated for 50 min at 37 °C. Treated cells were then combined with the growth medium containing SC2S dimer (5 µM final concentration) to 1 × 10^6^ cells ml^−1^ and incubated at 37 °C. At each timepoint, a portion (3 ml) of the cell suspension was withdrawn. Withdrawn cells were pelleted (300*g*, 3 min), washed with PBS (1 ml), pelleted again (300*g*, 3 min) and resuspended to a density of 1 × 10^7^ cells ml^−1^ in ice-cold lysis buffer (25 mM Tris pH 7.6, 150 mM NaCl, 1% NP-40, 1% sodium deoxycholate and 0.1% SDS) supplied with protease/phosphatase inhibitor tablets (Pierce A32961) and freshly prepared phenylmethylsulfonyl fluoride (1 mM). Lysates were further disrupted by sonication at low power (5 s × 3 cycles). Sonicated lysates were clarified by centrifugation at 4 °C (13,000*g*, 5 min). The supernatants were collected, and the total protein concentrations were determined by BCA assay (Pierce 23225). Samples were reduced and denatured at 95 °C for 5 min before being separated on a precast polyacrylamide gel (Invitrogen NuPAGE 4–12% Bis-Tris, 20 µg total protein per lane). Protein bands were then transferred to a polyvinylidene difluoride membrane (Invitrogen iBlot2). The membrane was incubated in blocking buffer (5% bovine serum albumin (BSA) in TBST pH 7.4) on a shaker at room temperature for 1 h and probed by the respective primary antibody (anti-pSTING: Cell Signaling Technology E9A9K, 1:1,000 dilution; anti-pIRF3: Cell Signaling Technology E7J8G, 1:1,000 dilution; anti-pTBK1: Cell Signaling Technology D52C2, 1:1,000 dilution; anti-β-actin: Cell Signaling Technology 13E5, 1:1,000 dilution) at 4 °C for 15 h. The membrane was washed three times with TBST and incubated with HRP-conjugated secondary antibody (anti-rabbit IgG, Cell Signaling Technology, 1:1,000 dilution) for 1 h at room temperature. After being washed three times with TBST, the membrane was treated with ECL reagent (Pierce 32106) and imaged using automatic exposure (Bio-Rad ChemiDoc MP).

### Western blots of β-glucuronidase expression in CT26 mouse tumour models

Tumour samples isolated from the mice were homogenized and lysed in RIPA buffer containing protease inhibitor (Roche), phosphatase inhibitors (Sigma) and 0.1% Benzonase (Sigma) on ice for 30 min. The lysates were centrifuged at 21,000*g* for 15 min at 4 °C. The supernatant was collected, and the total protein concentrations were determined using BCA assay (Sigma). The supernatants were separated on 12% SDS–PAGE gels (30 µg total protein per lane) and transferred to polyvinylidene difluoride membranes (GE Healthcare). Membranes were then blocked with 5% BSA in TBST for 1 h at room temperature and then probed with the primary antibodies (anti-GUSB: Proteintech 16332-1-AP, 1:1,000 dilution; anti-Vinculin, Cell Signaling Technology #18799, 1:1,000 dilution) at 4 °C overnight. The membranes were then washed three times with TBST and incubated with the HRP-conjugated secondary antibody (goat anti-mouse IgG, Abcam ab205719, 1:10,000 dilution) for 1 h at room temperature. The membranes were then washed three times and imaged using ECL substrate (BioRad #170-5060) and Amersham 800 Imaging System (Cytiva).

### Zebrafish care and handling

The zebrafish (*Danio rerio*) model was handled and maintained according to the standard protocols of the European Animal Welfare Legislation, Directive 2010/63/EU (European Commission, 2016) and Champalimaud Fish Platform. All protocols were approved by the Champalimaud Animal Ethical Committee and Portuguese institutional organizations—ORBEA (Órgão de Bem-Estar e Ética Animal/Animal Welfare and Ethics Body) and DGAV (Direção Geral de Alimentação e Veterinária/Directorate General for Food and Veterinary).

### Zebrafish transgenic and mutant lines

Depending on the purpose of each experiment, different genetically modified zebrafish lines were used in this study: Tg(mpeg1:mcherry-F; tnfa:GFP-F)^41^, Tg(mpeg1:mcherry-F)^41^ and mutants casper and nacre^61^.

### Hs578T cell labelling

TNBC cell line Hs578T was cultured and expanded in Dulbecco’s modified Eagle medium (DMEM) high glucose (Biowest) supplemented with 10% FBS (Sigma-Aldrich), 100 U ml^−1^ of penicillin–streptomycin (Hyclone) and supplemented with insulin at 10 μg ml^−1^ (Sigma-Aldrich). Hs578T cells were cultured in a humidified atmosphere containing 5% CO_2_ at 37 °C. Hs578T were authenticated through short tandem repeat profile analysis and tested routinely for mycoplasma contamination. Hs578T cells were grown to 70% confluence, washed once with Dulbecco’s PBS (Biowest) and detached with EDTA (1 mM) by scrapping. Cell suspension was collected to a microcentrifuge tube, mixed with lipophilic dye Deep Red Cell Tracker (1 μl ml^−1^ of cell suspension, 10 mM stock) (Life Technologies) for 10 min at 37 °C in darkness and washed with Dulbecco’s PBS. Cells were centrifuged at 250*g*, for 4 min at 4 °C, and resuspended in DMEM. Cell viability was assessed by the trypan blue exclusion method, and cell number was determined by haemocytometer counting. Cells were resuspended in growth medium to a final density of 5 × 10^5^ cells μl^−1^. Fluorescently labelled Hs578T cells were injected using borosilicate glass microcapillaries under a fluorescence scope (Zeiss Axio Zoom. V16) equipped with a mechanical micropipette (World Precision Instruments, Pneumatic Pico pump PV820). Cells were injected into the PVS of 2 dpf zebrafish embryo previously anaesthetized with 1× Tricaine (Sigma-Aldrich). After the injection, zebrafish xenografts were left for 10 min in 1× Tricaine, transferred to E3 medium and kept at 34 °C. At 1 dpi, zebrafish xenografts were screened for the presence of tumoural mass. Xenografts with cells in the yolk sac or cellular debris were discarded, while the successful ones were grouped according to the sizes of their tumours using the size of their eyes as a scale. The xenografts were then exposed to E2 media containing DMSO or the test compounds. Xenografts were checked daily; dead ones were removed, and the E2 medium with DMSO or compounds was refreshed. Four days later, the zebrafish xenografts were euthanized, fixed overnight with 4% (v/v) formaldehyde (Thermo Scientific) at 4 °C and preserved at −20 °C in pure methanol. For transgenic zebrafish line Tg(mpeg1:mcherry-F; tnfa:GFP-F), fixation was performed with PIPES for optimal fluorescence signal preservation.

### Zebrafish xenograft live imaging

At 2 dpi (1 dpt), controls and treated Tg(mpeg1:mcherry-F; tnfa:GFP-F) xenografts were carefully selected under a fluorescent scope to ensure that only the double-positive transgenics were imaged. Selected xenografts were anaesthetized and mounted onto a coverslip with 0.8% low-melting agarose with 1× Tricaine in E2 medium. The mounted xenografts were imaged using a LSM 980 Upright confocal laser scanning microscope, with a 25× water objective lens. Then, *z*-stack images of the tumours were obtained within a 3-µm interval. At the end of the acquisition, xenografts were carefully recovered and returned to the initial conditions (DMSO, MSA2, SC2S, N1/E4 and β-GlcA-N1/E4). At 3 dpt, the same xenografts were imaged once again. Image analysis was performed using FIJI software (ImageJ 2.14).

### Zebrafish xenograft imaging and analysis

Fixed zebrafish xenograft images were acquired using a LSM 980 Upright confocal laser scanning microscope, with a 5-μm interval. Generated images were processed using the FIJI/ImageJ software. The number of cells was quantified using the Cell Counter plugin in ImageJ software. To assess tumour size, three representative slices of the tumour, from the top (*Z*_first_), middle (*Z*_middle_) and bottom (*Z*_last_), per *z* stack per xenograft were analysed, and a proxy of total cell number of the entire tumour (4′,6-diamidino-2-phenylindole (DAPI) nuclei) was estimated as follows: tumour size = (total number of slices/1.5) × (*Z*_first_ + Z_middle_ + Z_last_)/3. The correction factor of 1.5 was estimated based on human cells with nuclei averaging 10–12 μm in diameter. The number of activated caspase-3-positive cells, macrophages, M1 and M2-like TNF-positive macrophages were counted in every slide along the tumour. The transgenic xenografts negative for TNF were used to quantify TAMs and phagocytosis. To get the percentage of each population of M1 and M2-like, the obtained number was divided by its corresponding tumour size.

### Whole-mount immunofluorescence

Xenografts stored in pure MeOH were rehydrated by a series of decreasing MeOH concentrations (75%, 50%, 25%, v/v MeOH diluted in PBS/0.1% Triton, w/v). Xenografts were then permeabilized with 0.1% (w/v) Triton in PBS and blocked in a mixture of 1× PBS, BSA, DMSO, Triton 1% (w/v) and goat serum for 1 h at room temperature. The xenografts were then probed with primary antibodies (rabbit anti-cleaved Caspase-3, Cell Signaling Technologies, 1:100 dilution; rabbit anti-mCherry, Abcam, 1:100 dilution; mouse anti-GFP, Roche, 1:100 dilution) overnight and incubated with secondary antibodies (Alexa goat anti-rabbit 594, Thermo Scientific, 1:400 dilution; Alexa goat anti-mouse 488, Thermo Scientific, 1:400 dilution) and DAPI (50 μg ml^−1^) at 4 °C overnight. The xenografts were washed, fixed and mounted between two coverslips, allowing for double side acquisition using Mowiol mounting media (Sigma).

### Zebrafish experiment statistical analysis

Statistical analysis was performed using the GraphPad Prism 9.0 software. All datasets were challenged by D’Agostino and Pearson and Shapiro–Wilk normality tests. In general, datasets with a Gaussian distribution were analysed by parametric unpaired *t*-test, and datasets that did not pass the normality tests were analysed by nonparametric unpaired Mann–Whitney test. Clearance datasets were analysed using Fisher’s exact test. All tests were two-sided with a confidence interval of 95%.

### Maintenance of cell lines for mouse experiments

Mouse colon adenocarcinoma MC38 cells were purchased from Kerafast (ENH204-FP). The cells were cultured in DMEM (Gibco, Thermo Scientific #21969035), supplemented with 10% heat-inactivated FBS (Gibco, Thermo Scientific), 1× GlutaMAX (Gibco, Thermo Scientific) and 1× penicillin–streptomycin solution (Gibco, Thermo Scientific). Murine colon adenocarcinoma cell line CT26-overexpressing mouse β-glucuronidase enzyme (CT26mβGUS) was a gift from Dr Steve R. Roffler from the Institute of Biomedical Sciences, Academia Sinica, Taiwan. The cells were cultured in RPMI 1640 medium (Gibco, Thermo Scientific) supplemented with 10% heat-inactivated FBS (Gibco, Thermo Scientific), 1× GlutaMAX (Gibco, Thermo Scientific) and 1× penicillin–streptomycin solution (Gibco, Thermo Scientific). Both cell lines were cultured at 37 °C under a humidified atmosphere containing 5% CO_2_.

### Mouse tumour models

All animal experiments were conducted at the Gulbenkian Institute for Molecular Medicine (GIMM, Lisbon). Animal work was performed with strict adherence to the Portuguese Law (Portaria 1005/92) and the European Guideline 86/609/EEC. The Federation of European Laboratory Animal Science Associations guidelines and recommendations concerning laboratory animal welfare were followed. All animal experiments were approved by the Portuguese official veterinary department for welfare licensing – Direção Geral de Alimentação e Veterinária (DGAV) and the IMM Animal Ethics Committee (authorization AWB_2021_03_GB_TargCancerDrugs). Eight-week-old male and female C57BL/6J or BALB/c mice (purchased from Charles River) were subcutaneously inoculated in the right flank with 100 μl of 1 × 10^6^ MC38 cells or 5 × 10^6^ CT26mβGUS, respectively, in a 1:1 mixture of Dulbecco’s modified Eagle medium (Gibco) with Matrigel (Corning). Tumour growth was monitored over time by performing daily bilateral vernier caliper measurements. Tumour volumes were estimated with the formula: (length × width^2^)/2. Treatments were initiated when tumours reached 90–100 mm^3^. Treatments were randomly assigned to mice according to tumour volumes. Treatments were administered intratumourally (or intraperitoneally for component β-GlcA-N1). Animals were observed every 1–2 days and euthanized when either the tumour volume reached ~1,000 mm^3^, the body weight loss exceeded 20% or ulceration started to appear. For immune characterizations, inflammatory cells were isolated from the tumours, spleens and inguinal lymph nodes 24 h after the second treatment. Blood was collected and sera was obtained for cytokine profiling. Collected data were analysed with GraphPad Prism 9.

### Multiplexed analysis of cytokines

This study used Luminex xMAP technology for multiplexed quantification of 44 mouse cytokines, chemokines and growth factors. The multiplexing analysis was performed using the Luminex 200 system (Luminex) by Eve Technologies Corp. Forty-five markers were simultaneously measured in the samples using Eve Technologies’ Mouse Cytokine 44-Plex Discovery Assay, which consists of two separate kits, one 32-plex and one 12-plex (MilliporeSigma), following the manufacturer’s protocol. The 32-plex consisted of eotaxin, G-CSF, GM-CSF, IFNγ, IL-1α, IL-1β, IL-2, IL-3, IL-4, IL-5, IL-6, IL-7, IL-9, IL-10, IL-12(p40), IL-12(p70), IL-13, IL-15, IL-17, IP-10, KC, LIF, LIX, MCP-1, M-CSF, MIG, MIP-1α, MIP-1β, MIP-2, RANTES, TNF and VEGF. The 12-plex consisted of 6Ckine/Exodus2, erythropoietin, fractalkine, IFNβ-1, IL-11, IL-16, IL-20, MCP-5, MDC, MIP-3α, MIP-3β and TARC. Assay sensitivities of these markers range from 0.3 to 30.6 pg ml^−1^ for the 45-plex. The sensitivities of individual analytes are available in the MilliporeSigma MILLIPLEX MAP protocol.

### LC–MS/MS analysis of in situ SC2S formation

Tumour-bearing mice treated with β-GlcA-N1(IP) + E4 (IT) were euthanized at 1, 2, 4, 8, 24 and 72 h posttreatment (*n* = 3 for each timepoint). The blood, tumour, lung, kidney, liver and spleen were collected for each mouse. Three mice treated with vehicle were included as control samples. Whole blood samples were centrifuged at 2,000*g*, 4 °C for 10 min. Supernatant plasma was aspirated into an Eppendorf tube and frozen at −80 °C. Tumour and organ tissues were snap-frozen in liquid nitrogen and stored at −80 °C until further analysis. The plasma samples (10-µl aliquots) were prepared by protein precipitation extraction. The organic layer was evaporated to dryness and reconstituted in a solution equivalent to the initial chromatographic starting conditions. The tissue samples were analysed by homogenization to give a 200 ng ml^−1^ homogenate concentration. The homogenised tissue samples (10-µl aliquots) were then taken through the same preparation procedure as the plasma samples. All samples were analysed using the PKB Core Facility’s 6500 LC–MS/MS system, in negative ion mode. The chromatography was carried out using a gradient method on a Kinetex EVO C18 column (50 × 2.1 mm). Analysis was conducted in four batches, consisting of calibration standards, quality control samples, blank control samples, internal standard-only samples and study samples. Calibration curves were constructed using linear regression with a 1/*x*^2^ weighting. A calibration range of 1–500 ng ml^−1^ (5–2,500 ng g^−1^ in tissue) was used. Precision and accuracy were well within the PK/B core facility’s acceptance criteria of ±20%. The LC–MS/MS method was developed and qualified ahead of the sample analysis. The performance of the method met normal Core Facility acceptance criteria for analysis.

### X-ray crystallography

The 8xHis-tagged R232-STING_138–379_ construct was expressed using vector pEXP-MBP (Addgene 112568). *E. coli* BL21(DE3) cells, harbouring the recombinant plasmid, were cultivated in 2xYT medium (supplemented with 0.1 mg ml^−1^ ampicillin) at 37 °C until OD_600_ reached ~0.8 and then cooled to 18 °C for 30 min. Isopropyl 1-thio-d-galactopyranoside was added to 0.4 mM, and growth continued at 18 °C for 16 h. Cells were pelleted by centrifugation and resuspended in lysis buffer (30 mM HEPES buffer, pH 7.5, 0.5 M NaCl,10 mM imidazole, 0.5 mM TCEP, 5% glycerol and 100 mM phenylmethylsulfonyl fluoride) before flash-freezing in liquid nitrogen. After 1 day, cells were thawed and lysed by sonication on ice (5 s ON, 10 s OFF, 5 min total ON). Proteins were purified using Ni-NTA resin (Qiagen) and eluted stepwise in binding buffer with 300 mM imidazole. Removal of histidine tag was performed at 4 °C overnight using recombinant TEV protease (at 1:20 molar ratio) while dialysing (SnakeSkin Dialysis Tubing, 10 kDa cut-off weight, Thermo Fisher Scientific) against gel filtration buffer (10 mM HEPES pH 7.5, 150 mM NaCl, 0.5 mM TCEP and 5% glycerol). Proteins were further purified by reverse affinity in Ni-NTA followed by gel filtration (Superdex 200 16/60, GE Healthcare). Protein in gel filtration buffer was concentrated to 6.7 mg ml^−1^ using 10 kDa MWCO centrifugal concentrators (Millipore) at 5 °C. Compounds in 100% DMSO were added to protein (247 µM final solution) at 2.2 mM final concentration (~2.2% DMSO) and incubated on ice for approximately 1 h. This mixture was centrifuged at maximum speed for 10 min at 5 °C before setting up 200-nl sitting drops with protein–inhibitor complex and reservoir solution at ratios of 1:1 and 1:2. Crystallization experiments were performed at 20 °C. Crystals were flash-cooled in liquid nitrogen for data collection. Diffraction data were collected at the Diamond Light Source at the I04 beamline. The best-diffracting crystals grew under the conditions described in the table below. Diffraction data were integrated using XDS (within autoPROC)^62^ and scaled using AIMLESS from the CCP4 software suite^63^. Molecular replacement and initial refinement were performed using Dimple (PDB ID 4KSY was used as the molecular replacement search model for phasing). Follow-up refinements and manual model adjustments were conducted using BUSTER^64^ and Coot^65^, respectively. Structure validation was performed using MolProbity^66^. The final model and structure factors were deposited in the PDB (PDB ID: 9QVT).

### Reporting summary

Further information on research design is available in the Nature Portfolio Reporting Summary linked to this article.

## Online content

Any methods, additional references, Nature Portfolio reporting summaries, source data, extended data, supplementary information, acknowledgements, peer review information; details of author contributions and competing interests; and statements of data and code availability are available at 10.1038/s41557-025-01930-9.

## Supplementary information

Supplementary Information Supplementary Figs. 1–6, synthetic schemes, synthetic procedures, NMR spectra, high-resolution mass spectrometry spectra and supplementary source data.

Reporting Summary

## Source data

Source Data Fig. 2 Statistical source data.

Source Data Fig. 3 Statistical source data.

Source Data Fig. 3 Original blot. Statistical source data.

Source Data Fig. 4 Statistical source data.

Source Data Fig. 5 Statistical source data.

Source Data Fig. 6 Statistical source data.

**Extended Data Fig. 1:**
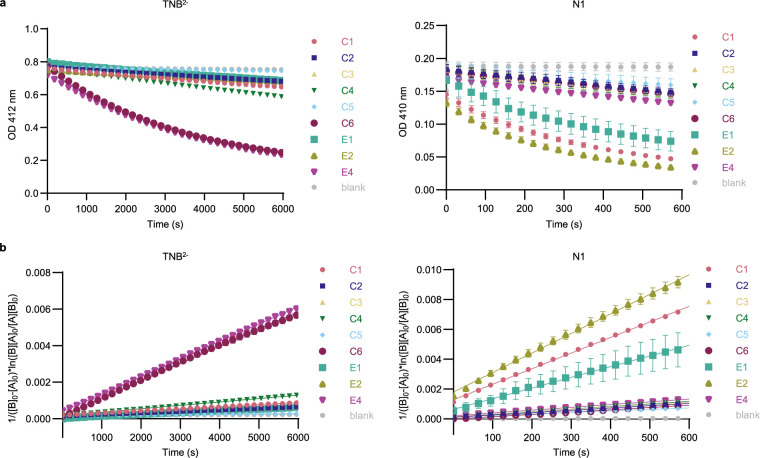
Original absorbance data for deriving second order alkylation rate constants. **a** Absorbance time-course data. **b** Linearized second-order rate plots. [A]_0_ = 100 μM, initial concentration of the nucleophile; [B]_0_ = 250 μM, initial concentration of the electrophile. Each data point is presented as mean ± STD from three independent measurements. Validity of the assay was confirmed by LC-MS analysis of selected reactions at the end of the assay, which showed the alkylated thiol as the sole product. See Supplementary Fig. 2 for details. Source data

**Extended Data Fig. 3:**
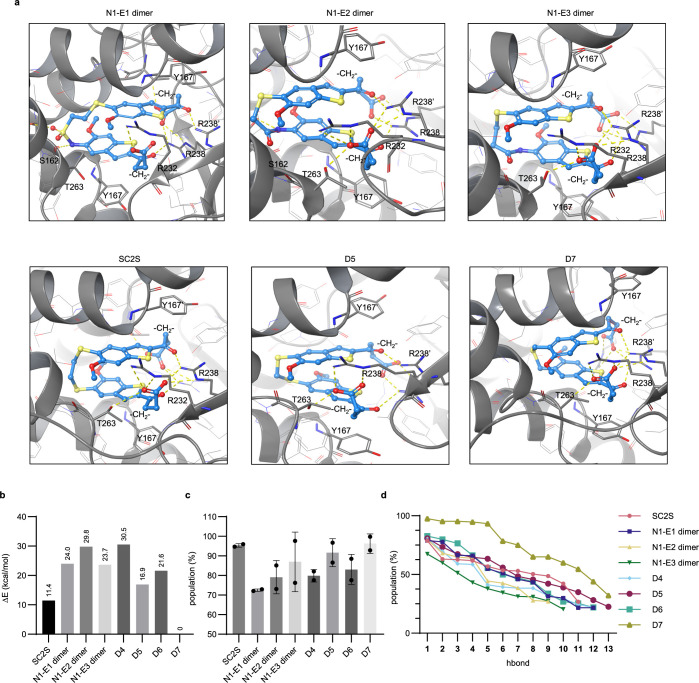
Molecular dynamics (MD) simulations of STING complexed with different dimers. **a** Representative frame from 1 µs MD simulations. The key interacting residues (S162, T263, Y167, R232, and R238) are highlighted. Hydrogen bonds are depicted as yellow dashed lines. **b** Theoretical binding energy (relative to compound D7) of each dimer calculated using the MM-PBSA method. See the Method section for details. **c** Distance between the aromatic ring center of Y167 and the carbon atom of the methylene group (CO-CH_2_-) of the dimers over the course of the simulation, reflecting the stability of this interaction. **d** Population of hydrogen bonds observed in the MD simulation trajectory of each complex.

**Extended Data Fig. 4:**
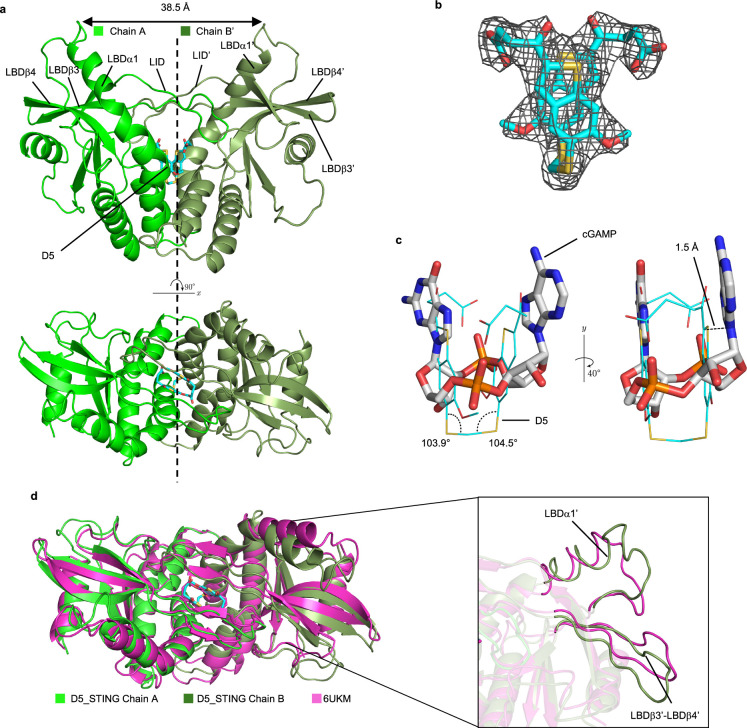
Additional structural analysis of D5-bound hSTING LBD. **a** Cartoon representation of the dimeric crystal structure of STING LBD bound to compound D5 at 2.31 Å resolution, followed by a 90° rotated view. The displayed distance was measured between the α-carbon (Cα) of Y186 in both chains (as spheres). Superposition of apo (PDB: 4EMU) and D5-bound (PDB: 9QVT) STING structures, aligned by chain A and shown in cartoon representation. The distances displayed were calculated between the Cα atoms of residue Y186 in chains A and B (represented as spheres). **b** Electron density map (2Fo-Fc, contoured at 1.0 σ) of the ligand, overlaid with its stick representation. **c** Superposition of D5- and 2’,3’ cGAMP-bound STING LBD crystal structures, emphasizing the angle formed by the C–S–C bonds in the linker region of D5. In both views, it is apparent that D5 penetrates deeper into the binding pocket and is also narrower at the base of the site compared to 2’,3’-cGAMP. **d** Superposition of D5- (PDB: 9QVT) and MSA2-bound (PDB: 6UKM) STING LBD crystal structures, highlighting conformational differences in chain B when chains A are aligned.

**Extended Data Fig. 5:**
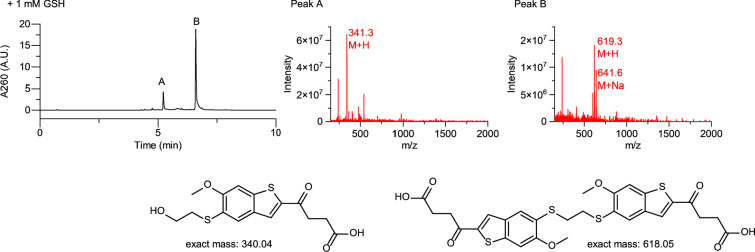
LC-MS analysis of the reaction between N1 and E4 in the presence of glutathione (1 mM). N1 (50 μM) and E4 (50 μM) were reacted at neutral pH (25 mM HEPES pH 7.0, 150 mM NaCl) and in the presence of reduced glutathione (1 mM). The reaction mixture was incubated at 37 °C for 2 h. No glutathione-adduct of E4 was identified.

**Extended Data Fig. 6:**
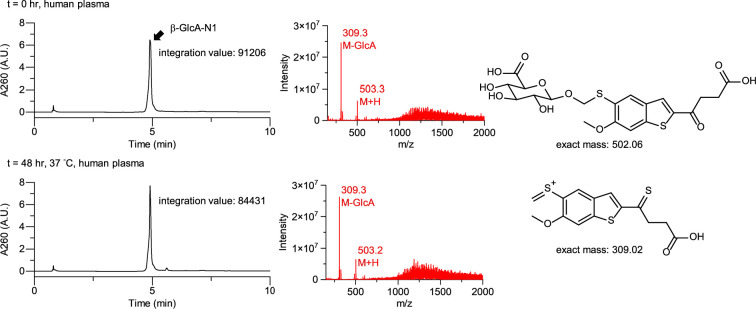
Stability test of β-GlcA-N1 (1 mM) in human plasma. At the indicated time point, an 80 µL aliquot was loaded onto an Agilent Bond Elut solid phase extraction column (C18, 3 mL bed volume). The collected eluent was lyophilized, redissolved in a 50% (v/v) acetonitrile in water (0.1% v/v formic acid), and analyzed by LC-MS.

Source Data Extended Data Fig. 1 Statistical source data.

**Extended Data Fig. 7:**
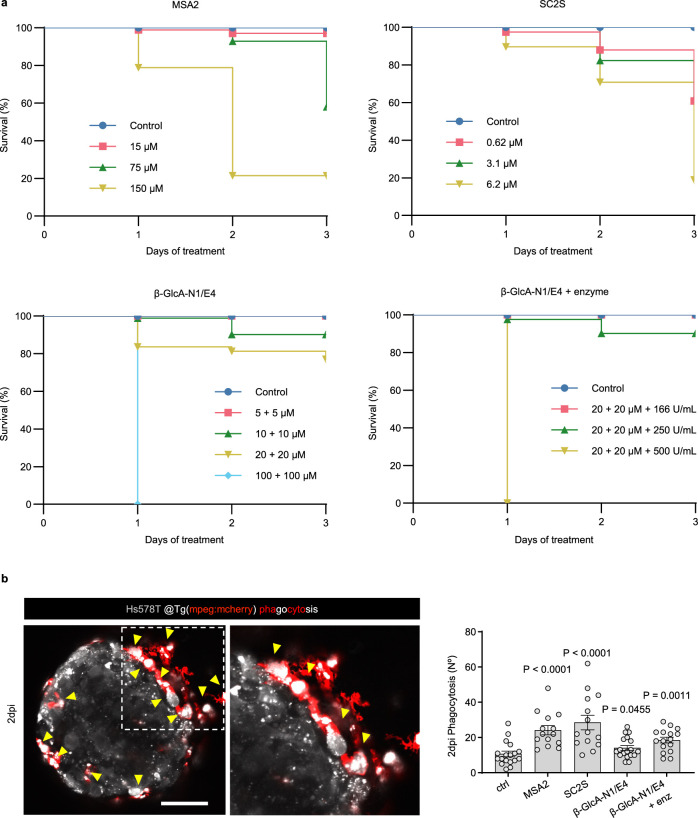
Additional zebrafish experiment data. **a** MTC of β-GlcA-N1/E4 treatments on non-tumor zebrafish larvae. Groups of 30 noninjected zebrafish larvae were exposed to different concentrations of the compounds (MSA2, SC2S, β-GlcA-N1/E4 and β-GlcA-N1/E4 + β-glucuronidase) for 3 consecutive days, with daily renewal of the E2 media containing the drugs. The tested concentrations of the compounds, in different combinations, were the following: MSA2: 15 μM, 75 μM and 150 μM; SC2S: 0.62 μM, 3.1 μM and 6.2 μM; β-GlcA-N1/E4: 5 μM + 5 μM, 10 μM + 10 μM, 20 μM + 20 μM and 100 μM + 100 μM; β-GlcA-N1/E4 + β-glucuronidase: 20 μM + 20 μM + 166 U/mL, 20 μM + 20 μM + 250 U/mL and 20 μM + 20 μM + 500 U/mL. Toxicity was assessed daily by counting the total number of dead larvae and checking for the presence of morphologic changes such cardiac edemas or curved tails. Control groups were treated with the highest matched DMSO concentration (0.04%, v/v). **b** Left: representative image of phagocytosis in a xenograft treated with β-GlcA-N1/E4 and β-glucuronidase at 2dpi in Tg(mpeg1:mcherry-F). Each yellow arrow indicates a phagocytic event. Right: n° of phagocytic events at 2dpi. Data are presented as mean ± SEM. Statistical analysis follows that of Fig. 5. Source data

Source Data Extended Data Fig. 7 Statistical source data.

**Extended Data Fig. 8:**
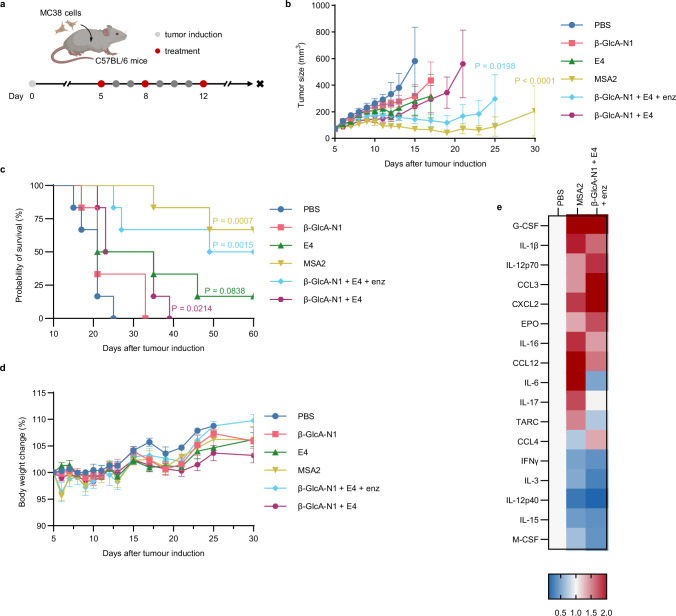
β-GlcA-N1/E4 tested on a MC38 syngeneic mouse model. **a** Experimental design. MC-38 cells were inoculated into the right flank of C57BL/6 mice and treated once tumors reached about 90 mm^3^. MSA2: 5 mg/kg. β-GlcA-N1: 12.5 mg/kg; E4: 5 mg/kg; N1 + E4: N1 12.5 mg/kg and E4 5 mg/kg; β-GlcA-N1/E4 + enz: 12.5 mg/kg of β-GlcA-N1 and 5 mg/kg of E4, preincubated with 500 units of *E. coli* β-glucuronidase at 37 °C for one hour. N1 was administered intraperitoneally (IP); all other compounds were administered intratumorally (IT). **b** Tumor growth curves. Data were analyzed by one-way ANOVA, followed by multiple comparisons adjusted with the Sidák-Holm correction. **c** Survival curves. Differences between the compound-treated groups and the vehicle-treated control group were analyzed by the Log-Rank test. **d** Body weight monitoring. **e** Cytokine profiling of sera obtained on Day 9. Source data

Source Data Extended Data Fig. 8 Statistical source data.

**Extended Data Fig. 9:**
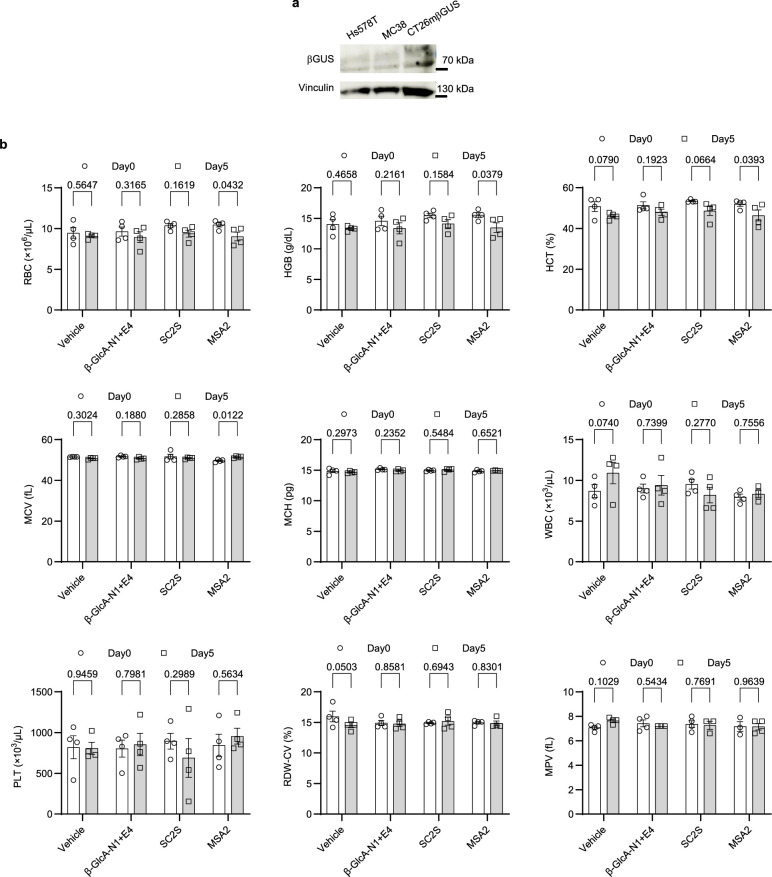
Additional data of CT26mβGUS mouse tumor model experiment. **a** Confirmation of β-glucuronidase overexpression in CT26mβGUS cells by Western blots. **b** Haematotoxicity data. MCV: Mean Corpuscular Volume; MCH: Mean Corpuscular Haemoglobin; WBC: White Blood Cell Count; PLT: Platelet Count; RDW-CV: Red Cell Distribution Width - Coefficient of Variation; MPV: Mean Platelet Volume. RBC: red blood cell count; HGB: haemoglobin; HCT: haematocrit; MCV: Mean Corpuscular Volume; MCH: Mean Corpuscular Haemoglobin; WBC: White Blood Cell Count; PLT: Platelet Count; RDW-CV: Red Cell Distribution Width - Coefficient of Variation; MPV: Mean Platelet Volume. Data were analyzed by two-way ANOVA, followed by multiple comparisons adjusted with the Sidák-Holm correction. Source data

**Extended Data Fig. 10:**
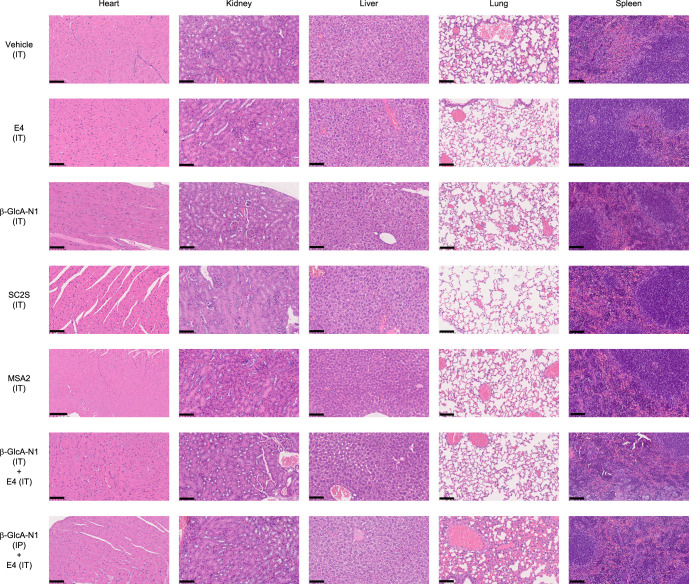
Histopathology analysis of non-targeted tissues. Organs were harvested, fixed in 10% formalin, embedded in paraffin, sectioned into 3 μm-thick sections, and stained with Hematoxylin and Eosin (H&E). Whole sections were acquired with the NanoZoomer-SQ Digital slide scanner (Hamamatsu) and pathology assessment was performed by an experimental pathologist (PF) at GIMM’s Histopathology Facility. Scale bars: 100 μm.

Source Data Extended Data Fig. 9 Original blot.

Source Data Extended Data Fig. 9 Statistical source data.

## Figures and Tables

**Extended Data Fig. 2:**
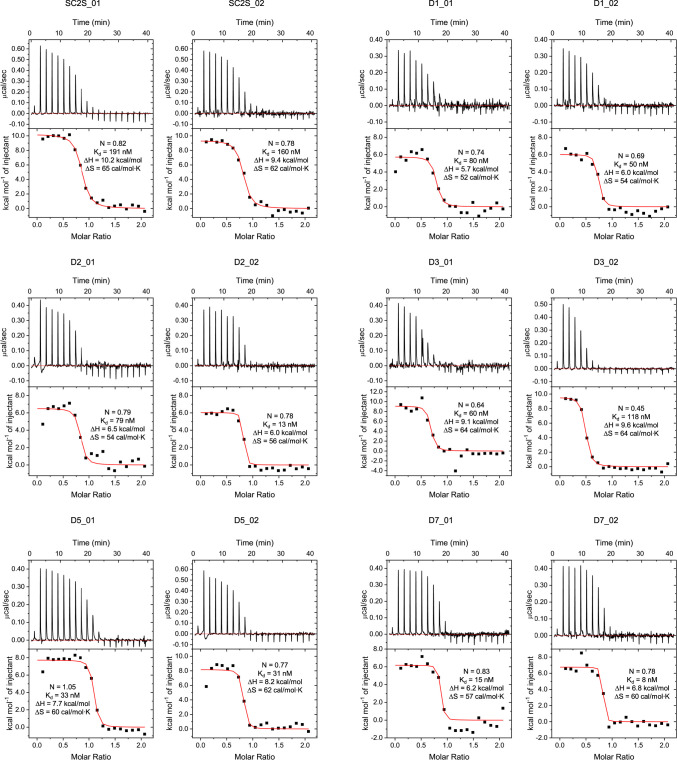
Isothermal titration calorimetry (ITC) data for binding constant measurements. Purified hSTINGwt138-379 (250 μM of homodimer) was titrated into each dimer (25 μM) dissolved in a matching buffer (25 mM HEPES pH 7.5, 150 mM NaCl) at 25 °C and 750 rpm stirring. Binding constants were reported as the average from duplicate measurements.

## Data Availability

The co-crystal structure of compound D5 and STING LBD has been deposited in the RCSB Protein Data Bank with the accession code 9QVT. Detailed synthetic procedures and compound characterization data such as NMR and high-resolution mass spectrometry spectra are available in the Supplementary Information. Source data are provided with this paper.
