# Supplementary material for: Tumour-specific STING agonist synthesis via a two-component prodrug system
Source: Nat Chem. 2025 Sep 16;17(12):1941–51. doi: 10.1038/s41557-025-01930-9 (PMC12669040; doi:10.1038/s41557-025-01930-9)
Supplement: Supplementary file 1 — Supplementary Figs. 1–6, synthetic schemes, synthetic procedures, NMR spectra, high-resolution mass spectrometry spectra and supplementary source data. [file 41557_2025_1930_MOESM1_ESM.pdf]

nature chemistry

Supplementary information https://doi.org/10.1038/s41557-025-01930-9

Tumour-specific STING agonist synthesis via a two-component prodrug system

In the format provided by the authors and unedited

Table of contents

Supplementary Figures **2**

Figure 1. LC-MS analysis of reactions between analogs N1 and E1 to E4 2

Figure 2. LC-MS analysis of selected reaction mixtures from rate constant measurements 3

Figure 3. Calibration curve for SC2S dimer quantification by HPLC 3

Figure 4. LC-MS analysis of competition experiments between E4 and electrophiles C1-6 4

Figure 5. Characterizations of purified recombinant wild-type human STING 5

Figure 6. LC-MS analysis of β-GlcA-N1/E4 reactions in the presence or absence of β-glucuronidase 5

Supplementary Tables **6**

Table 1. Crystallographic refinement statistics of Compound D5 and STING LBD. 6

Synthetic Schemes **7**

Scheme 1. Synthesis of covalent dimers D1 to D7 and SC2S 7

Scheme 2. Synthesis of analogs N1 and E1 to E4 8

Scheme 3. Synthesis of β-GlcA-N1 8

Synthetic Procedures **9**

NMR spectra **36**

HRMS spectra **84**

References **109**

Supplementary Source Data **110**

Supplementary Figures

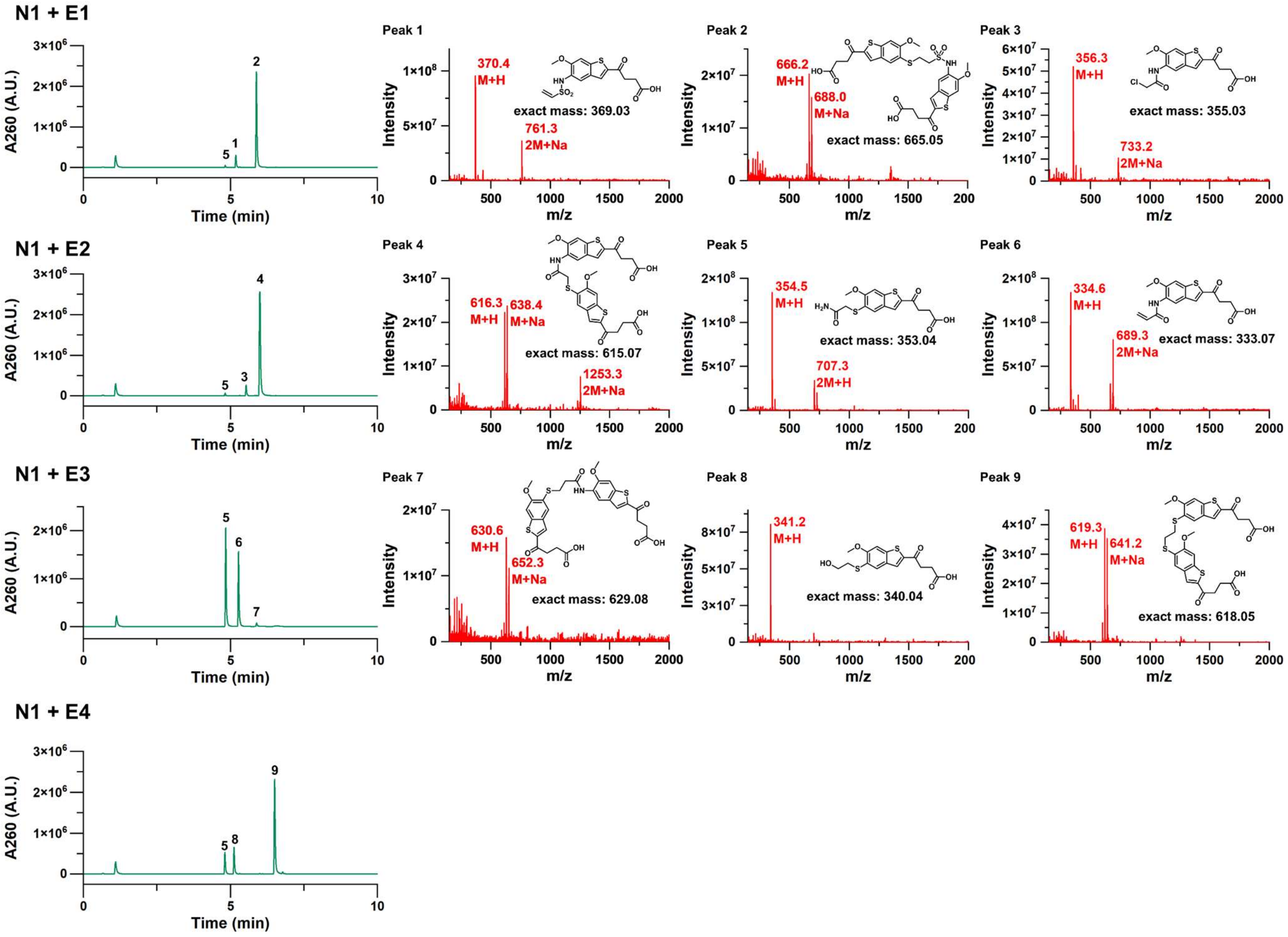

Supplementary Fig. 1 LC-MS analysis of reactions between analogs N1 and E1 to E4.

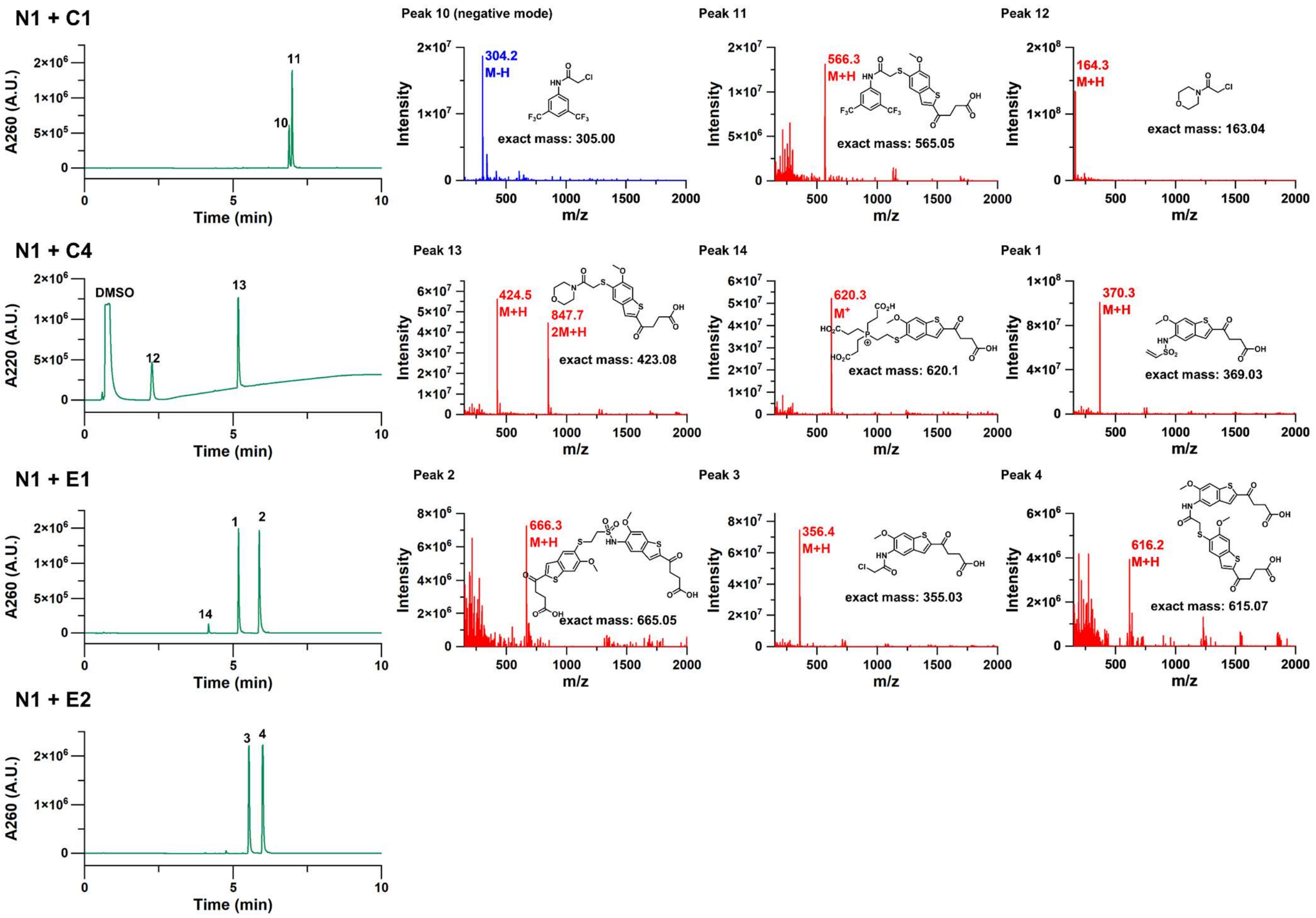

Supplementary Fig. 2 LC-MS analysis of selected reaction mixtures from rate constant measurements.

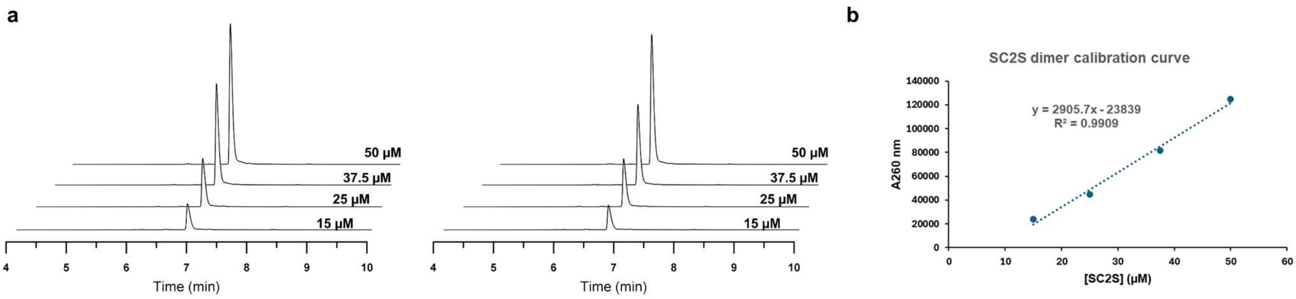

Supplementary Fig. 3 Calibration curve for SC2S dimer quantification by HPLC. a. A260 traces of purified SC2S standard diluted to different concentrations. b. Linear calibration curve constructed from duplicate injections.

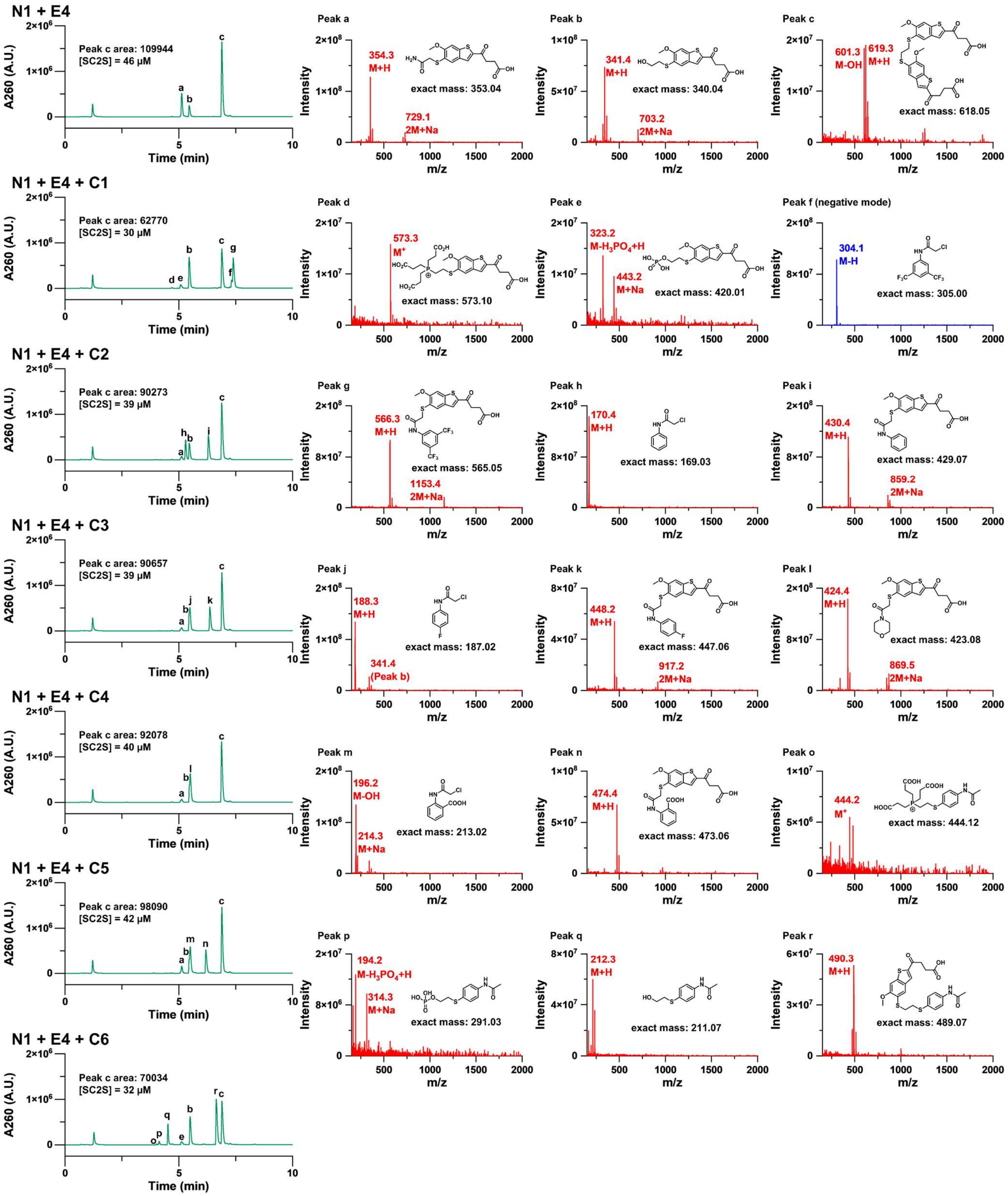

Supplementary Fig. 4 LC-MS analysis of competition experiments between E4 and electrophiles C1-6. The yield of each reaction was calculated from the integration value of Peak c using the calibration curve in Supplementary Fig 3.

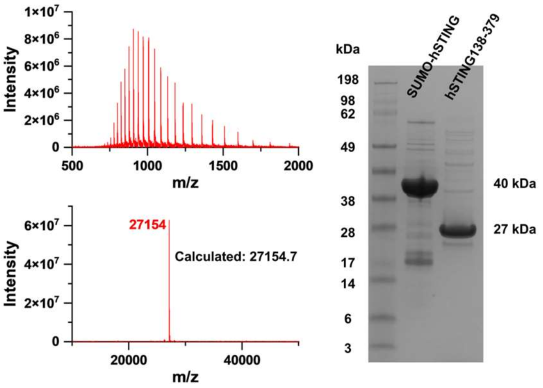

Supplementary Fig. 5 Characterizations of purified recombinant wild-type human STING (aa 138-379).

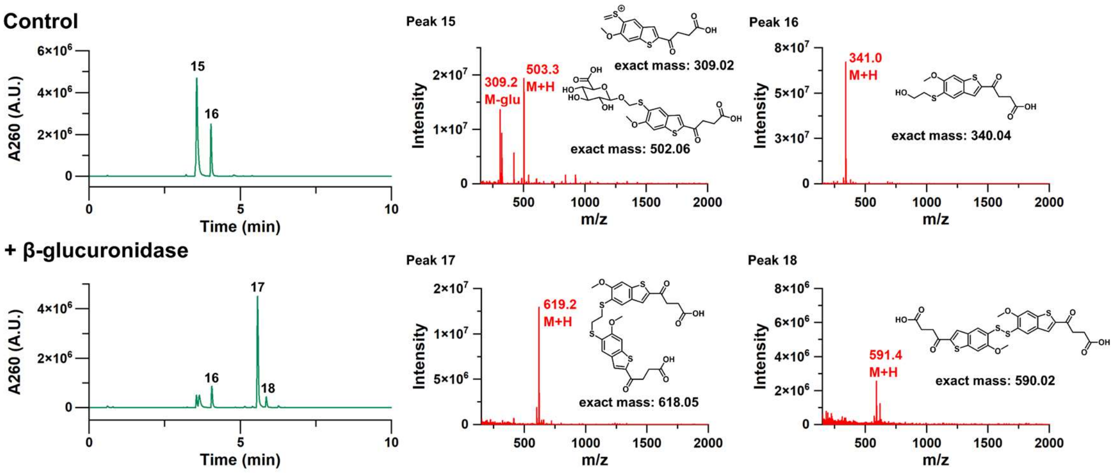

Supplementary Fig. 6 LC-MS analysis of β-GlcA-N1/E4 reactions in the presence or absence of β-glucuronidase.

Supplementary Tables

Data collection	Compound D5
PDB ID	9QVT
X-ray source	Diamond Light Source
Beamline	I04
Wavelength (Å)	0.9537
Spatial group	C2
Unit cell (Å)	89.68, 75.60, 34.14, β= 97.75
Molecules / unit cell	1
Resolution range (Å)	57.579 - 2.305
High resolution range (Å)	2.575 - 2.305
Unique reflections	6416 (321)
R_{merge}	0.095 (1.474)
Mean I/σ(I)	10.2 (1.4)
Mean CC(1/2)	0.959 (0.586)
Completeness (ellipsoidal) (%)	82.3 (40.4)
Redundancy	6.8 (7.2)
**Refinement**	
Resolution (Å)	57.579 - 2.305
RMS bond (Å)	0.56
RMS angle (º)	0.680
Average B factor (protein/solvent) ($Å^2$)	57.5 / 31.6
R_{cryst} / R_{free} (%)	34.6 / 42.4
Nº of atoms (protein/solvent)	1279/27
**Ramachandran statistics (%)**	
Favored / Allowed / Outliers (%)	90/10/0
Crystallization condition	0.1 M Bis Tris Propane pH 7.5; 0.2 M Sodium nitrate, 20% (w/v) PEG 3350, 10% Ethylene glycol

Supplementary Table. 1 Crystallographic refinement statistics of Compound D5 and STING LBD.

Synthetic Schemes

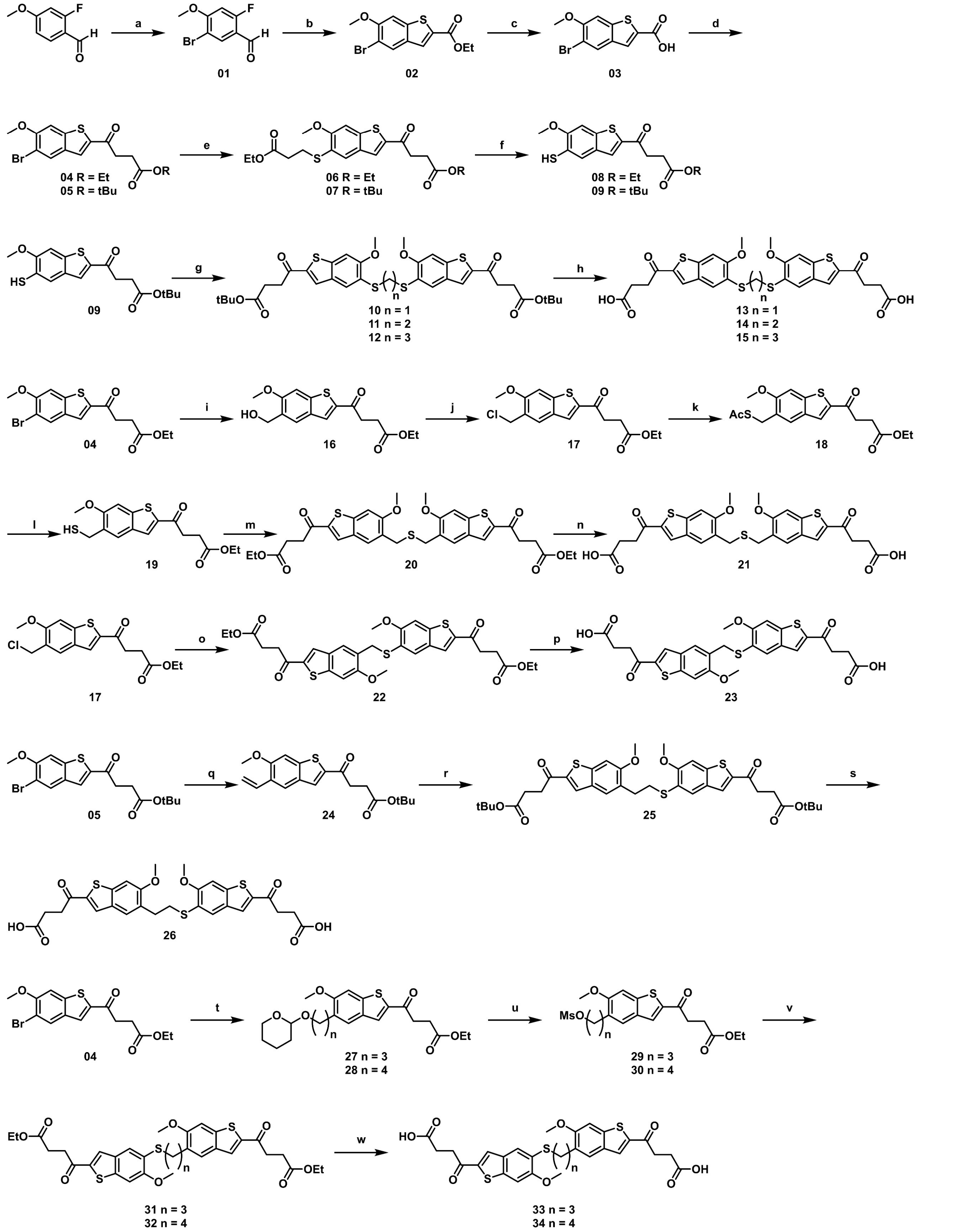

Scheme 1. Synthesis of covalent dimers D1 to D7 and SC2S. (a) Br_2, MeOH. (b) ethyl thioglycolate, K_2CO_3, DMF, 60 °C. (c) KOH, THF/H_2O, 60 °C. (d) oxalyl chloride, THF; 3-ethoxy-3-oxopropylzinc bromide, LiCl, CuI, THF for R = Et;

tert-butyl 3-bromopropanoate/Rieke zinc, LiCl, CuI, THF for R = tBu. (e) ethyl 3-mercaptopropanoate, $Pd_2(dba)_3$, Xantphos, DIPEA, 1,4-dioxane, reflux. (f) NaOEt, EtOH/THF, -20 °C. (g) DBU, bromochloromethane/toluene then DIPEA, DMF for n =1; 1,2-dibromoethane, DIPEA, DMF for n = 2; 1,3-dibromopropane, DIPEA, DMF for n = 3. (h) TFA/DCM. (i) (tributylstannyl)methanol, Xphos Pd G2, 1,4-dioxane, 90 °C. (j) MsCl, TEA, DCM. (k) potassium thioacetate, DMF, 45 °C. (l) NaOEt, EtOH/THF. (m) Compound 17, NaI, DMF. (n) LiOH, THF/H_2O. (o) Compound 4, DIPEA, DMF. (p) LiOH, THF/H_2O. (q) vinylboronic acid pinacol ester, K_3PO_4, $Pd(OAc)_2$, SPhos, THF/H_2O. (r) Compound 9, AIBN, MeOH, reflux. (s) TFA/DCM. (t) 2-(3-bromopropoxy)tetrahydro-2*H*-pyran/Rieke zinc, LiCl, Xphos Pd G4, THF, 50 °C for n = 3; 2-(4-bromobutoxy)tetrahydro-2*H*-pyran/Rieke zinc, LiCl, Xphos Pd G4, THF, 50 °C for n = 4. (u) TsOH, EtOH; MsCl, DIPEA, DCM. (v) Compound 8, DIPEA, DMF. (w) LiOH, THF/H_2O.

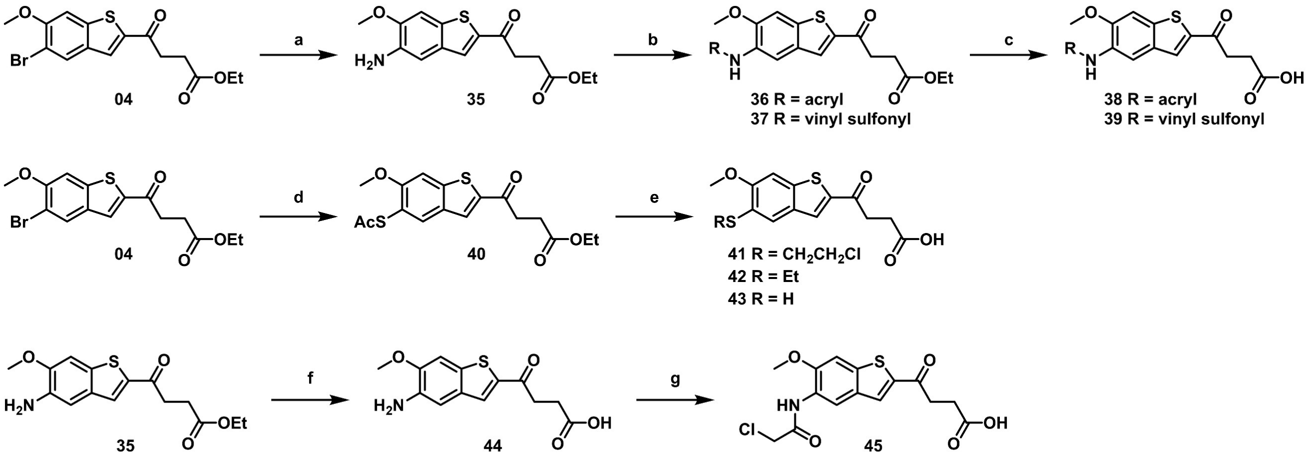

Scheme 2. Synthesis of analogs N1 and E1 to E4. (a) NaN_3, *N*,*N'*-dimethyl-1,2-cyclohexadiamine, CuI, sodium ascorbate, EtOH/water, reflux; TCEP, TEA, THF/H_2O. (b) acryloyl chloride, TEA, DCM for R = acryl; ethenesulfonyl chloride, TEA, DCM for R = vinyl sulfonyl. (c) LiOH, THF/H_2O. (d) potassium thioacetate, $Pd_2(dba)_3$, Xantphos, DIPEA, 1,4-dioxane, reflux. (e) LiOH then 1-bromo-2-chloroethane, THF/H_2O for R = CH_2CH_2Cl; LiOH then bromoethane, THF/H_2O for R = ethyl. (f) LiOH, THF/H_2O. (g) 2-chloroacetyl chloride, DIPEA, THF.

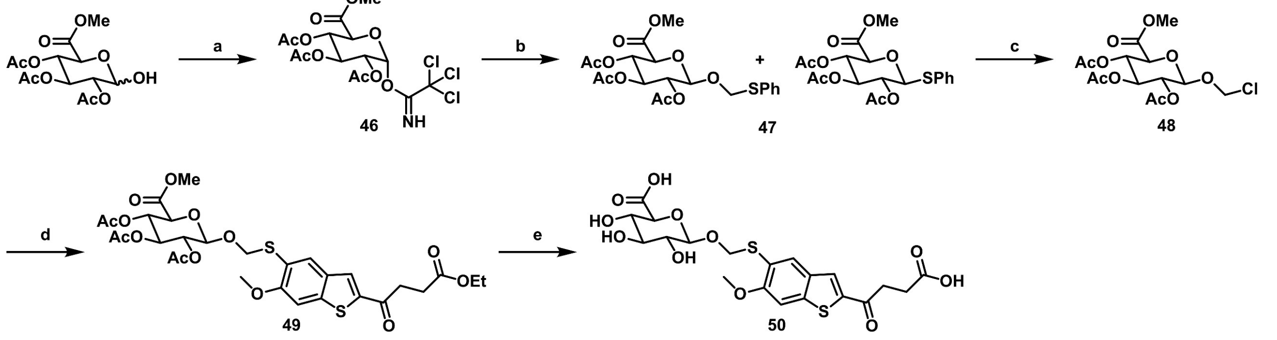

Scheme 3. Synthesis of β-GlcA-N1. **(a)** trichloroacetonitrile, DBU, DCM. **(b)** (phenylthio)methanol, AgOTf, 4Å MS, DCM. **(c)** SO_2Cl_2, DCM. **(d)** Compound 8, DIPEA, DMF. **(e)** LiOH, THF/H_2O.

Synthetic Procedures

Compound 01

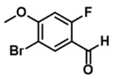

The compound was synthesized according to a published procedure.[1] 2-fluoro-4-methoxybenzaldehyde (8.4 g, 54.5 mmol) was suspended in methanol (40 mL) at 0 °C. Bromine (5.6 mL, , 17.4 g, 108.7 mmol) was added portionwise, and the reaction mixture was stirred at 0 °C for 1 hour 40 minutes. Sodium metabisulfite (21.3 g, 112 mmol) dissolved in water (280 mL) was then poured in all at once to quench the remaining bromine. The quenched mixture was stirred at room temperature for another 15 minutes. The resulting solids were then collected by vacuum filtration. The filter cake was washed with water (25 mL × 5) and dried in vacuo to afford the desired product as a light tan powder. 9.83 g, 77% yield.

HRMS (ESI/TOF) m/z: $[M+H]^+$ Calculated for $C_8H_7BrFO_2$ 232.9614; Found 232.9499 (poor ionization).

^1H NMR (500 MHz, DMSO) δ 10.02 (s, 1H), 7.97 (d, *J* = 7.5 Hz, 1H), 7.26 (d, *J* = 12.6 Hz, 1H), 3.98 (s, 3H).

^{13}C NMR (126 MHz, DMSO-d_6): δ 185.56 (d, *J* = 4.4 Hz), 164.12 (d, J = 258 Hz), 161.51 (d, *J* = 11.8 Hz), 132.63 (d, *J* = 3.9 Hz), 117.94 (d, *J* = 9.8 Hz), 106.59 (d, *J* = 3.0 Hz), 101.80 (d = 26.3 Hz), 57.65.

^{19}F NMR (470.61 MHz, DMSO-d_6): δ -118.44.

Compound 02

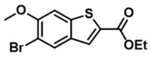

The compound was synthesized according to a published procedure.[1] Compound 01 (11.4 g, 48.9 mmol), potassium carbonate (20.2 g, 146.2 mmol), and ethyl thioglycolate (5.36 mL, 5.87 g, 48.9 mmol) were suspended in DMF (50 mL). The mixture was stirred under nitrogen at 60 °C for 18 hours. Upon completion, DMF was removed in vacuo at 55 °C. The resulting pink solid residue was dissolved in DCM (150 mL) and washed with water (150 mL × 1, 100 mL × 3). The organic layer was dried over magnesium sulfate, filtered, and concentrated in vacuo to afford the desired product as a pinkish, crystalline solid, which was used without further purification. 14.6 g, 95% yield.

HRMS (ESI/TOF) m/z: $[M+H]^+$ Calculated for $C_{12}H_{12}BrO_3S$ 316.9670; Found 316.9669 (poor ionization).

^1H NMR (500 MHz, DMSO) δ 8.26 (s, 1H), 8.04 (s, 1H), 7.78 (s, 1H), 4.33 (q, *J* = 7.1 Hz, 2H), 3.93 (s, 3H), 1.32 (t, *J* = 7.1 Hz, 3H).

^{13}C NMR (126 MHz, DMSO) δ 161.81, 154.74, 142.46, 133.21, 131.61, 129.79, 129.45, 110.52, 105.34, 61.40, 56.70, 14.17.

Compound 03

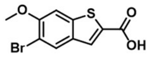

The compound was synthesized according to a published procedure.[1] To a stirred solution of Compound 02 (6 g, 19.0 mmol) in THF (50 mL) was added potassium hydroxide (3.2 g, 57.0 mmol) dissolved in water (50 mL). The reaction mixture was stirred vigorously at 60 °C for 22 hours. Upon complete consumption of the S.M. as confirmed by TLC analysis, THF was removed in vacuo, and the residual solution was acidified with citric acid (1 M aqueous) to pH 6. Additional water (50 mL) was added, and the resulting white precipitates were collected by vacuum filtration. The filter cake was washed with water (50 mL × 4) and dried in vacuo to afford the desired product as an off-white powder. 5.2 g, 95% yield.

HRMS (ESI/TOF) m/z: $[M-H-CO_2]^-$ Calculated for $C_9H_6BrO_1S$ 242.9302; Found 242.9307. $[2M-2H+Na]^-$ Calculated for $C_{20}H_{12}Br_2O_6S_2Na$ 594.8320; Found 594.8358.

^1H NMR (500 MHz, DMSO) δ 8.23 (s, 1H), 7.94 (s, 1H), 7.77 (s, 1H), 3.93 (s, 3H).

^{13}C NMR (126 MHz, DMSO) δ 163.45, 154.39, 142.39, 134.39, 133.54, 129.20, 128.80, 110.18, 105.39, 56.66.

Compound 04

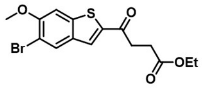

The synthesis of Compound 04 was modified from a published procedure.[1] Copper (I) iodide (135 mg, 0.71 mmol) and lithium chloride (69 mg, 1.6 mmol) in a dry two-neck was dissolved in anhydrous THF (4 mL) under nitrogen. The solution was cooled in an ice bath, and (3-ethoxy-3-oxopropyl)zinc(II) bromide (0.5 M in THF, 10.6 mL, 5.3 mmol) was rapidly added. The resulting mixture was stirred at 0 °C for another 5 minutes. 5-bromo-6-methoxybenzo[b]thiophene-2-carbonyl chloride (1.06 g, 3.47 mmol) suspended in anhydrous THF (10 + 4 mL in two portions) was then added. The reaction mixture was stirred at 0 °C for 2.5 hours. Upon completion, the mixture was diluted in DCM (150 mL) and washed with saturated ammonium chloride (150 mL × 1, 100 mL × 1). The combined aqueous layer was extracted once more with DCM (30 mL). The organic layers were combined, dried over magnesium sulfate, filtered, and concentrated in vacuo. Purification by flash column chromatography (dry-loading, 0 to 6% ethyl acetate in DCM/hexanes = 1/1) afforded the desired product as a white crystalline solid. 0.81 g, 63% yield. If desired, the product could be further purified by first dissolving in the minimum amount of refluxing ethyl acetate followed by gradual cooling to -20 °C. The recrystallized product could then be collected by vacuum filtration and washed with ice-cold ethyl acetate.

HRMS (ESI/TOF) m/z: $[M+H]^+$ Calculated for $C_{15}H_{16}BrO_4S$ 372.9932; Found 372.9950. $[M+Na]^+$ Calculated for $C_{15}H_{15}BrO_4SNa$ 394.9752; Found 394.9755. $[2M+H]^+$ Calculated for $C_{30}H_{30}Br_2O_8S_2Na$ 764.9626; Found 764.9620. $[M-OEt]^+$ Calculated for $C_{13}H_{10}BrO_3S$ 326.9514; Found 326.9600.

^1H NMR (700 MHz, $CDCl_3$) δ 8.05 (s, 1H), 7.85 (s, 1H), 7.29 (s, 1H), 4.16 (q, J = 7.2 Hz, 2H), 3.31 (t, J = 6.8 Hz, 2H), 2.78 (t, J = 6.7 Hz, 2H), 1.27 (t, J = 7.1 Hz, 3H).

^{13}C NMR (176 MHz, $CDCl_3$) δ 192.21, 172.74, 155.76, 143.59, 142.21, 133.94, 130.00, 128.14, 111.59, 104.37, 60.94, 56.70, 33.82, 28.46, 14.34.

Compound 05

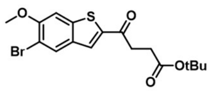

Rieke zinc (50 mg/mL THF slurry, approximately 20 mL, 15 mmol) was cannulated under nitrogen overpressure into a dry 50 mL two-neck flask equipped with a reflux condenser. tert-butyl 3-bromopropanoate (1.5 mL, 1.9 g, 9 mmol) was injected in one-shot at room temperature, and the slurry was refluxed for 1.5 hours. The suspension was then allowed to cool to room temperature and settle for 1 hour. The resulting organozinc bromide solution was cannula-filtered under nitrogen overpressure. The resulting dark-brown colloidal-like solution was left to stand at room temperature overnight to further settle the finer particles. Compound 03 (1.00 g, 3.48 mmol) was suspended in anhydrous THF (15 mL) under nitrogen and cooled in an ice bath. Oxalyl chloride (1.5 mL, 2.2 g, 17 mmol) and two drops of DMF were added sequentially, and the reaction was stirred for 14 hours while gradually warming up to room temperature. The volatiles were removed in vacuo, and the resulting pale-yellow solid was redissolved in anhydrous THF (15 mL) without further purification. Lithium chloride (76 mg, 1.8 mmol) dried with heating under vacuum and copper (I) iodide (133 mg, 0.7 mmol) were dissolved in anhydrous THF (4 mL) under nitrogen. The solution was cooled in an ice bath, to which was transferred the pre-cooled organozinc bromide solution via a syringe, taking care not to disturb the settled precipitates. The resulting mixture was stirred at 0 °C for 5 mins, and the acyl chloride solution was quickly added. The reaction was allowed to proceed at °C for 2.5 hours and quenched with saturated ammonium chloride (10 mL). The quenched mixture was diluted in ethyl acetate (150 mL) and washed with saturated ammonium chloride (100 mL × 2). The combined aqueous layer was further extracted with ethyl acetate (30 mL). The organic layers were combined, dried over magnesium sulfate, filtered, and concentrated in vacuo. Purification by flash column chromatography (dry-loading, 0 to 35% ethyl acetate in hexanes) afforded the desired product as a pale-yellow oil, which foamed and solidified under vacuum. 0.91 g, 65% yield. If desired, the product could be further recrystallized in ethyl acetate/hexanes mixtures.

HRMS (ESI/QTOF) m/z: $[M\text{-}tBu+H]^+$ Calculated for $C_{13}H_{12}BrO_4S$ 344.9619; Found 344.9627; $[M\text{-}OtBu]^+$ Calculated for $C_{13}H_{10}BrO_3S$ 326.9514; Found 326.9518. $[M+H]^+$ Calculated for $C_{17}H_{20}BrO_4S$ 401.0245; Found 401.0241. $[2M+Na]^+$ Calculated for $C_{34}H_{38}Br_2O_8S_2Na$ 823.0232; Found 823.0247.

^{1}H NMR (500 MHz, $CDCl_3$) δ 8.05 (s, 1H), 7.84 (s, 1H), 7.29 (s, 1H), 3.98 (s, 3H), 3.25 (t, J = 6.7 Hz, 2H), 2.71 (t, J = 6.8 Hz, 2H), 1.45 (s, 9H).

^{13}C NMR (126 MHz, $CDCl_3$) δ 192.41, 171.95, 155.70, 143.54, 142.38, 133.95, 129.96, 128.05, 111.53, 104.37, 80.98, 56.69, 33.91, 29.62, 28.22.

Compound 06

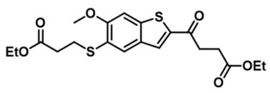

The reaction condition was based on a previous publication.[2] Compound 04 (0.77 g, 2.1 mmol), Xantphos (119 mg, 0.21 mmol), $Pd_2(dba)_3$ (95 mg, 0.1 mmol), ethyl 3-mercaptopropanoate (0.4 mL, 0.4 g, 3 mmol), and DIPEA (0.72 mL, 0.53 g, 4.1 mmol) were dissolved in 1,4-dioxane (10.5 mL) in a 50 mL flask equipped with a reflux condenser. The solution was degassed (3 freeze-pump-thaw cycles) and refluxed at 115 °C under nitrogen for 16 hours. Upon completion, the crude mixture was diluted in DCM and filtered through a Celite pad. The filter cake was further washed with DCM, and

the combined filtrate was concentrated in vacuo to afford an orange crude residue. Purification by flash column chromatography (wet-loading in DCM, 0 to 10% ethyl acetate in DCM/hexanes = 1/1) afforded the desired product as a white solid. 0.83 g, 94% yield.

HRMS (ESI/QTOF) m/z: $[M\text{-}OEt]^+$ Calculated for $C_{18}H_{19}O_5S_2$ 379.0674; Found 379.0744. $[M+H]^+$ Calculated for $C_{20}H_{25}O_6S_2$ 425.1093; Found 425.1170.

^{1}H NMR (500 MHz, $CDCl_3$) δ 7.87 (s, 1H), 7.78 (s, 1H), 7.25 (s, 1H), 4.16 (q, *J* = 7.1 Hz, 2H), 4.13 (q, *J* = 7.2 Hz, 2H), 3.97 (s, 3H), 3.31 (t, *J* = 6.8 Hz, 2H), 3.18 (t, *J* = 7.4 Hz, 2H), 2.77 (t, *J* = 6.8 Hz, 2H), 2.63 (t, *J* = 7.4 Hz, 2H), 1.25 (q, *J* = 7.1 Hz, 3H + 3H).

^{13}C NMR (126 MHz, $CDCl_3$) δ 192.21, 172.74, 171.85, 158.04, 143.34, 141.46, 133.35, 128.66, 127.44, 123.75, 103.33, 60.90, 60.87, 56.36, 34.25, 33.75, 28.49, 27.61, 14.31.

Compound 07

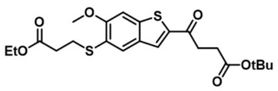

The reaction condition was based on a previous publication.[2] Compound 05 (0.39 g, 0.99 mmol), ethyl 3-mercaptopropanoate (0.19 mL, 0.20 g, 1.48 mmol), $Pd_2(dba)_3$ (45 mg, 0.05 mmol), Xantphos (57 mg, 99 mmol), and DIPEA (0.34 mL, 0.26 g, 1.97 mmol) in a 50 mL flask was dissolved in 1,4-dioxane (5 mL). A reflux condenser was attached, and the system was degassed (3 freeze-pump-thaw cycles) and refilled with argon. The reaction mixture was refluxed for 15 hours. Upon completion, the mixture was diluted in ethyl acetate (50 mL) and filtered over a pad of Celite. The orange filtrate was concentrated in vacuo to afford an oily residue, which was further purified by flash column chromatography (wet-loading, 0 to 10% ethyl acetate in DCM/hex = 1/1) to afford the desired product as a pale-orange gum, which solidified upon standing under vacuum. Quantitative yield.

HRMS (ESI/QTOF) m/z: $[M\text{-}tBu+H]^+$ Calculated for $C_{18}H_{21}O_6S_2$ 397.0780; Found 397.0786. $[M\text{-}OtBu]^+$ Calculated for $C_{18}H_{19}O_5S_2$ 379.0674; Found 379.0665.

^{1}H NMR (500 MHz, $CDCl_3$) δ 7.87 (s, 1H), 7.79 (s, 1H), 7.26 (s, 1H), 4.13 (q, *J* = 7.1 Hz, 2H), 3.98 (s, 3H), 3.25 (t, *J* = 6.8 Hz, 2H), 3.18 (t, *J* = 7.5 Hz, 2H), 2.70 (t, *J* = 6.8 Hz, 2H), 2.63 (t, *J* = 7.5 Hz, 2H), 1.44 (s, 9H), 1.24 (s, 3H).

^{13}C NMR (126 MHz, $CDCl_3$) δ 192.44, 171.98, 171.88, 158.04, 143.35, 141.67, 133.40, 128.58, 127.53, 123.68, 103.36, 80.92, 60.92, 56.38, 34.29, 33.89, 29.69, 28.21, 27.66, 14.33.

Compound 08

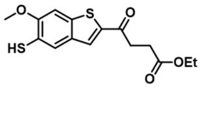

Compound 06 (0.83 g, 2.0 mmol) and ethanethiol (0.23 mL, 0.19 g, 3.1 mmol) were dissolved in anhydrous THF (10 mL) under nitrogen and cooled to approximately -20 °C in a MeOH/water/dry ice bath (MeOH/water = 20/80, v/v). To the stirred solution was added dropwise sodium ethanolate (21% in ethanol, 0.40 g, 2.2 mL, 5.9 mmol). The reaction was allowed to proceed for 2 hours, during which time the temperature was kept below -10 °C. Upon completion, the reaction

was quenched by saturated ammonium chloride at -10 °C, diluted in DCM (75 mL), and washed with saturated ammonium chloride (75 mL × 2). The combined aqueous layer was further extracted with DCM (25 mL). The organic layers were combined, dried over magnesium sulfate, filtered, and concentrated in vacuo to afford a yellow solid, which was used in the next step without further purifications.

HRMS (ESI/TOF) m/z: $[M+H]^+$ Calculated for $C_{15}H_{17}O_4S_2$ 325.0568; Found: 325.0557. $[M+Na]^+$ Calculated for $C_{15}H_{16}O_4S_2Na$ 347.0388; Found 347.0378.

^1H NMR (500 MHz, $CDCl_3$) δ 7.81 (s, 1H), 7.75 (s, 1H), 7.24 (s, 1H), 4.16 (q, *J* = 7.2 Hz, 2H), 3.98 (s, 3H), 3.91 (s, 1H), 3.31 (t, *J* = 6.8 Hz, 2H), 2.77 (t, *J* = 6.8 Hz, 2H), 1.26 (t, *J* = 7.1 Hz, 3H).

^{13}C NMR (126 MHz, $CDCl_3$) δ 192.24, 172.79, 155.43, 142.00, 141.53, 133.32, 128.27, 125.60, 120.91, 103.34, 60.90, 56.44, 33.79, 28.49, 14.33.

Compound 09

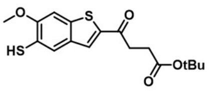

Compound 07 (100 mg, 0.22 mmol) and ethanethiol (25 μL, 22 mg, 0.35 mmol) were dissolved in anhydrous THF (10 mL) under nitrogen. The solution was cooled to -20 °C in a cooling bath (MeOH/water = 20/80 with dry ice) and stirred for 10 minutes. Sodium ethoxide (0.25 mL, 46 mg, 21% in ethanol, 0.67 mmol) was slowly added dropwise, and the reaction was allowed to proceed at -20 °C to -15 °C for 2 hours. The reaction mixture was then quenched with saturated ammonium chloride (10 mL) at -20 °C, diluted in ethyl acetate (50 mL), and washed with saturated ammonium chloride (50 mL × 2). The combined aqueous layer was extracted again with ethyl acetate (20 mL). The organic layers were combined, dried over magnesium sulfate, filtered, and concentrated in vacuo. Purification by flash column chromatography (wet-loading in DCM/hex =1 /1; 0 to 30% ethyl acetate in hexanes) afforded the desired product as a colorless gum, which solidified upon standing at -20 °C. 63 mg, 81% yield.

HRMS (ESI/QTOF) m/z: $[M\text{-}tBu+H]^+$ Calculated for $C_{13}H_{13}O_4S_2$ 297.0256; Found 297.0286. $[M\text{-}OtBu]^+$ Calculated for $C_{13}H_{11}O_3S_2$ 279.0150; Found 279.0177. $[M+H]^+$ Calculated for $C_{17}H_{21}O_4S_2$ 353.0882; Found 353.0881.

^1H NMR (500 MHz, $CDCl_3$) δ 7.74 (s, 1H), 7.67 (s, 1H), 7.18 (s, 1H), 3.93 (s, 3H), 3.88 (s, 1H), 3.22 (t, *J* = 6.8 Hz, 2H), 2.68 (t, *J* = 6.8 Hz, 2H), 1.43 (s, 9H).

^{13}C NMR (126 MHz, $CDCl_3$) δ 192.37, 171.91, 155.23, 141.78, 141.53, 133.20, 128.14, 125.41, 120.70, 103.19, 80.79, 56.33, 33.77, 29.57, 28.13.

Compound 10

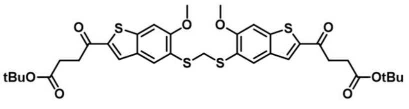

Compound 09 (64 mg, 0.18 mmol) and DIPEA (95 μL, 0.54 mmol) were dissolved in anhydrous toluene (2 mL) under argon. Bromochloromethane (0.24 mL, 0.47 g, 3.6 mmol) was added, and the solution was stirred for another 3 hours

at room temperature. As the reaction did not proceed, DBU (40 μL, 41 mg, 0.27 mmol) was added rapidly. The reaction was allowed to proceed for 10 minutes, after which time complete consumption of the starting material was confirmed by TLC analysis. The volatiles were removed in vacuo, and additional Compound 09 (68 mg, 0.19 mmol) was added. The mixture was redissolved in anhydrous DMF (2 mL) and stirred for 19 hours at room temperature. At this point more DIPEA (100 μL, 74 mg, 0.57 mmol) was added, and the reaction mixture was stirred for another 20 hours. The resulting suspension with white precipitates were redissolved in DCM (75 mL) and washed with aqueous lithium chloride (75 mL × 2). The organic layer was dried over magnesium sulfate, filtered, and concentrated in vacuo. Purification by flash column chromatography (wet-loading in DCM; 0 to 10% ethyl acetate in DCM/hexanes = 1/1) afforded the desired product as a white crystalline solid. 100 mg, 77% yield.

HRMS (ESI/QTOF) m/z: $[M+H]^+$ Calculated for $C_{35}H_{41}O_8S_4$ 717.1685; Found 717.1763. $[M\text{-}2tBu\text{-}H_2O+H]^+$ Calculated for $C_{27}H_{23}O_7S_4$ 587.0327; Found 587.0362.

^1H NMR (700 MHz, $CDCl_3$) δ 7.82 (s, 2H), 7.81 (s, 2H), 7.16 (s, 2H), 4.42 (s, 2H), 3.91 (s, 6H), 3.25 (t, *J* = 6.7 Hz, 4H), 2.71 (t, *J* = 6.7 Hz, 4H), 1.45 (s, 18H).

^{13}C NMR (176 MHz, $CDCl_3$) δ 192.45, 172.02, 158.19, 144.09, 141.78, 133.26, 129.61, 128.56, 122.52, 103.31, 80.93, 56.32, 37.07, 33.89, 29.64, 28.24.

Compound 11

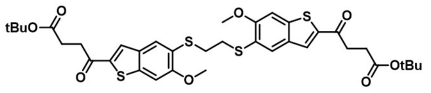

Compound 09 (80 mg, 0.23 mmol) was dissolved in anhydrous DMF (0.39 mL) under argon. 1,2-Dibromoethane (21 mg, 0.11 mmol, 0.21 mL of 100 mg/mL solution in DMF) and DIPEA (80 μL, 59 mg, 0.45 mmol) were added sequentially. The reaction mixture was stirred for 17 hours at room temperature. The resulting colorless solution was diluted in DCM (75 mL) and washed with aqueous lithium chloride (0.5 M, 75 mL × 2). The organic layer was dried over magnesium sulfate, filtered, and concentrated in vacuo. Purification by flash column chromatography (wet-loading in DCM/hexanes mixtures; 0 to 10% ethyl acetate in DCM/hexanes = 1/1) afforded the desired product as a white solid. 78 mg, 93% yield.

HRMS (ESI/QTOF) m/z: $[M+H]^+$ Calculated for $C_{36}H_{43}O_8S_4$ 731.1841; Found 731.1906. $[M\text{-}2tBu+H]^+$ Calculated for $C_{28}H_{27}O_8S_4$ 619.0589; Found 619.0605. $[M\text{-}2tBu\text{-}H_2O+H]^+$ Calculated for $C_{28}H_{25}O_7S_4$ 601.0484; Found 601.0550.

^1H NMR (700 MHz, $CDCl_3$) δ 7.78 (s, 2H), 7.70 (s, 2H), 7.24 (s, 2H), 3.87 (s, 6H), 3.24 (t, *J* = 6.7 Hz, 4H), 3.11 (s, 4H), 2.71 (t, *J* = 6.7 Hz, 4H), 1.45 (s, 18H).

^{13}C NMR (176 MHz, $CDCl_3$) δ 192.49, 172.02, 158.13, 143.51, 141.82, 133.34, 128.50, 128.00, 123.17, 103.46, 80.95, 56.33, 33.88, 31.92, 29.67, 28.23.

Compound 12

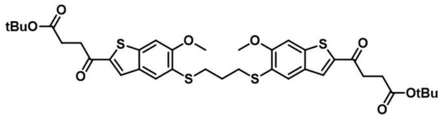

Compound 09 (80 mg, 0.23 mmol) was dissolved in anhydrous DMF (0.37 mL) under argon. 1,3-dibromopropane (23 mg, 0.11 mmol, 0.23 mL as 100 mg/mL DMF solution) and anhydrous DIPEA (80 μL, 59 mg, 0.45 mmol) were added sequentially. After being stirred for several minutes a large amount of white precipitate formed. The suspension was then stirred for another 48 hours under argon. The reaction mixture was then diluted in DCM (75 mL) and washed with aqueous lithium chloride (0.5 M, 75 mL × 2). The organic layer was dried over magnesium sulfate, filtered, and concentrated in vacuo. Purification by flash column chromatography (wet-loading in DCM, 0 to 10% ethyl acetate in DCM/hexanes = 1/1) afforded the desired product as a white solid. 53 mg, 62% yield.

HRMS (ESI/QTOF) m/z: $[M+H]^+$ Calculated for $C_{37}H_{45}O_8S_4$ 745.1998; Found 745.2065. $[M-2tBu+H]^+$ Calculated for $C_{29}H_{27}O_7S_4$ 615.0640; Found 615.0668.

^{1}H NMR (700 MHz, $CDCl_3$) δ 7.79 (s, 2H), 7.70 (s, 2H), 7.24 (s, 2H), 3.97 (s, 6H), 3.24 (t, *J* = 6.8 Hz, 4H), 3.11 (t, *J* = 7.0 Hz, 4H), 2.70 (t, *J* = 6.8 Hz, 4H), 2.03 (p, *J* = 7.0 Hz, 2H), 1.45 (s, 18H).

^{13}C NMR (176 MHz, $CDCl_3$) δ 192.42, 172.04, 157.72, 142.74, 141.58, 133.44, 128.47, 126.14, 124.69, 103.24, 80.92, 56.39, 33.91, 31.25, 29.68, 28.23, 27.90.

Compound 13

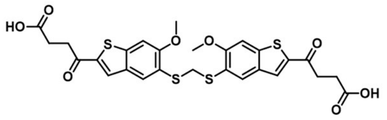

Compound 10 (95 mg, 0.13 mmol) was dissolved in a mixture of TFA and DCM (10% TFA, v/v, 5 mL). The solution was stirred for 3 hours at room temperature. The volatiles were then removed azeotropically with toluene. The solid residue was resuspended in ethanol (10 mL) and refluxed briefly for 5 minutes. The suspension was allowed to cool to room temperature. The precipitates were collected by vacuum filtration, washed with ice-cold ethanol (2 mL × 2) and room temperature DCM (2 mL × 2), and dried in vacuo to afford the desired product as a white powder. 45 mg, 56% yield.

HRMS (ESI/QTOF) m/z: $[M+H]^+$ Calculated for $C_{27}H_{25}O_8S_4$ 605.0433; Found 605.0433. $[M-H_2O+H]^+$ Calculated for $C_{27}H_{23}O_7S_4$ 587.0327; Found 587.0362.

^{1}H NMR (700 MHz, DMSO) δ 12.18 (s, 2H), 8.21 (s, 2H), 7.97 (s, 2H), 7.61 (s, 2H), 4.64 (s, 2H), 3.88 (s, 6H), 3.26 (t, *J* = 6.5 Hz, 4H), 2.60 (t, *J* = 6.4 Hz, 4H).

^{13}C NMR (176 MHz, DMSO) δ 192.77, 173.64, 156.89, 141.96, 140.83, 133.10, 129.94, 126.52, 122.82, 103.89, 56.36, 33.67, 33.15, 27.90.

Compound 14

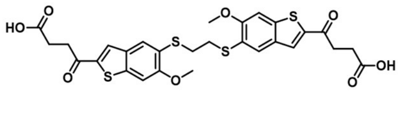

Compound 11 (73 mg, 0.10 mmol) was dissolved in a mixture of trifluoroacetic acid and DCM (10% TFA, v/v). The resulting orange solution was stirred at room temperature for 6 hours, during which time it gradually decolorized and a fine precipitate formed. The precipitates were collected by vacuum filtration and washed extensively with DCM. The

pale-yellow powder was resuspended in a mixture of water and acetonitrile and lyophilized to afford the desired product as a white powder. 37 mg, 60% yield.

HRMS (ESI/TOF) m/z: $[M+H]^+$ Calculated for $C_{28}H_{27}O_8S_4$ 619.0589; Found 619.0634. $[M+Na]^+$ Calculated for $C_{28}H_{26}O_8S_4Na$ 641.0409; Found 641.0472. $[M-OH]^+$ Calculated for $C_{28}H_{25}O_7S_4$ 601.0484; Found 601.0530. $[2M+Na]^+$ Calculated for 1259.0920; Found 1259.1046.

^{1}H NMR (700 MHz, DMSO) δ 12.19 (s, 2H), 8.16 (s, 2H), 7.85 (s, 2H), 7.65 (s, 2H), 3.86 (s, 6H), 3.25 (t, *J* = 6.1 Hz, 4H), 3.20 (s, 4H), 2.60 (t, *J* = 6.7 Hz, 4H).

^{13}C NMR (126 MHz, DMSO) δ 192.75, 173.68, 156.93, 141.53, 140.78, 133.23, 129.92, 125.38, 123.66, 104.00, 56.32, 33.17, 30.28, 27.93.

Compound 15

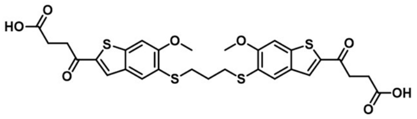

Compound 12 (53 mg, 0.071 mmol) was dissolved in a mixture of trifluoroacetic acid and DCM (10% TFA, v/v). Immediately upon dissolution the reaction mixture turned dark yellow. The reaction was allowed to proceed at room temperature for 6 hours, during which time it gradually decolorized and precipitates formed. The precipitates were then collected by vacuum filtration, washed extensively with DCM, resuspended in a mixture of water and acetonitrile, and lyophilized to afford the desired product as an off-white powder. 29 mg, 64% yield.

HRMS (ESI/TOF) m/z: $[M+Na]^+$ Calculated for $C_{29}H_{28}O_8S_4Na$ 655.0565; Found 655.0572.

^{1}H NMR (700 MHz, DMSO) δ 12.20 (s, 2H), 8.15 (s, 2H), 7.84 (s, 2H), 7.64 (s, 2H), 3.91 (s, 6H), 3.24 (t, *J* = 6.4 Hz, 4H), 3.12 (t, *J* = 7.1 Hz, 4H), 2.60 (t, *J* = 6.4 Hz, 4H), 1.95 (p, *J* = 7.0 Hz, 2H).

^{13}C NMR (176 MHz, DMSO) δ 192.65, 173.64, 156.64, 140.89, 140.65, 133.26, 129.78, 124.73, 123.98, 103.77, 56.33, 33.15, 29.44, 27.88, 27.16.

Compound 16

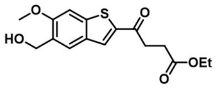

Compound 04 (0.4 g, 1.08 mmol), Xphos Pd G2 (40 mg, 0.05 mmol), and (tributylstannyl)methanol (0.59 mL, 0.20 g, 2.11 mmol) were suspended in anhydrous 1,4-dioxane (10 mL). The suspension was degassed (3 freeze-pump-thaw cycles) and stirred at 90 °C for 3 hours under an argon atmosphere. Upon completion, the reaction mixture was diluted in ethyl acetate (50 mL) and washed with aqueous potassium fluoride (1 M, 50 mL × 2). The combined aqueous layer was further extracted with ethyl acetate (30 mL). The cloudy organic layers were combined, dried over magnesium sulfate, and filtered through a Celite pad. The filter pad was rinsed with ethyl acetate (20 mL × 2), resulting in a clear yellow solution. The combined filtrate was concentrated in vacuo and purified by flash column chromatography (wet-loading in DCM, 0 to 90% ethyl acetate in hexanes) to afford the desired product as a white solid. 0.31 g, 89% yield.

HRMS (ESI/QTOF) m/z: $[M+H]^+$ Calculated for $C_{16}H_{19}O_5S$ 323.0954; Found 323.1007. $[M-OEt]^+$ Calculated for $C_{14}H_{13}O_4S$ 277.0535; Found 277.0572.

^{1}H NMR (500 MHz, $CDCl_3$) δ 7.90 (s, 1H), 7.77 (s, 1H), 7.27 (s, 1H), 4.76 (s, 2H), 4.16 (q, *J* = 7.1 Hz, 2H), 3.94 (s, 3H), 3.31 (t, *J* = 6.8 Hz, 2H), 2.77 (t, *J* = 6.8 Hz, 2H), 1.26 (t, *J* = 7.1 Hz, 3H).

^{13}C NMR (126 MHz, $CDCl_3$) δ 192.28, 172.84, 158.02, 144.11, 141.12, 132.92, 129.39, 129.17, 125.30, 103.05, 62.00, 60.89, 55.88, 33.75, 28.51, 14.32.

Compound 17

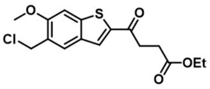

Compound 16 (0.21 g, 0.64 mmol) and anhydrous DIPEA (0.22 mL, 0.16 g, 1.27 mmol) were dissolved in anhydrous DCM (5 mL) under nitrogen. The solution was cooled in an ice bath, and methanesulfonyl chloride (59 μL, 87 mg, 0.76 mmol) was added dropwise over 3 minutes. The reaction was allowed to proceed for another 18 hours while gradually warming up to room temperature. The reaction mixture was concentrated in vacuo, redissolved in a mixture of DCM and hexanes, and purified by flash column chromatography (wet-loading, 0 to 50% ethyl acetate in hexanes) to afford the chlorinated product as a white crystalline solid. 181 mg, 83% yield.

HRMS (ESI/QTOF) m/z: $[M+H]^+$ Calculated for $C_{16}H_{18}ClO_4S$ 341.0614; Found 341.0656. $[M-OEt]^+$ Calculated for $C_{14}H_{12}ClO_3S$ 295.0196; Found 295.0234.

^{1}H NMR (700 MHz, $CDCl_3$) δ 7.91 (s, 1H), 7.85 (s, 1H), 7.29 (s, 1H), 4.73 (s, 2H), 4.16 (q, *J* = 7.2 Hz, 2H), 3.97 (s, 3H), 3.32 (t, *J* = 6.8 Hz, 2H), 2.78 (t, *J* = 6.7 Hz, 2H), 1.26 (t, *J* = 7.1 Hz, 3H).

^{13}C NMR (176 MHz, $CDCl_3$) δ 192.24, 172.78, 157.69, 145.10, 141.47, 132.78, 129.20, 127.44, 125.91, 103.54, 60.90, 56.11, 41.91, 33.78, 28.50, 14.33.

Compound 18

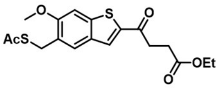

Compound 16 (83 mg, 0.26 mmol) and triethylamine (72 μL, 52 mg, 0.51 mmol) were dissolved in anhydrous DCM (5 mL) under nitrogen and cooled in an ice bath. To the solution was added methanesulfonyl chloride (24 μL, 35 mg, 0.31 mmol), and the reaction was allowed to proceed for 20 hours while gradually warming up to room temperature. The reaction mixture was diluted in DCM (50 mL) and washed with saturated ammonium chloride (50 mL × 2). The combined aqueous layer was further extracted with DCM (30 mL). The organic layers were combined, dried over magnesium sulfate, filtered, and concentrated in vacuo to afford a white solid residue. To the residue was added potassium thioacetate (59 mg, 0.51 mmol), and the mixture was suspended in anhydrous DMF (2 mL) under nitrogen. The suspension was warmed up to 45 °C and stirred for 17 hours. Upon completion, the reaction mixture was diluted in DCM (50 mL) and washed with aqueous lithium chloride (0.5 M, 75 mL × 2). The combined aqueous layer was further extracted with DCM (25 mL). The organic layers were combined, dried over magnesium sulfate, filtered, and

concentrated in vacuo to afford an oily residue. Purification by flash column chromatography (wet-loading in a mixture of DCM and hexanes; 0 to 50% ethyl acetate in hexanes) afforded the desired product as an off-white solid. 65 mg, 66% yield.

HRMS (ESI/QTOF) m/z: $[M+H]^+$ Calculated for $C_{18}H_{21}O_5S_2$ 381.0831; Found 381.0842. $[M-OEt]^+$ Calculated for $C_{16}H_{15}O_4S_2$ 335.0412; Found 335.0482.

^1H NMR (700 MHz, $CDCl_3$) δ 7.89 (s, 1H), 7.85 (s, 1H), 7.24 (s, 1H), 4.20 (s, 2H), 4.16 (q, *J* = 7.2 Hz, 2H), 3.94 (s, 3H), 3.31 (t, *J* = 6.7 Hz, 2H), 2.77 (t, *J* = 6.7 Hz, 2H), 2.32 (s, 3H), 1.26 (t, *J* = 7.2 Hz, 3H).

^{13}C NMR (176 MHz, $CDCl_3$) δ 195.72, 192.28, 172.78, 157.88, 144.08, 141.14, 132.81, 129.34, 127.28, 126.19, 103.19, 60.88, 56.00, 33.73, 30.56, 28.84, 28.54, 14.33.

Compound 19

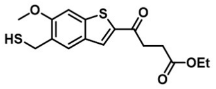

Compound 18 (60 mg, 0.16 mmol) was dissolved in anhydrous THF (4 mL) under argon and cooled to 0 °C. To the stirred solution was added sodium ethanolate (21% in ethanol, 59 µL, 11 mg, 0.16 mmol). The reaction was allowed to proceed for 1.5 hours at 0 °C, resulting in a cloudy suspension. The volatiles were removed in vacuo, and the deacetylated crude residue was used in the next step without further purifications.

Compound 20

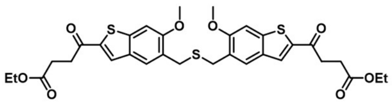

Compound 17 (60 mg, 0.18 mmol) and sodium iodide (24 mg, 0.16 mmol) were added to the previously obtained crude Compound 19 (approximately 0.16 mmol). The starting materials were dissolved in anhydrous DMF (3 mL) under argon at room temperature, and the reaction mixture was stirred for another 16 hours. The crude reaction was diluted in DCM (50 mL) and washed with hydrochloric acid (0.5 M, 50 mL × 2). The combined aqueous layer was further extracted with DCM (25 mL). The organic layers were combined, dried over magnesium sulfate, filtered, and concentrated in vacuo. Purification by flash column chromatography (wet-loading in DCM, 0 to 15% ethyl acetate in hexanes) afforded the desired product as a white solid. 61 mg, 61% yield

HRMS (ESI/QTOF) m/z: $[M+H]^+$ Calculated for $C_{32}H_{35}O_8S_3$ 643.1494; Found 643.1492. $[M-OEt]^+$ Calculated for $C_{30}H_{29}O_7S_3$ 597.1076; Found 597.1099.

^1H NMR (700 MHz, $CDCl_3$) δ 7.83 (s, 2H), 7.69 (s, 2H), 7.24 (s, 2H), 4.17 (q, *J* = 7.2 Hz, 4H), 3.90 (s, 6H), 3.81 (s, 4H), 3.31 (t, *J* = 6.8 Hz, 4H), 2.78 (t, *J* = 6.8 Hz, 4H), 1.27 (t, *J* = 7.2 Hz, 6H).

^{13}C NMR (176 MHz, $CDCl_3$) δ 192.25, 172.86, 158.08, 143.65, 141.02, 132.76, 129.16, 126.90, 126.79, 103.23, 60.89, 55.97, 33.77, 31.11, 28.53, 14.34.

Compound 21

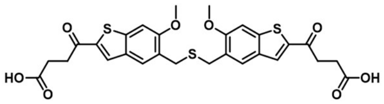

Compound 20 (58 mg, 0.09 mmol) was suspended in a mixture of THF (5 mL) and water (5 mL). Aqueous sodium hydroxide (1 M, 0.2 mL, 0.2 mmol) was added, and the suspension was stirred at room temperature for 6 hours. As the reaction proceeded slowly, aqueous lithium hydroxide (1 M, 0.2 mL, 0.2 mmol) was added, and the reaction mixture was stirred overnight, resulting in a clear solution. The solution was then acidified to pH 3 with citric acid (1 M in water), and most residual THF was removed in vacuo, resulting in a cloudy suspension. The precipitates were collected by vacuum filtration, washed with water (3 mL × 2), and dried in vacuo to afford the desired product as an off-white powder. 27 mg, 51% yield.

HRMS (ESI/QTOF) m/z: $[M+H]^+$ Calculated for $C_{28}H_{27}O_8S_3$ 587.0868; Found 587.0858. $[M-H_2O+H]^+$ Calculated for $C_{28}H_{25}O_7S_3$ 569.0763; Found 569.0766.

^{1}H NMR (700 MHz, DMSO) δ 12.19 (s, 2H), 8.25 (s, 2H), 7.86 (s, 2H), 7.62 (s, 2H), 3.88 (s, 6H), 3.77 (s, 4H), 3.27 (t, *J* = 6.5 Hz, 4H), 2.60 (t, *J* = 6.4 Hz, 4H).

^{13}C NMR (176 MHz, DMSO) δ 192.72, 173.66, 157.55, 142.52, 140.41, 132.45, 130.46, 126.73, 126.49, 103.93, 56.00, 33.14, 30.17, 27.91.

Compound 22

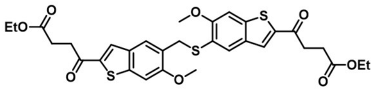

Compound 17 (60 mg, 0.18 mmol), Compound 08 (63 mg, 0.19 mmol), and DIPEA (46 µL, 34 mg, 0.26 mmol) were dissolved in anhydrous DMF (2 mL) under nitrogen. The reaction was allowed to proceed at room temperature for 40 hours, diluted in DCM (50 mL), and washed with aqueous lithium chloride (0.5 M, 50 mL × 2). The combined aqueous layer was extracted with DCM (25 mL). The organic layers were combined, drided over magnesium sulfate, filtered, and concentrated in vacuo to afford an orange solid residue. Purification by flash column chromatography (wet-loading in DCM, 0 to 20% ethyl acetate in DCM/hexanes = 1/1) afforded the desired product as an off-white solid. 79 mg, 72% yield.

HRMS (ESI/QTOF) m/z: $[M+H]^+$ Calculated for $C_{31}H_{33}O_8S_3$ 629.1338; Found 629.1373. $[M-OEt]^+$ Calculated for $C_{29}H_{27}O_7S_3$ 583.0919; Found 583.0880.

^{1}H NMR (700 MHz, $CDCl_3$) δ 7.79 (s, 1H), 7.78 (s, 1H), 7.64 (s, 1H), 7.63 (s, 1H), 7.26 (s, 1H), 7.25 (s, 1H), 4.23 (s, 2H), 4.15 (q, *J* = 7.1 Hz, 4H), 3.96 (s, 3H), 3.92 (s, 3H), 3.28 (t, *J* = 6.7 Hz, 4H), 2.76 (t, *J* = 6.6 Hz, 2H), 2.75 (t, *J* = 6.7 Hz, 2H), 1.25 (t, *J* = 7.1 Hz, 6H).

^{13}C NMR (176 MHz, $CDCl_3$) δ 192.26, 192.23, 172.81, 172.80, 158.01, 158.00, 143.93, 143.16, 141.32, 141.07, 133.37, 132.82, 129.24, 128.76, 127.28, 126.82, 125.63, 124.95, 103.20, 103.09, 60.90, 60.88, 56.39, 56.03, 33.75, 33.72, 32.25, 28.49, 28.48, 14.33.

Compound 23

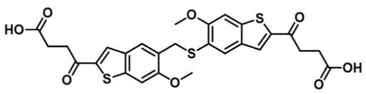

Compound 22 (70 mg, 0.11 mmol) was suspended in a mixture of THF (10 mL) and water (10 mL). Aqueous sodium hydroxide (1 M, 0.25 mL, 0.25 mmol) was added, and the reaction was stirred at room temperature for 6 hours. Additional aqueous lithium hydroxide (1 M, 0.25 mL, 0.25 mmol) was added, and the reaction was allowed to proceed at 40 °C for a prolonged period until it eventually clarified to a clear solution. The reaction mixture was then acidified with citric acid (1 M aqueous) to pH 3, and the residual THF was removed in vacuo. The resulting precipitates were collected by vacuum filtration, washed sequentially with water (3 mL × 5) and DCM (3 mL × 5), and dried in vacuo to afford the desired product as an off-white solid. 52 mg, 81% yield.

HRMS (ESI/QTOF) m/z: $[M+H]^+$ Calculated for $C_{27}H_{25}O_8S_3$ 573.0712; Found 573.0751. $[M-H_2O+H]^+$ $C_{27}H_{23}O_7S_3$ Calculated for $C_{27}H_{23}O_7S_3$ 555.0606; Found 555.0621.

^{1}H NMR (700 MHz, DMSO) δ 12.18 (s, 2H), 8.25 (s, 1H), 8.19 (s, 1H), 7.90 (s, 1H), 7.84 (s, 1H), 7.66 (s, 2H), 4.27 (s, 2H), 3.92 (s, 3H), 3.91 (s, 3H), 3.25 (t, J = 6.0 Hz, 2H), 3.24 (s, J = 6.5 Hz, 2H), 2.59 (t, J = 6.1 Hz, 2H), 2.58 (t, J = 6.2 Hz, 2H).

^{13}C NMR (176 MHz, DMSO) δ 192.75, 192.70, 173.64, 157.59, 156.73, 142.83, 141.21, 140.64, 140.52, 133.19, 132.56, 130.53, 129.97, 126.90, 125.04, 124.93, 124.74, 104.01, 103.74, 56.35, 56.12, 33.14, 33.12, 30.36, 27.88.

Compound 24

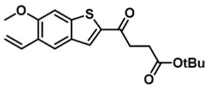

The reaction condition was taken from the literature.[3] Compound 04 (0.15 g, 0.38 mmol), 4,4,5,5-tetramethyl-2-vinyl-1,3,2-dioxaborolane (70 µL, 64 mg, 0.4 mmol), tripotassium phosphate (0.24 g, 1.1 mmol), Sphos (15 mg, 0.038 mmol), and palladium(II) acetate (7.9 mg, 0.035 mmol) were dissolved in 1,4-dioxane (3 mL) and water (0.1 mL). The solution was degassed (3 freeze-pump-thaw cycles) and heated under nitrogen for 2 hours. The reaction mixture was allowed to cool to room temperature, diluted in ethyl acetate (50 mL), and filtered through a Celite pad. The filter cake was further washed with ethyl acetate (20 mL), and the combined filtrate was concentrated in vacuo to afford an oily residue. Purification by flash column chromatography (wet-loading in DCM/hexanes; 0 to 20% ethyl acetate in hexanes) afforded 109 mg of an off-white gum, which solidified after standing under vacuum. Further purification by recrystallization (refluxing hexanes/diethyl ether) afforded the desired product as a white, needle-like crystalline solid. 55 mg, 42% yield.

HRMS (ESI/QTOF) m/z: $[M-OtBu]^+$ Calculated for $C_{15}H_{13}O_3S$ 273.0586; Found 273.0677. $[M-tBu+H]^+$ Calculated for $C_{15}H_{15}O_4S$ 291.0691; Found 291.0736.

^{1}H NMR (500 MHz, CD_2Cl_2) δ 7.96 (s, 1H), 7.94 (s, 1H), 7.30 (s, 1H), 7.08 (dd, J = 17.7, 11.2 Hz, 1H), 5.82 (dd, J = 17.7, 1.4 Hz, 1H), 5.33 (dd, J = 12.5, 1.4 Hz, 1H), 3.93 (s, 3H), 3.25 (t, J = 6.7 Hz, 2H), 2.66 (t, J = 6.7 Hz, 2H), 1.43 (s, 9H).

^{13}C NMR (176 MHz, CD_2Cl_2) δ 192.78, 172.09, 157.86, 144.13, 141.66, 133.43, 131.91, 129.70, 127.23, 123.59, 115.40,

103.56, 80.83, 56.21, 34.06, 29.91, 28.18.

Compound 25

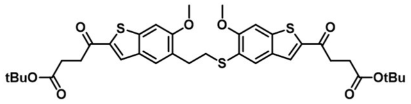

Compound 24 (50 mg, 0.14 mmol), Compound 09 (64 mg, 0.18 mmol), and AIBN (2.5 mg, 0.015 mmol) were suspended in methanol (6 mL). The suspension was degassed (3 freeze-pump-thaw cycles) and refluxed under argon for 20 hours. Methanol was removed in vacuo, leaving behind an off-white solid. Purification by flash column chromatography (wet-loading in DCM/hexanes mixtures, 0 to 10% ethyl acetate in DCM/hexanes = 1/1) afforded a white solid. As the product was inseparable from a small amount of oxidized disulfide impurity, the solid was dissolved in DCM (4 mL), ethanethiol (0.2 mL), and DIPEA (2 drops). The mixture was stirred at room temperature for 2 hours and purified directly by flash column chromatography (0 to 10% ethyl acetate in DCM/hexanes = 1/1) to afford the pure product as a white solid. 69 mg, 69% yield.

HRMS (ESI/QTOF) m/z: $[M+H]^+$ Calculated for $C_{36}H_{43}O_8S_3$ 699.2120; Found 699.2135. $[M\text{-}2tBu+H]^+$ Calculated for $C_{28}H_{27}O_8S_3$ 587.0868; Found 587.0858. $[M\text{-}2tBu\text{-}H_2O+H]^+$ Calculated for $C_{28}H_{25}O_7S_3$ 569.0763; Found 569.0766.

^1H NMR (700 MHz, $CDCl_3$) δ 7.87 (s, 1H), 7.83 (s, 1H), 7.74 (s, 1H), 7.62 (s, 1H), 7.23 (s, 1H), 7.21 (s, 1H), 3.97 (s, 3H), 3.91 (s, 3H), 3.26 (t, J = 6.8 Hz, 2H), 3.25 (t, J = 6.8 Hz, 2H), 3.24 – 3.20 (m, 2H), 3.09 – 3.06 (m, 2H), 2.71 (t, J = 6.8 Hz, 2H), 2.70 (t, J = 6.8 Hz, 2H), 1.45 (s, 18H).

^{13}C NMR (176 MHz, $CDCl_3$) δ 192.48, 192.43, 172.07, 172.06, 158.29, 157.60, 143.39, 142.45, 141.48, 141.09, 133.46, 132.94, 129.06, 128.45, 128.37, 126.96, 125.67, 125.35, 103.04, 103.03, 80.93, 80.89, 56.35, 55.82, 33.91, 33.89, 31.93, 31.07, 29.72, 29.68, 28.23.

Compound 26

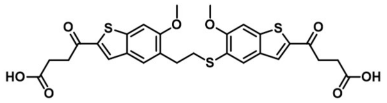

Compound 25 (63 mg, 0.09 mmol) was dissolved in a mixture of TFA and DCM (10% TFA, v/v, 5 mL). The solution was stirred at room temperature for 3 hours. The volatiles were then removed azeotropically with toluene. The residue was resuspended in ethanol (5 mL) and briefly refluxed for 5 minutes. The suspension was allowed to cool to room temperature. The precipitates were then collected by vacuum filtration, washed with ice-cold ethanol (2 mL × 2) and room temperature DCM (2 mL × 2), and dried in vacuo to afford the desired product as an off-white powder. 36 mg, 68% yield.

HRMS (ESI/QTOF) m/z: $[M+H]^+$ Calculated for $C_{28}H_{27}O_8S_3$ 587.0868; Found 587.0858. $[M\text{-}H_2O+H]^+$ Calculated for $C_{28}H_{25}O_7S_3$ 569.0763; Found 569.0766.

^1H NMR (700 MHz, DMSO) δ 12.20 (s, 2H), 8.25 (s, 1H), 8.20 (s, 1H), 7.89 (s, 1H), 7.83 (s, 1H), 7.63 (s, 1H), 7.60 (s,

1H), 3.91 (s, 3H), 3.90 (s, 3H), 3.274 (t, *J* = 6.0 Hz, 2H), 3.268 (t, *J* = 6.9 Hz, 2H), 3.22 (t, *J* = 7.6 Hz, 2H), 3.01 (t, *J* = 7.5 Hz, 2H), 2.61 (t, *J* = 6.4 Hz, 2H), 2.60 (t, *J* = 6.4 Hz, 2H).

^{13}C NMR (176 MHz, DMSO) δ 192.73, 192.70, 173.67, 173.65, 157.86, 156.57, 142.26, 140.79, 140.62, 140.29, 133.28, 132.62, 130.39, 129.85, 127.66, 126.97, 125.06, 123.94, 103.67, 56.30, 56.00, 33.15, 30.36, 29.78, 27.93, 27.91.

Compound 27

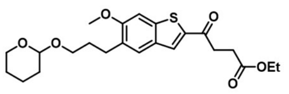

Rieke zinc (5 g/dL, 5 mL, 3.84 mmol) was transferred to a two-neck flask with a syringe under nitrogen. 2-(3-Bromopropoxy)tetrahydro-2*H*-pyran (0.5 mL, 2.95 mmol) was added, and the resulting suspension was refluxed for 2 hours. The suspension was allowed to cool to room temperature and to settle for 1 hour. The supernatant was transferred to a dry flask using a cannula equipped with a piece of filter paper. The resulting dark brown slurry (assumed to be approximately 0.5 M of organozinc reagent) was used in the next step without further purification. Compound 04 (100 mg, 0.27 mmol), Xphos Pd G4 (12 mg, 0.014 mmol), and lithium chloride (23 mg, 0.54 mmol) were dissolved in anhydrous THF (3 mL) under nitrogen. To the stirred solution was quickly added the previously prepared 3-(2-tetrahydro-2*H*-pyranoxy)propylzinc bromide (0.5 M, 0.81 mL, 0.41 mmol). The resulting dark solution was stirred at 50 °C for 1 hour. The reaction mixture was then allowed to cool to room temperature, diluted in DCM (50 mL), and filtered through a Celite pad. The filter cake was washed with additional DCM (25 mL × 2). The combined filtrate was washed with saturated ammonium chloride (50 mL × 2). The combined aqueous layer was futher extracted with DCM (25 mL). The organic layers were combined, dried over magnesium sulfate, filtered, and concentrated in vacuo to afford an oily residue. Purification by flash column chromatography (wet-loading in DCM/hexanes mixtures; 0 to 50% ethyl acetate in hexanes) afforded the desired product as a colorless, viscous gum. 65 mg, 56% yield.

HRMS (ESI/QTOF) m/z: $[M+H]^+$ Calculated for $C_{23}H_{31}O_6S$ 435.1842; Found 435.1881. $[M-THP+H]^+$ Calculated for $C_{18}H_{23}O_5S$ 351.1266; Found 351.1266. $[M-THP-OEt]^+$ Calculated for $C_{16}H_{17}O_4S$ 305.0848; Found 305.0857.

^{1}H NMR (500 MHz, $CDCl_3$) δ 7.88 (s, 1H), 7.61 (s, 1H), 7.22 (s, 1H), 4.59 (dd, *J* = 4.4, 3.0 Hz, 1H), 4.16 (q, *J* = 7.1 Hz, 2H), 3.90 (s, 3H), 3.89 – 3.76 (m, 2H), 3.53 – 3.47 (m, 1H), 3.46 - 3.41 (m, 1H), 3.32 (t, *J* = 6.8 Hz, 2H), 2.83 - 2.73 (m, 2H), 2.78 (t, *J* = 6.7 Hz, 2H), 1.93 (p, *J* = 6.9 Hz, 2H), 1.89 – 1.80 (m, 1H), 1.76 - 1.70 (m, 1H), 1.65 – 1.50 (m, 4H), 1.26 (t, *J* = 7.1 Hz, 3H).

^{13}C NMR (176 MHz, $CDCl_3$) δ 192.27, 172.87, 158.65, 142.79, 140.53, 132.89, 130.49, 129.29, 126.31, 102.77, 99.06, 67.21, 62.57, 60.86, 55.72, 33.75, 30.95, 29.66, 28.59, 27.43, 25.65, 19.86, 14.33.

Compound 28

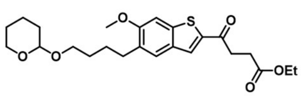

Lithium chloride (66 mg, 1.6 mmol) was weighed in a two-neck flask and dried with heating under vacuum. Xphos Pd G4 (34 mg, 0.039 mmol) was added under nitrogen, followed by the addition of Compound 04 (0.29 g, 0.78 mmol) in THF (5 mL). Previously prepared (4-((tetrahydro-2H-pyran-2-yl)oxy)butyl)zinc(II) bromide (assumed to be 0.5 M in THF,

2.3 mL, 1.2 mmol) was added at room temperature, and the reaction was allowed to proceed at 50 °C for 2.5 hours under nitrogen. The reaction mixture was then diluted in ethyl acetate (50 mL) and filtered through a Celite pad. The filter cake was washed with additional ethyl acetate (20 mL), and the filtrate was washed with saturated ammonium chloride (50 mL × 2). The combined aqueous layer was further extracted with ethyl acetate (25 mL). The organic layers were combined, dried over magnesium sulfate, filtered, and concentrated in vacuo to afford a brown gum. Purification by flash column chromatography (wet-loading in DCM/hexanes, 0 to 50% ethyl acetate in hexanes) afforded the desired product as a pale-green gum. 136 mg, 39% yield.

HRMS (ESI/QTOF) m/z: $[M\text{-}THP+H]^+$ Calculated for $C_{19}H_{25}O_5S$ 365.1423; Found 365.1465. $[M\text{-}THP\text{-}OEt]^+$ Calculated for $C_{17}H_{19}O_4S$ 319.1004; Found 319.1055. $[M+H]^+$ Calculated for $C_{24}H_{32}O_6S$ 449.1998; Found 449.2014.

^1H NMR (700 MHz, CD_2Cl_2) δ 7.91 (s, 1H), 7.64 (s, 1H), 7.27 (s, 1H), 4.55 (t, *J* = 3.4 Hz, 1H), 4.13 (q, *J* = 7.1 Hz, 2H), 3.91 (s, 3H), 3.82 (ddd, *J* = 11.2, 8.3, 3.0 Hz, 1H), 3.74 (dt, *J* = 9.5, 6.6 Hz, 1H), 3.45 (dt, *J* = 10.6, 4.7 Hz, 1H), 3.39 (dt, *J* = 9.6, 6.3 Hz, 1H), 3.30 (t, *J* = 6.7 Hz, 2H), 2.73 (t, *J* = 6.7 Hz, 4H), 1.83 – 1.76 (m, 1H), 1.74 – 1.61 (m, 5H), 1.57 – 1.45 (m, 4H), 1.25 (t, *J* = 7.1 Hz, 3H).

^{13}C NMR (176 MHz, CD_2Cl_2) δ 192.56, 172.92, 159.00, 142.73, 140.80, 133.22, 131.38, 129.64, 126.48, 103.05, 99.18, 67.66, 62.47, 60.99, 55.98, 33.92, 31.21, 30.63, 29.97, 28.73, 26.79, 26.00, 20.07, 14.39.

Compound 29

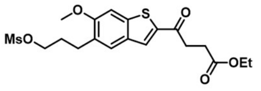

Compound 27 (54 mg, 0.12 mmol) was dissolved in ethanol (2 mL). 4-methylbenzenesulfonic acid (2 mg, 100 mg/mL in ethanol, 20 µL, 11.6 µmol) was added, and the solution was stirred at room temperature overnight until TLC analysis indicated complete deprotection of the THP group. The reaction mixture was diluted in ethyl acetate (50 mL) and washed with saturated sodium bicarbonate (50 mL × 2). The combined aqueous layer was further extracted with ethyl acetate (15 mL). The organic layers were combined, dried over magnesium sulfate, filtered, and concentrated in vacuo to afford 45 mg of white solid residue. The residue was then dissolved in anhydrous DCM (5 mL) under nitrogen. Anhydrous DIPEA (50 µL, 37 mg, 0.29 mmol) was added, and the solution was cooled in an ice bath. Methanesulfonyl chloride (25 µL, 37 mg, 0.32 mmol) was added dropwise, and the reaction mixture was allowed to gradually warm up to room temperature. After 24 hours of stirring, TLC analysis indicated completion of the reaction. The reaction mixture was diluted in DCM (50 mL) and washed with saturated ammonium chloride (50 mL × 2). The combined aqueous layer was extracted with DCM (15 mL). The organic layers were combined, dried over magnesium sulfate, filtered, and concentrated in vacuo to afford a gum-like residue. Purification by flash column chromatography (wet-loading in DCM/hexanes; 0 to 70% ethyl acetate in hexanes) afforded the desired product as a colorless gum. 46 mg, 87% yield over two steps.

HRMS (ESI/QTOF) m/z: $[M+H]^+$ Calculated for $C_{19}H_{25}O_7S_2$ 429.1042; Found 429.1076. $[M\text{-}OEt]^+$ Calculated for $C_{17}H_{19}O_6S_2$ 383.0623; Found 383.0674.

^1H NMR (700 MHz, $CDCl_3$) δ 7.89 (s, 1H), 7.62 (s, 1H), 7.25 (s, 1H), 4.25 (t, *J* = 6.3 Hz, 2H), 4.16 (q, *J* = 7.1 Hz, 2H), 3.92 (s, 3H), 3.32 (t, *J* = 6.8 Hz, 2H), 3.01 (s, 3H), 2.83 (t, *J* = 7.4 Hz, 2H), 2.78 (t, *J* = 6.8 Hz, 2H), 2.12 – 2.05 (m, 2H), 1.26 (t, *J* = 7.2 Hz, 3H).

^{13}C NMR (176 MHz, $CDCl_3$) δ 192.29, 172.83, 158.37, 143.22, 140.91, 132.93, 129.16, 128.75, 126.67, 103.06, 69.59, 60.88, 55.79, 37.56, 33.76, 29.13, 28.55, 26.91, 14.33.

Compound 30

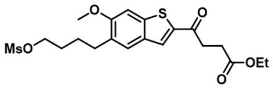

Compound 28 (125 mg, 0.28 mmol) and PTSA (4.8 mg, 0.028 mmol, 48 µL of 100 mg/mL stock in EtOH) were dissolved in ethanol (5 mL) and stirred at room temperature overnight until TLC analysis indicated complete consumption of the starting material. The reaction mixture was diluted in ethyl acetate (50 mL) and washed with saturated sodium bicarbonate (50 mL × 2). The combined aqueous layer was further extracted with ethyl acetate (15 mL). The organic layers were combined, dried over magnesium sulfate, filtered, and concentrated in vacuo. The residue was then dissolved in anhydrous DCM (3 mL) under nitrogen and cooled in an ice bath. DIPEA (0.12 mL, 0.09 g, 0.7 mmol) and methane sulfonyl chloride (43 µL, 64 mg, 0.56 mmol) were added sequentially. The reaction was allowed to gradually warm up to room temperature. After 24 hours, TLC analysis indicated complete consumption of the starting material. The reaction mixture was diluted in DCM (50 mL) and washed with saturated ammonium chloride (50 mL × 2). The combined aqueous layer was extracted with DCM (20 mL). The organic layers were combined, dried over magnesium sulfate, filtered, and concentrated in vacuo. Purification by flash column chromatography (wet-loading in DCM/hexanes; 0 to 70% ethyl acetate in hexanes) afforded the desired product as a colorless gum. 103 mg, 84% yield.

HRMS (ESI/QTOF) m/z: $[M+H]^+$ Calculated for $C_{20}H_{27}O_7S_2$ 443.1199; Found 443.1267. $[M-OEt]^+$ Calculated for $C_{18}H_{21}$O6S2 397.0780; Found 397.0770.

^{1}H NMR (700 MHz, $CDCl_3$) δ 7.88 (s, 1H), 7.58 (s, 1H), 7.23 (s, 1H), 4.26 (t, J = 6.4 Hz, 2H), 4.16 (q, J = 7.2 Hz, 2H), 3.91 (s, 3H), 3.32 (t, J = 6.8 Hz, 2H), 2.99 (s, 3H), 2.78 (t, J = 6.8 Hz, 2H), 2.74 (t, J = 7.5 Hz, 2H), 1.84 – 1.78 (m, 2H), 1.78 – 1.72 (m, 2H), 1.26 (t, J = 7.2 Hz, 3H).

^{13}C NMR (176 MHz, $CDCl_3$) δ 192.28, 172.85, 158.45, 142.95, 140.74, 132.92, 130.05, 129.20, 126.32, 102.93, 70.04, 60.87, 55.77, 37.52, 33.76, 30.00, 28.93, 28.57, 25.71, 14.33.

Compound 31

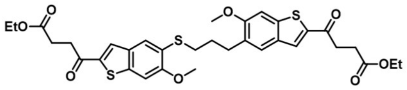

Compound 29 (36 mg, 0.084 mmol) and Compound 08 (38 mg, 0.12 mmol) were dissolved in anhydrous DMF (1.5 mL) under nitrogen. DIPEA (29 µL, 22 mg, 0.17 mmol) was added rapidly, and the resulting dark-red solution was stirred at room temperature for 19 hours. The resulting decolorized reaction mixture with precipitates were dissolved in DCM (50 mL) and washed with aqueous lithium chloride (0.5 M, 50 mL × 2). The combined aqueous layer was further extracted with DCM (25 mL × 1, 10 mL × 1). The organic layers were combined, dried over magnesium sulfate, filtered, and concentrated in vacuo. Purification by flash column chromatography (wet-loading in DCM; 0 to 20% ethyl acetate in DCM/hexanes = 1/1) afforded the desired product as an off-white solid. 37 mg, 67% yield.

HRMS (ESI/QTOF) m/z: $[M+H]^+$ Calculated for $C_{33}H_{37}O_8S_3$ 657.1651; Found 657.1685. $[M-OEt]^+$ Calculated for

$C_{31}H_{31}O_7S_3$ 611.1232; Found 611.1264. $[M\text{-}2OEt+H]^+$ Calculated for $C_{29}H_{25}O_6S_3$ 565.0814; Found 565.0824.

^1H NMR (700 MHz, $CDCl_3$) δ 7.86 (s, 1H), 7.79 (s, 1H), 7.60 (s, 1H), 7.59 (s, 1H), 7.24 (s, 1H), 7.23 (s, 1H), 4.163 (q, *J* = 7.1 Hz, 2H), 4.158 (q, *J* = 7.1 Hz, 2H), 3.95 (s, 3H), 3.87 (s, 3H), 3.31 (t, *J* = 6.6 Hz, 2H), 3.30 (t, *J* = 6.4 Hz, 2H), 2.96 (t, *J* = 7.3 Hz, 2H), 2.88 (t, *J* = 7.3 Hz, 2H), 2.78 (t, *J* = 6.7 Hz, 2H), 2.77 (t, *J* = 6.8 Hz, 2H), 2.01 (p, *J* = 7.3 Hz, 2H), 1.27 (t, *J* = 7.1 Hz, 3H), 1.26 (t, *J* = 7.1 Hz, 3H).

^{13}C NMR (176 MHz, $CDCl_3$) δ 192.15, 192.11, 172.72, 172.69, 158.39, 157.51, 142.86, 142.26, 141.17, 140.64, 133.30, 132.76, 129.54, 129.08, 128.47, 126.41, 125.41, 125.21, 102.93, 102.81, 60.77, 60.74, 56.22, 55.62, 33.66, 33.62, 31.44, 29.77, 28.49, 28.42, 28.38, 14.209, 14.206.

Compound 32

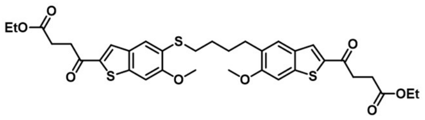

Compound 30 (90 mg, 0.20 mmol) and Compound 08 (79 mg, 0.24 mmol) were dissolved in anhydrous DMF (2 mL) under argon. Anhydrous DIPEA (71 µL, 53 mg, 0.41 mmol) was rapidly added, and the resulting dark-red solution was stirred at room temperature for 19 hours. Upon completion, the now pale-yellow reaction mixture was diluted in DCM (75 mL) and washed with aqueous lithium chloride (0.5 M, 75 mL × 2). The combined organic layer was extracted with DCM (20 mL). The organic layers were combined, dried over magnesium sulfate, filtered, and concentrated in vacuo. Purification by flash column chromatography (wet-loading in DCM, 0 to 20% ethyl acetate in DCM/hexanes = 1/1) afforded the desired product as an off-white solid. 107 mg, 76% yield.

HRMS (ESI/QTOF) m/z: $[M+H]^+$ Calculated for $C_{34}H_{39}O_8S_3$ 671.1808; Found 671.1799. $[M\text{-}OEt]^+$ Calculated for $C_{32}H_{33}O_7S_3$ 625.1389 Found 625.1418. $[M\text{-}2OEt\text{-}H]^+$ Calculated for $C_{30}H_{27}O_6S_3$ 579.0970; Found 579.0941.

^1H NMR (700 MHz, $CDCl_3$) δ 7.852 (s, 1H), 7.850 (s, 1H), 7.66 (s, 1H), 7.56 (s, 1H), 7.23 (s, 1H), 7.22 (s, 1H), 4.16 (q, *J* = 7.2 Hz, 4H), 3.97 (s, 3H), 3.90 (s, 3H), 3.31 (t, *J* = 6.8 Hz, 4H), 2.97 (t, *J* = 7.2 Hz, 2H), 2.784 (t, *J* = 6.8 Hz, 2H), 2.778 (t, *J* = 6.8 Hz, 2H), 2.73 (t, *J* = 7.5 Hz, 2H), 1.82 (p, *J* = 7.3 Hz, 2H), 1.76 (p, *J* = 6.9 Hz, 2H), 1.27 (t, *J* = 7.1 Hz, 6H).

^{13}C NMR (176 MHz, $CDCl_3$) δ 192.28, 192.24, 172.88, 172.84, 158.54, 157.61, 142.79, 142.37, 141.28, 140.63, 133.45, 132.90, 130.52, 129.22, 128.64, 126.20, 125.81, 125.31, 103.05, 102.83, 60.91, 60.87, 56.37, 55.76, 33.80, 33.76, 32.18, 30.12, 29.01, 28.57, 28.52, 28.49, 14.34.

Compound 33

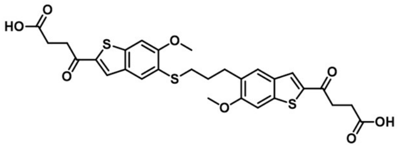

Compound 31 (33 mg, 0.05 mmol) was suspended in a mixture of THF (3 mL) and water (3 mL). Aqueous lithium

hydroxide (1 M, 0.15 mL, 0.15 mmol) was added, and the suspension was stirred at room temperature for several hours. Additional lithium hydroxide (0.2 mL, 0.2 mmol) was added, and the reaction mixture was warmed up to 40 °C and stirred for 2 days. As some starting material still remained, more lithium hydroxide (0.1 mL, 0.1 mmol) along with methanol (2 mL) was added, and the suspension was continuously stirred until it eventually clarified. The solution was then acidified with citric acid (1 M aqueous), and the residual THF was removed in vacuo. The precipitates were collected by vacuum filtration, washed with water (3 mL × 2) and methanol (2 mL × 2), and dried in vacuo to afford the desired product as an off-white powder. 22 mg, 73% yield.

HRMS (ESI/QTOF) m/z: $[M+H]^+$ Calculated for $C_{29}H_{29}O_8S_3$ 601.1025; Found 601.1052. $[M-H_2O+H]^+$ Calculated for $C_{29}H_{27}O_7S_3$ 583.0919; Found 583.0880.

^{1}H NMR (700 MHz, DMSO) δ 12.19 (s, 2H), 8.23 (s, 1H), 8.15 (s, 1H), 7.765 (s, 1H), 7.757 (s, 1H), 7.63 (s, 1H), 7.60 (s, 1H), 3.90 (s, 3H), 3.86 (s, 3H), 3.25 (t, *J* = 6.2 Hz, 4H) (overlapped triplets), 2.98 (t, *J* = 7.2 Hz, 2H), 2.83 (t, *J* = 7.4 Hz, 2H), 2.60 (t, *J* = 6.4 Hz, 2H), 2.59 (t, *J* = 6.4 Hz, 2H), 1.93 (p, *J* = 7.4 Hz, 2H).

^{13}C NMR (176 MHz, DMSO) δ 192.70, 192.68, 173.66, 173.64, 157.96, 156.66, 141.84, 140.77, 140.62, 140.16, 133.26, 132.67, 130.45, 129.83, 129.03, 126.30, 125.17, 123.86, 103.71, 103.61, 56.30, 55.85, 33.15, 33.11, 30.11, 29.07, 28.04, 27.90.

Compound 34

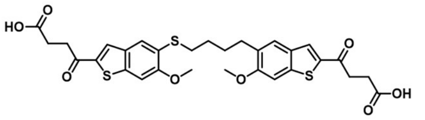

Compound 32 (87 mg, 0.13 mmol) was suspended in a mixture of THF (3 mL) and water (3 mL). Aqueous lithium hydroxide (1 M, 0.52 mL, 0.52 mmol) was added, and the suspension was stirred at room temperature overnight. The clarified solution was then acidified with citric acid (1 M aqueous) to pH 3, and residual THF was removed in vacuo. The precipitates that formed were collected by vacuum filtration, washed with water (3 mL × 2) and methanol (3 mL × 2), and dried in vacuo to afford the desired product as an off-white powder. 56 mg, 70% yield.

HRMS (ESI/QTOF) m/z: $[M+H]^+$ Calculated for $C_{30}H_{31}O_8S_3$ 615.1182; Found 615.1175. $[M-H_2O+H]^+$ Calculated for $C_{30}H_{29}O_7S_3$ 597.1076; Found 597.1099.

^{1}H NMR (700 MHz, DMSO) δ 8.22 (s, 1H), 8.20 (s, 1H), 7.82 (s, 1H), 7.73 (s, 1H), 7.63 (s, 1H), 7.58 (s, 1H), 3.90 (s, 3H), 3.88 (s, 3H), 3.26 (t, *J* = 6.4 Hz, 2H), 3.25 (t, *J* = 6.6 Hz, 2H), 2.99 (t, *J* = 7.2 Hz, 2H), 2.69 (t, *J* = 7.5 Hz, 2H), 2.60 (t, *J* = 5.2 Hz, 2H), 2.59 (t, *J* = 5.5 Hz, 2H), 1.76 (p, *J* = 7.5 Hz, 2H), 1.66 (p, *J* = 7.2 Hz, 2H).

^{13}C NMR (176 MHz, DMSO) δ 192.71, 192.69, 173.68, 157.99, 156.57, 141.66, 140.66, 140.60, 140.10, 133.30, 132.63, 130.35, 129.93, 129.70, 126.17, 125.41, 123.72, 103.65, 103.51, 56.29, 55.85, 33.18, 33.15, 30.36, 29.29, 28.31, 27.96, 27.72.

Compound 35

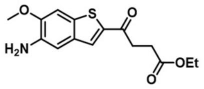

The synthesis was based on published methodologies.[4, 5] Compound 04 (0.5 g, 1.34 mmol), copper (I) iodide (51 mg, 0.27 mmol), sodium ascorbate (53 mg, 0.27 mmol), (1*S*,2*S*)-*N*,*N*-dimethylcyclohexane-1,2-diamine (57 mg, 0.4 mmol) were suspended in a mixture of degassed (3 freeze-pump-thaw cycles) ethanol and water (7/3, v/v, 7.5 mL). The reaction mixture was refluxed under a nitrogen atmosphere for 17.5 hours. Upon cooling to room temperature, the mixture was diluted in water (50 mL) and extracted with DCM (50 mL × 1, 20 mL × 1). The organic layers were combined and washed again with water (50 mL). The combined aqueous layer was extracted once more with DCM (20 mL) to clear the emulsion layer. All organic layers were then combined, dried over magnesium sulfate, filtered, and concentrated in vacuo to afford a black, oily residue, which was then dissolved in a mixture of THF and water (10/1, v/v, 5 mL). Tris(2-carboxyethyl)phosphine hydrochloride (0.46 g, 1.6 mmol) and triethylamine (0.22 mL, 0.16 g, 1.6 mmol) were added in one-shot, and the reaction mixture was stirred at room temperature for 2 hours until the progress had stopped as monitored by TLC analysis. The reaction mixture was then dried over magnesium sulfate, filtered, and concentrated in vacuo. The reddish residue was purified by flash column chromatography (wet loading with DCM; 0 to 10% ethyl acetate in DCM) afforded the desired product as a yellow crystalline solid. 0.2 g, 48% yield.

HRMS (ESI/TOF) m/z: $[M+H]^+$ Calculated for $C_{15}H_{17}NO_4S$ 308.0957; Found 308.1102. $[M-OEt]^+$ Calculated for $C_{13}H_{12}NO_3S$ 262.0538; Found 262.0689. $[2M+Na]^+$ Calculated for $C_{30}H_{34}N_2O_8S_2Na$ 637.1655; Found 637.1855.

^1H NMR (500 MHz, $CDCl_3$): δ 7.75 (s, 1H), 7.14 (s, 1H), 7.08 (s, 1H), 4.15 (q, *J* = 7.2 Hz, 2H), 3.93 (s, 3H), 3.29 (t, *J* = 6.7 Hz, 2H), 2.76 (t, *J* = 6.7 Hz, 2H), 1.25 (t, *J* = 7.3 Hz, 3H).

^{13}C NMR (126 MHz, $CDCl_3$) δ 192.16, 172.88, 150.01, 140.70, 136.00, 134.92, 133.39, 128.68, 108.66, 102.76, 60.80, 55.89, 33.72, 28.57, 14.29.

Compound 36

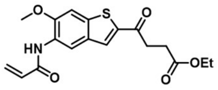

Compound 35 (100 mg, 0.32 mmol) was dissolved in anhydrous DCM (1.5 mL) and cooled in an ice bath. Acryloyl chloride (31.5 µL, 35 mg, 0.39 mmol) was added in one-shot, followed by the addition of triethylamine (136 µL, 99 mg, 0.98 mmol). The mixture was stirred at 0 °C for 5 minutes and allowed to gradually warm up over 1.5 hours. Upon completion, the reaction mixture was diluted in DCM (30 mL) and washed with saturated ammonium chloride (50 mL). The aqueous layer was extracted once more with DCM (30 mL). The organic layers were combined, dried over magnesium sulfate, filtered, and concentrated in vacuo. Purification by flash column chromatography (wet loading in DCM; 0 to 10% ethyl acetate in DCM) afforded the desired product as a pale-yellow solid. 102 mg, 87% yield.

HRMS (ESI/TOF) m/z: $[M+H]^+$ Calculated for $C_{18}H_{20}NO_5S$ 362.1062; Found 362.1260. $[M-OEt]^+$ Calculated for $C_{16}H_{14}NO_4S$ 316.0644; Found 316.0824. $[2M+Na]^+$ Calculated for $C_{36}H_{38}N_2O_{10}S_2Na$ 745.1866; Found 745.2120.

^1H NMR (500 MHz, $CDCl_3$): δ 9.00 (s, 1H), 8.00 (s, 1H), 7.90 (s, 1H), 6.45 (dd, *J* = 16.8, 1.4 Hz, 1H), 6.31 (dd, *J* = 16.9, 10.2 Hz, 1H), 5.79 (dd, *J* = 10.2, 1.2 Hz, 1H), 4.16 (q, *J* = 7.1 Hz, 2H), 3.99 (s, 3H), 3.32 (t, *J* = 6.8 Hz, 2H), 2.77 (t, *J* = 6.7 Hz, 2H), 1.26 (t, *J* = 7.1 Hz, 3H).

^{13}C NMR (126 MHz, $CDCl_3$) δ 192.28, 172.77, 163.57, 149.28, 141.67, 139.09, 133.20, 131.46, 129.85, 127.91, 126.89, 115.92, 102.82, 60.87, 56.34, 33.74, 28.51, 14.33.

Compound 37

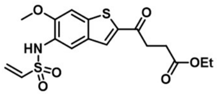

Compound 35 (93 mg, 0.3 mmol) was dissolved in anhydrous DCM (3 mL) and cooled in an ice bath. Ethenesulfonyl chloride (33 µL, 45 mg, 0.36 mmol) was added in one-shot, followed by the addition of triethylamine (125 µL, 91 mg, 0.9 mmol). The reaction mixture was stirred at room temperature for 5 minutes and allowed to gradually warm up over 2.5 hours. Upon completion, the mixture was partitioned between DCM (50 mL) and brine (50 mL). The aqueous layer was extracted again with DCM (30 mL). The organic layers were combined, dried over magnesium sulfate, filtered, and concentrated in vacuo. Purification by flash column chromatography (wet loading in DCM; 0 to 20% ethyl acetate in hexanes) to afford the desired product as an off-white solid. 62 mg, 52% yield.

HRMS (ESI/TOF) m/z: $[M+H]^+$ Calculated for $C_{17}H_{20}NO_6S_2$ 398.0732; Found 398.0939. $[M-OEt]^+$ Calculated for $C_{15}H_{14}NO_5S_2$ 352.0314; Found 352.0475. $[2M+Na]^+$ Calculated for $C_{34}H_{38}N_2O_{12}S_4Na$ 817.1206; Found 817.1418.

^1H NMR (500 MHz, $CDCl_3$): δ 7.94 (s, 1H), 7.91 (s, 1H), 7.28 (s, 1H), 6.94 (s, 1H), 6.51 (dd, *J* = 16.5, 9.9 Hz, 1H), 6.27 (d, *J* = 16.4 Hz, 1H), 5.92 (d, *J* = 9.9 Hz, 1H), 4.16 (q, *J* = 7.2 Hz, 2H), 3.97 (s, 3H), 3.31 (t, *J* = 6.6 Hz, 2H), 2.78 (t, *J* = 6.7 Hz, 2H), 1.27 (t, *J* = 7.2 Hz, 3H).

^{13}C NMR (126 MHz, $CDCl_3$) δ 192.26, 172.73, 150.27, 142.23, 140.38, 134.79, 133.03, 129.30, 128.70, 125.37, 116.48, 103.58, 60.92, 56.44, 33.74, 28.48, 14.34.

Compound 38

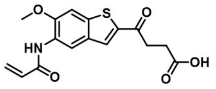

To Compound 36 (90 mg, 0.25 mmol) suspended in THF (4 mL) was added an aqueous solution of lithium hydroxide monohydrate (23 mg, 0.55 mmol, 2 mL). The reaction mixture was stirred at room temperature for 2 hours until TLC analysis indicated complete consumption of the starting material. THF was removed in vacuo, and the residual solution was acidified to pH 3 with citric acid (1 M aqueous). The resulting solids were collected by vacuum filtration, washed with water (2 mL × 6), and dried in vacuo to afford the desired product as a pale-yellow powder. 72 mg, 87% yield.

HRMS (ESI/TOF) m/z: $[M+H]^+$ Calculated for $C_{16}H_{16}NO_5S$ 334.0749; Found 334.0920. $[M-OH]^+$ Calculated for $C_{16}H_{14}NO_4S$ 316.0644; Found 316.0809. $[2M+Na]^+$ Calculated for $C_{32}H_{30}N_2O_{10}S_2Na$ 689.1240; Found 689.1488.

^1H NMR (500 MHz, DMSO): δ 12.19 (s, 1H), 9.53 (s, 1H), 8.71 (s, 1H), 8.32 (s, 1H), 7.72 (s, 1H), 6.76 (dd, *J* = 16.8, 10.5 Hz, 1H), 6.28 (dd, *J* = 17, 2 Hz, 1H), 5.76 (dd, *J* = 10, 2 Hz, 1H), 3.96 (s, 3H), 3.28 (t, *J* = 6.4 Hz, 2H), 2.60 (t, *J* = 6.4 Hz, 2H).

^{13}C NMR (126 MHz, DMSO): δ 192.74, 173.67, 163.56, 150.89, 140.87, 138.67, 132.44, 131.94, 130.93, 126.95, 126.60, 117.94, 104.06, 56.29, 33.15, 27.91.

Compound 39

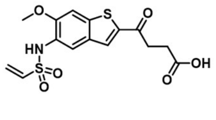

To Compound 37 (55 mg, 0.14 mmol) dissolved in THF (3 mL) was added an aqueous solution of lithium hydroxide monohydrate (13.4 mg, 0.32 mmol, 2 mL). The reaction was allowed to proceed at room temperature for 3 hours, at which time additional lithium hydroxide monohydrate (10 mg, 0.24 mmol, 0.5 mL) was added. The mixture was stirred for another 1 hour until TLC analysis indicated complete consumption of the starting material. Citric acid (1 M aqueous) was added dropwise to acidify the solution to pH 3, and THF was removed in vacuo. The resulting solids were collected by vacuum filtration, washed with water (2 mL × 4), and dried in vacuo to afford the desired product as a yellow crystalline solid. 43 mg, 84% yield.

HRMS (ESI/TOF) m/z: $[M+H]^+$ Calculated for $C_{15}H_{16}NO_6S_2$ 370.0419; Found 370.0549. $[2M+Na]^+$ Calculated for $C_{30}H_{30}N_2O_{12}S_4Na$ 761.0580; Found 761.0761. $[3M+K]^+$ $C_{45}H_{45}N_3O_{18}S_6K$ 1146.0660; Found 1146.0590. $[4M+K]^+$ Calculated for $C_{60}H_{60}N_4O_{24}S_8K$ 1515.1002; Found 1515.0935.

^{1}H NMR (500 MHz, DMSO): δ 12.19 (s, 1H), 9.31 (s, 1H), 8.33 (s, 1H), 7.85 (s, 1H), 7.70 (s, 1H), 6.77 (dd, *J* = 16.3, 9.9 Hz, 1H), 6.00 (d, *J* = 16.6 Hz, 1H), 5.95 (d, *J* = 9.8 Hz, 1H), 3.89 (s, 3H), 3.26 (t, *J* = 6.4 Hz, 2H), 2.60 (t, J = 6.3 Hz, 2H).

^{13}C NMR (126 MHz, DMSO): δ 192.76, 173.63, 153.08, 141.08, 140.52, 136.54, 132.47, 130.76, 126.49, 125.15, 121.38, 104.72, 56.27, 33.13, 27.90.

Compound 40

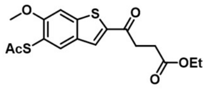

The synthesis was based on a published methodology.[2] Compound 04 (0.34 g, 0.92 mmol), potassium thioacetate (0.52 g, 4.55 mmol), $Pd_2(dba)_3$ (84 mg, 0.092 mmol), Xantphos (53 mg, 0.092 mmol), and DIPEA (0.48 mL, 0.36 g, 2.76 mmol) were suspended in 1,4-dioxane (2 mL). The mixture was degassed (3 freeze-pump-thaw cycles) and refluxed under argon for 17 hours. Upon cooling to room temperature, the reaction mixture was diluted in DCM (50 mL) and filtered through a Celite pad. The filtrate was washed once with water (50 mL), and the aqueous layer was extracted again with DCM (30 mL). The organic layers were combined, dried over magnesium sulfate, filtered, and concentrated in vacuo to afford an oily residue. Purification by flash column chromatography (wet loading in DCM; 0 to 5% ethyl acetate in DCM/hexanes = 1/1) to afford the desired product as a pale-red oil which solidified upon standing at -20 °C. 0.24 g, 72% yield.

HRMS (ESI/TOF) m/z: $[M+H]^+$ Calculated for $C_{17}H_{19}O_5S_2$ 367.0674; Found 367.0792. $[M-OEt]^+$ Calculated for $C_{15}H_{13}O_4S_2$ 321.0256; Found 321.0366. $[2M+Na]^+$ Calculated for $C_{34}H_{36}O_{10}S_4Na$ 755.1090; Found 755.1236.

^{1}H NMR (500 MHz, $CDCl_3$) δ 7.90 (s, 1H), 7.88 (s, 1H), 7.34 (s, 1H), 4.16 (q, *J* = 7.1 Hz, 2H), 3.92 (s, 3H), 3.31 (t, *J* = 6.8 Hz, 2H), 2.77 (t, *J* = 6.8 Hz, 2H), 2.44 (s, 3H), 1.26 (t, *J* = 7.1 Hz, 3H).

^{13}C NMR (126 MHz, $CDCl_3$) δ 193.35, 192.21, 172.68, 158.55, 146.52, 141.81, 134.16, 133.24, 128.87, 116.43, 104.20, 60.88, 56.49, 33.76, 30.23, 28.44, 14.30.

Compound 41

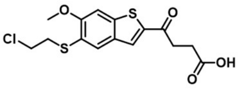

To Compound 40 (0.1 g, 0.27 mmol) under a nitrogen atmosphere was added a degassed (3 freeze-pump-thaw cycles) solution of lithium hydroxide monohydrate (62 mg, 1.48 mmol) in THF (3 mL) and water (4 mL). The reaction was allowed to proceed at room temperature for an hour until LC-MS analysis indicated complete hydrolysis of both the thioester and the ester functional groups. 1-Bromo-2-chloroethane (69 µL, 119 mg, 0.83 mmol) was then added portionwise, and the alkylation was allowed to proceed for 2 hours at room temperature. THF was then removed in vacuo, and the remaining solution was acidified to pH 4 with citric acid (1 M aqueous). The resulting solids were collected by vacuum filtration, washed with water (2 mL × 5), and dried in vacuo to afford an orange powder. Subsequent purification by flash column chromatography (wet loading in 5% MeOH/DCM; 0 to 16% MeOH in DCM) afforded the desired product as an off-white solid. 42 mg, 43% yield.

HRMS (ESI/TOF) m/z: $[M+H]^+$ Calculated for $C_{15}H_{16}ClO_4S_2$ 359.0179; Found 359.0185. $[2M+Na]^+$ Calculated for $C_{30}H_{30}Cl_2O_8S_4Na$ 739.0099; Found 739.0093. $[3M+K]^+$ Calculated for $C_{45}H_{45}Cl_3O_{12}S_6K$ 1114.9909; Found 1114.9813.

^{1}H NMR (500 MHz, DMSO): δ 12.23 (s, 1H), 8.26 (s, 1H), 7.95 (s, 1H), 7.69 (s, 1H), 3.93 (s, 3H), 3.78 (t, J = 7.3 Hz, 2H), 3.33 (t, J = 7.3 Hz, 2H), 3.27 (s, J = 6.5 Hz, 2H), 2.60 (t, J = 6.4 Hz, 2H).

^{13}C NMR (126 MHz, DMSO): δ 192.75, 173.65, 156.91, 141.71, 140.82, 133.26, 130.04, 125.45, 123.01, 104.13, 56.41, 42.82, 33.26, 33.16, 27.92.

Compound 42

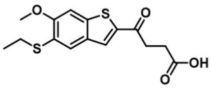

To Compound 40 (96 mg, 0.26 mmol) under a nitrogen atmosphere was injected a degassed (3 freeze-pump-thaw cycles) mixture of aqueous lithium hydroxide (1 M, 1.31 mL, 1.31 mmol), water (1.69 mL), and THF (2 mL). The mixture was stirred for 1 hour at room temperature, at which point bromoethane (59 µL, 86 mg, 0.8 mmol) was injected in one-shot. After another 0.5 hour the reaction mixture was acidified with citric acid (1 M aqueous), resulting in the formation of some gum-like solids. The solids were collected by vacuum filtration, washed with water (1 mL × 2), and redissolved in DCM containing 5% (v/v) MeOH. The solution was dried over magnesium sulfate, filtered, and concentrated in vacuo to afford a flake-like residue. Purification by flash column chromatography (wet loading in DCM containing a trace amount of MeOH; 0 to 10% MeOH in DCM) afforded 53 mg of pale yellow solid. The solid was dissolved in DMSO and further purified by reversed-phase chromatography (Agilent C18 Bond-elute C18 cartridge; 0 to 100% acetonitrile in water; 0.1% v/v formic acid). Lyophilization of the collected fractions afforded the desired product as a white crystalline solid. 28 mg, 34% yield.

HRMS (ESI/TOF) m/z: $[M+Na]^+$ Calculated for $C_{15}H_{16}O_4S_2Na$ 347.0388; Found 347.0397.

^{1}H NMR (500 MHz, DMSO) δ 8.24 (s, 1H), 7.82 (s, 1H), 7.64 (s, 1H), 3.91 (s, 3H), 3.26 (t, *J* = 6.3 Hz, 2H), 2.97 (q, *J* = 7.3 Hz, 2H), 2.60 (t, *J* = 6.3 Hz, 2H), 1.28 (t, *J* = 7.3 Hz, 3H).

^{13}C NMR (126 MHz, DMSO) δ 192.72, 173.66, 156.47, 140.61, 140.59, 133.32, 129.95, 125.34, 123.50, 103.65, 56.30, 33.17, 27.97, 24.54, 13.61.

Compound 43

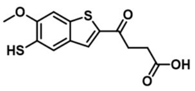

To a solution of Compound 40 (0.22 g, 0.6 mmol) in THF (5 mL) was added a degassed (nitrogen-purged) aqueous solution of lithium hydroxide (1 M, 3 mL, 3 mmol) under a nitrogen atmosphere at room temperature. The reaction was allowed to proceed for 3 hours until TLC analysis indicated complete consumption of the starting material. THF was removed in vacuo, and the residual solution was acidified by dropwise addition of citric acid (1M aqueous) until a large amount of precipitate formed. The precipitate was collected by vacuum filtration, washed with water (5 mL × 3), and dried in vacuo to afford the desired product as an orange solid. 155 mg, 87% yield.

HRMS (ESI/TOF) m/z: $[M+H]^+$ Calculated for $C_{13}H_{13}O_4S_2$ 297.0256; Found 297.0233. $[M+Na]^+$ Calculated for $C_{13}H_{12}O_4S_2Na$ 319.0075; Found 319.0154. $[M-OH]^+$ Calculated for $C_{13}H_{11}O_3S_2$ 279.0150; Found 279.0155. $[2M+Na]^+$ Calculated for $C_{26}H_{24}O_8S_4Na$ 615.0252; Found 615.0367. $[2M-H_2+H]^+$ Calculated for $C_{26}H_{23}O_8S_4$ 591.0276; Found 591.0338. $[3M+K]^+$ Calculated for $C_{39}H_{36}O_{12}S_6K$ 927.0169; Found 927.0170.

^{1}H NMR (400 MHz, DMSO) δ 12.19 (s, 1H), 8.20 (s, 1H), 7.96 (s, 1H), 7.65 (s, 1H), 5.15 (s, 1H), 3.93 (s, 3H), 3.27 (t, *J* = 6.4 Hz, 2H), 2.60 (t, *J* = 6.3 Hz, 2H).

^{13}C NMR (126 MHz, DMSO-d_6): δ 192.77, 173.66, 155.38, 140.84, 140.49, 133.20, 129.60, 125.16, 121.47, 104.01, 56.39, 33.19, 27.89.

Compound 44

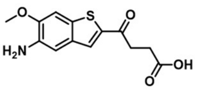

To a solution of Compound 35 (54 mg, 0.18 mmol) in THF (5 mL) was added an aqueous solution of lithium hydroxide monohydrate (40 mg, 0.95 mmol, 3 mL). The reaction was allowed to proceed at room temperature for 1.5 hours. Upon complete consumption of the starting material as indicated by TLC analysis, THF was removed in vacuo. The residual solution was acidified to pH 5 by dropwise addition of citric acid (1 M aqueous). The resulting solids were collected by vacuum filtration, washed with water (2 mL × 3), and dried in vacuo to afford the desired product as a bright-yellow solid. 38 mg, 77% yield.

HRMS (ESI/TOF) m/z: $[M-OH]^+$ Calculated for $C_{13}H_{12}NO_3S$ 262.0538; Found 262.0501. $[M+H]^+$ Calculated for $C_{13}H_{14}NO_4S$ 280.0644; Found 280.0600. $[M+Na]^+$ $C_{13}H_{13}NO_4SNa$ 302.0463; Found 302.0404.

^{1}H NMR (500 MHz, DMSO): δ 12.16 (s, 1H), 8.08 (s, 1H), 7.40 (s, 1H), 7.12 (s, 1H), 5.00 (s, 2H), 3.88 (s, 3H), 3.24 (t, *J* = 6.4 Hz, 2H), 2.58 (t, *J* = 6.4 Hz, 2H).

^{13}C NMR (126 MHz, DMSO): δ 192.44, 173.71, 149.45, 139.74, 137.47, 133.43, 131.86, 129.91, 107.04, 102.95, 55.74, 33.06, 27.96.

Compound 45

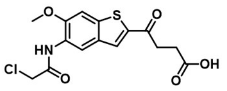

Compound 44 (24 mg, 0.08 mmol) and DIPEA (45 µL, 33 mg, 0.26 mmol) were dissolved in anhydrous THF (1.5 mL) and cooled in an ice bath. Chloroacetyl chloride (20 µL, 28 mg, 0.25 mmol) was added dropwise over 5 minutes. The reaction mixture was then allowed to warm up to room temperature over 45 minutes, during which time its color changed from orange to dark purple. Upon completion of the reaction confirmed by LC-MS analysis, the crude mixture was diluted in DCM (50 mL) and washed with saturated ammonium chloride (50 mL). The aqueous layer was extracted once more with DCM (50 mL), and the organic layers were combined, dried over magnesium sulfate, filtered, and concentrated in vacuo to afford a pink solid residue. Purification by flash column chromatography (wet loading in 5% MeOH/DCM; 0 to 10% MeOH in DCM) to afford the desired product as a pink solid. 21 mg, 67% yield.

HRMS (ESI/TOF) m/z: $[M+Na]^+$ Calculated for $C_{15}H_{14}ClNO_5SNa$ 378.0179; Found 378.0191. $[2M+Na]^+$ Calculated for $C_{30}H_{28}Cl_2N_2O_{10}S_2Na$ 733.0461; Found 733.0488.

^{1}H NMR (500 MHz, DMSO): δ 12.18 (s, 1H), 9.66 (s, 1H), 8.65 (s, 1H), 8.34 (s, 1H), 7.74 (s, 1H), 4.44 (s, 2H), 3.97 (s, 3H), 3.28 (t, *J* = 6.3 Hz, 2H), 2.60 (t, *J* = 6.3 Hz, 2H).

^{13}C NMR (126 MHz, DMSO): δ 192.75, 173.66, 165.06, 150.58, 141.01, 138.88, 132.42, 130.94, 126.11, 117.41, 104.21, 56.40, 43.42, 33.15, 27.89.

Compound 46

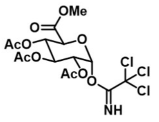

Methyl 2,3,4-tri-*O*-acetyl-beta-*D*-glucopyranuronate (0.49 g, 1.46 mmol) and trichloroacetonitrile (1.46 mL, 2.10 g, 14.6 mmol) were dissolved in anhydrous DCM (3 mL). The solution was cooled in an ice bath, and a catalytic amount of DBU (22 µL, 22 mg, 0.15 mmol) was injected dropwise. The reaction mixture was stirred for 50 minutes at 0 °C and was allowed to gradually warm up to room temperature over an hour. The crude mixture was directly loaded onto silica gel and purified by flash column chromatography (20 to 50% ethyl acetate in hexanes) to afford the desired α-isomer as a white solid. 0.57 g, 82% yield.

HRMS (ESI/TOF) m/z: $[M+Na]^+$ Calculated for $C_{15}H_{18}Cl_3NO_{10}Na$ 501.9865; Found 501.9883. $[2M+Na]^+$ Calculated for $C_{30}H_{36}Cl_6N_2O_{20}Na$ 978.9862; Found 978.9887.

^{1}H NMR (500 MHz, $CDCl_3$): δ 8.73 (s, 1H), 6.64 (d, *J* = 4.1 Hz, 1H), 5.62 (t, *J* = 9.9 Hz, 1H), 5.27 (dd, *J* = 10.2, 9.7 Hz, 1H), 5.15 (dd, *J* = 10.2, 3.6 Hz, 1H), 4.50 (d, *J* = 10.2 Hz, 1H), 3.75 (s, 3H), 2.05 (s, 3H), 2.04 (s, 3H), 2.01 (s, 3H).

^{13}C NMR (126 MHz, $CDCl_3$) δ 169.91, 169.85, 169.60, 167.27, 160.71, 92.75, 90.64, 70.60, 69.58, 69.22, 69.07, 53.17,

20.79, 20.61, 20.53.

Compound 47

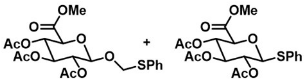

The synthesis was based on a published method.[6] Paraformaldehyde (1 g, 33 mmol) and thiophenol (3.43 mL, 3.69 g, 33 mmol) in the presence of a catalytic amount of sodium ethoxide (21% in ethanol, 3 drops) were stirred at 110 °C for 0.5 hour under a nitrogen atmosphere, leading to a colorless oil. The obtained (phenylthio)methanol was used without further purifications. Compound 46 (1.35 g, 2.82 mmol) and (phenylthio)methanol (1.18 g, 8.42 mmol) were dissolved in anhydrous DCM (6 mL) in the presence of activated 4Å molecular sieves in a two-neck flask. The suspension was stirred under a nitrogen atmosphere at room temperature for 0.5 hour and then cooled in ice bath. Silver triflate (53 mg, 0.21 mmol) was added by briefly removing the septum, and the reaction mixture was stirred for another hour at 0 °C. The suspension was then filtered through a Celite pad. The pad was washed with DCM (40 mL). The filtrate was transferred to a separatory funnel and washed with saturated sodium bicarbonate (30 mL × 2). The combined aqueous layer was extracted once more with DCM (25 mL). The organic layers were combined, dried over magnesium sulfate, filtered, and concentrated in vacuo to afford an oily residue. Purification by flash column chromatography (wet loading in DCM; 0 to 50% ethyl acetate in hexanes) afforded a colorless oil containing the desired product along with the thioglycoside side product as an inseparable mixture. 0.73 g, 56% yield (mixture).

HRMS (ESI/TOF) m/z: $[M+Na]^+$ Calculated for $C_{20}H_{24}O_{10}SNa$ 479.0988; Found 479.0987. $[M+NH_4]^+$ Calculated for $C_{20}H_{28}NO_{10}S$ 474.1434; Found 474.1431.

Compound 48

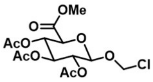

The synthesis was based on a published method.[6] To a solution of Compound 47 (0.73 g, 1.6 mmol) in anhydrous DCM (8 mL) was added sulfuryl chloride (143 µL, 0.24 g, 1.77 mmol) dropwise over the course of 5 minutes at 0 °C. The resulting bright-yellow solution was stirred at 0 °C for another 20 minutes. The crude reaction was then diluted in DCM (25 mL) and washed with saturated sodium bicarbonate (25 mL × 2). The combined aqueous layer was extracted once more with DCM (25 mL). The organic layers were combined, dried over magnesium sulfate, filtered, and concentrated in vacuo. Purification by flash column chromatography (wet loading in DCM; 0 to 50% ethyl acetate in hexanes; silica gel pre-deactivated by flushing ethyl acetate containing 1% TEA through; eluent was TEA-free) to afford the desired product as a colorless oil, which solidified upon standing at -20 °C. 0.46 g, 75% yield.

HRMS (ESI/TOF) m/z: $[M+Na]^+$ Calculated for $C_{14}H_{19}ClO_{10}Na$ 405.0565; Found 405.0557. $[M+NH_4]^+$ Calculated for $C_{14}H_{23}ClNO_{10}$ 400.1011; Found 400.1004.

^{1}H NMR (500 MHz, CD_2Cl_2): δ 5.59 (d, J = 6.5 Hz, 1H), 5.51 (d, J = 6.5 Hz, 1H), 5.30 (t, J = 9.3 Hz, 1H), 5.17 (t, J = 9.6 Hz, 1H), 5.01 (dd, J = 9.1, 7.6 Hz, 1H), 4.14 (d, J = 9.8 Hz, 1H), 3.72 (s, 3H), 2.04 (s, 3H), 2.01 (s, 3H), 2.00 (s, 3H).

^{13}C NMR (126 MHz, CD_2Cl_2): δ 170.10, 169.77, 169.56, 167.34, 97.20, 78.02, 73.03, 71.76, 70.62, 69.49, 53.24, 20.78,

20.76, 20.67.

Compound 49

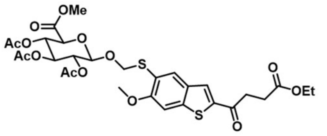

Compound 08 (55 mg, 0.17 mmol) and compound 48 (75 mg, 0.2 mmol) were dissolved in anhydrous DMF (3 mL). DIPEA (59 µL, 44 mg, 0.34 mmol) was injected dropwise under a nitrogen atmosphere at room temperature. The reaction was allowed to proceed for 1.5 hours until LC-MS analysis indicated completion of the reaction. The crude mixture was directly loaded onto silica gel and purified by flash column chromatography (0 to 50% ethyl acetate in hexanes). The collected fractions were concentrated in vacuo, redissolved in DCM, and washed with water (50 mL × 2) to remove co-eluted DMF. The organic layer was dried over magnesium sulfate, filtered, and concentrated in vacuo to afford the desired product as a white solid. 90 mg, 79% yield.

HRMS (ESI/TOF) m/z: $[M+H]^+$ Calculated for $C_{29}H_{35}O_{14}S_2$ 671.1469; Found 671.1520.

^{1}H NMR (500 MHz, CD_2Cl_2): δ 7.92 (s, 1H), 7.91 (s, 1H), 7.30 (s, 1H), 5.42 (d, *J* = 12.3 Hz, 1H), 5.26 (t, *J* = 9.5 Hz, 1H), 5.14 (t, *J* = 9.6 Hz, 1H), 5.14 (d, *J* =12.6 Hz, 1H), 5.09 (d, *J* = 7.8 Hz, 1H), 4.98 (dd, *J* = 9.4, 7.8 Hz, 1H), 4.12 (q, *J* = 7.1 Hz, 2H), 4.06 (d, *J* = 9.8 Hz, 1H), 3.96 (s, 3H), 3.72 (s, 3H), 3.30 (t, *J* = 6.6 Hz, 2H), 2.73 (t, *J* = 6.6 Hz, 2H), 2.00 (s, 3H), 1.95 (s, 3H), 1.46 (s, 3H), 1.24 (t, *J* = 7.2 Hz, 3H).

^{13}C NMR (126 MHz, CD_2Cl_2): δ 192.59, 172.78, 170.09, 169.78, 169.36, 167.54, 157.45, 143.12, 141.96, 133.84, 129.00, 126.25, 123.40, 103.59, 96.33, 72.82, 72.04, 70.88, 70.34, 69.76, 60.98, 56.66, 53.16, 33.95, 28.64, 20.71, 20.65, 20.24, 14.37.

Compound 50

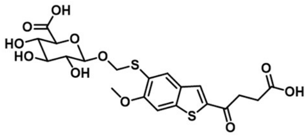

To Compound 49 (90 mg, 0.13 mmol) dissolved in THF (4 mL) was added aqueous lithium hydroxide (1 M, 1.34 mL, 1.34 mmol) at 0 °C. The reaction was allowed to proceed at 0 °C for 2 hours until LC-MS analysis confirmed complete hydrolysis of all the protecting groups. The mixture was acidified with citric acid (1 M aqueous) to pH 4 and THF was removed in vacuo. The resulting slightly opaque solution was purified by reverse-phase column chromatography (Agilent C18 Bond-elute C18 cartridge; eluted with 0 to 40% acetonitrile in water; 0.1% formic acid, v/v). The collected fractions were lyophilized to afford the desired product as a flake-like white solid. 51 mg, 76% yield.

HRMS (ESI/TOF) m/z: $[M-\beta GlcAOH]^+$ Calculated for $C_{14}H_{13}O_4S_2$ 309.0256; Found 309.0250. $[M+H]^+$ Calculated for $C_{20}H_{23}O_{11}S_2$ 503.0682; Found 503.0679.

^{1}H NMR (500.2 MHz, DMSO): δ 8.22 (s, 1H), 8.21 (s, 1H), 7.66 (s, 1H), 5.28(d, *J* = 5.0 Hz, 1H), 5.26 (d, *J* = 11.9 Hz, 1H), 5.15 (s, 1H), 5.12 (d, *J* = 11.9 Hz, 1H), 4.62 (d, *J* = 7.8 Hz, 1H), 3.92 (s, 3H), 3.64 (d, *J* = 9.8 Hz, 1H), 3.35 (t, *J* =

9.3 Hz, 1H), 3.28 (t, *J* = 6.4 Hz, 2H), 3.21 (t, *J* = 9.0 Hz, 1H), 3.09 (td, *J* = 8.4, 4.8 Hz, 1H), 2.61 (t, *J* = 6.3 Hz, 2H).

^{13}C NMR (125.79 MHz, DMSO): δ 192.71, 173.65, 170.30, 156.36, 141.50, 140.65, 133.38, 130.16, 125.79, 124.10, 103.72, 99.81, 75.98, 75.77, 72.87, 71.51, 69.75, 56.37, 33.14, 27.89.

NMR Spectra

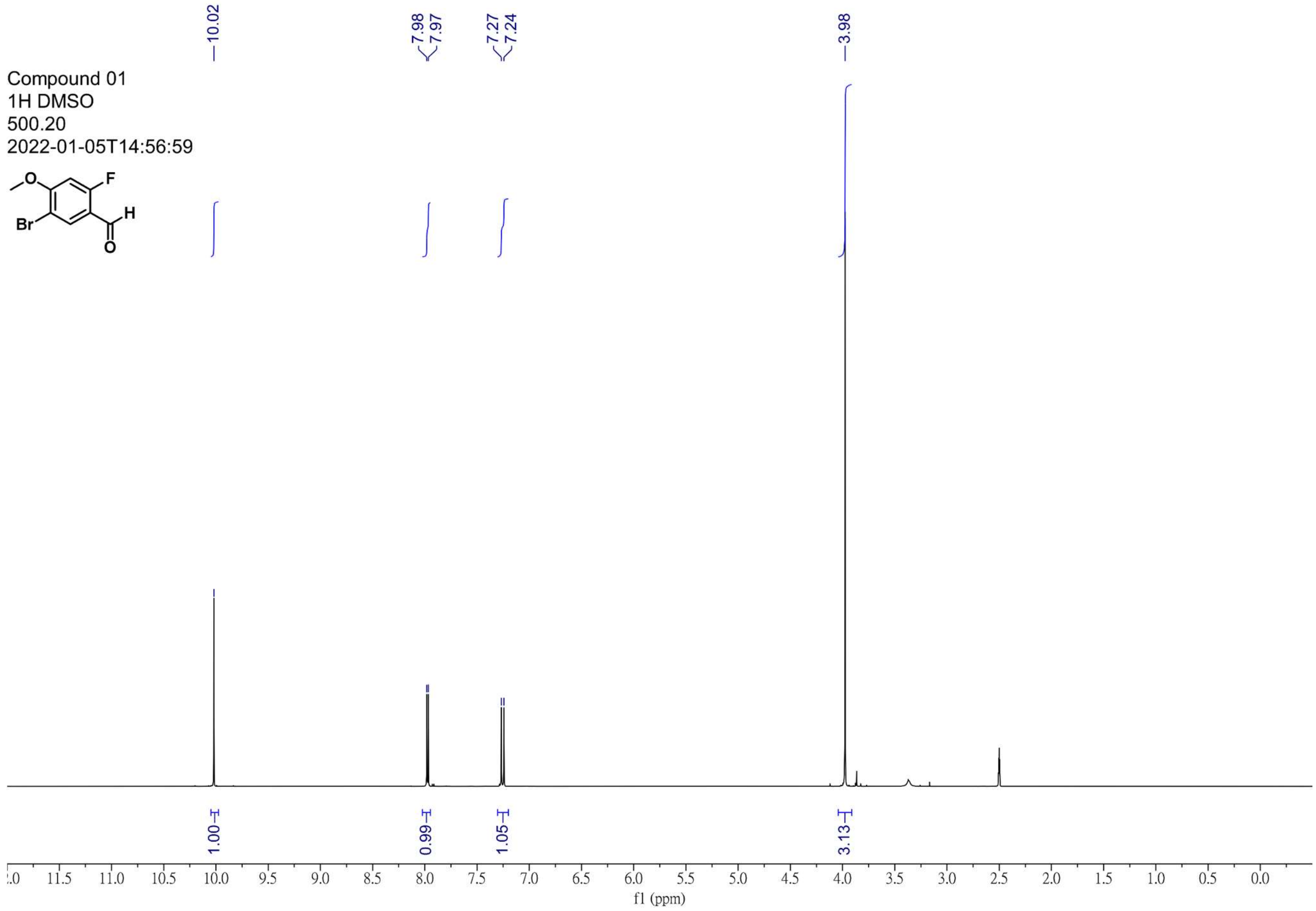
10.02
7.98
7.97
7.27
7.24
3.98
Compound 01
1H DMSO
500.20
2022-01-05T14:56:59
F
H
Br
O
1.00
0.99
1.05
3.13
f1 (ppm)

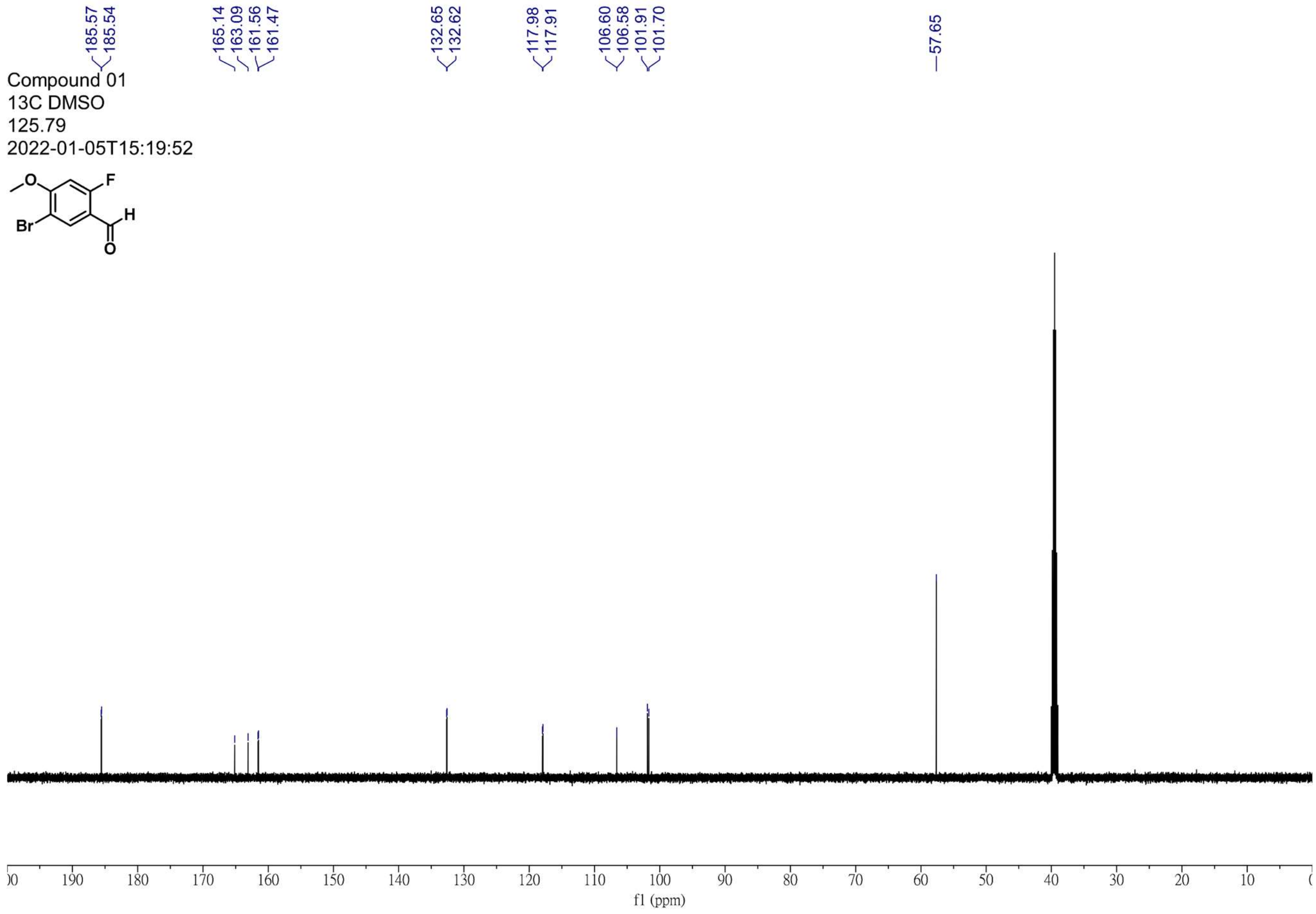
185.57
185.54
165.14
163.09
161.56
161.47
132.65
132.62
117.98
117.91
106.60
106.58
101.91
101.70
57.65
Compound 01
13C DMSO
125.79
2022-01-05T15:19:52
F
H
Br
O
f1 (ppm)

Compound 02
1H DMSO
500.20
2022-01-08T15:56:49

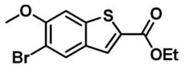

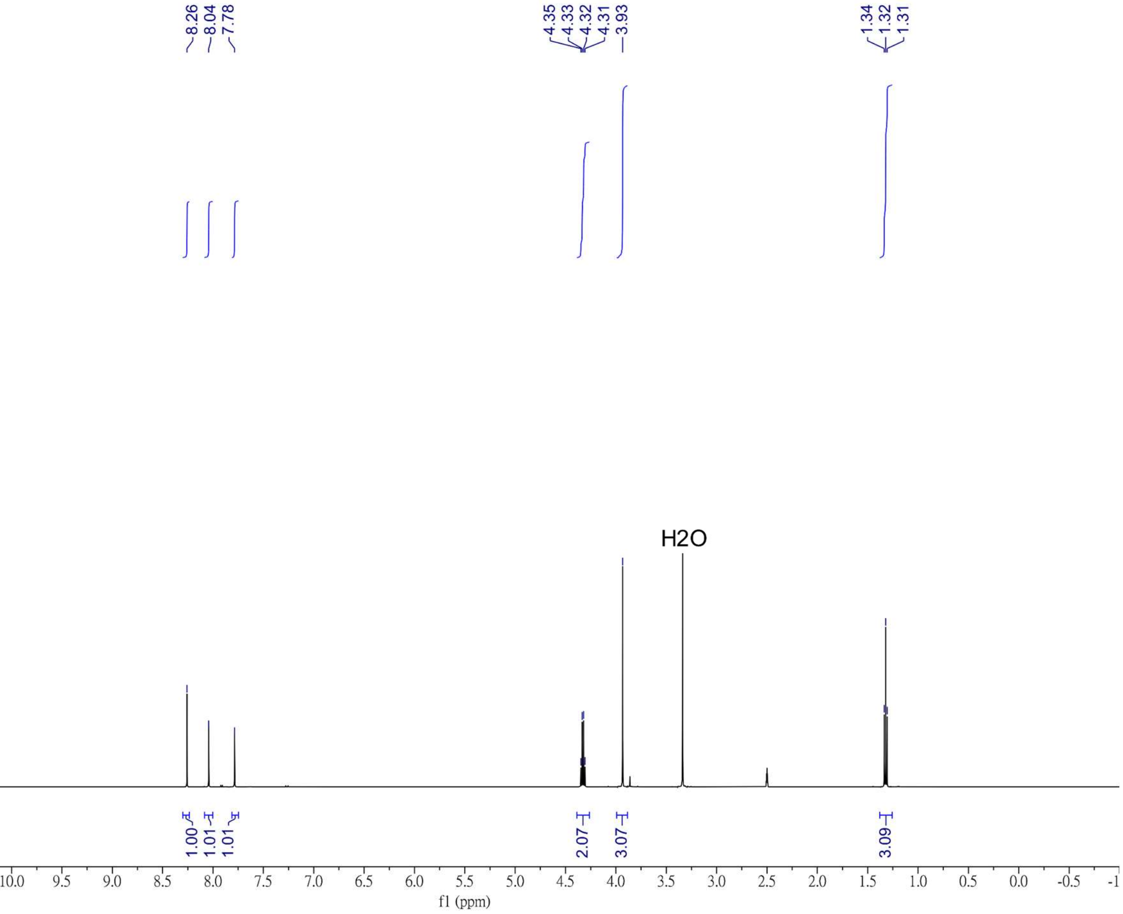

Compound 02
13C DMSO
125.79
2022-01-08T16:05:58

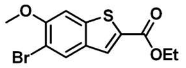

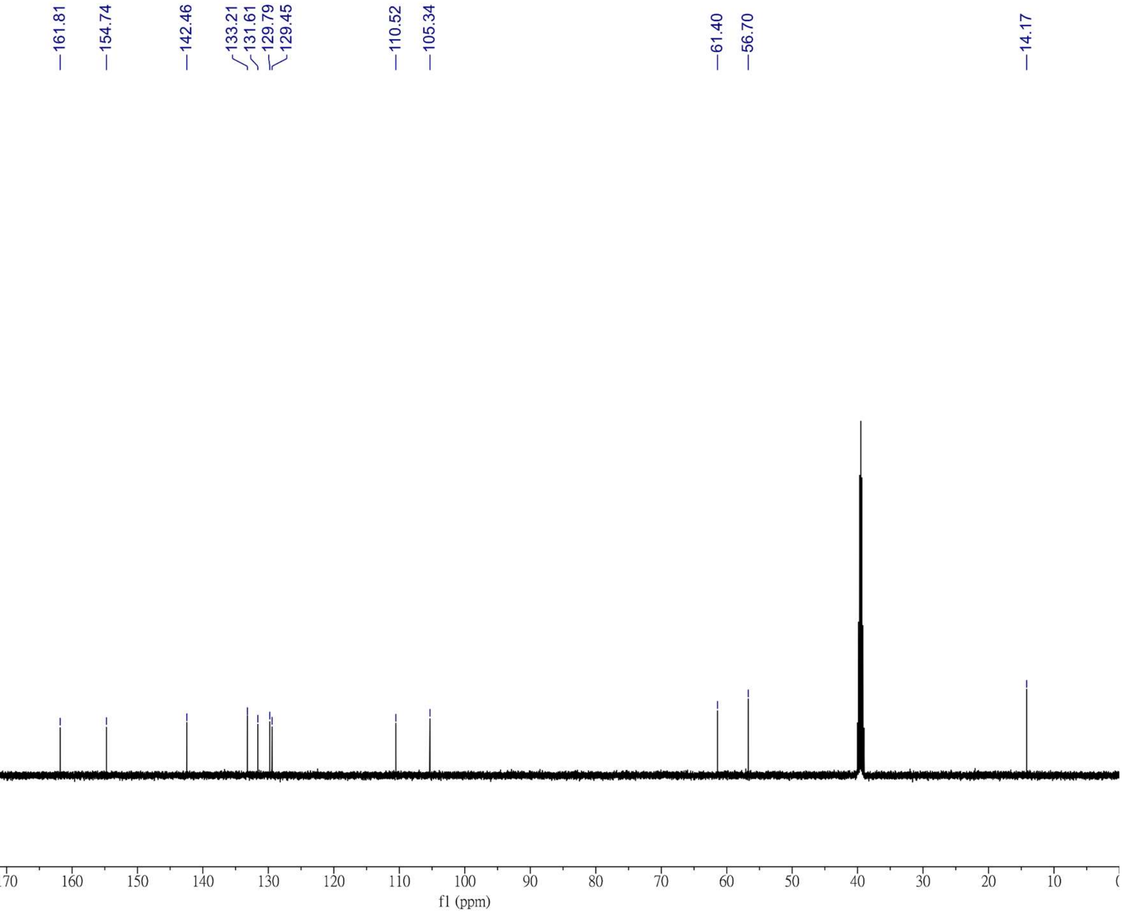

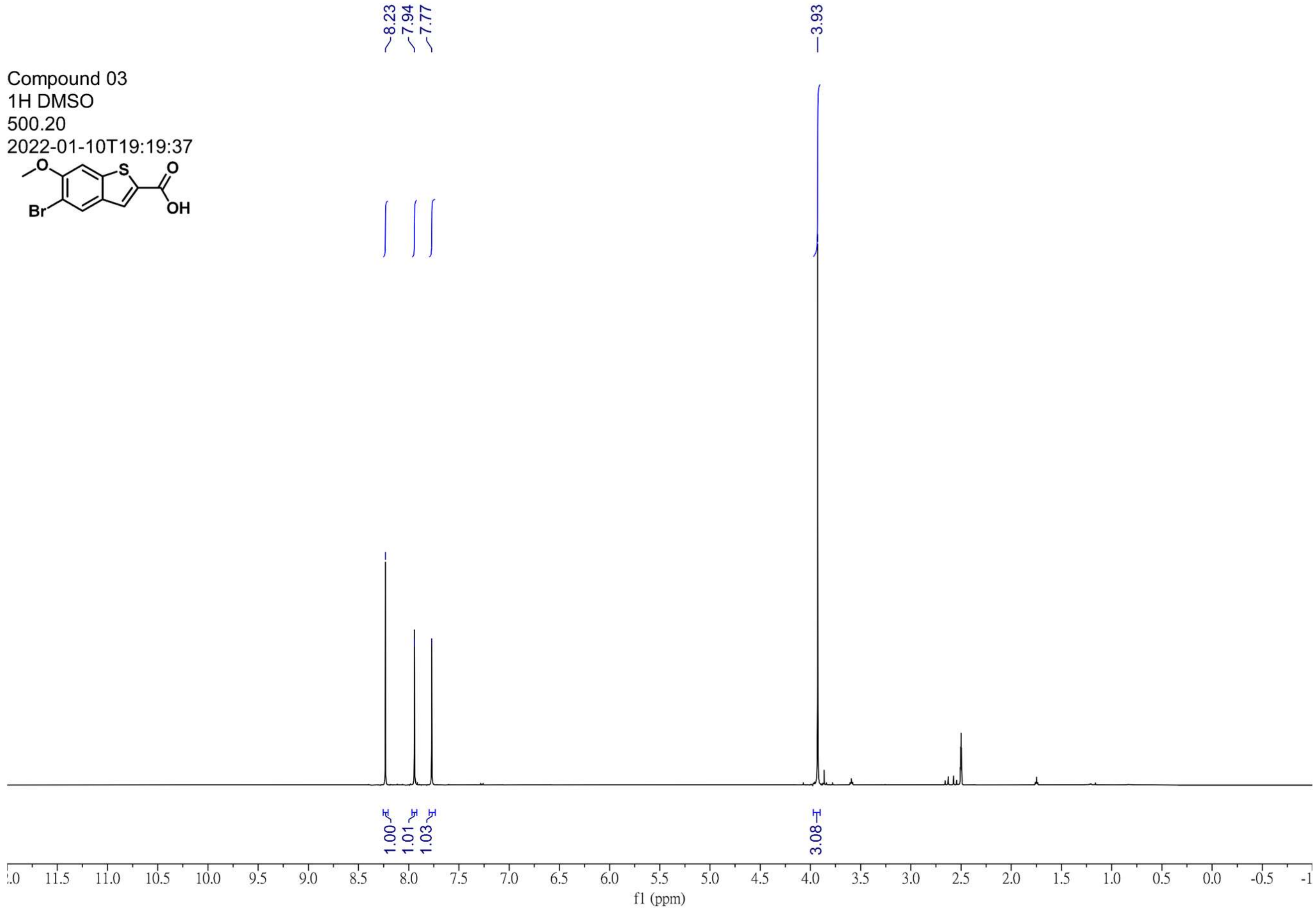
Compound 03
1H DMSO
500.20
2022-01-10T19:19:37
8.23
7.94
7.77
3.93
1.00
1.01
1.03
3.08
f1 (ppm)

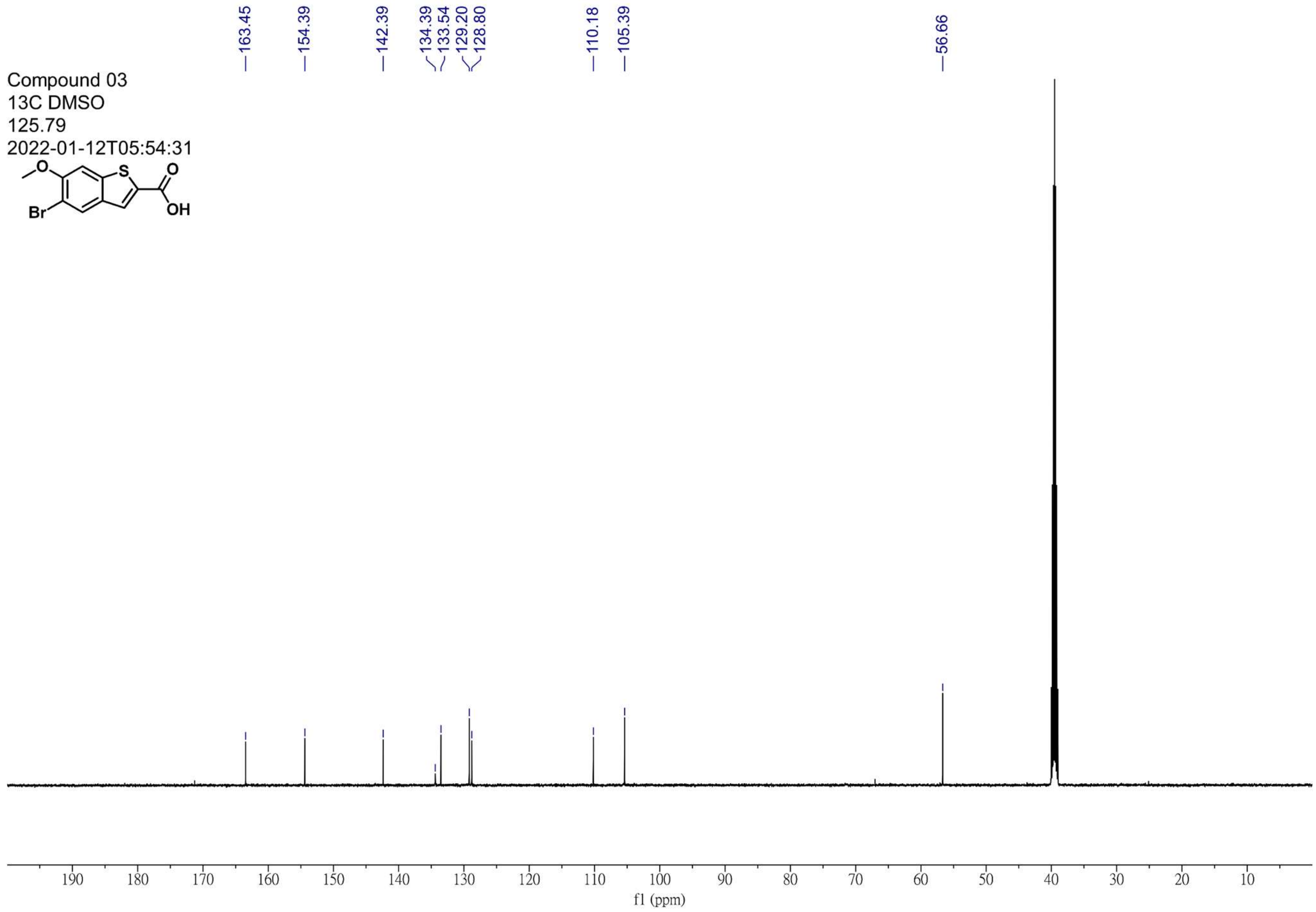
Compound 03
13C DMSO
125.79
2022-01-12T05:54:31
163.45
154.39
142.39
134.39
133.54
129.20
128.80
110.18
105.39
56.66
f1 (ppm)

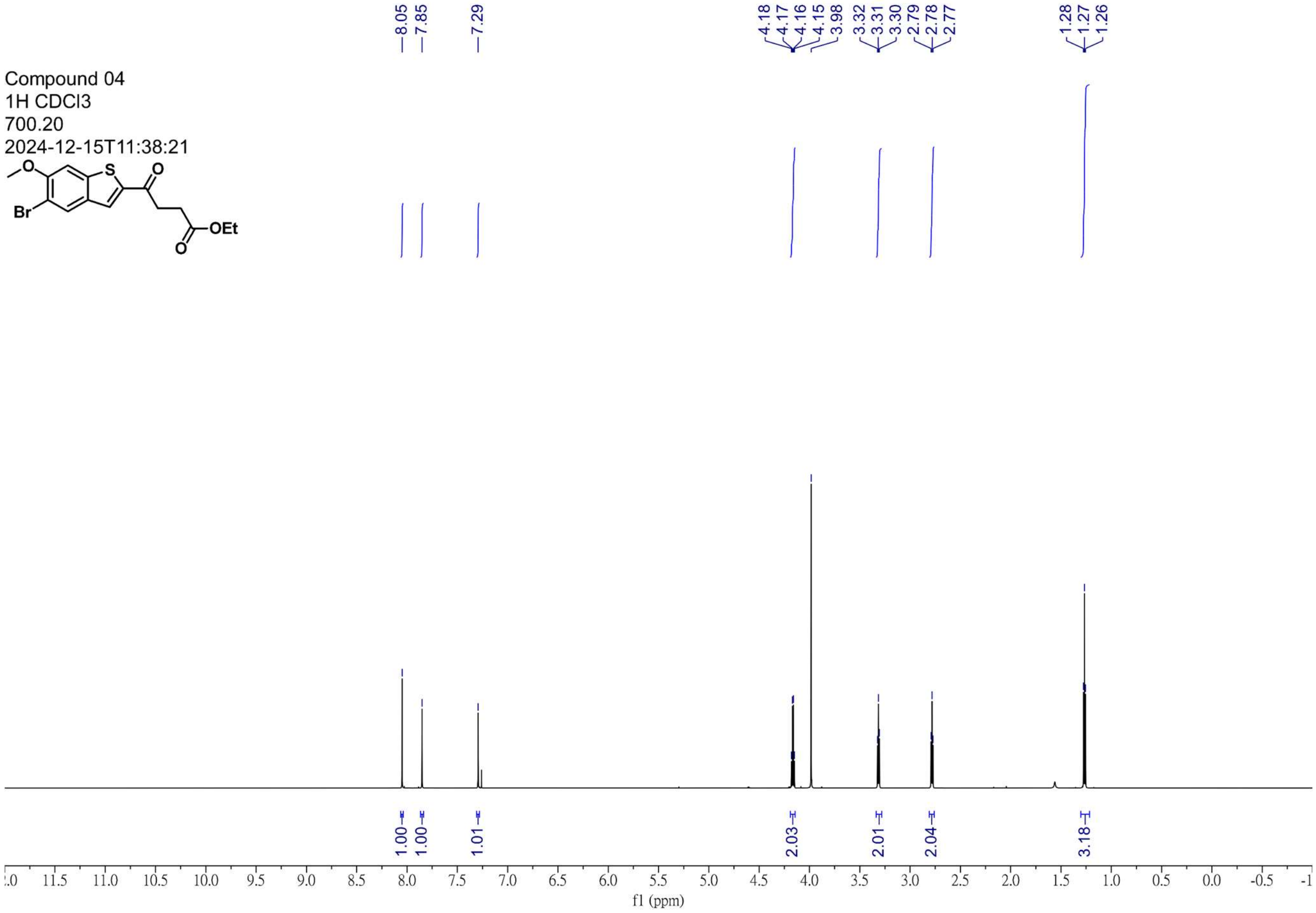
Compound 04
1H CDCl3
700.20
2024-12-15T11:38:21
Br
S
O
OEt
8.05
7.85
7.29
4.18
4.17
4.16
4.15
3.98
3.32
3.31
3.30
2.79
2.78
2.77
1.28
1.27
1.26
1.00
1.00
1.01
2.03
2.01
2.04
3.18
f1 (ppm)

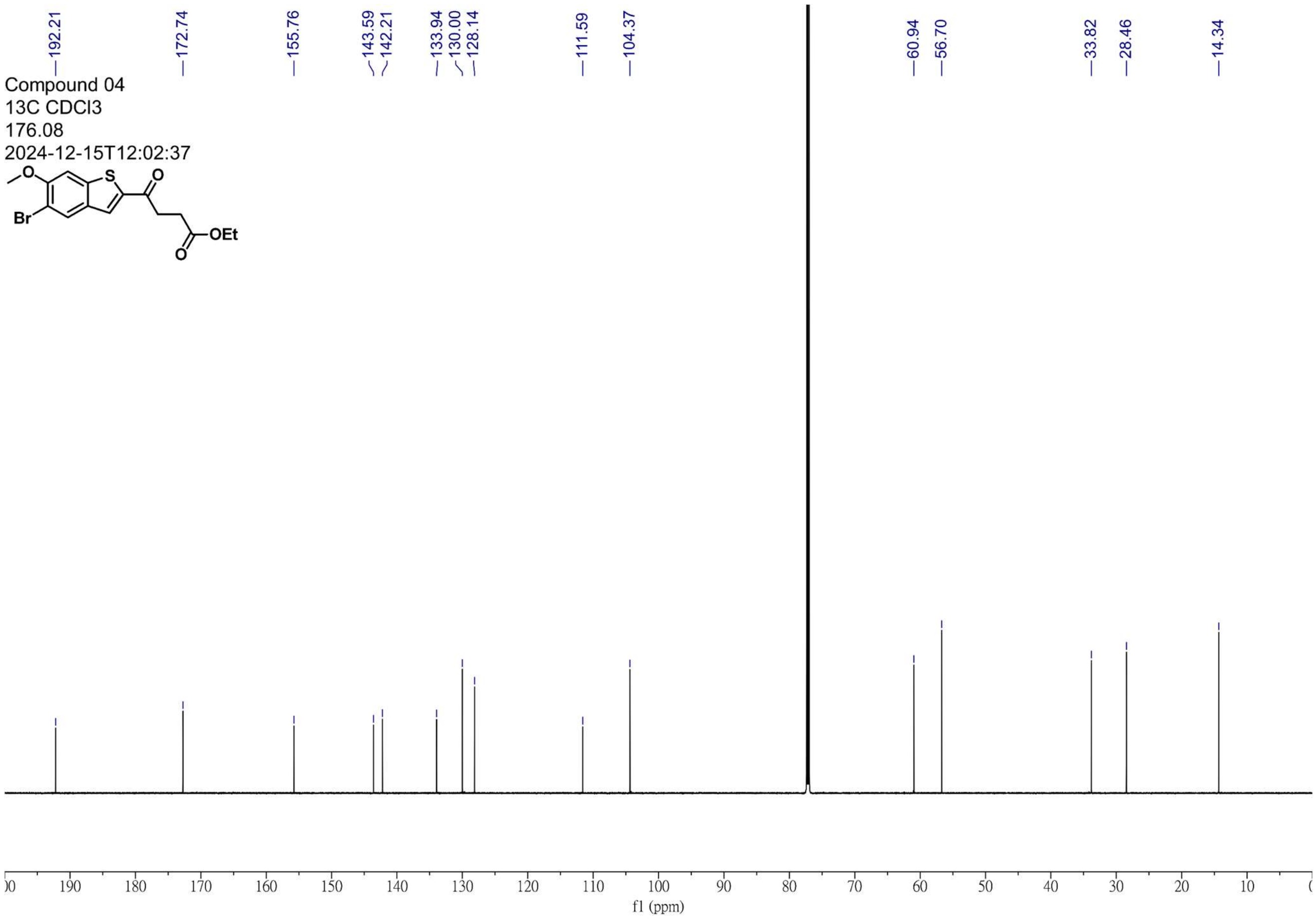
Compound 04
13C CDCl3
176.08
2024-12-15T12:02:37
Br
S
O
OEt
192.21
172.74
155.76
143.59
142.21
133.94
130.00
128.14
111.59
104.37
60.94
56.70
33.82
28.46
14.34
f1 (ppm)

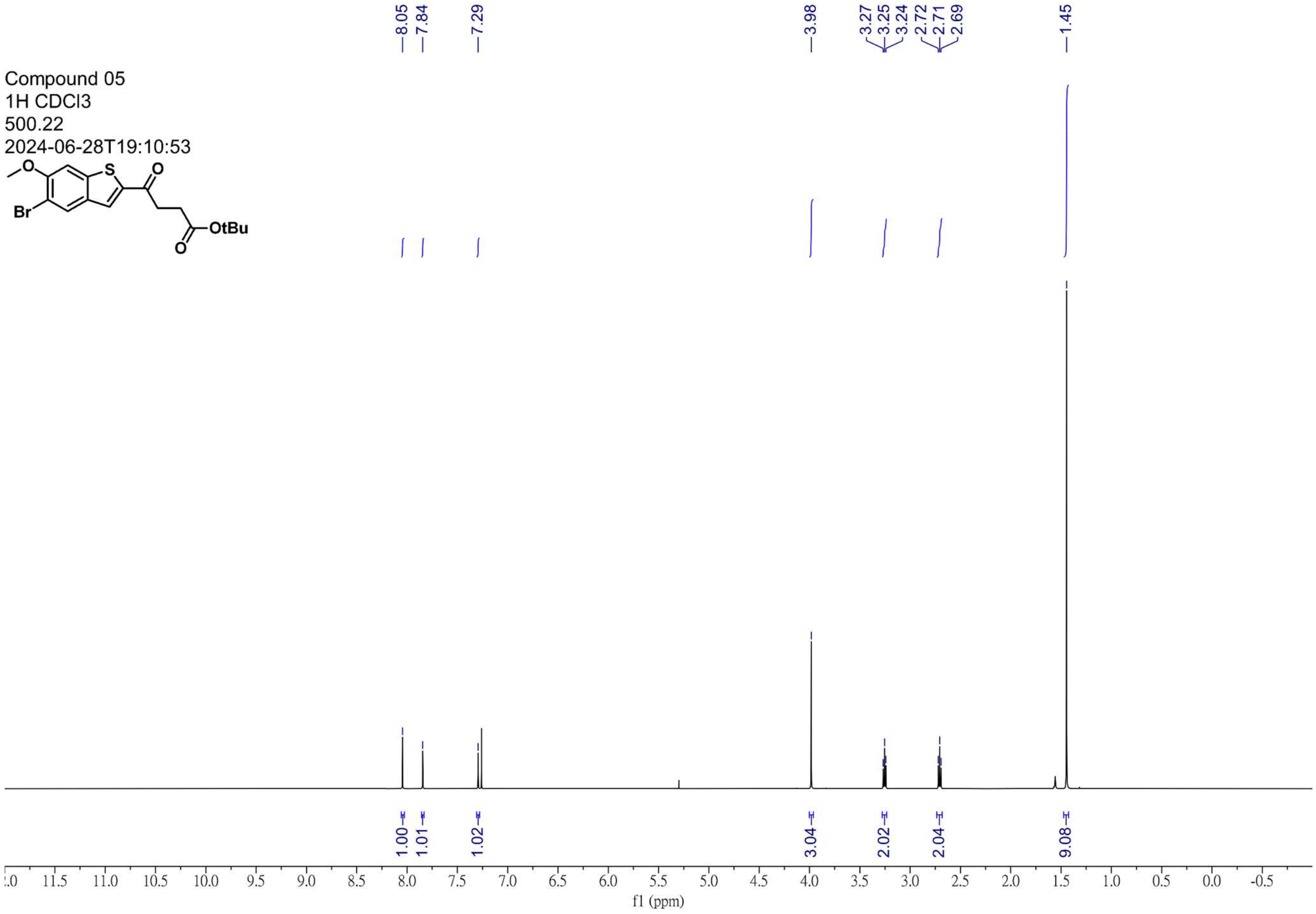
Compound 05
1H CDCl3
500.22
2024-06-28T19:10:53
Br
S
O
OtBu
8.05
7.84
7.29
3.98
3.27
3.25
3.24
2.72
2.71
2.69
1.45
1.00
1.01
1.02
3.04
2.02
2.04
9.08
f1 (ppm)

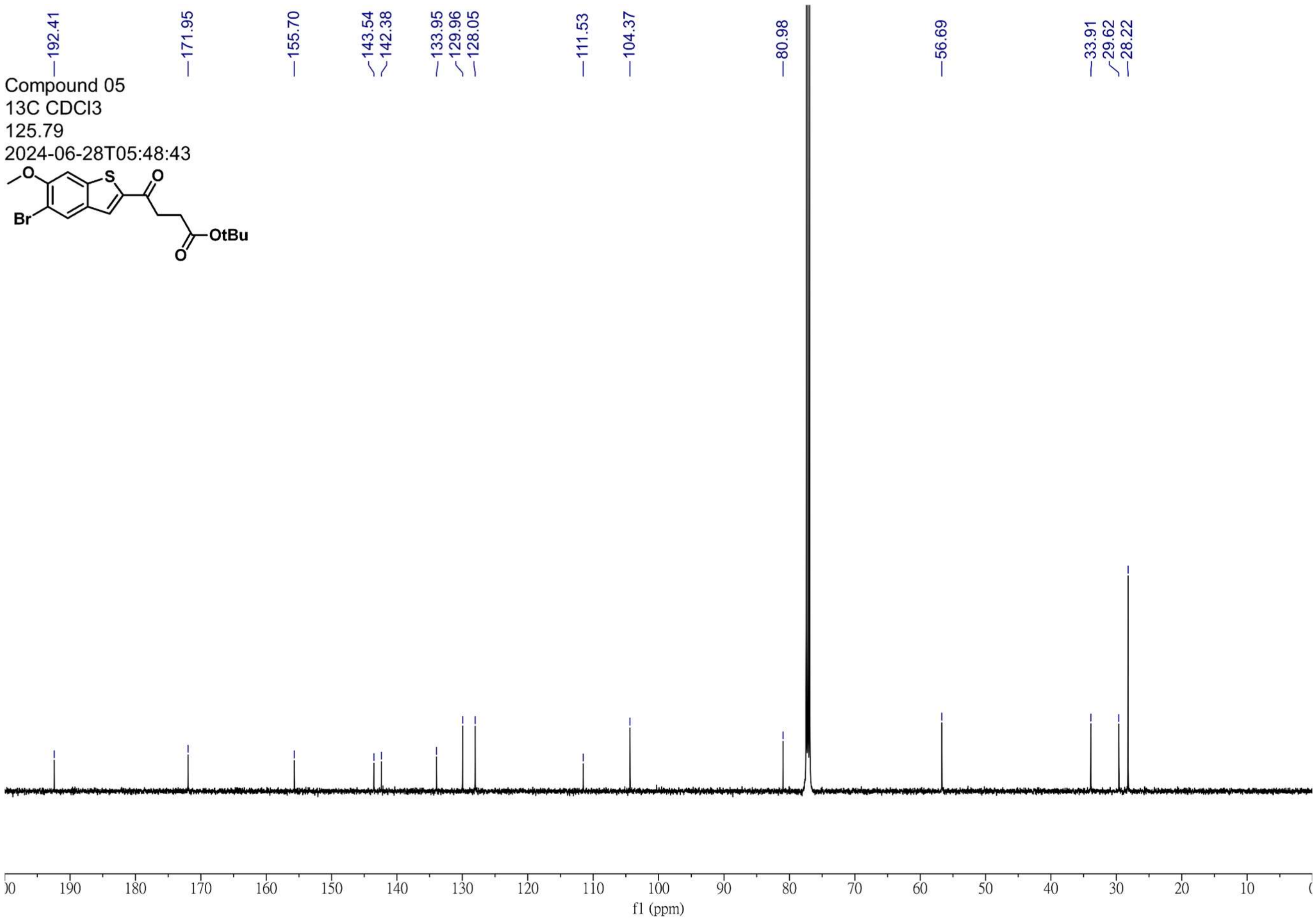
Compound 05
13C CDCl3
125.79
2024-06-28T05:48:43
Br
S
O
OtBu
192.41
171.95
155.70
143.54
142.38
133.95
129.96
128.05
111.53
104.37
80.98
56.69
33.91
29.62
28.22
f1 (ppm)

Compound 06
1H CDCl3
500.22
2023-01-27T16:16:51

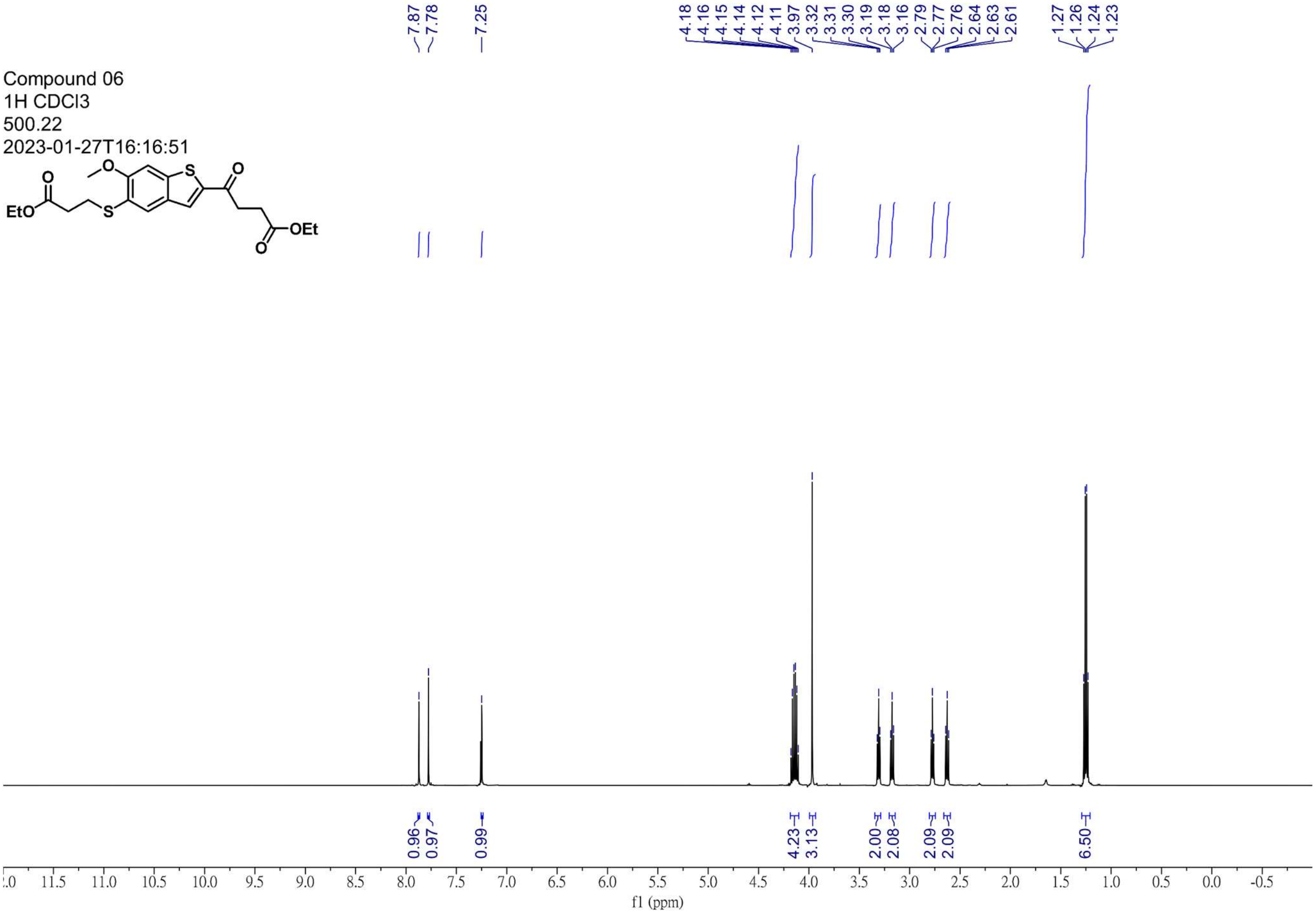

Compound 06
13C CDCl3
125.79
2023-01-27T16:26:05

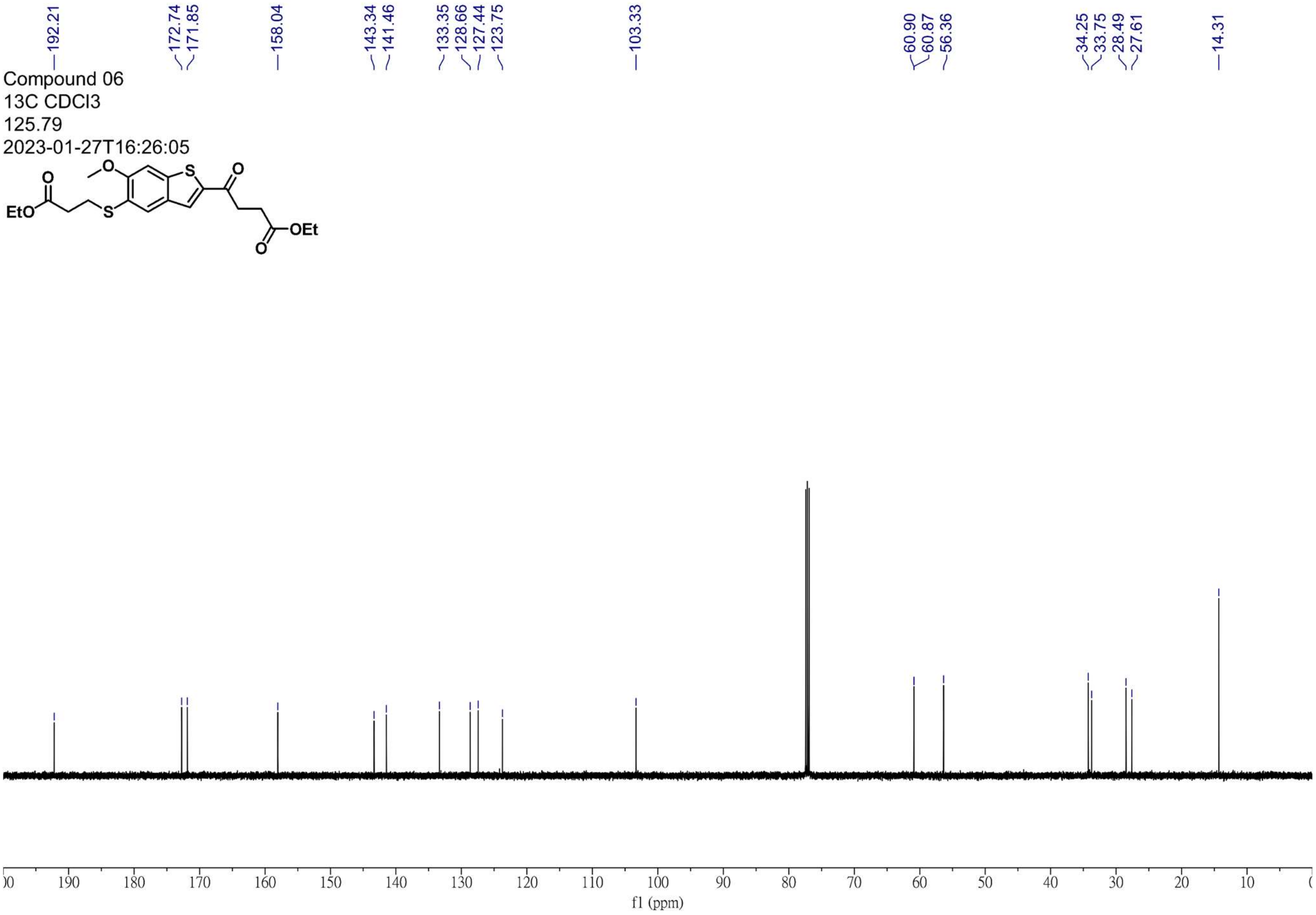

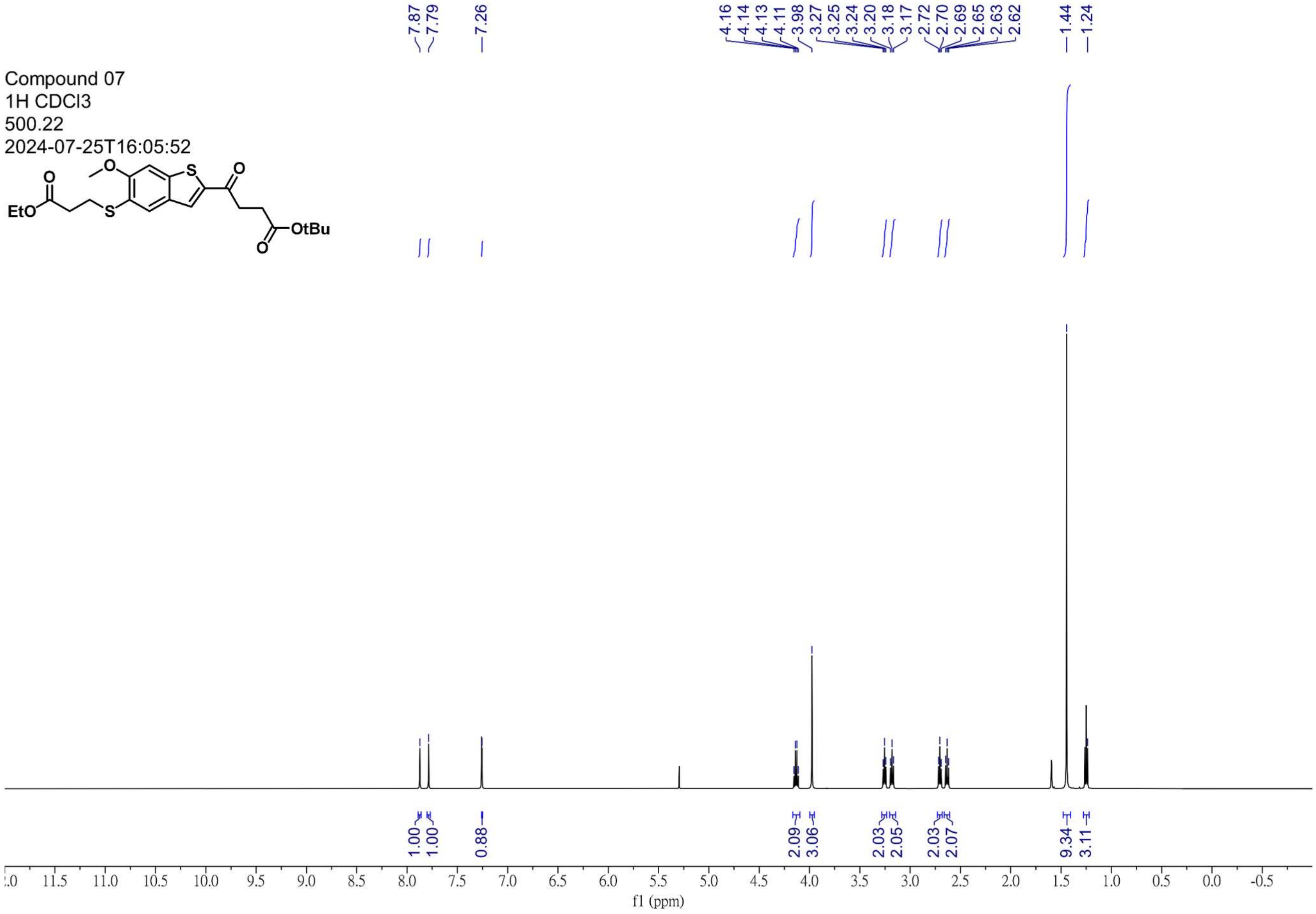

Compound 07
1H CDCl3
500.22
2024-07-25T16:05:52
7.87
7.79
7.26
4.16
4.14
4.13
4.11
3.98
3.27
3.25
3.24
3.20
3.18
3.17
2.72
2.70
2.69
2.65
2.63
2.62
1.44
1.24
1.00
1.00
0.88
2.09
3.06
2.03
2.05
2.03
2.07
9.34
3.11
f1 (ppm)

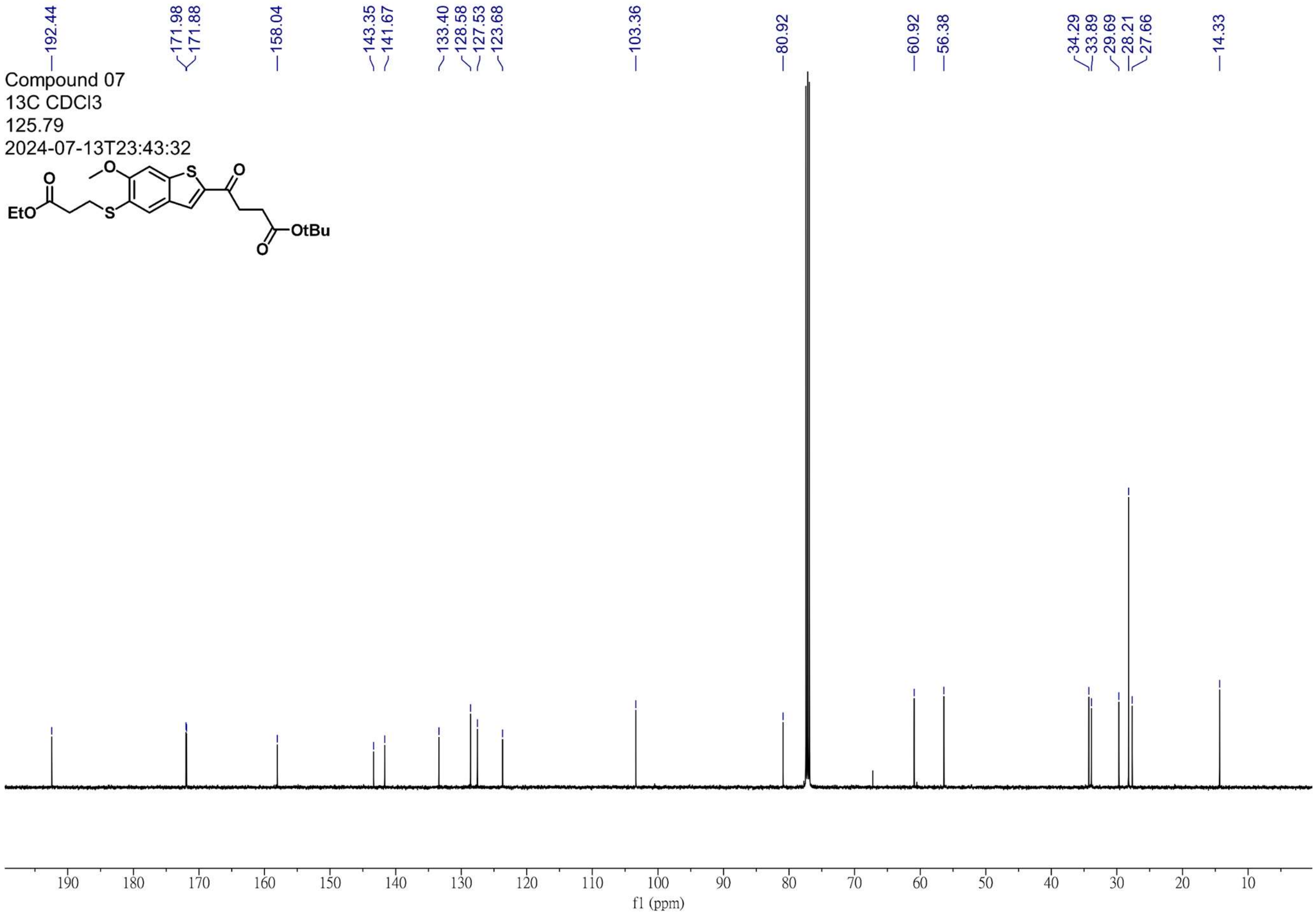

Compound 07
13C CDCl3
125.79
2024-07-13T23:43:32
192.44
171.98
171.88
158.04
143.35
141.67
133.40
128.58
127.53
123.68
103.36
80.92
60.92
56.38
34.29
33.89
29.69
28.21
27.66
14.33
f1 (ppm)

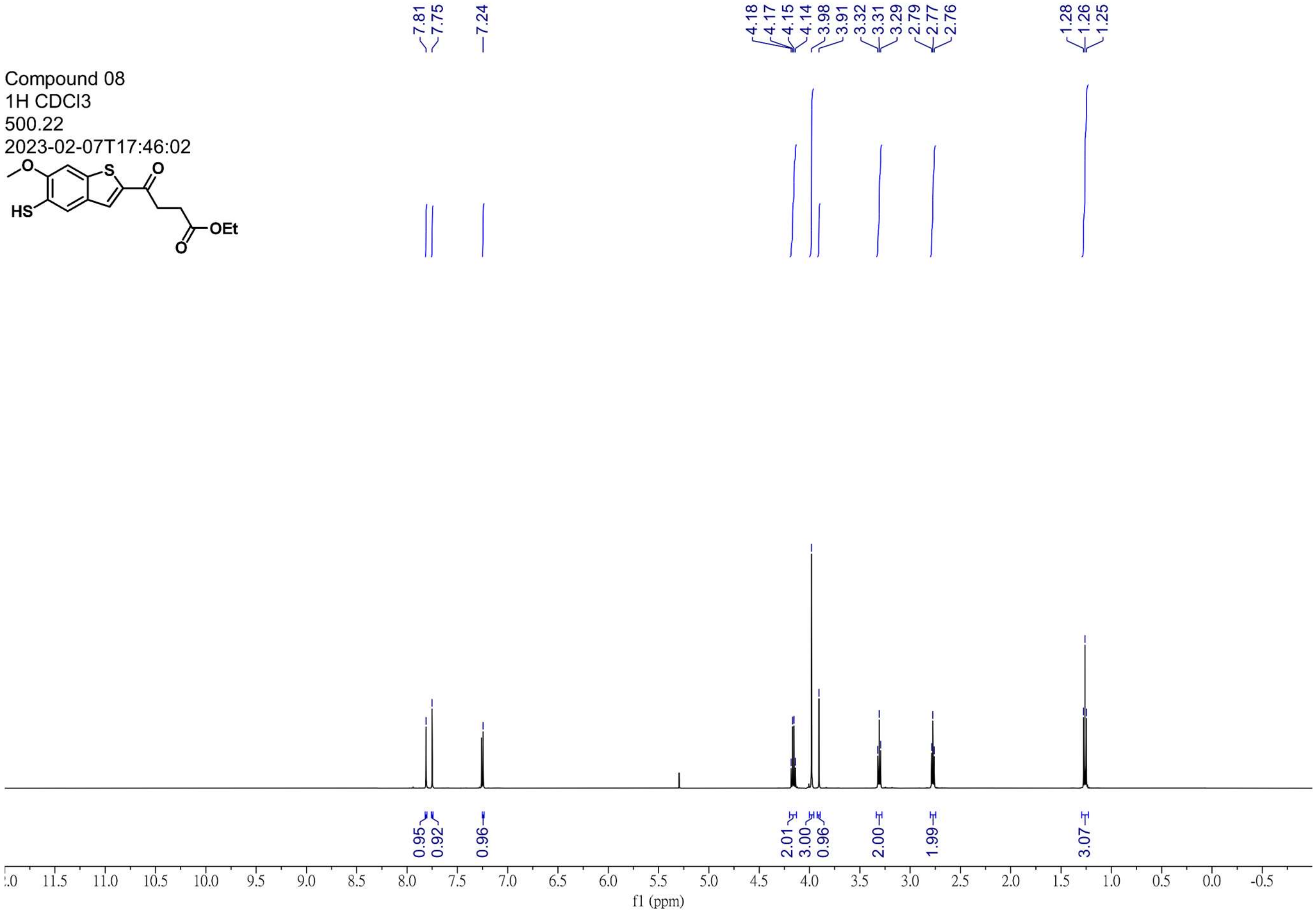
Compound 08
1H CDCl3
500.22
2023-02-07T17:46:02
HS
S
O
OEt
7.81
7.75
7.24
4.18
4.17
4.15
4.14
3.98
3.91
3.32
3.31
3.29
2.79
2.77
2.76
1.28
1.26
1.25
0.95
0.92
0.96
2.01
3.00
0.96
2.00
1.99
3.07
f1 (ppm)

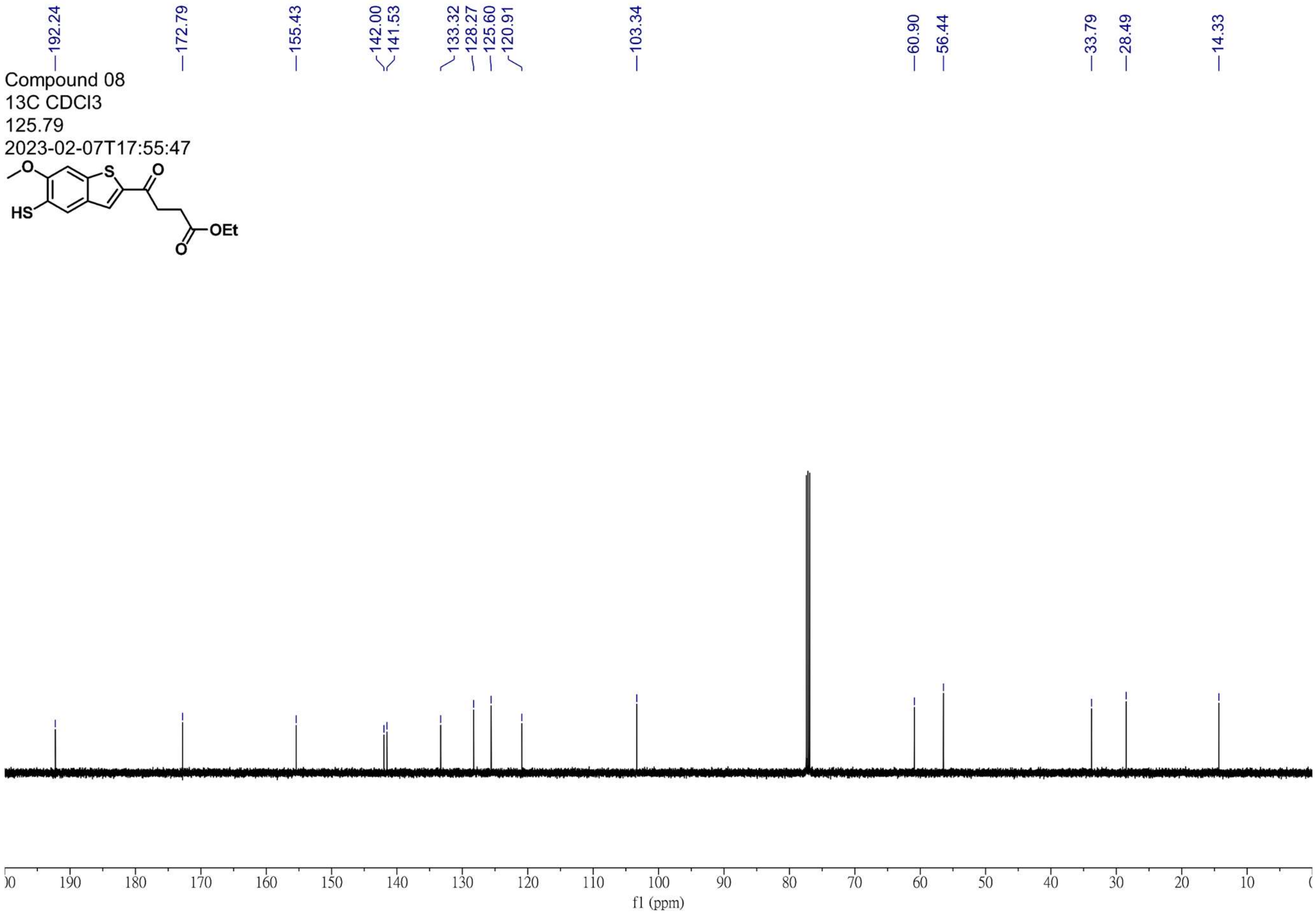
192.24
172.79
155.43
142.00
141.53
133.32
128.27
125.60
120.91
103.34
60.90
56.44
33.79
28.49
14.33
Compound 08
13C CDCl3
125.79
2023-02-07T17:55:47
HS
S
O
OEt
f1 (ppm)

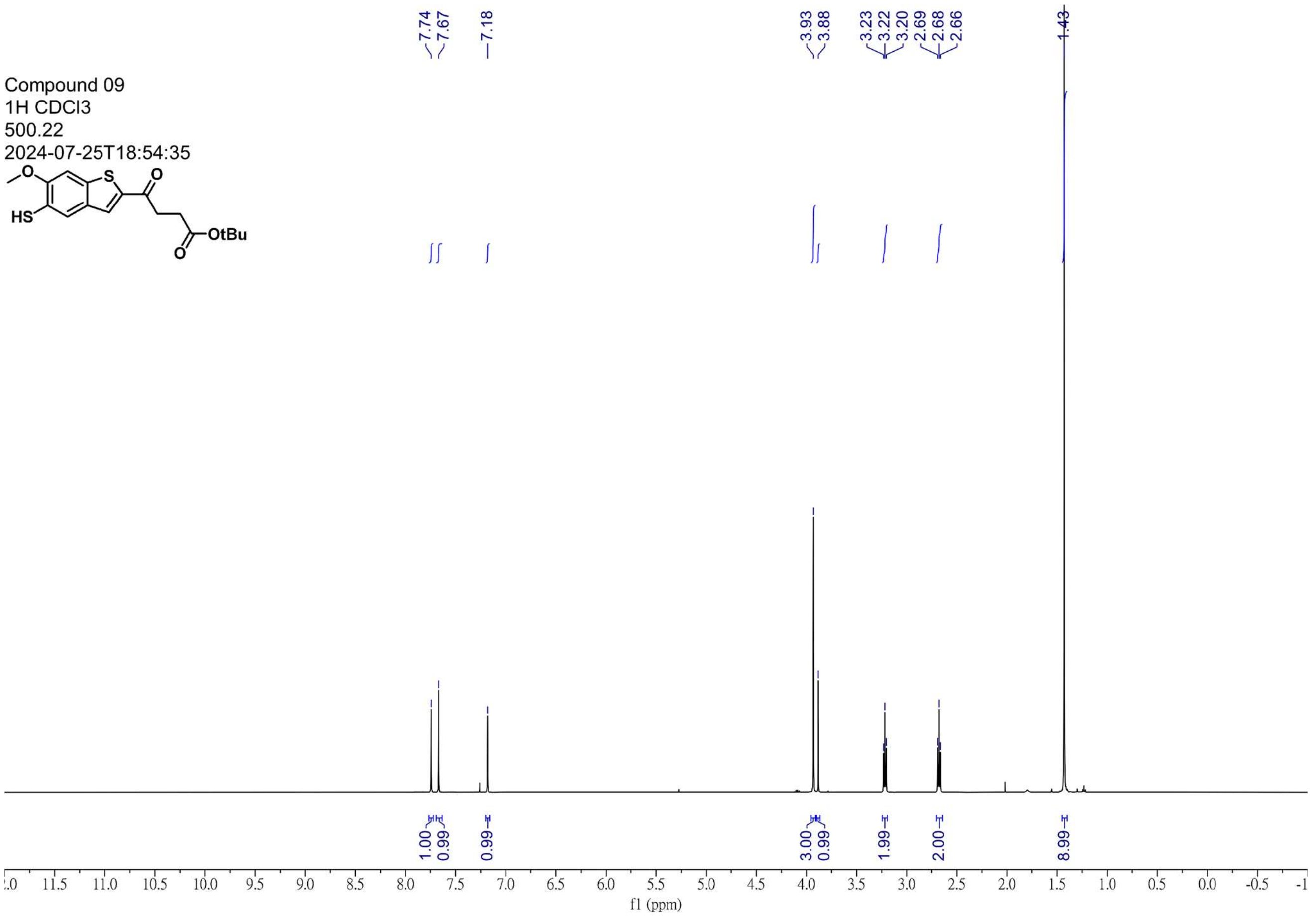
Compound 09
1H CDCl3
500.22
2024-07-25T18:54:35
HS
S
O
OtBu
O
7.74
7.67
7.18
3.93
3.88
3.23
3.22
3.20
2.69
2.68
2.66
1.43
1.00
0.99
0.99
3.00
0.99
1.99
2.00
8.99
f1 (ppm)

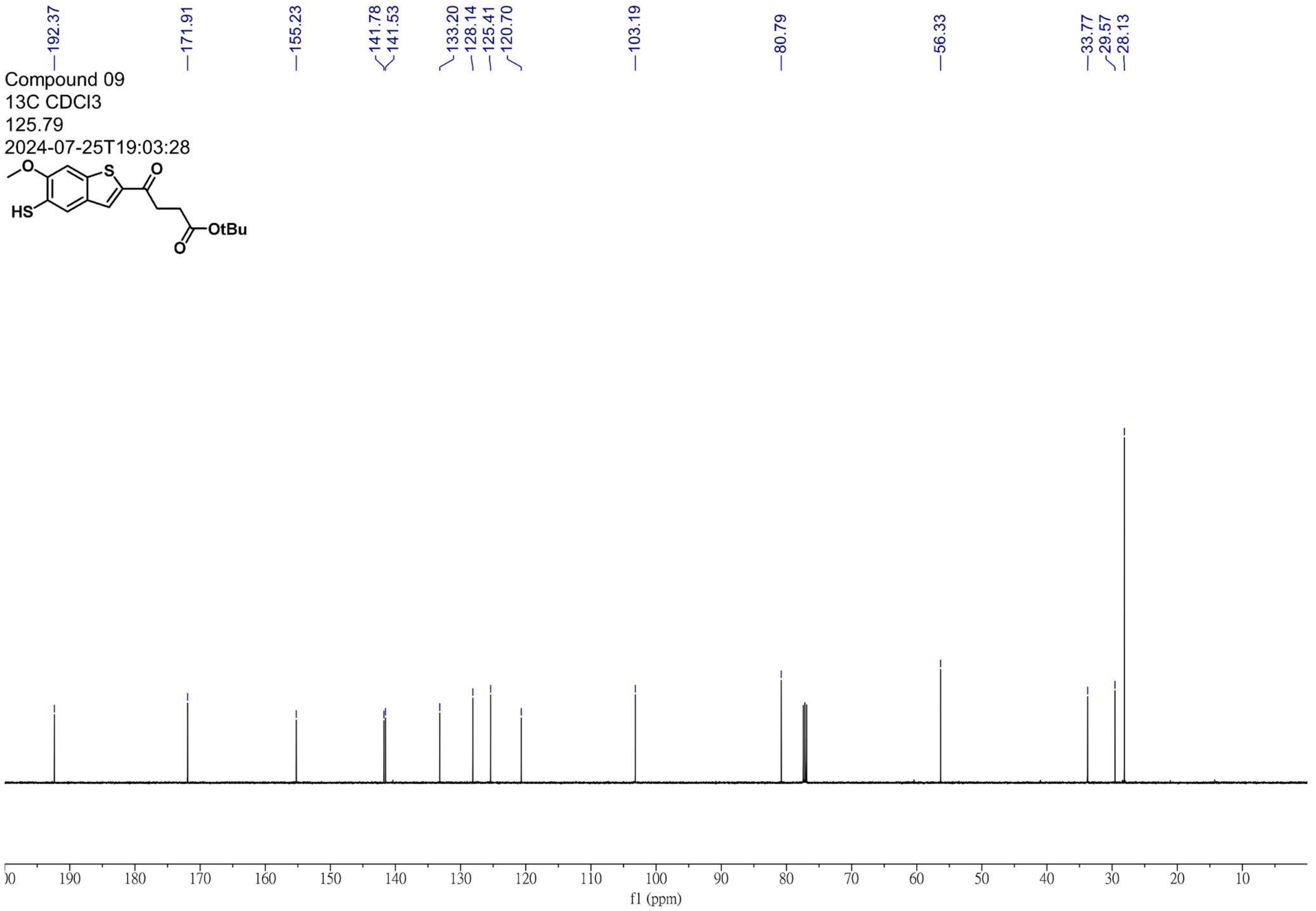
Compound 09
13C CDCl3
125.79
2024-07-25T19:03:28
HS
S
O
OtBu
O
192.37
171.91
155.23
141.78
141.53
133.20
128.14
125.41
120.70
103.19
80.79
56.33
33.77
29.57
28.13
f1 (ppm)

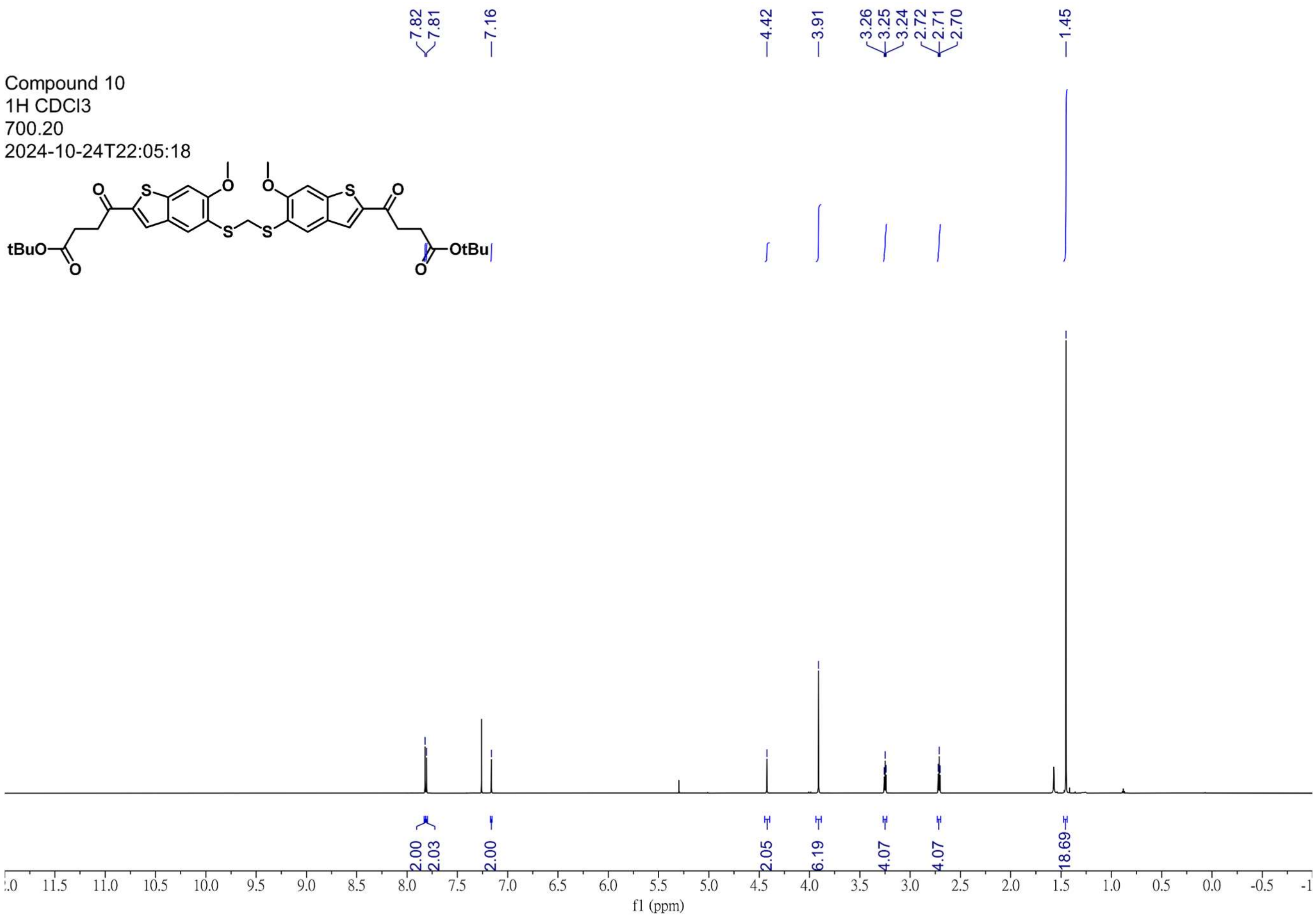
Compound 10
1H CDCl3
700.20
2024-10-24T22:05:18
tBuO
OtBu
7.82
7.81
7.16
4.42
3.91
3.26
3.25
3.24
2.72
2.71
2.70
1.45
2.00
2.03
2.00
2.05
6.19
4.07
4.07
18.69
f1 (ppm)

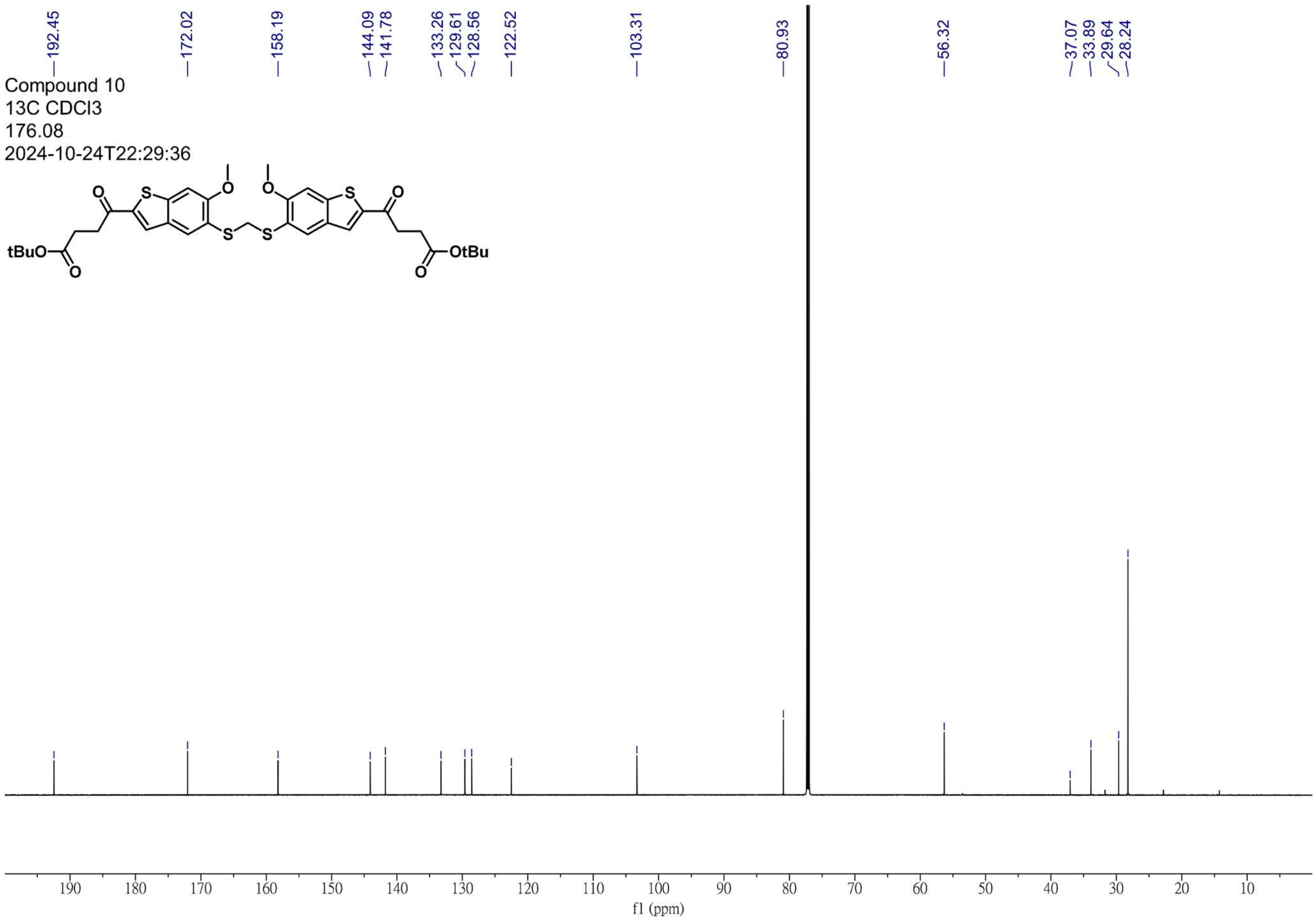
Compound 10
13C CDCl3
176.08
2024-10-24T22:29:36
tBuO
OtBu
192.45
172.02
158.19
144.09
141.78
133.26
129.61
128.56
122.52
103.31
80.93
56.32
37.07
33.89
29.64
28.24
f1 (ppm)

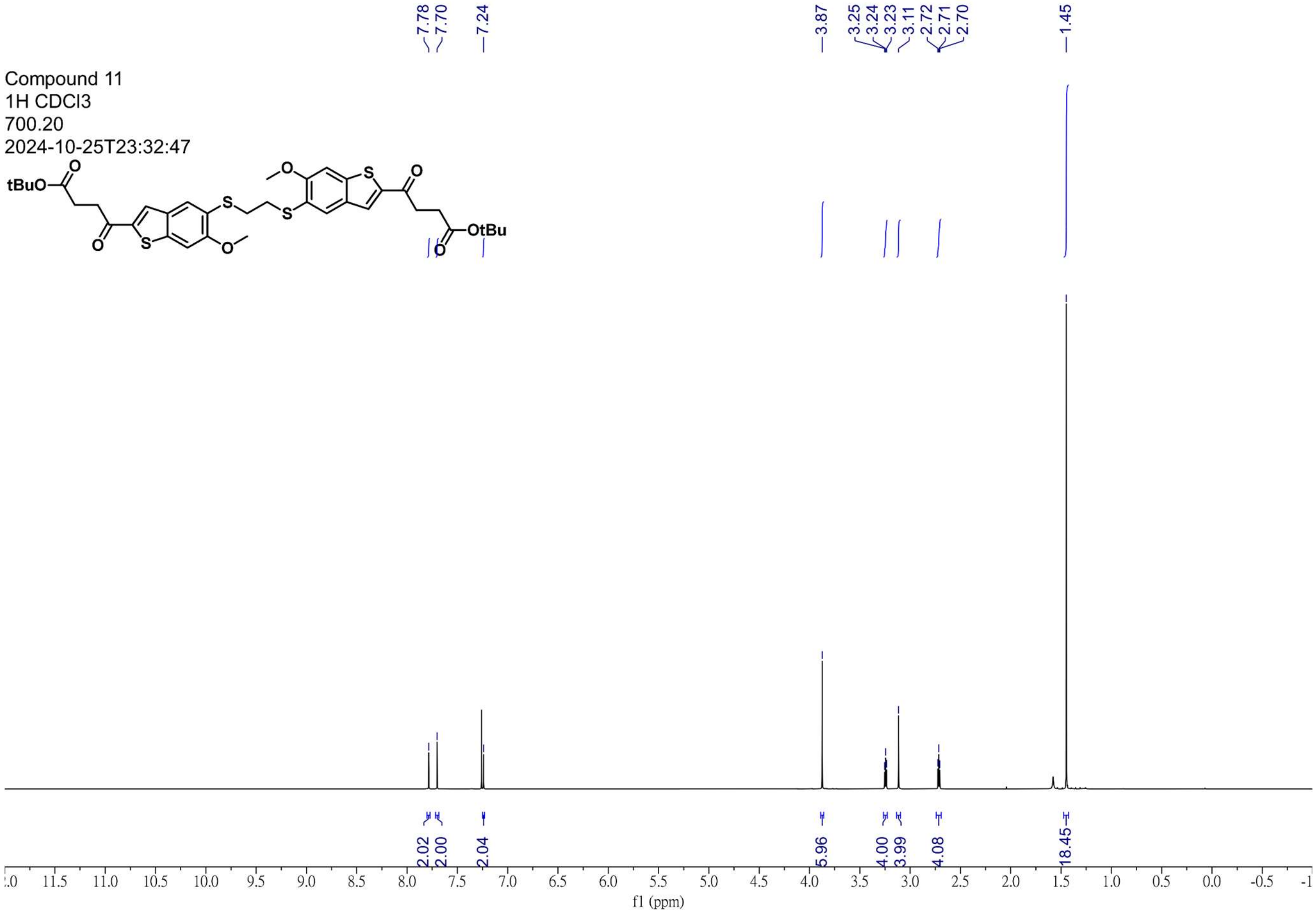
Compound 11
1H CDCl3
700.20
2024-10-25T23:32:47
tBuO
S
S
S
S
O
O
O
O
O
O
OtBu
7.78
7.70
7.24
3.87
3.25
3.24
3.23
3.11
2.72
2.71
2.70
1.45
2.02
2.00
2.04
5.96
4.00
3.99
4.08
18.45
f1 (ppm)

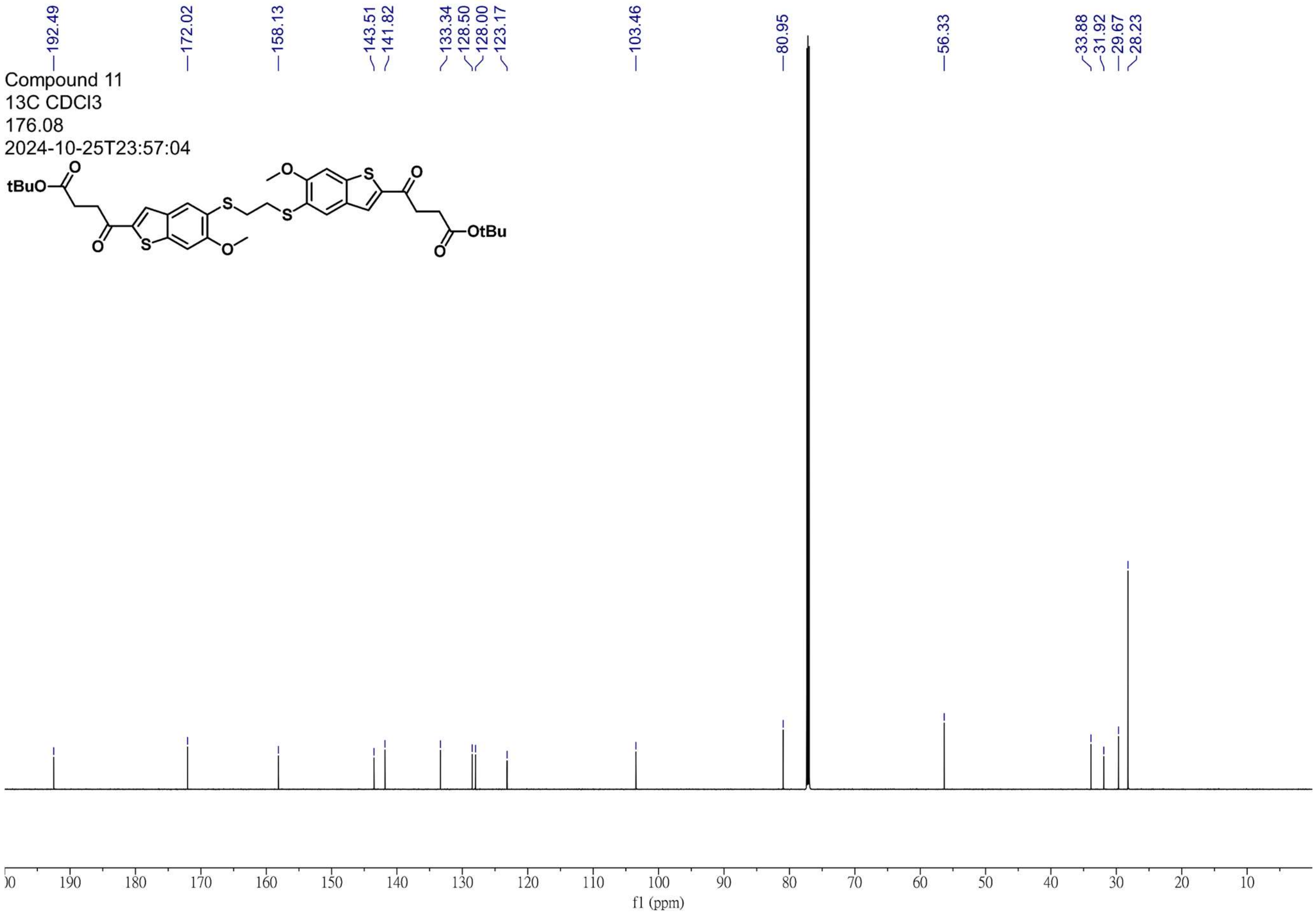
Compound 11
13C CDCl3
176.08
2024-10-25T23:57:04
tBuO
S
S
S
S
O
O
O
O
O
O
OtBu
192.49
172.02
158.13
143.51
141.82
133.34
128.50
128.00
123.17
103.46
80.95
56.33
33.88
31.92
29.67
28.23
f1 (ppm)

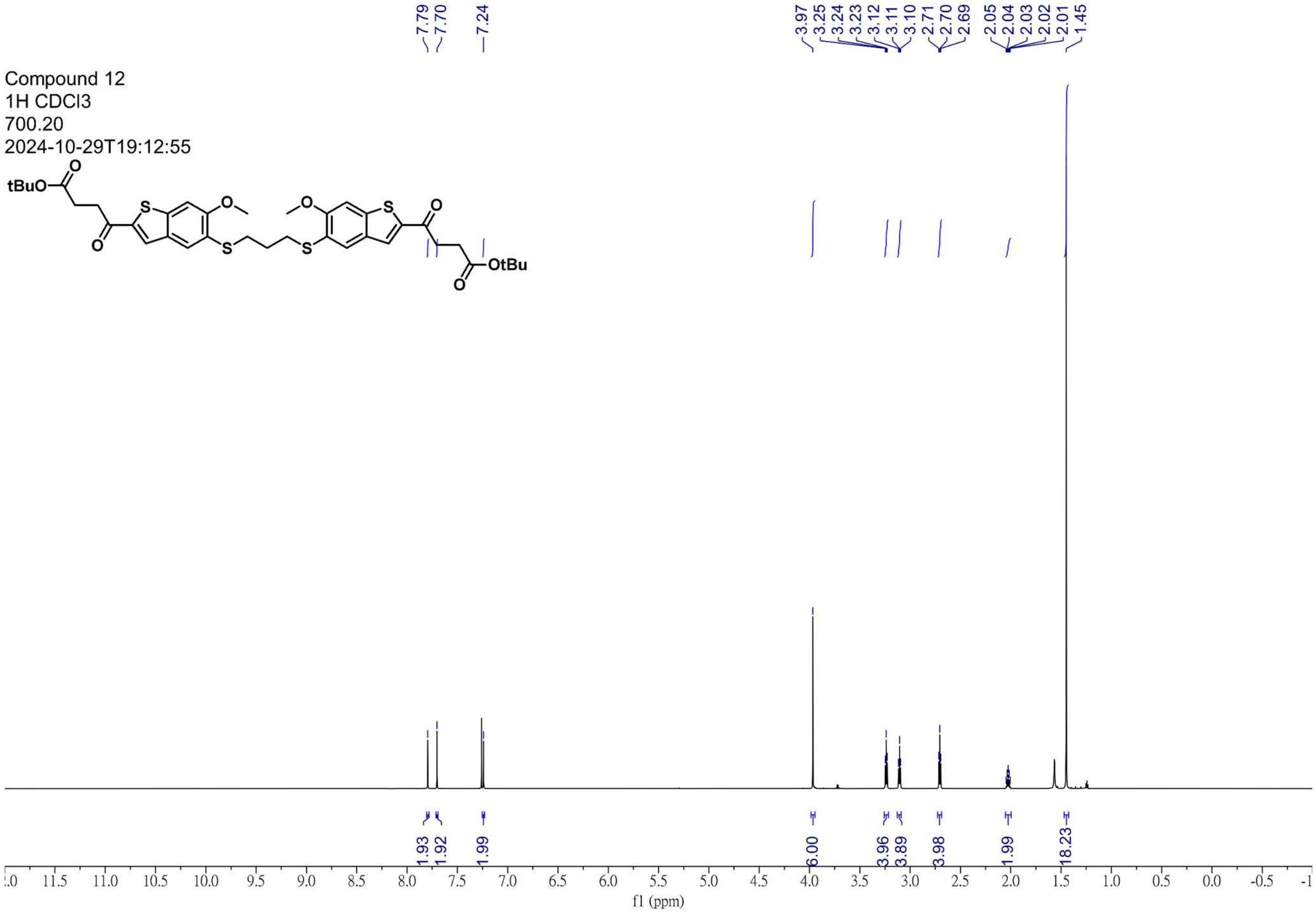
Compound 12
1H CDCl3
700.20
2024-10-29T19:12:55
tBuO
OtBu
7.79
7.70
7.24
3.97
3.25
3.24
3.23
3.12
3.11
3.10
2.71
2.70
2.69
2.05
2.04
2.03
2.02
2.01
1.45
1.93
1.92
1.99
6.00
3.96
3.89
3.98
1.99
18.23
f1 (ppm)

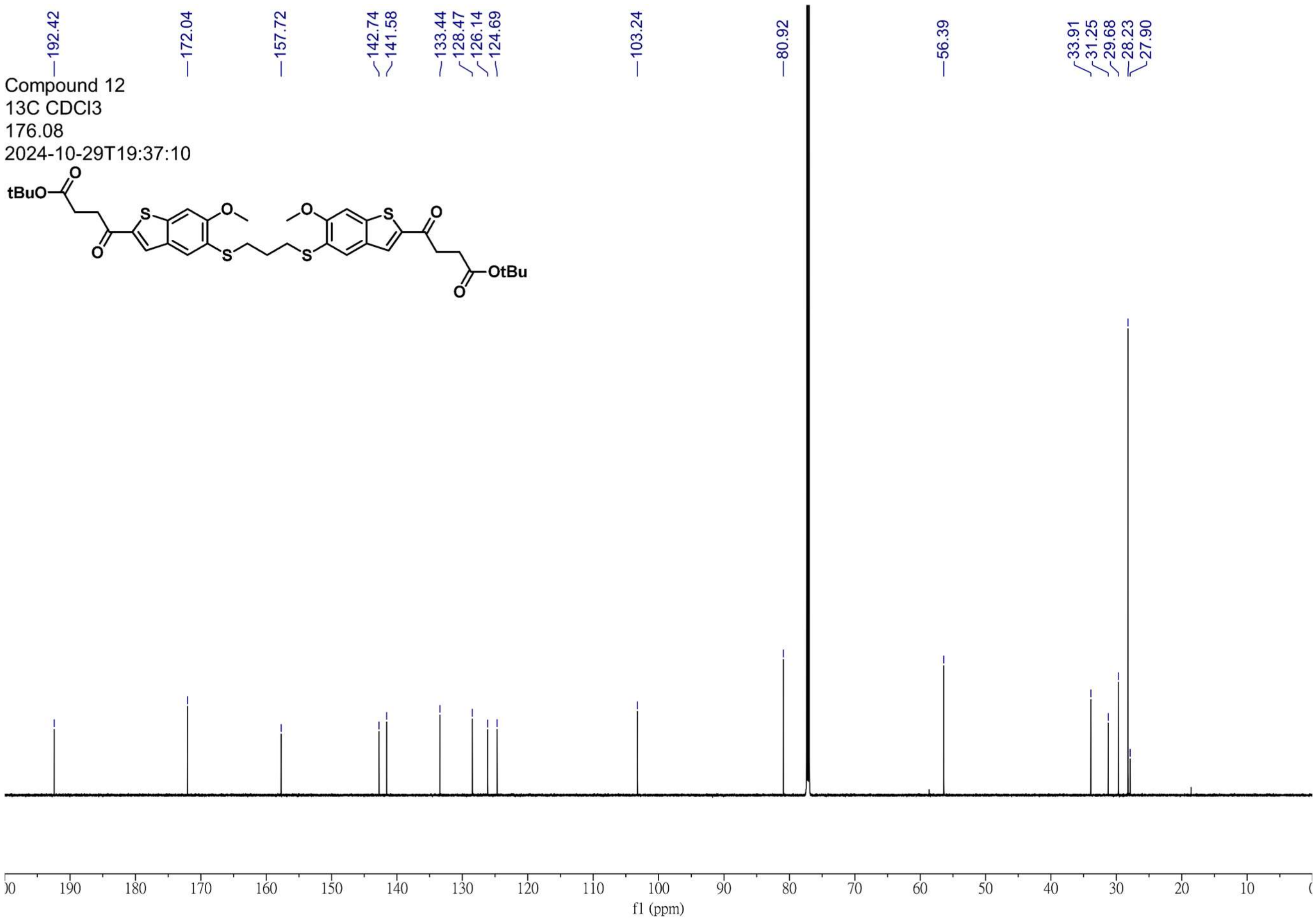
Compound 12
13C CDCl3
176.08
2024-10-29T19:37:10
tBuO
OtBu
192.42
172.04
157.72
142.74
141.58
133.44
128.47
126.14
124.69
103.24
80.92
56.39
33.91
31.25
29.68
28.23
27.90
f1 (ppm)

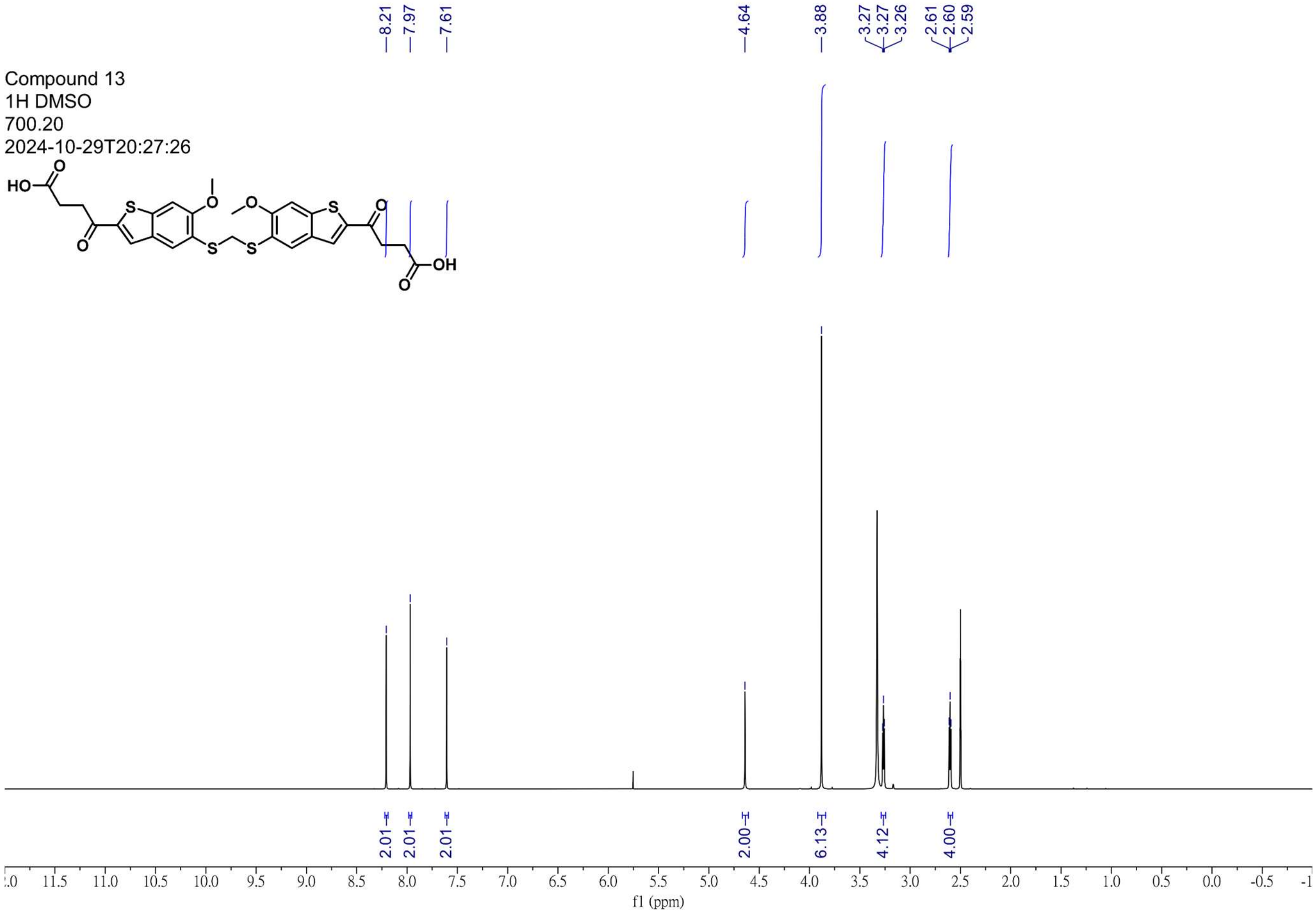
Compound 13
1H DMSO
700.20
2024-10-29T20:27:26
HO
O
S
O
S
S
O
S
O
OH
O
8.21
7.97
7.61
4.64
3.88
3.27
3.27
3.26
2.61
2.60
2.59
2.01
2.01
2.01
2.00
6.13
4.12
4.00
f1 (ppm)

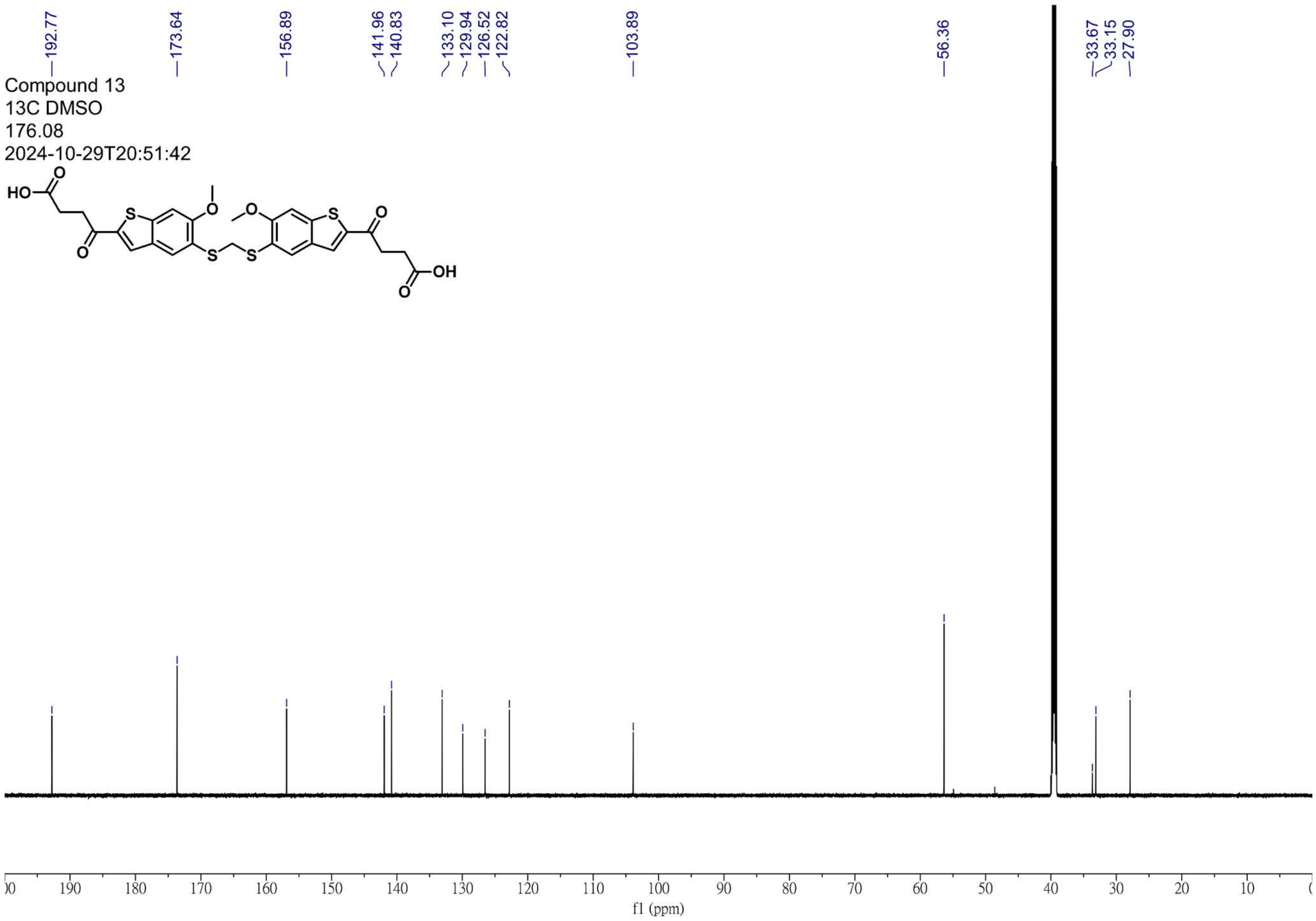
Compound 13
13C DMSO
176.08
2024-10-29T20:51:42
192.77
173.64
156.89
141.96
140.83
133.10
129.94
126.52
122.82
103.89
56.36
33.67
33.15
27.90
f1 (ppm)

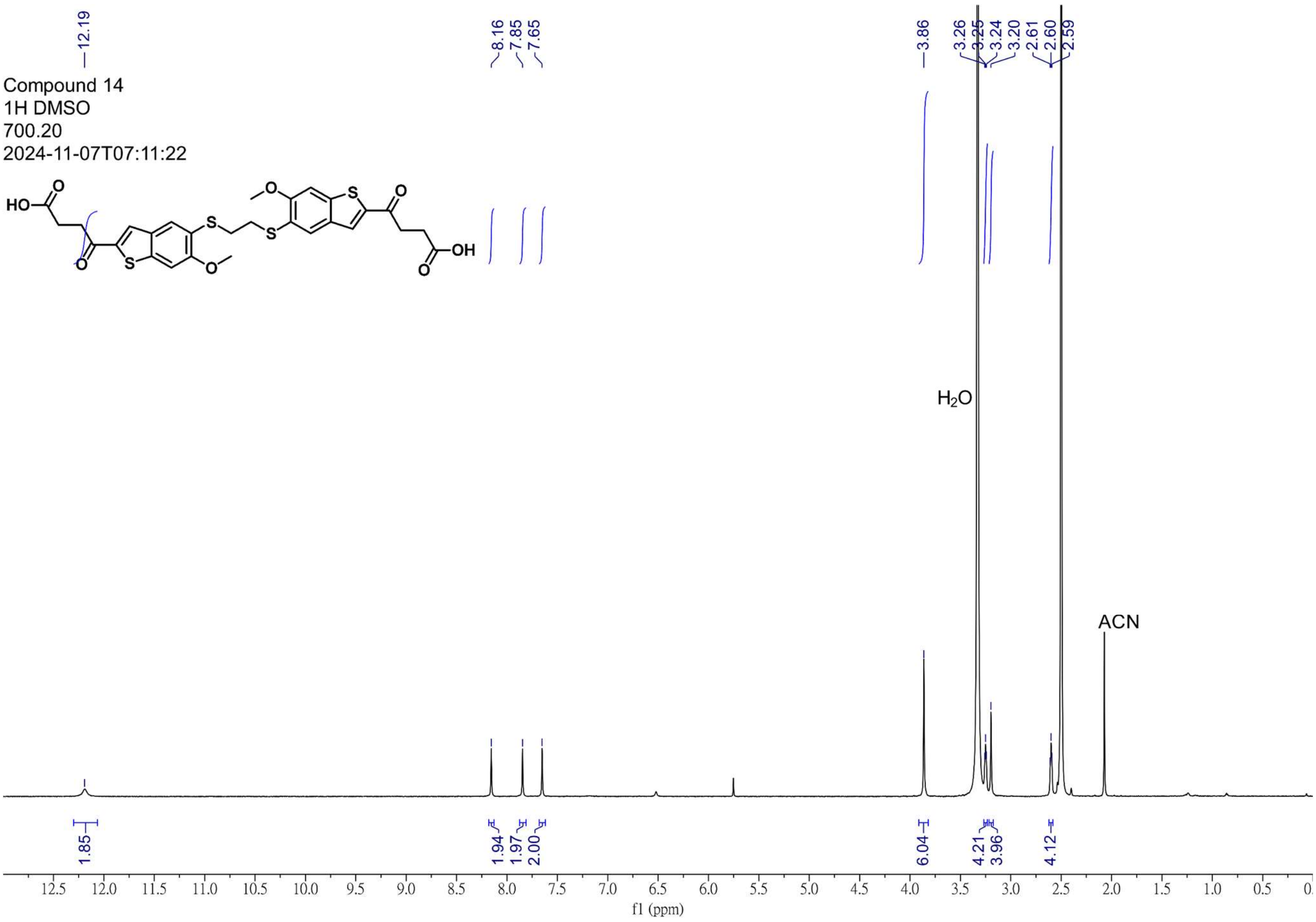

Compound 14
1H DMSO
700.20
2024-11-07T07:11:22
HO
O
O
S
S
O
S
S
O
O
OH
H2O
ACN
12.19
8.16
7.85
7.65
3.86
3.26
3.25
3.24
3.20
2.61
2.60
2.59
1.85
1.94
1.97
2.00
6.04
4.21
3.96
4.12
f1 (ppm)

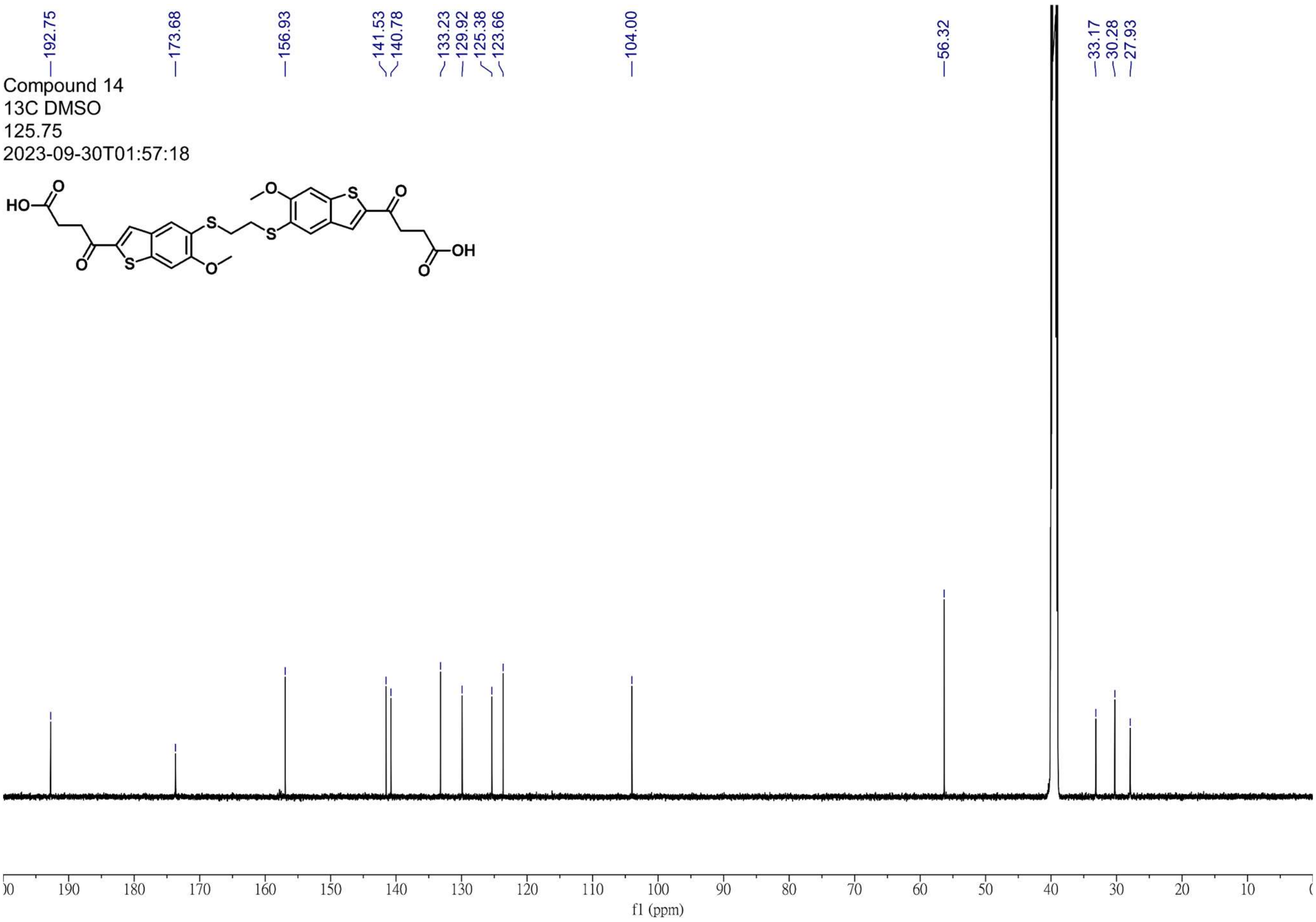

Compound 14
13C DMSO
125.75
2023-09-30T01:57:18
HO
O
O
S
S
O
S
S
O
O
OH
192.75
173.68
156.93
141.53
140.78
133.23
129.92
125.38
123.66
104.00
56.32
33.17
30.28
27.93
f1 (ppm)

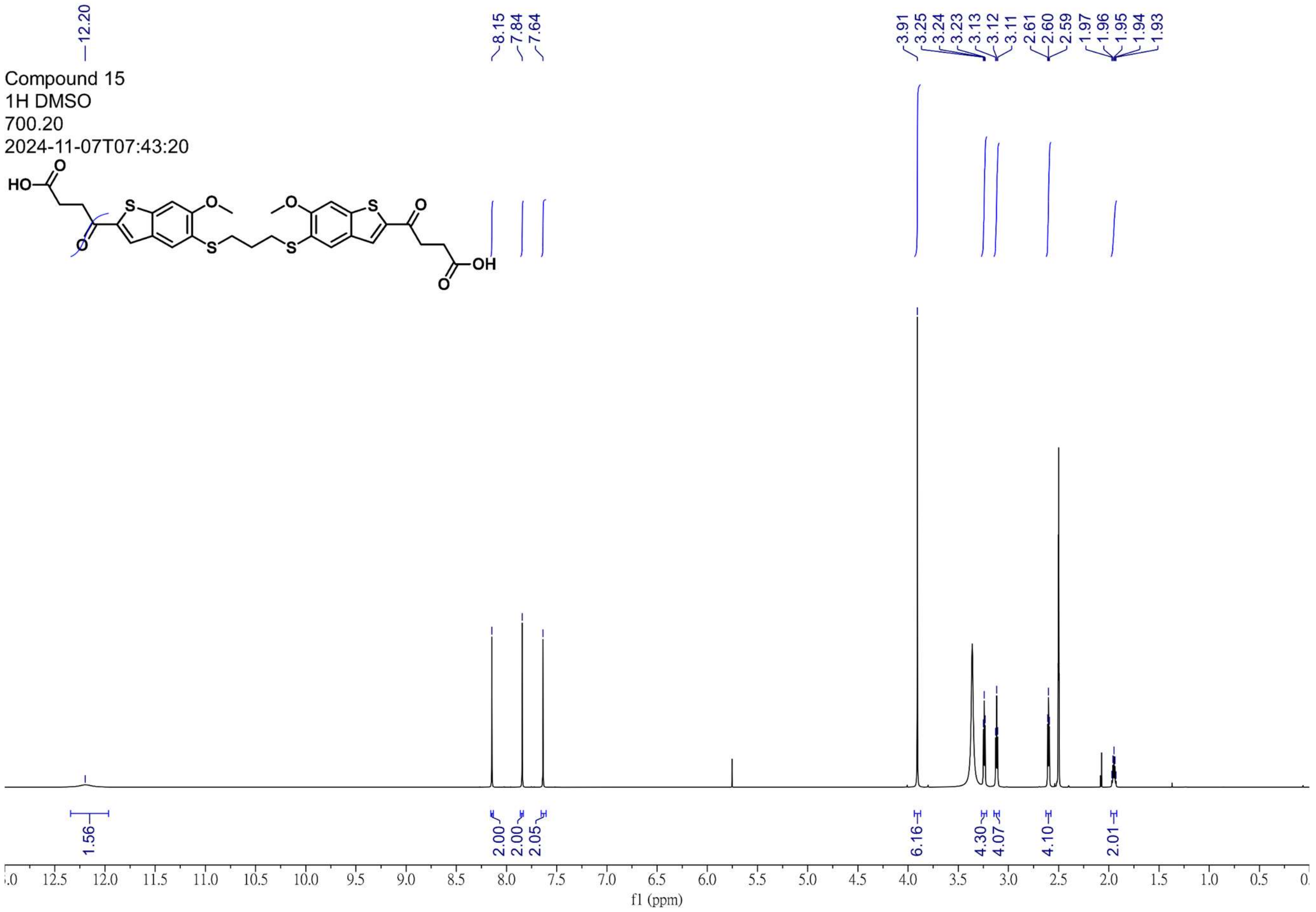
Compound 15
1H DMSO
700.20
2024-11-07T07:43:20
12.20
8.15
7.84
7.64
3.91
3.25
3.24
3.23
3.13
3.12
3.11
2.61
2.60
2.59
1.97
1.96
1.95
1.94
1.93
1.56
2.00
2.00
2.05
6.16
4.30
4.07
4.10
2.01
HO
O
S
O
S
S
O
S
O
OH
f1 (ppm)

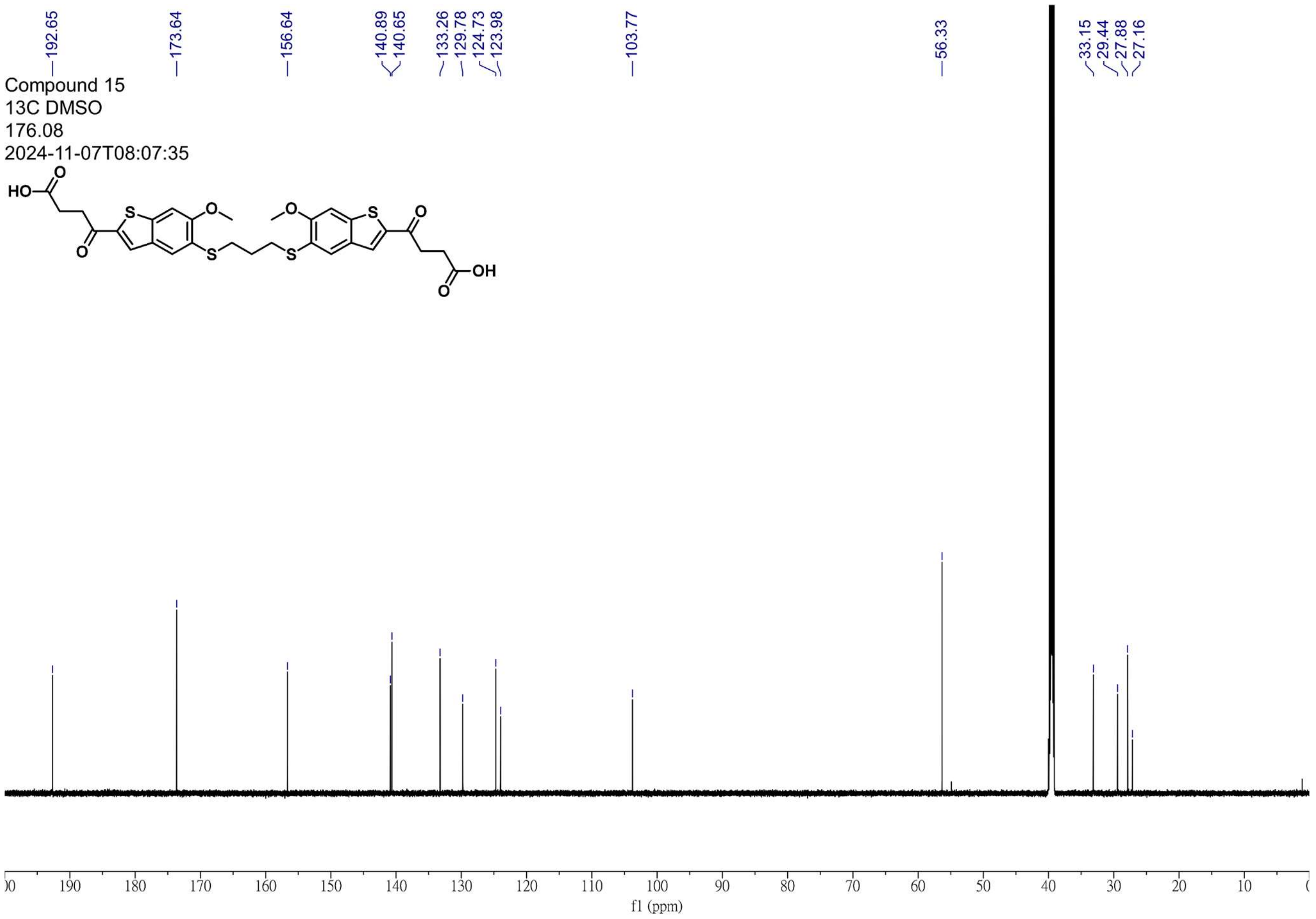
Compound 15
13C DMSO
176.08
2024-11-07T08:07:35
192.65
173.64
156.64
140.89
140.65
133.26
129.78
124.73
123.98
103.77
56.33
33.15
29.44
27.88
27.16
HO
O
S
O
S
S
O
S
O
OH
f1 (ppm)

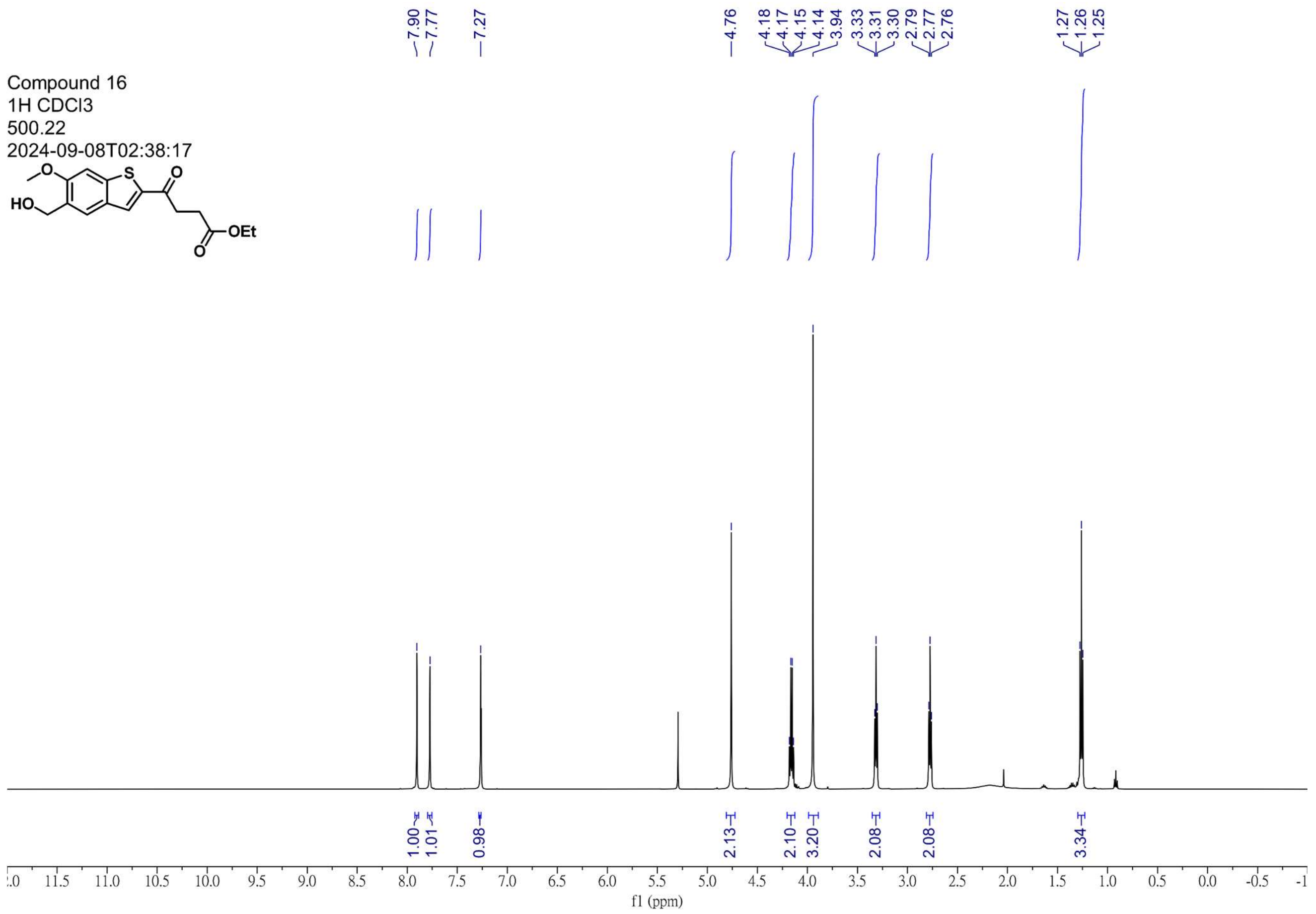
Compound 16
1H CDCl3
500.22
2024-09-08T02:38:17
HO
O
S
O
OEt
O
7.90
7.77
7.27
4.76
4.18
4.17
4.15
4.14
3.94
3.33
3.31
3.30
2.79
2.77
2.76
1.27
1.26
1.25
1.00
1.01
0.98
2.13
2.10
3.20
2.08
2.08
3.34
f1 (ppm)

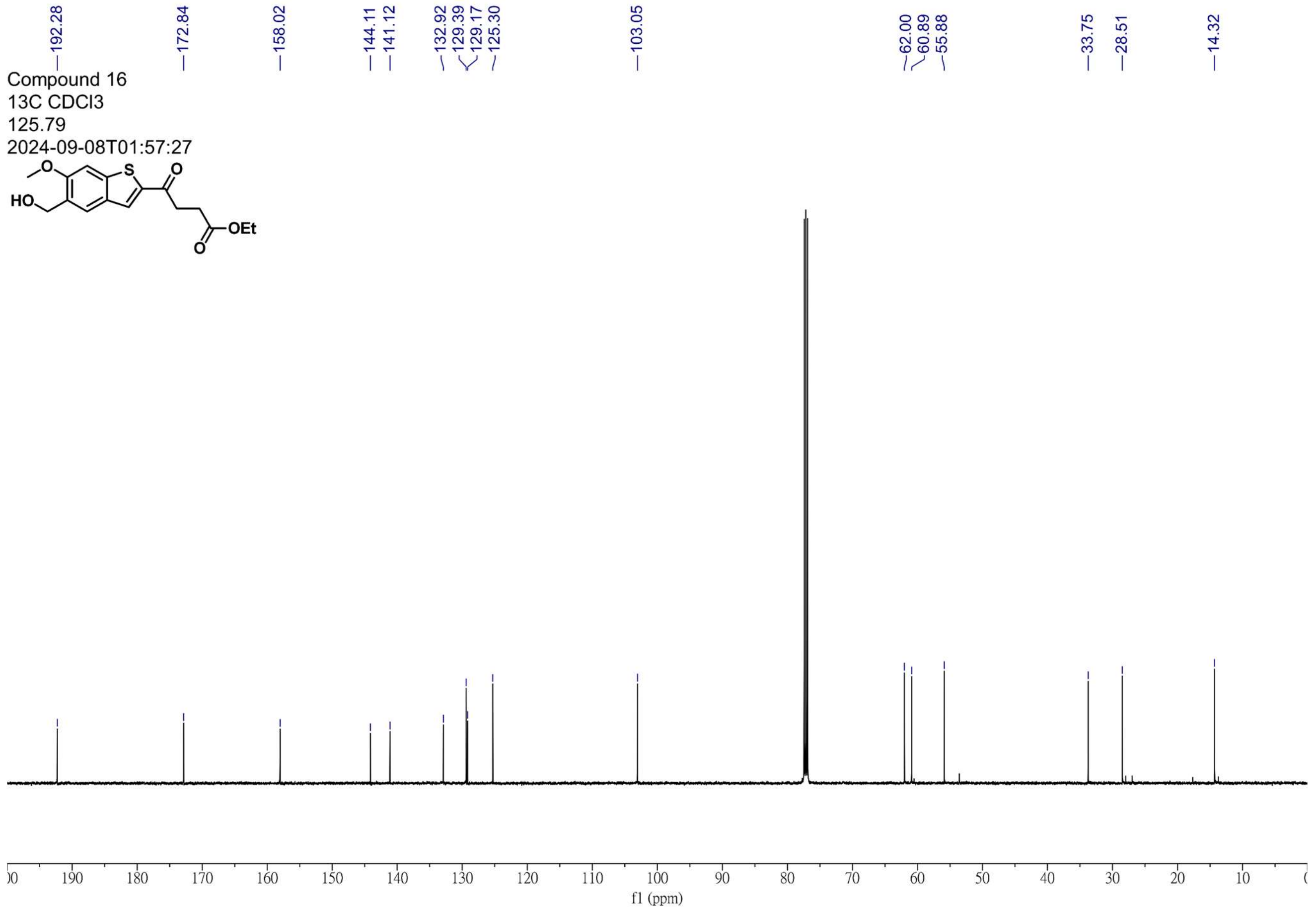
Compound 16
13C CDCl3
125.79
2024-09-08T01:57:27
HO
O
S
O
OEt
O
192.28
172.84
158.02
144.11
141.12
132.92
129.39
129.17
125.30
103.05
62.00
60.89
55.88
33.75
28.51
14.32
f1 (ppm)

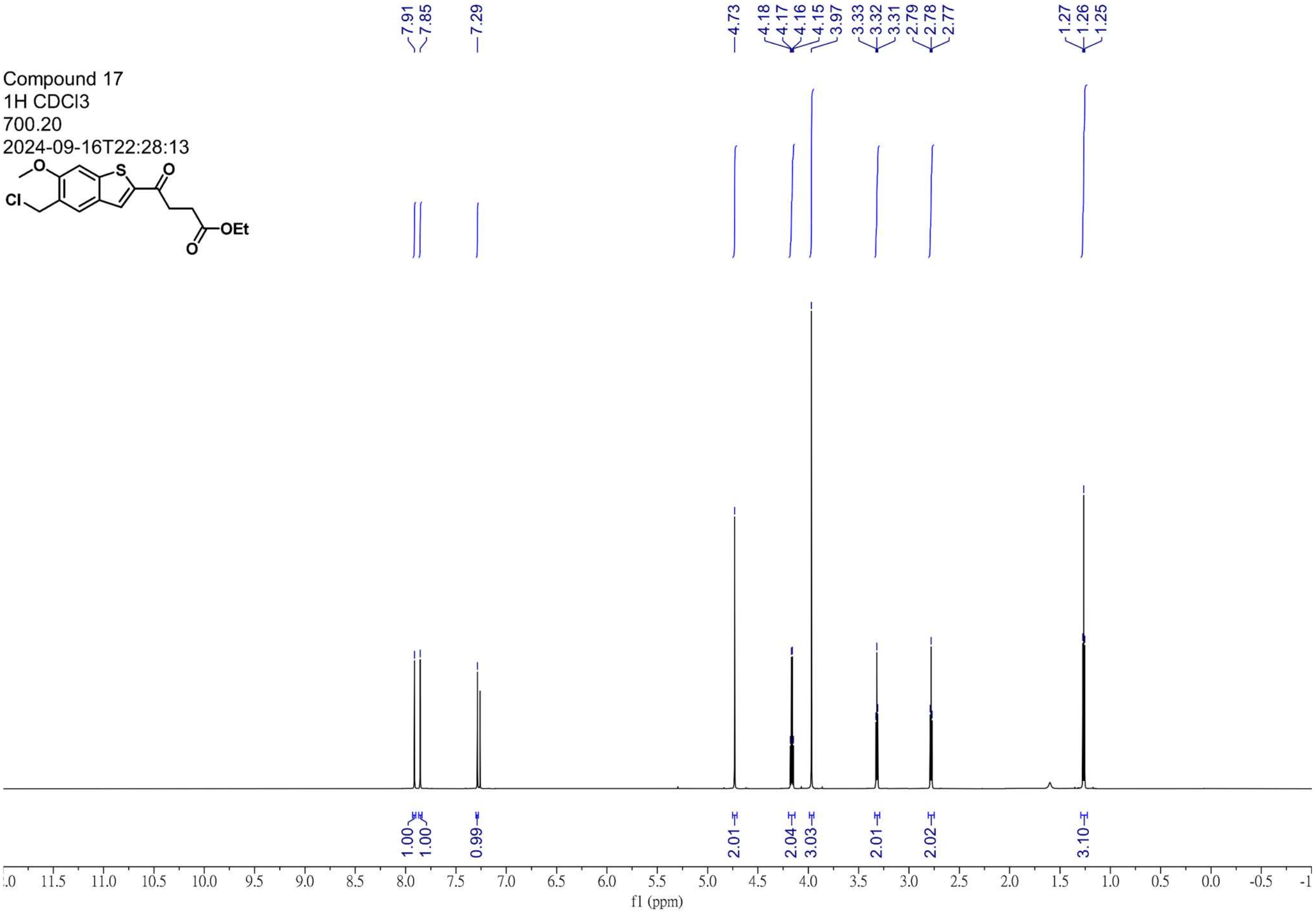
Compound 17
1H CDCl3
700.20
2024-09-16T22:28:13
Cl
S
O
OEt
O
7.91
7.85
7.29
4.73
4.18
4.17
4.16
4.15
3.97
3.33
3.32
3.31
2.79
2.78
2.77
1.27
1.26
1.25
1.00
1.00
0.99
2.01
2.04
3.03
2.01
2.02
3.10
f1 (ppm)

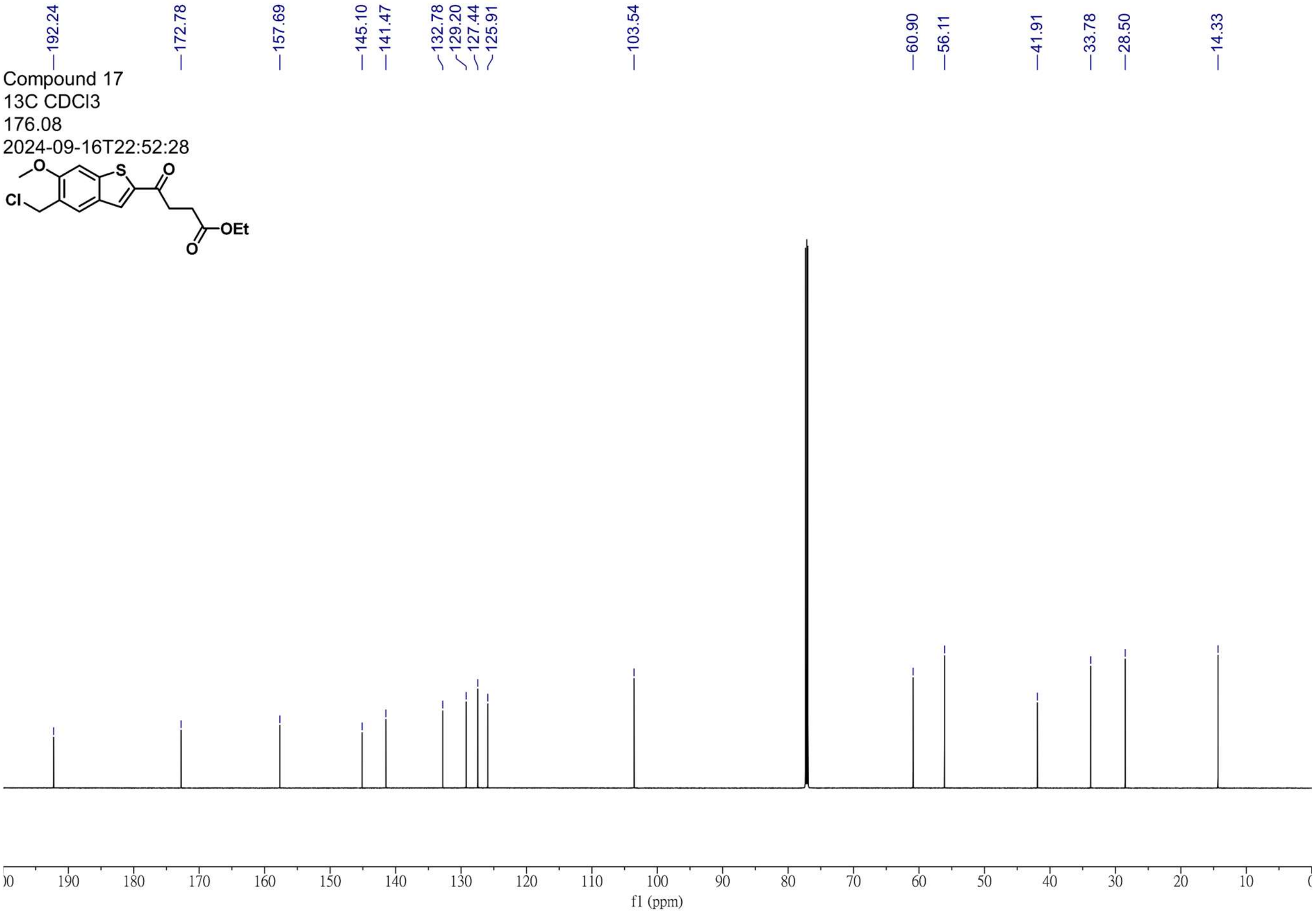
Compound 17
13C CDCl3
176.08
2024-09-16T22:52:28
Cl
S
O
OEt
O
192.24
172.78
157.69
145.10
141.47
132.78
129.20
127.44
125.91
103.54
60.90
56.11
41.91
33.78
28.50
14.33
f1 (ppm)

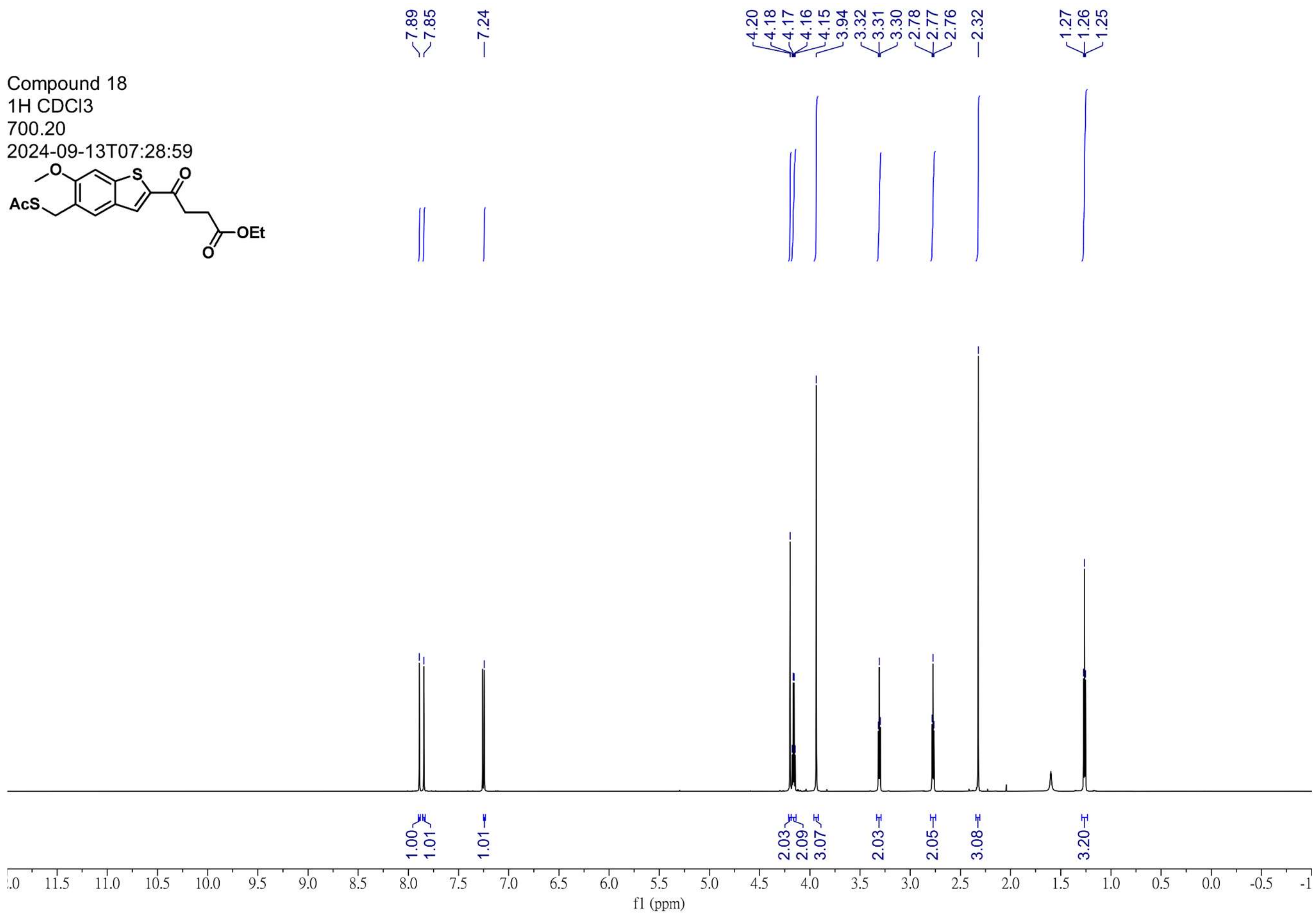
7.89
7.85
7.24
4.20
4.18
4.17
4.16
4.15
3.94
3.32
3.31
3.30
2.78
2.77
2.76
2.32
1.27
1.26
1.25
Compound 18
1H CDCl3
700.20
2024-09-13T07:28:59
AcS
S
O
OEt
1.00
1.01
1.01
2.03
2.09
3.07
2.03
2.05
3.08
3.20
f1 (ppm)

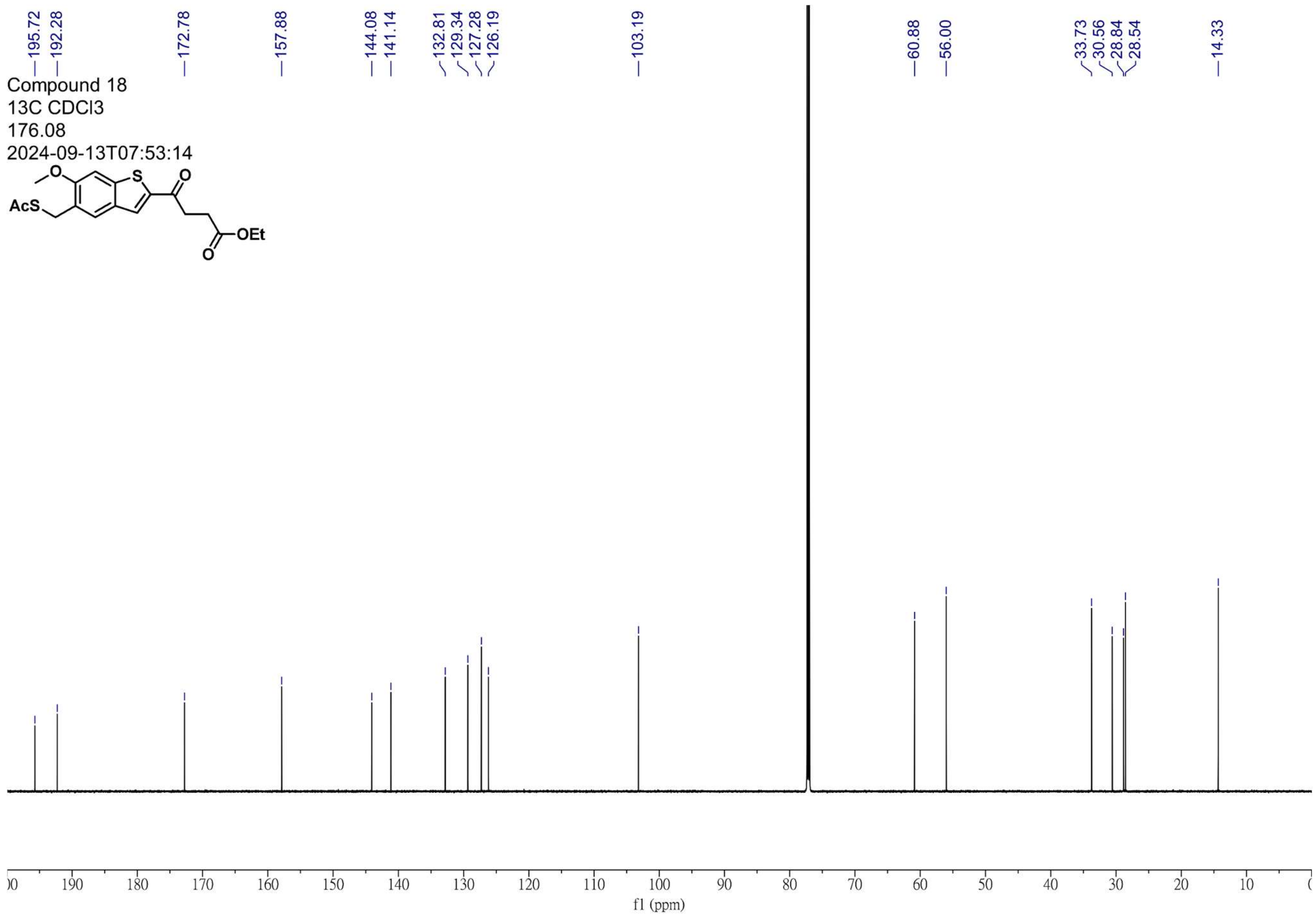
195.72
192.28
172.78
157.88
144.08
141.14
132.81
129.34
127.28
126.19
103.19
60.88
56.00
33.73
30.56
28.84
28.54
14.33
Compound 18
13C CDCl3
176.08
2024-09-13T07:53:14
AcS
S
O
OEt
f1 (ppm)

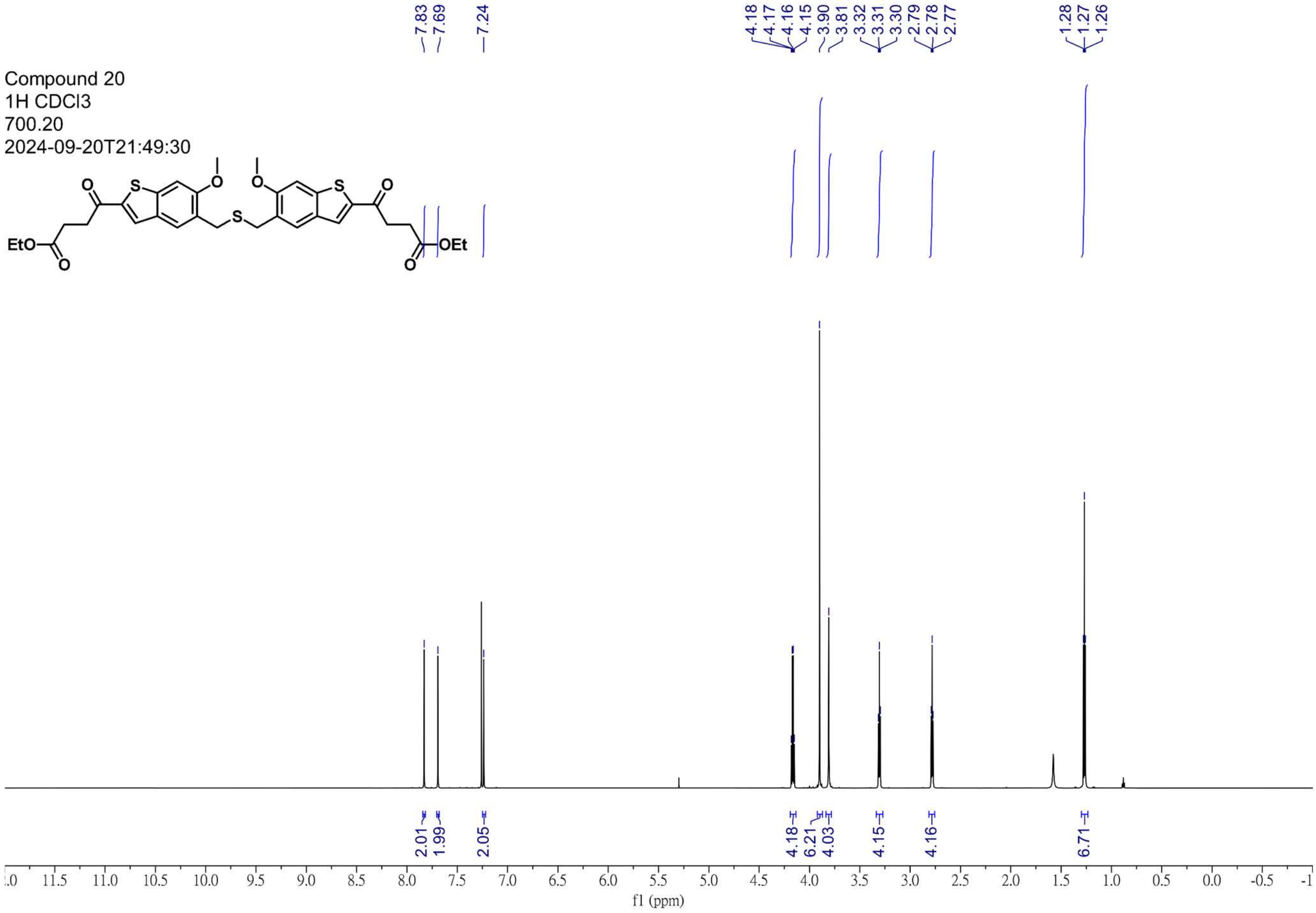
Compound 20
1H CDCl3
700.20
2024-09-20T21:49:30
EtO
O
O
S
O
S
O
S
O
OEt
O
7.83
7.69
7.24
4.18
4.17
4.16
4.15
3.90
3.81
3.32
3.31
3.30
2.79
2.78
2.77
1.28
1.27
1.26
2.01
1.99
2.05
4.18
6.21
4.03
4.15
4.16
6.71
f1 (ppm)

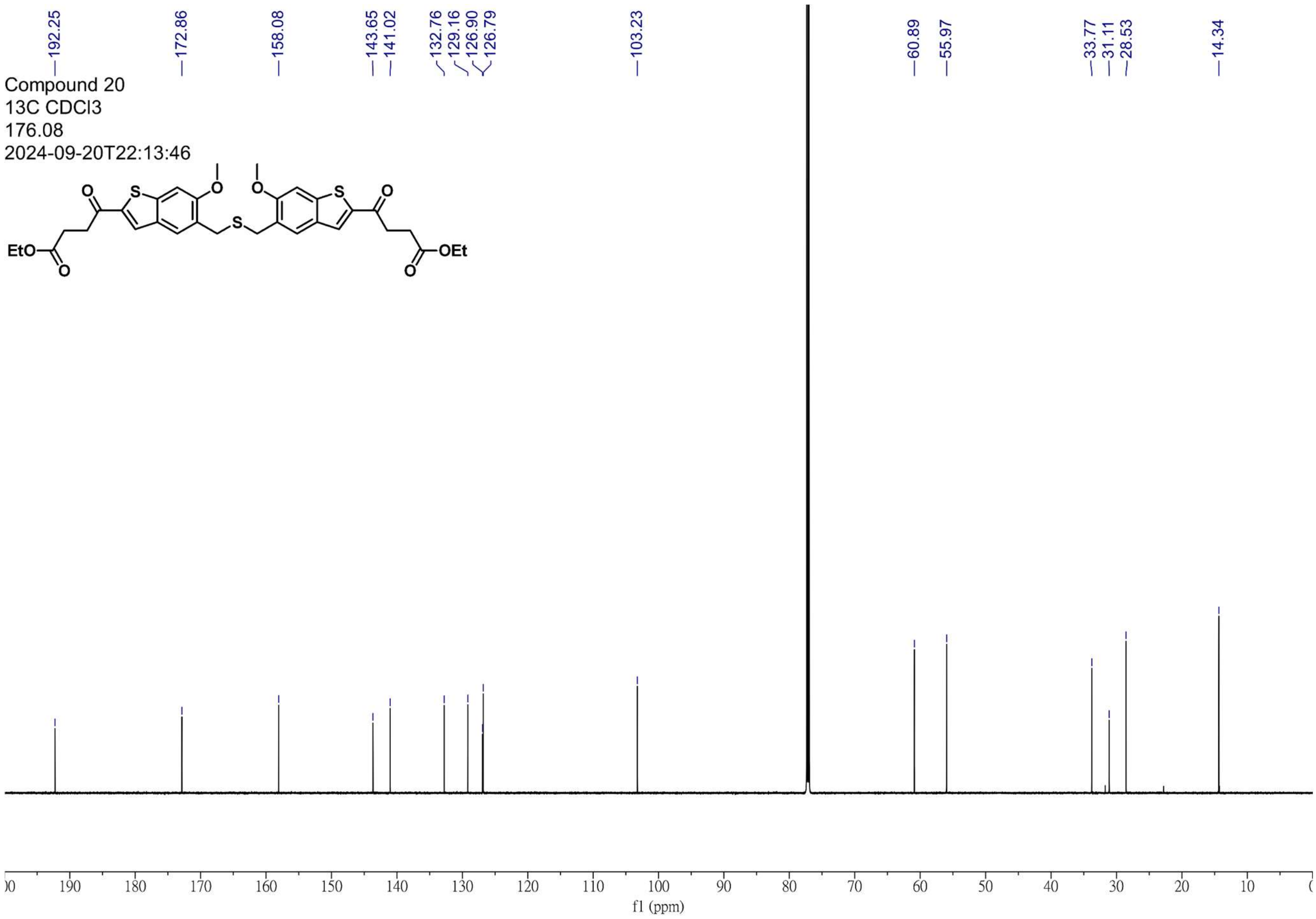
192.25
172.86
158.08
143.65
141.02
132.76
129.16
126.90
126.79
103.23
60.89
55.97
33.77
31.11
28.53
14.34
Compound 20
13C CDCl3
176.08
2024-09-20T22:13:46
EtO
O
S
O
S
O
OEt
f1 (ppm)

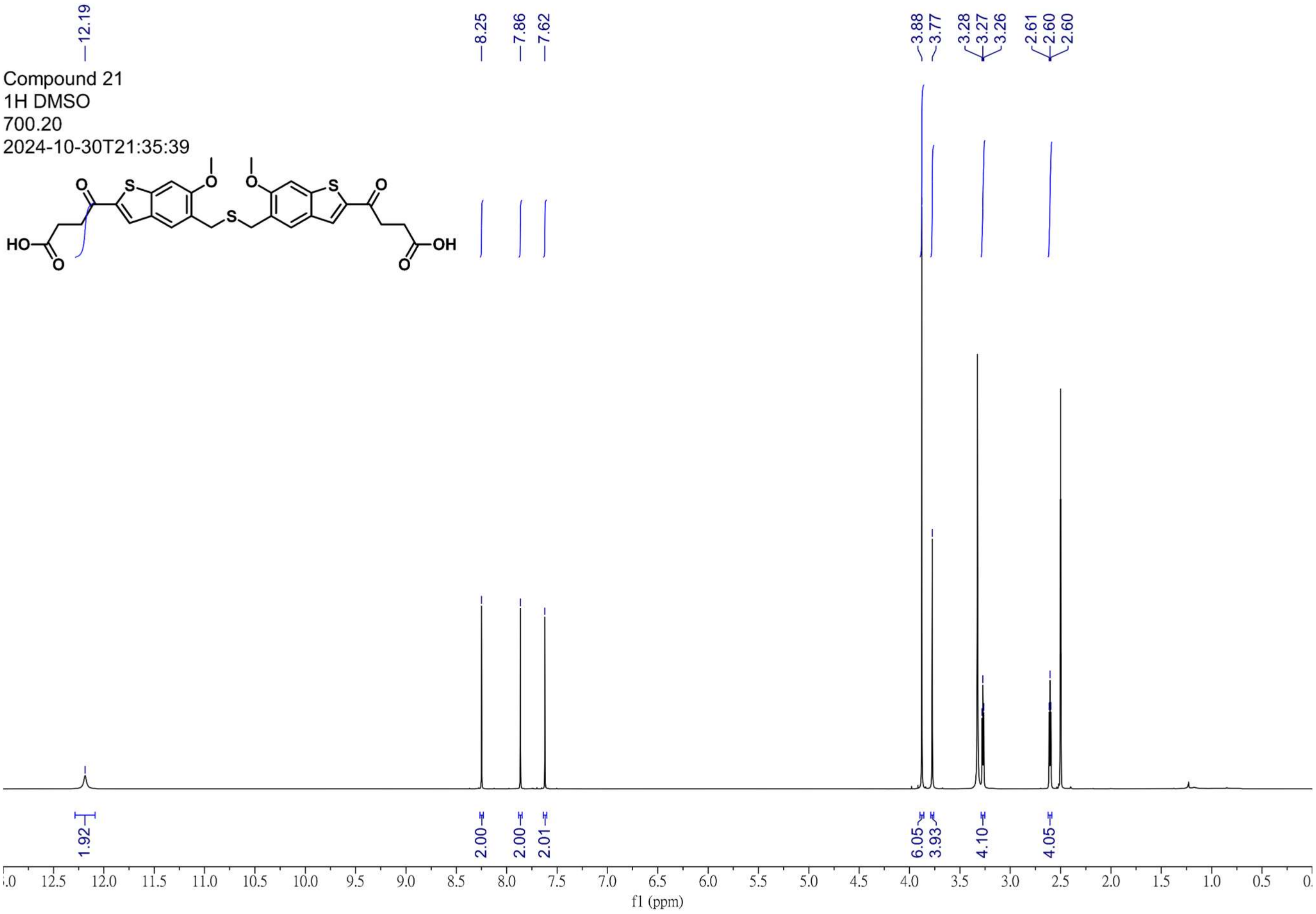
Compound 21
1H DMSO
700.20
2024-10-30T21:35:39
f1 (ppm)

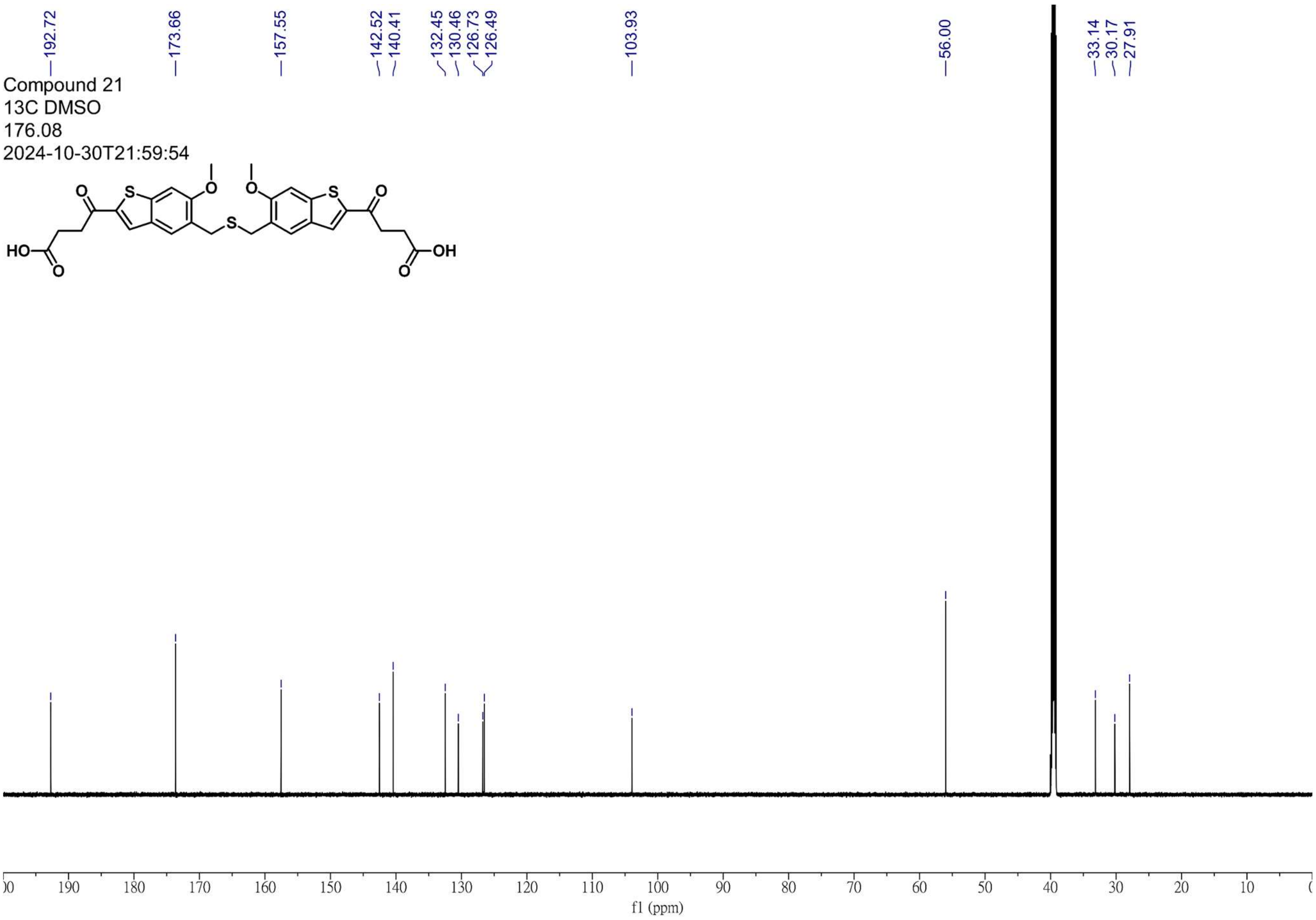
Compound 21
13C DMSO
176.08
2024-10-30T21:59:54
f1 (ppm)

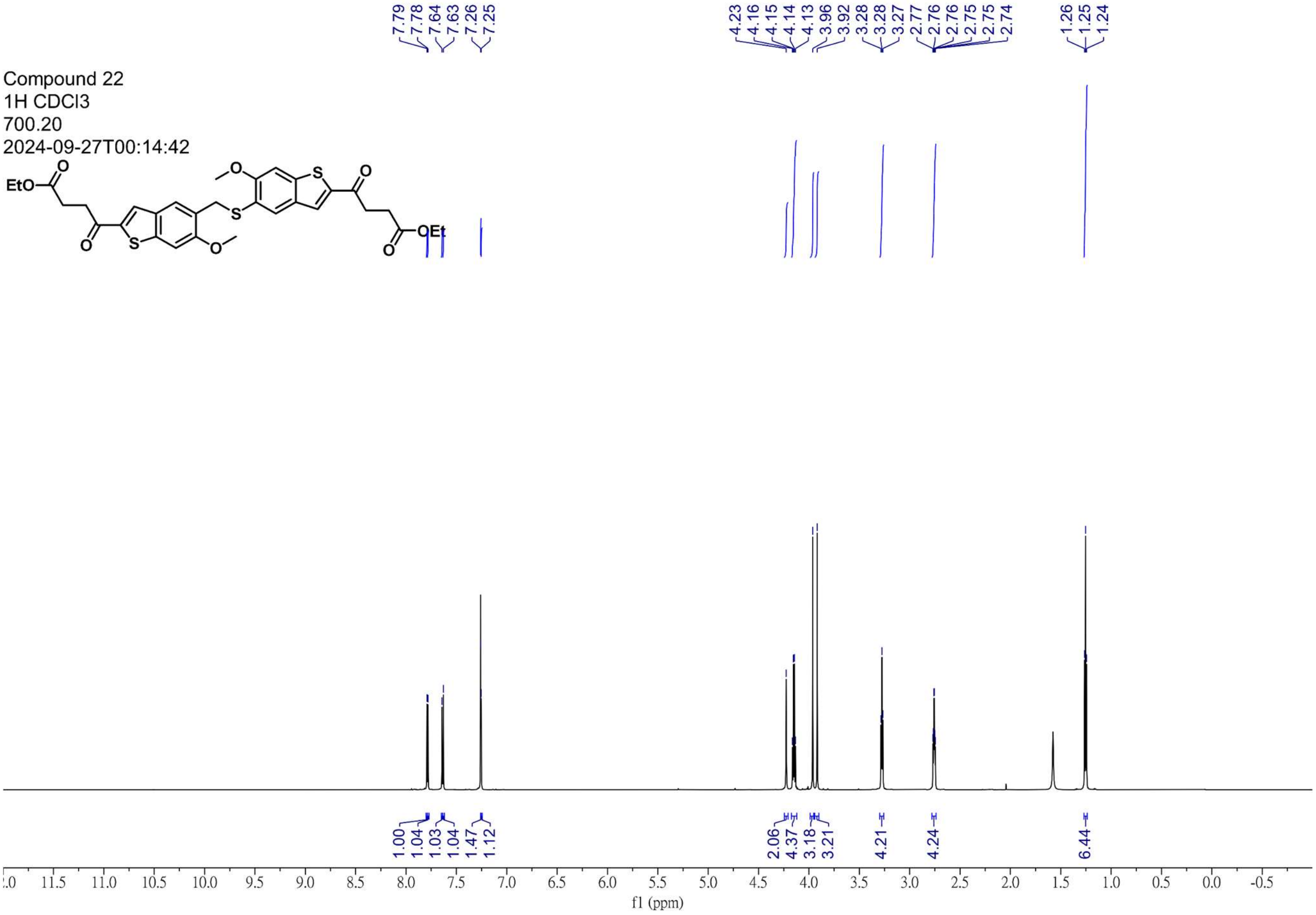
Compound 22
1H CDCl3
700.20
2024-09-27T00:14:42
7.79
7.78
7.64
7.63
7.26
7.25
4.23
4.16
4.15
4.14
4.13
3.96
3.92
3.28
3.28
3.27
2.77
2.76
2.76
2.75
2.75
2.74
1.26
1.25
1.24
1.00
1.04
1.03
1.04
1.47
1.12
2.06
4.37
3.18
3.21
4.21
4.24
6.44
f1 (ppm)

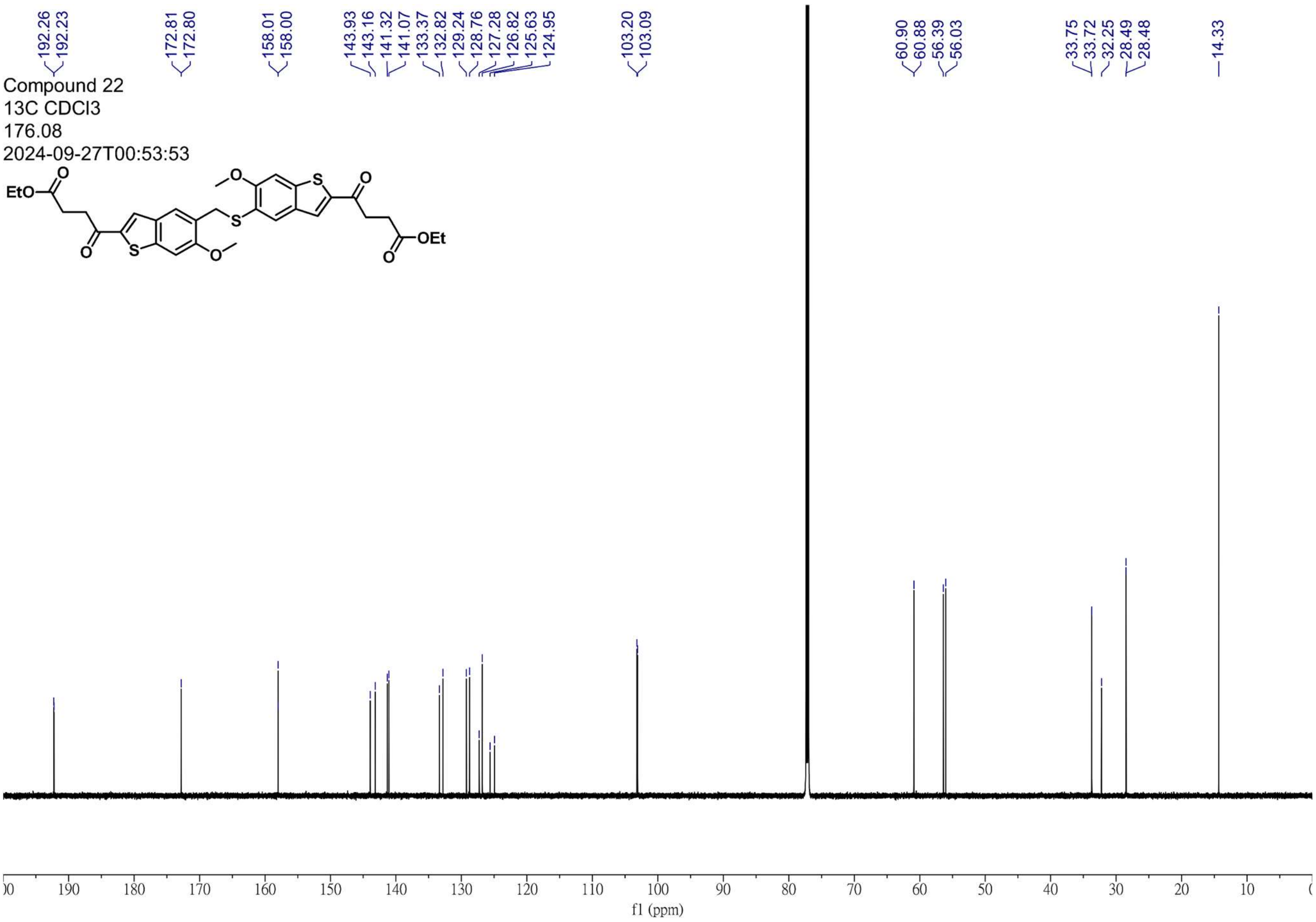
192.26
192.23
172.81
172.80
158.01
158.00
143.93
143.16
141.32
141.07
133.37
132.82
129.24
128.76
127.28
126.82
125.63
124.95
103.20
103.09
60.90
60.88
56.39
56.03
33.75
33.72
32.25
28.49
28.48
14.33
Compound 22
13C CDCl3
176.08
2024-09-27T00:53:53
f1 (ppm)

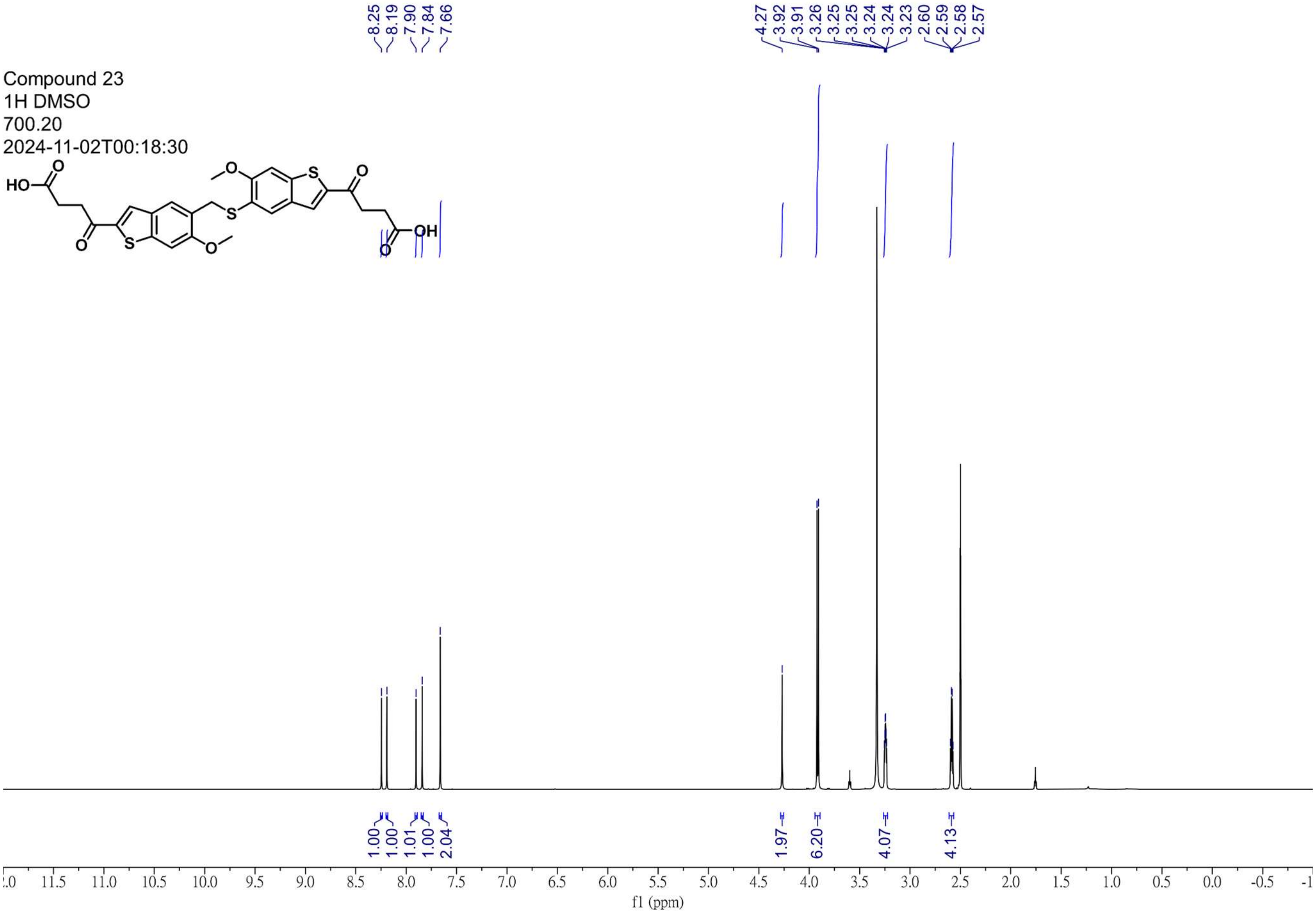
Compound 23
1H DMSO
700.20
2024-11-02T00:18:30
HO
O
O
S
O
S
S
O
O
OH
8.25
8.19
7.90
7.84
7.66
4.27
3.92
3.91
3.26
3.25
3.25
3.24
3.24
3.23
2.60
2.59
2.58
2.57
1.00
1.00
1.01
1.00
2.04
1.97
6.20
4.07
4.13
f1 (ppm)

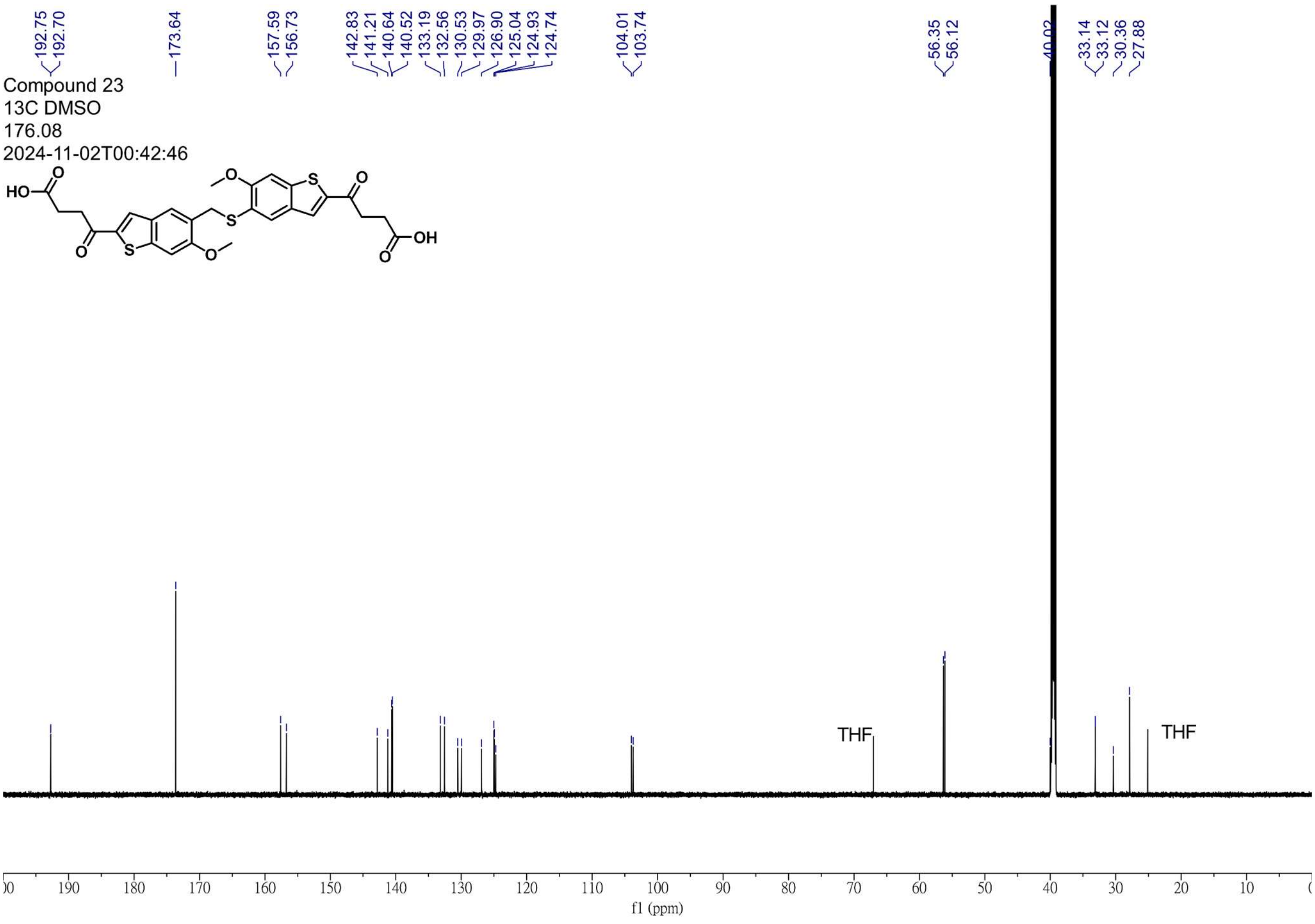
Compound 23
13C DMSO
176.08
2024-11-02T00:42:46
HO
O
O
S
O
S
S
O
O
OH
192.75
192.70
173.64
157.59
156.73
142.83
141.21
140.64
140.52
133.19
132.56
130.53
129.97
126.90
125.04
124.93
124.74
104.01
103.74
56.35
56.12
40.02
33.14
33.12
30.36
27.88
THF
THF
f1 (ppm)

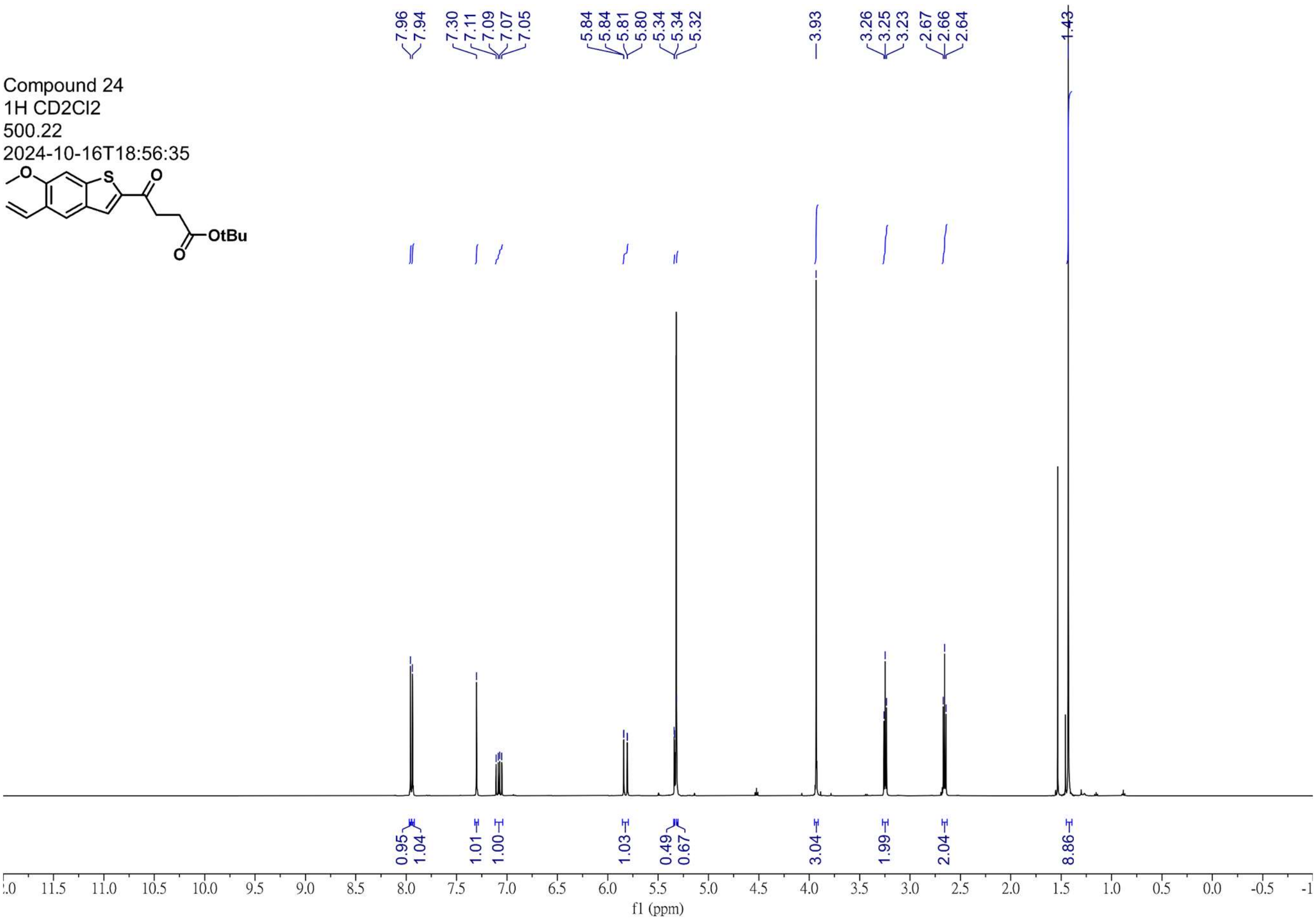
Compound 24
1H CD2Cl2
500.22
2024-10-16T18:56:35
S
O
OtBu
7.96
7.94
7.30
7.11
7.09
7.07
7.05
5.84
5.84
5.81
5.80
5.34
5.34
5.32
3.93
3.26
3.25
3.23
2.67
2.66
2.64
1.43
0.95
1.04
1.01
1.00
1.03
0.49
0.67
3.04
1.99
2.04
8.86
f1 (ppm)

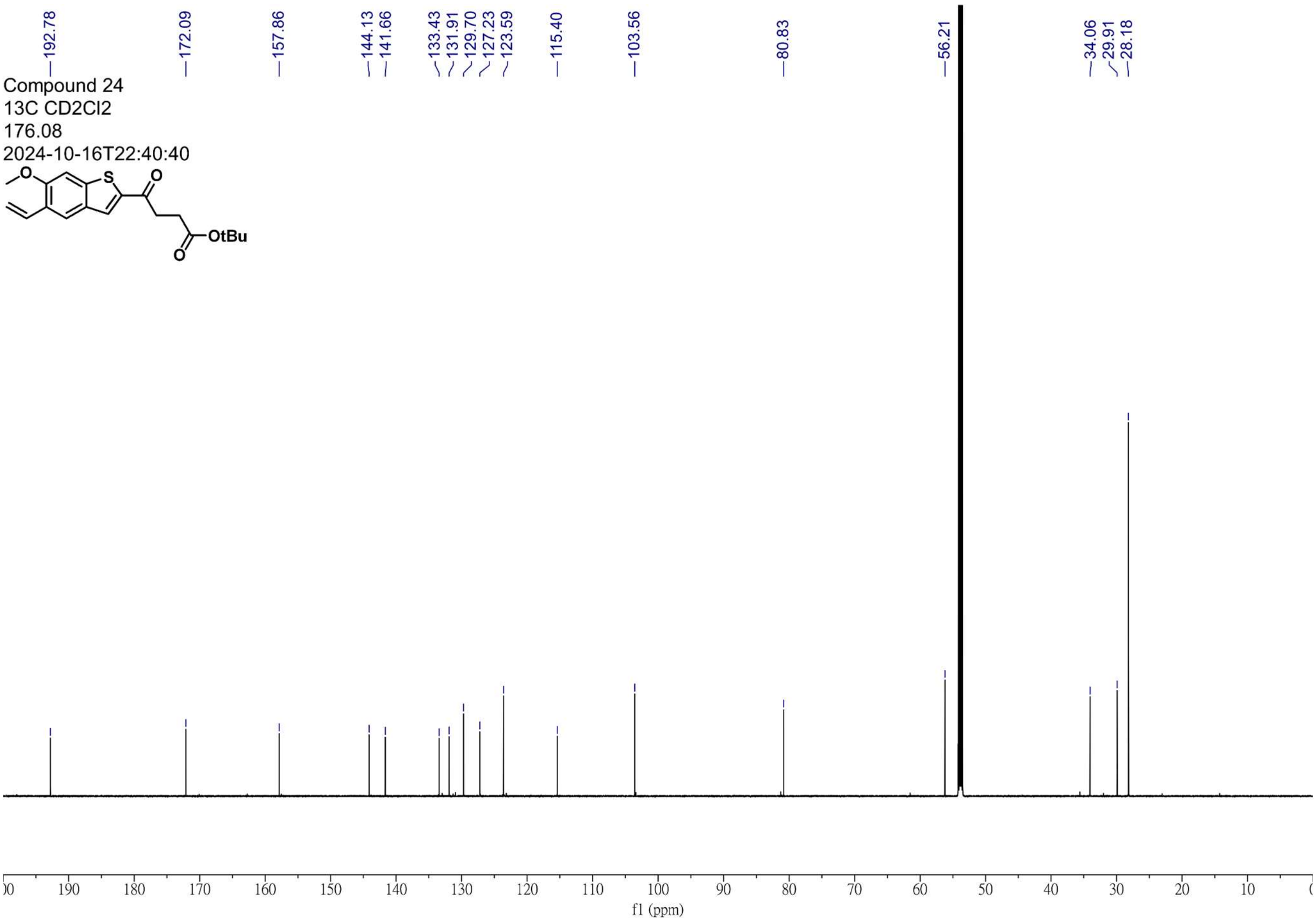
Compound 24
13C CD2Cl2
176.08
2024-10-16T22:40:40
S
O
OtBu
192.78
172.09
157.86
144.13
141.66
133.43
131.91
129.70
127.23
123.59
115.40
103.56
80.83
56.21
34.06
29.91
28.18
f1 (ppm)

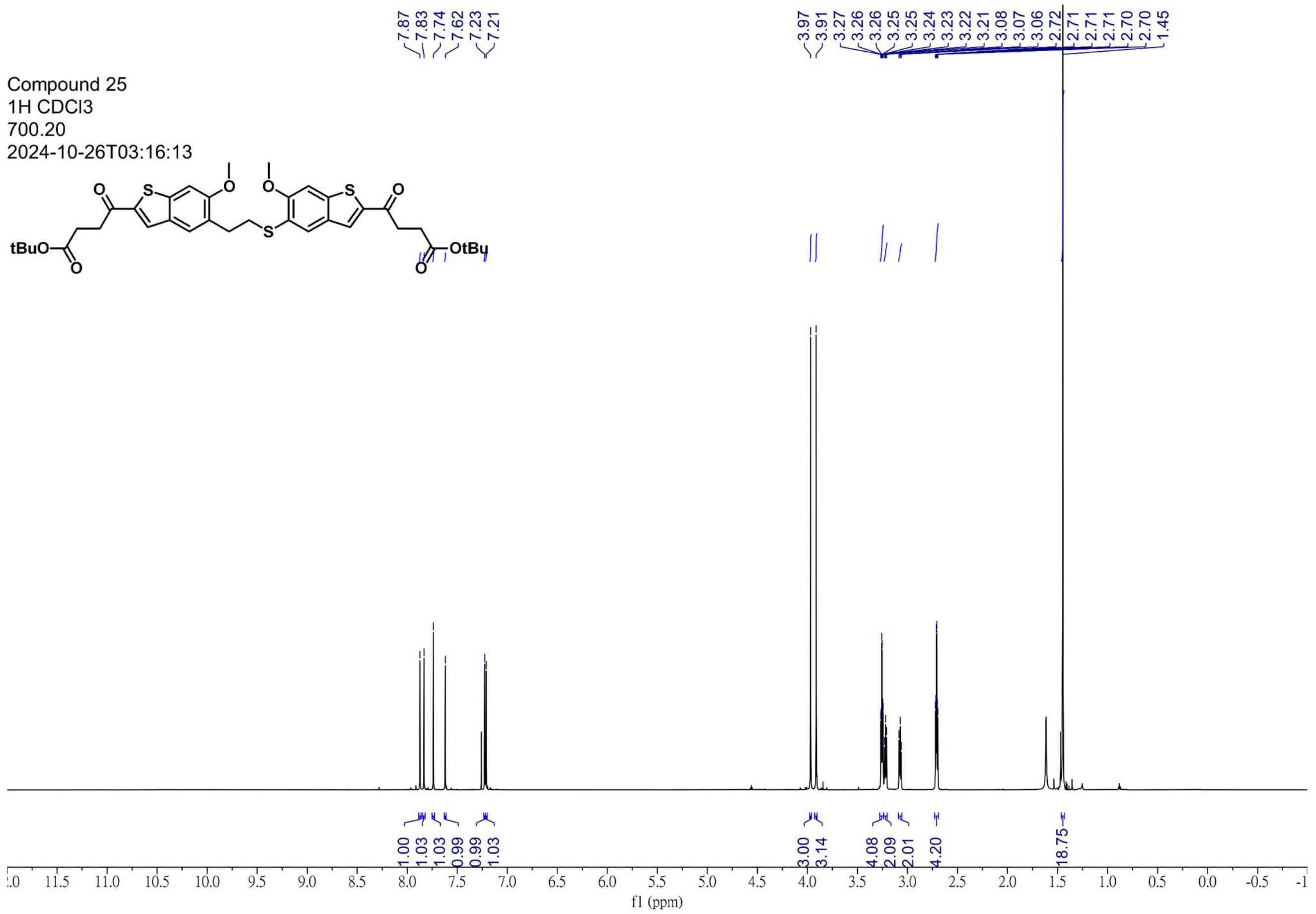
Compound 25
1H CDCl3
700.20
2024-10-26T03:16:13
tBuO
OtBu
7.87 7.83 7.74 7.62 7.23 7.21
3.97 3.91 3.27 3.26 3.26 3.25 3.25 3.24 3.23 3.22 3.21 3.08 3.07 3.06 2.72 2.71 2.71 2.71 2.70 2.70 1.45
1.00 1.03 1.03 0.99 0.99 1.03
3.00 3.14 4.08 2.09 2.01 4.20 18.75
f1 (ppm)

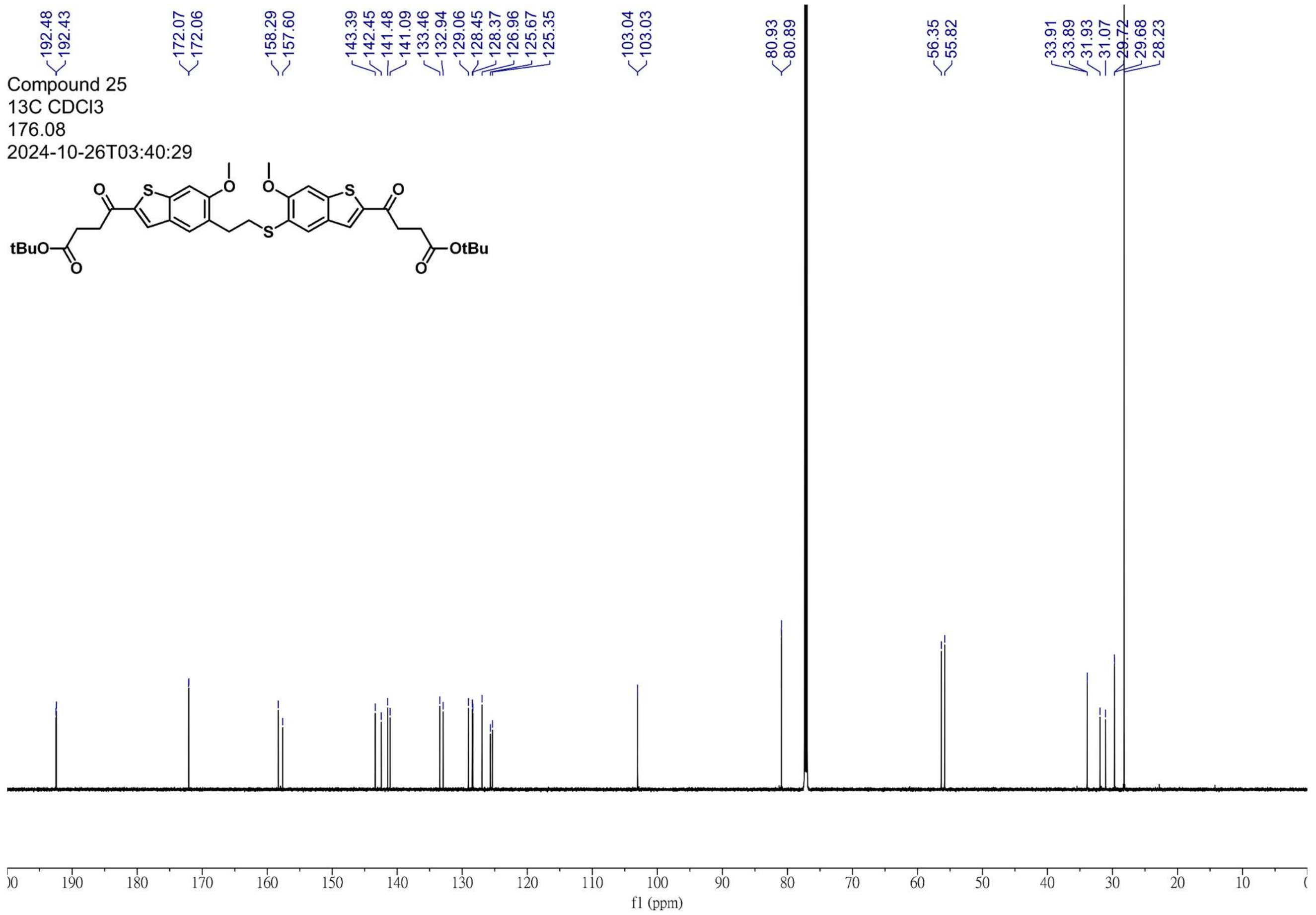
192.48 192.43 172.07 172.06 158.29 157.60 143.39 142.45 141.48 141.09 133.46 132.94 129.06 128.45 128.37 126.96 125.67 125.35 103.04 103.03 80.93 80.89 56.35 55.82 33.91 33.89 31.93 31.07 29.72 29.68 28.23
Compound 25
13C CDCl3
176.08
2024-10-26T03:40:29
tBuO
OtBu
f1 (ppm)

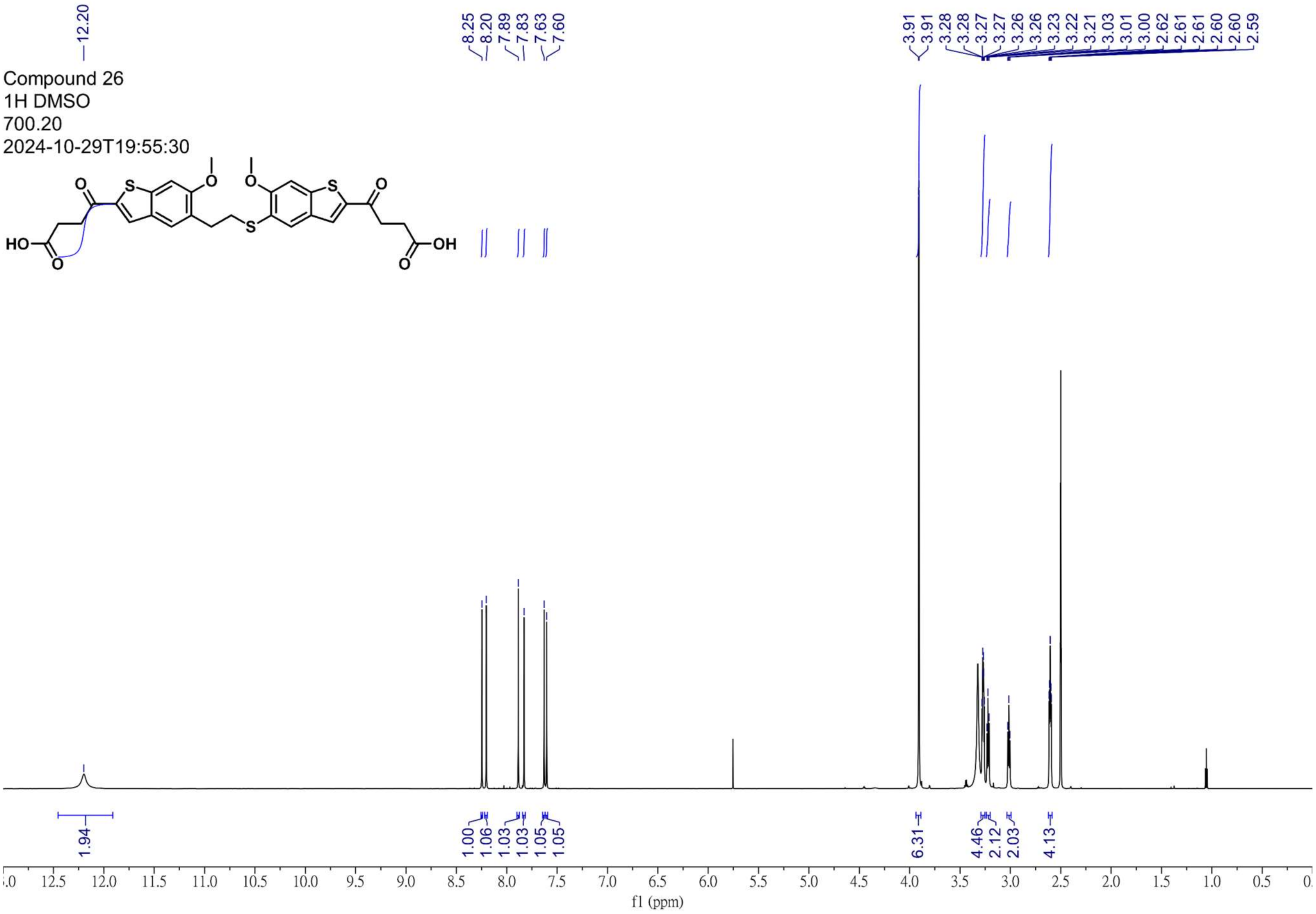
Compound 26
1H DMSO
700.20
2024-10-29T19:55:30
HO
O
S
O
S
O
S
O
OH
O
12.20
8.25
8.20
7.89
7.83
7.63
7.60
3.91
3.91
3.28
3.28
3.27
3.27
3.26
3.26
3.23
3.22
3.21
3.03
3.01
3.00
2.62
2.61
2.61
2.60
2.60
2.59
1.94
1.00
1.06
1.03
1.03
1.05
1.05
6.31
4.46
2.12
2.03
4.13
f1 (ppm)

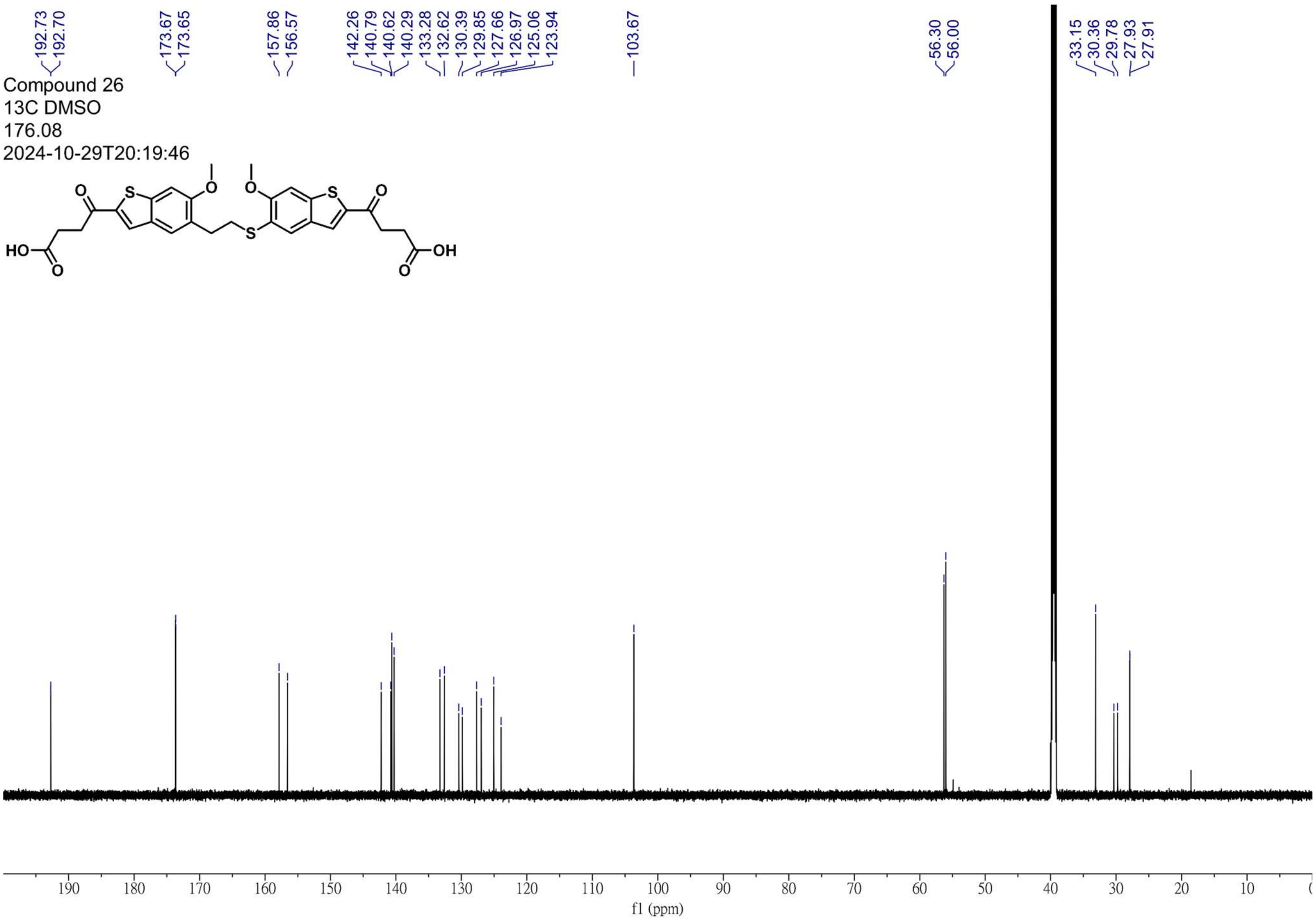
Compound 26
13C DMSO
176.08
2024-10-29T20:19:46
HO
O
S
O
S
O
S
O
OH
O
192.73
192.70
173.67
173.65
157.86
156.57
142.26
140.79
140.62
140.29
133.28
132.62
130.39
129.85
127.66
126.97
125.06
123.94
103.67
56.30
56.00
33.15
30.36
29.78
27.93
27.91
f1 (ppm)

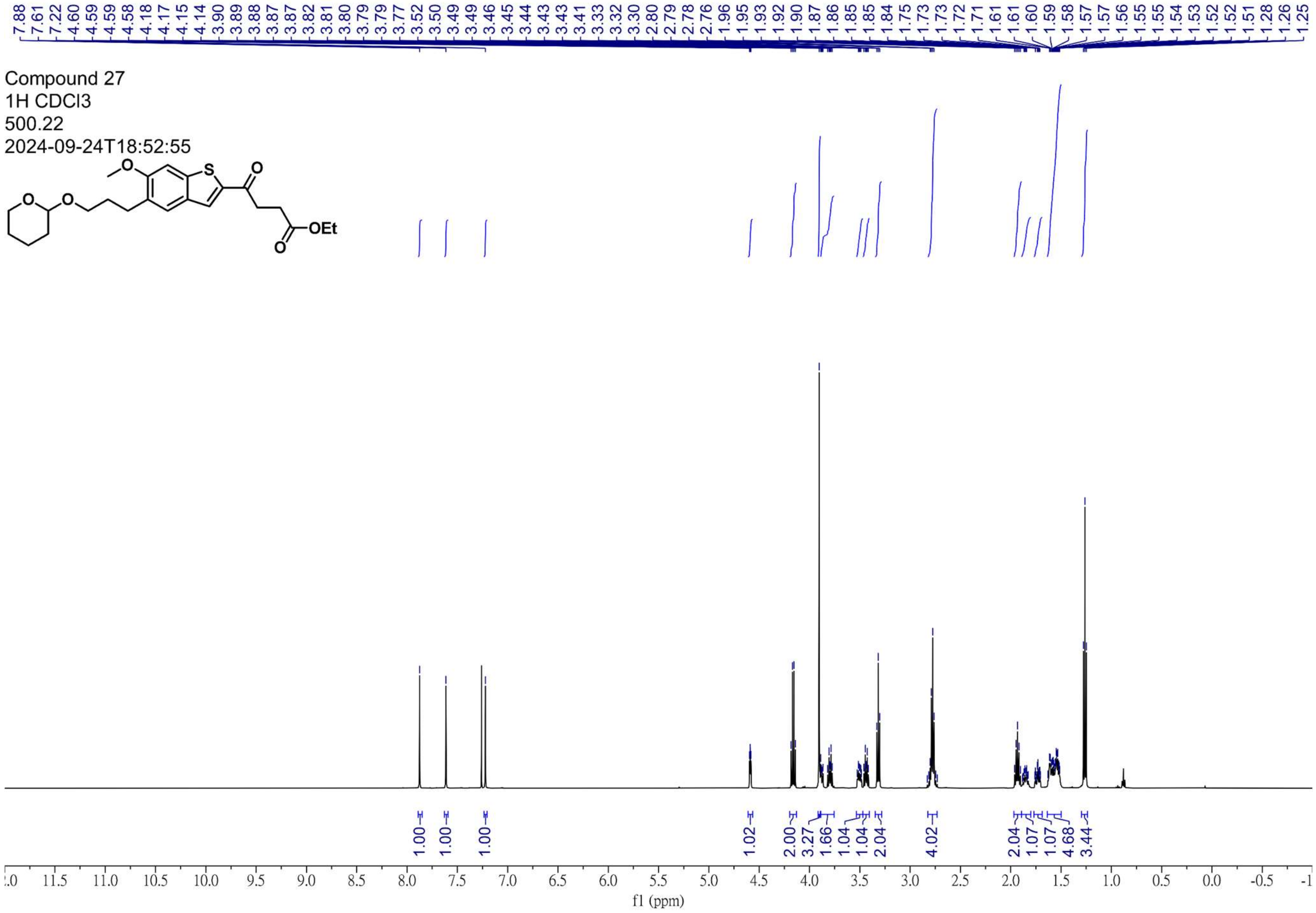
7.88 7.61 7.22 4.60 4.59 4.59 4.58 4.18 4.17 4.15 4.14 3.90 3.89 3.88 3.87 3.87 3.82 3.81 3.80 3.79 3.79 3.77 3.52 3.50 3.49 3.49 3.46 3.45 3.44 3.43 3.43 3.41 3.33 3.32 3.30 2.80 2.79 2.78 2.76 1.96 1.95 1.93 1.92 1.90 1.87 1.86 1.85 1.85 1.84 1.75 1.73 1.73 1.73 1.72 1.71 1.61 1.61 1.60 1.59 1.58 1.57 1.57 1.56 1.55 1.55 1.54 1.53 1.52 1.52 1.51 1.28 1.26 1.25
Compound 27
1H CDCl3
500.22
2024-09-24T18:52:55
O
S
O
O
O
OEt
O
1.00 1.00 1.00 1.02 2.00 3.27 1.66 1.04 1.04 2.04 4.02 2.04 1.07 1.07 4.68 3.44
f1 (ppm)

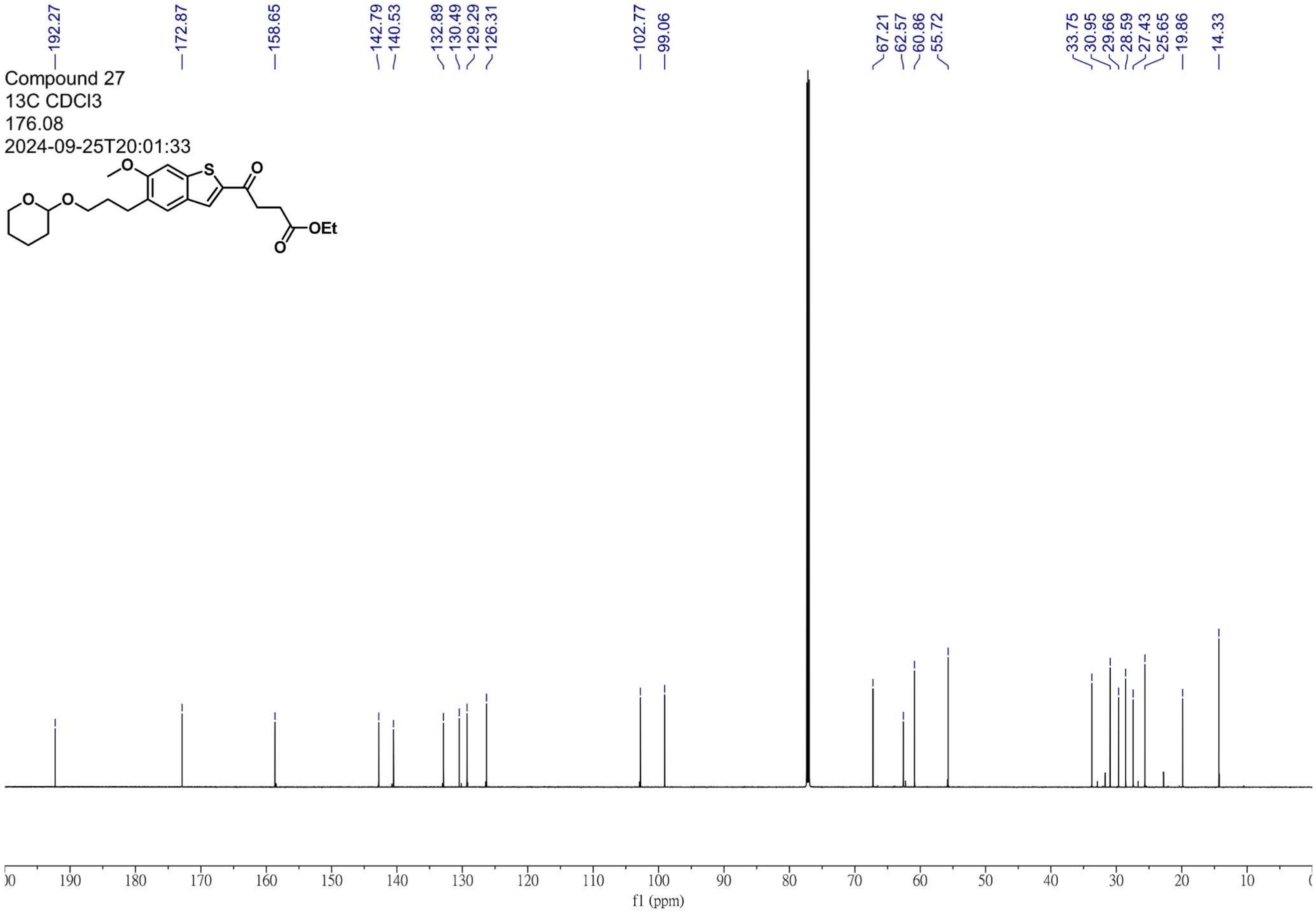
192.27 172.87 158.65 142.79 140.53 132.89 130.49 129.29 126.31 102.77 99.06 67.21 62.57 60.86 55.72 33.75 30.95 29.66 28.59 27.43 25.65 19.86 14.33
Compound 27
13C CDCl3
176.08
2024-09-25T20:01:33
O
S
O
O
O
OEt
O
f1 (ppm)

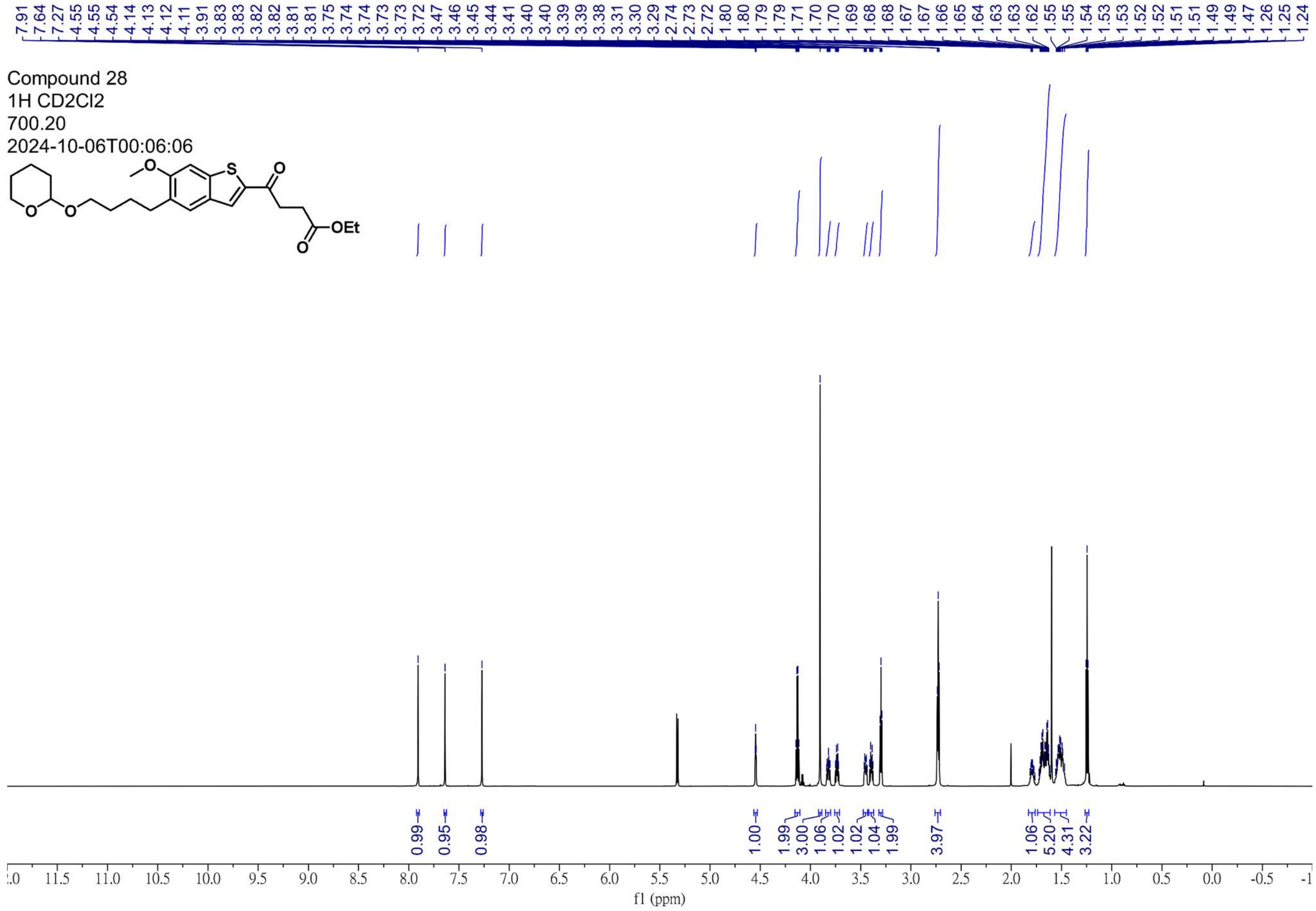
7.91 7.64 7.27 4.55 4.55 4.54 4.14 4.13 4.12 4.11 3.91 3.83 3.83 3.82 3.82 3.81 3.81 3.75 3.74 3.74 3.73 3.73 3.72 3.47 3.46 3.45 3.44 3.41 3.40 3.40 3.39 3.39 3.38 3.31 3.30 3.29 2.74 2.73 2.72 1.80 1.80 1.79 1.79 1.71 1.70 1.70 1.69 1.68 1.68 1.67 1.67 1.66 1.65 1.64 1.63 1.63 1.62 1.55 1.55 1.54 1.53 1.53 1.52 1.52 1.51 1.51 1.49 1.47 1.26 1.25 1.24
Compound 28
1H CD2Cl2
700.20
2024-10-06T00:06:06
S
O
OEt
0.99 0.95 0.98 1.00 1.99 3.00 1.06 1.02 1.02 1.04 1.99 3.97 1.06 5.20 4.31 3.22
11.5 11.0 10.5 10.0 9.5 9.0 8.5 8.0 7.5 7.0 6.5 6.0 5.5 5.0 4.5 4.0 3.5 3.0 2.5 2.0 1.5 1.0 0.5 0.0 -0.5
f1 (ppm)

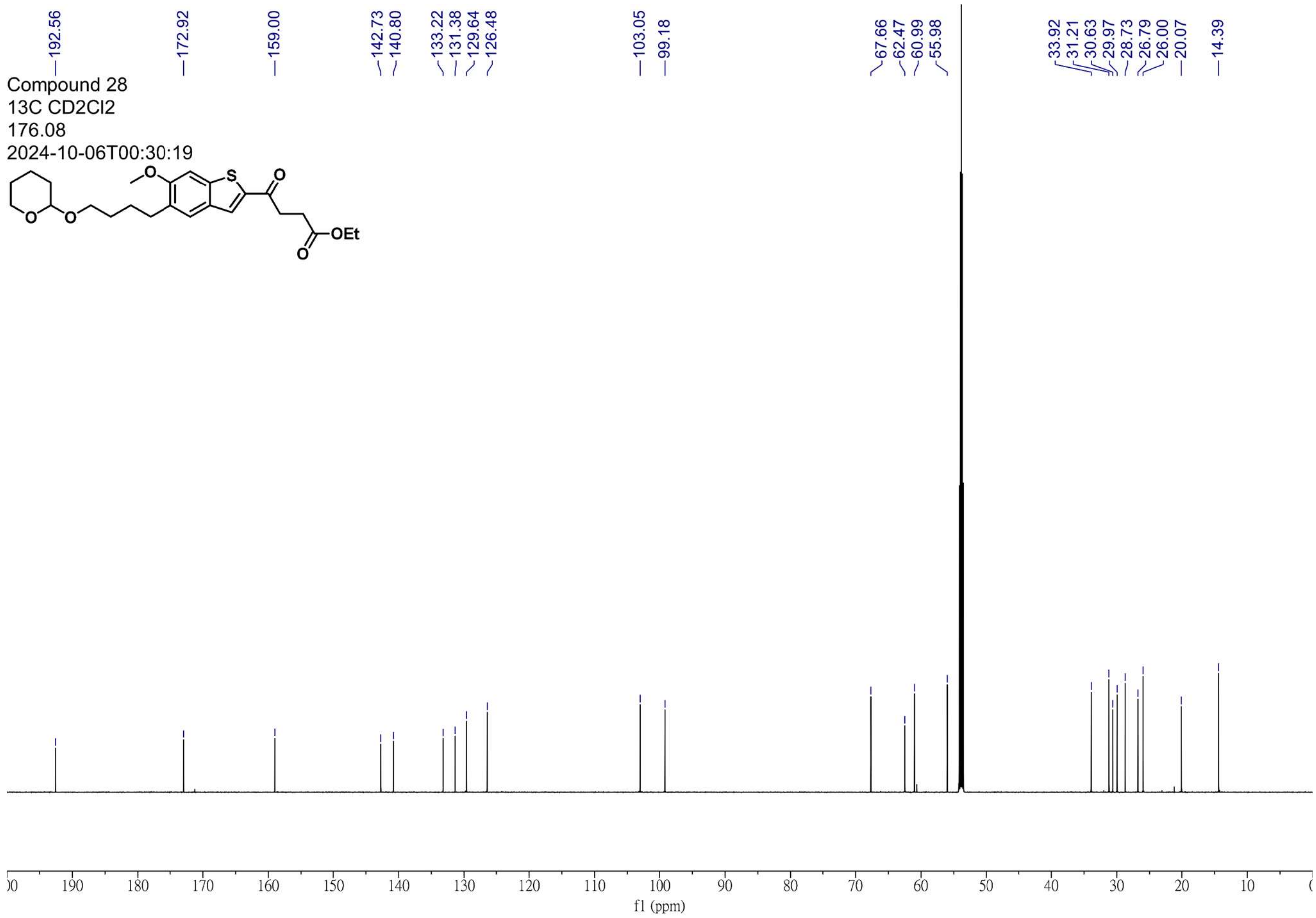
192.56 172.92 159.00 142.73 140.80 133.22 131.38 129.64 126.48 103.05 99.18 67.66 62.47 60.99 55.98 33.92 31.21 30.63 29.97 28.73 26.79 26.00 20.07 14.39
Compound 28
13C CD2Cl2
176.08
2024-10-06T00:30:19
S
O
OEt
190 180 170 160 150 140 130 120 110 100 90 80 70 60 50 40 30 20 10
f1 (ppm)

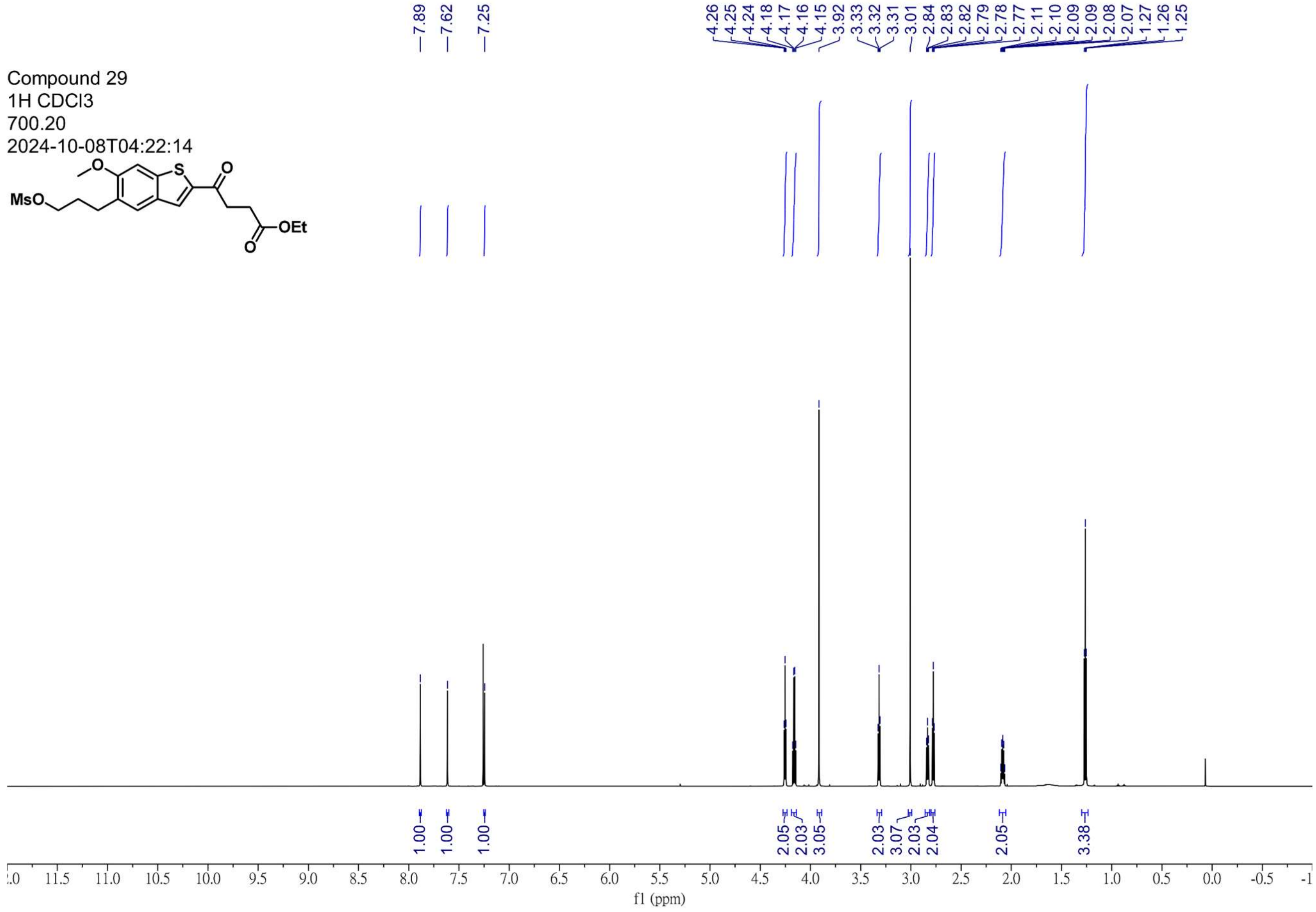
Compound 29
1H CDCl3
700.20
2024-10-08T04:22:14
MsO
OEt
f1 (ppm)

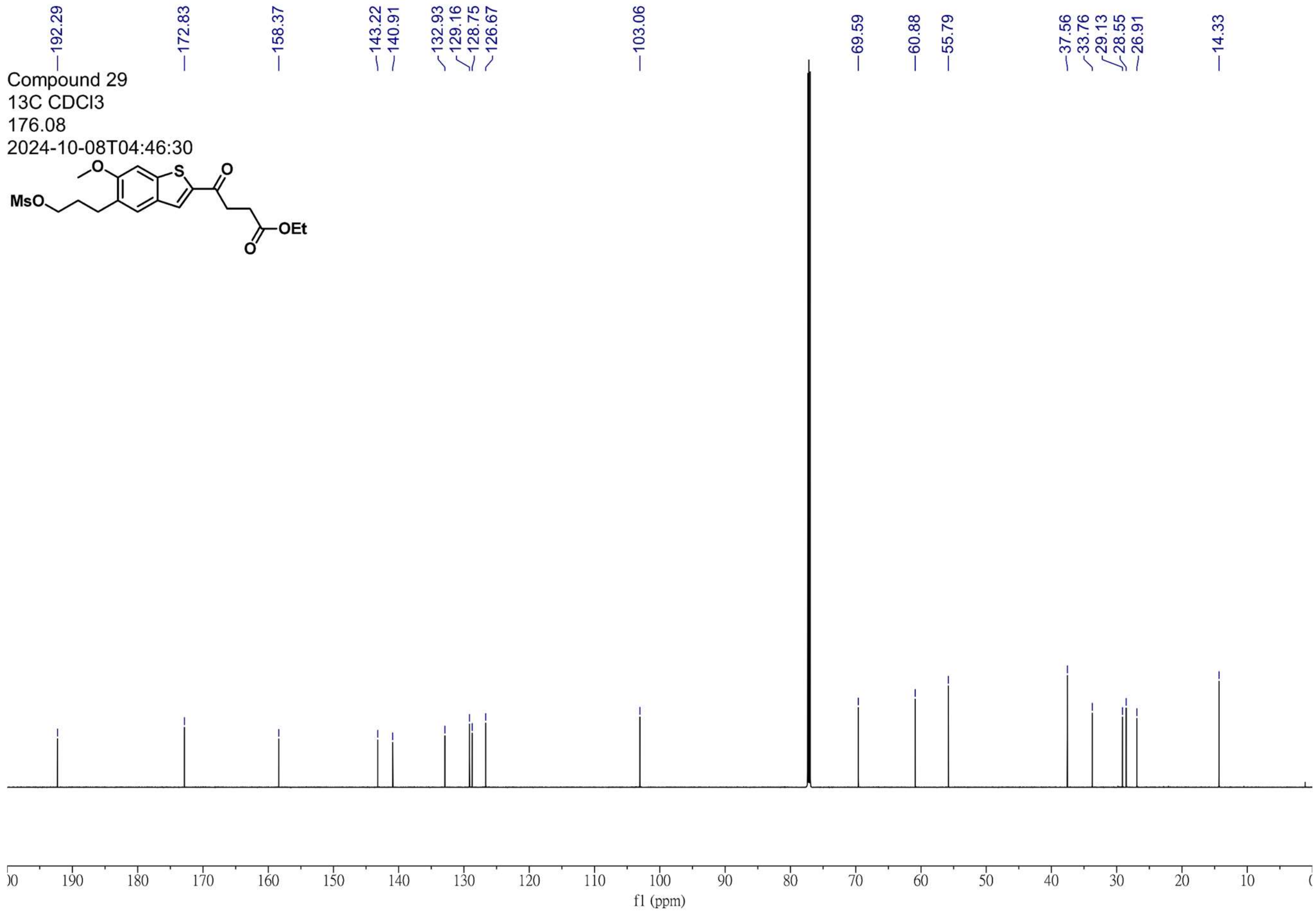
Compound 29
13C CDCl3
176.08
2024-10-08T04:46:30
MsO
OEt
f1 (ppm)

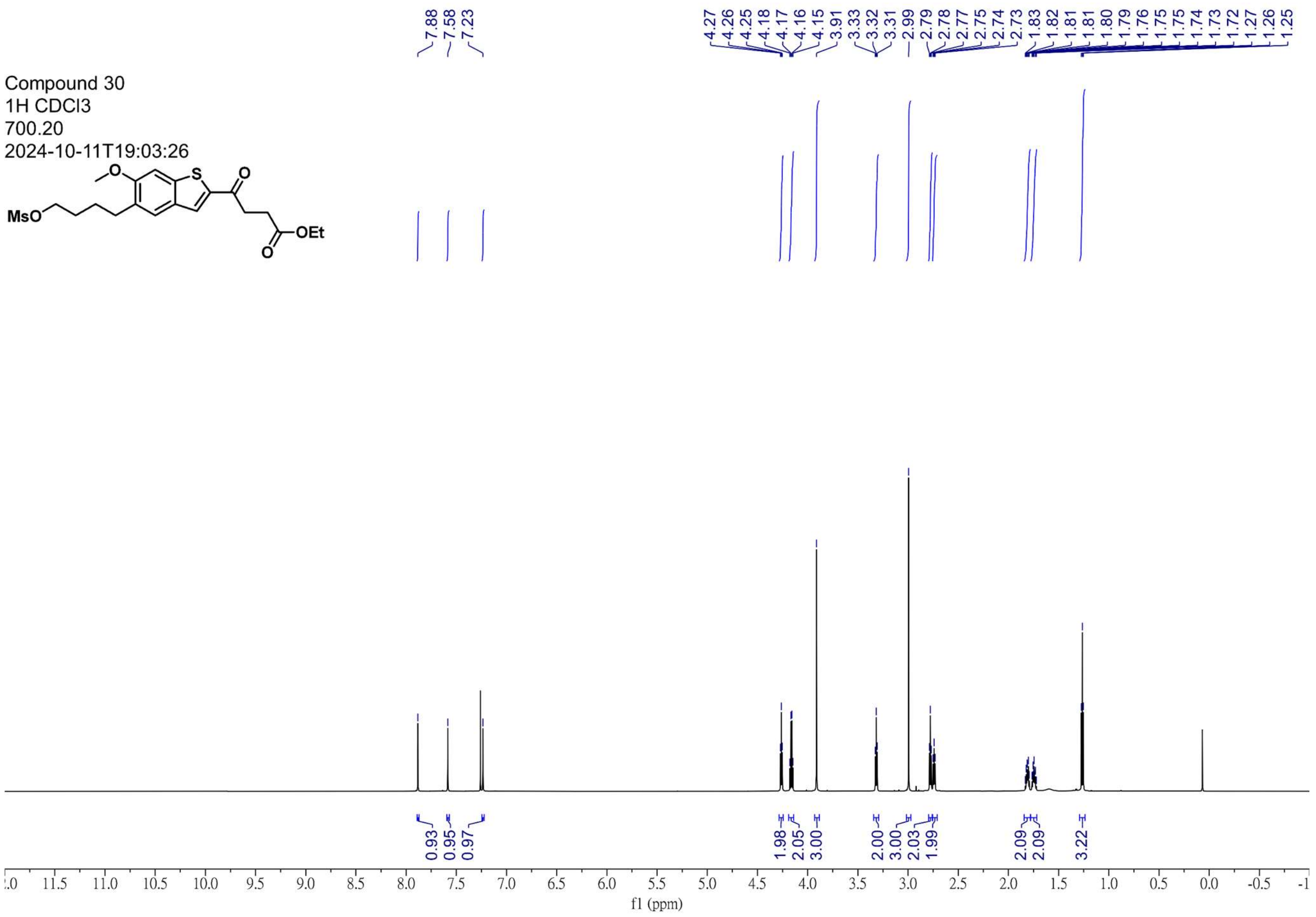
Compound 30
1H CDCl3
700.20
2024-10-11T19:03:26
MsO
OEt
7.88
7.58
7.23
4.27
4.26
4.25
4.18
4.17
4.16
4.15
3.91
3.33
3.32
3.31
2.99
2.79
2.78
2.77
2.75
2.74
2.73
1.83
1.82
1.81
1.81
1.80
1.79
1.76
1.75
1.75
1.74
1.73
1.72
1.27
1.26
1.25
0.93
0.95
0.97
1.98
2.05
3.00
2.00
3.00
2.03
1.99
2.09
2.09
3.22
f1 (ppm)

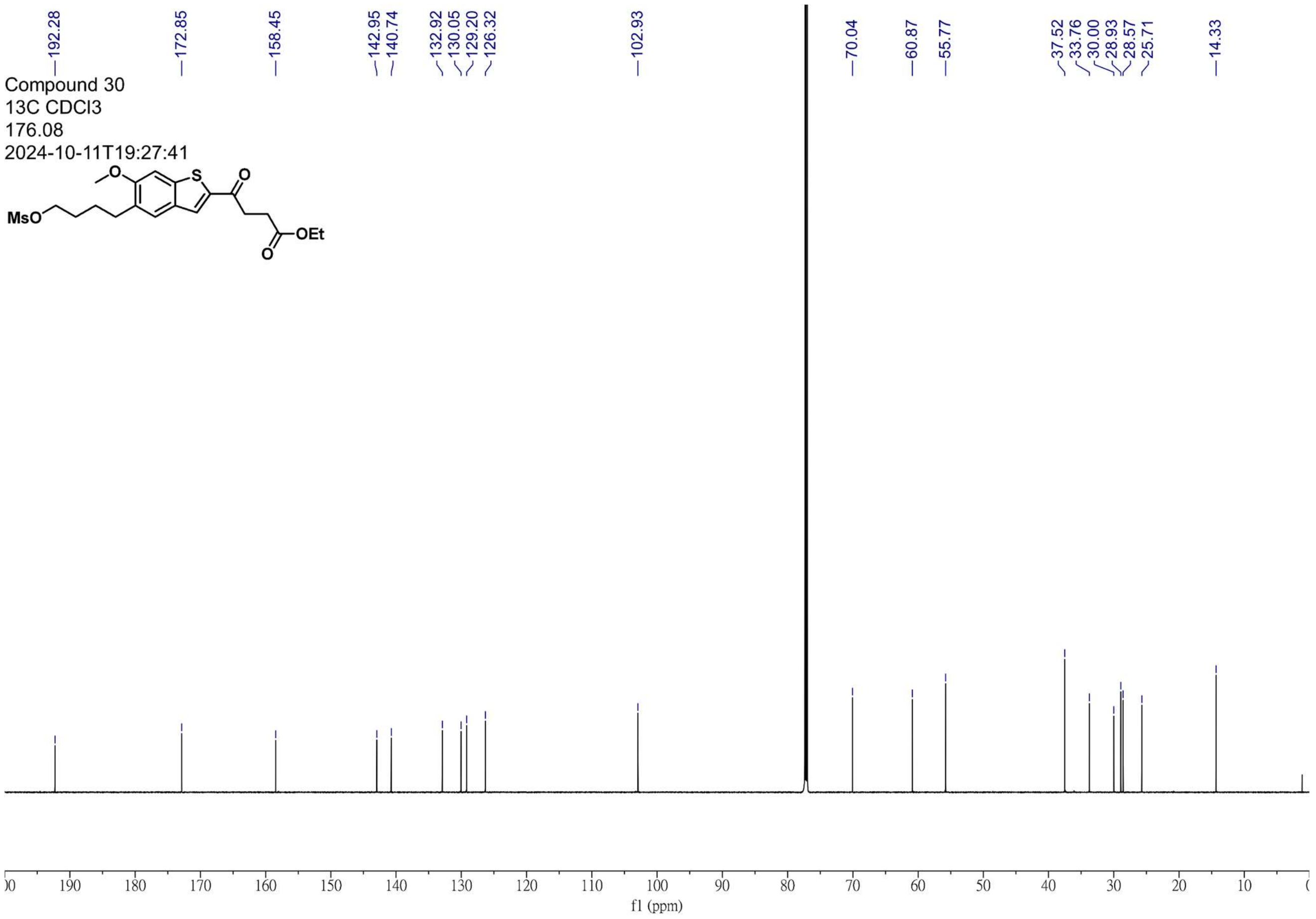
Compound 30
13C CDCl3
176.08
2024-10-11T19:27:41
MsO
OEt
192.28
172.85
158.45
142.95
140.74
132.92
130.05
129.20
126.32
102.93
70.04
60.87
55.77
37.52
33.76
30.00
28.93
28.57
25.71
14.33
f1 (ppm)

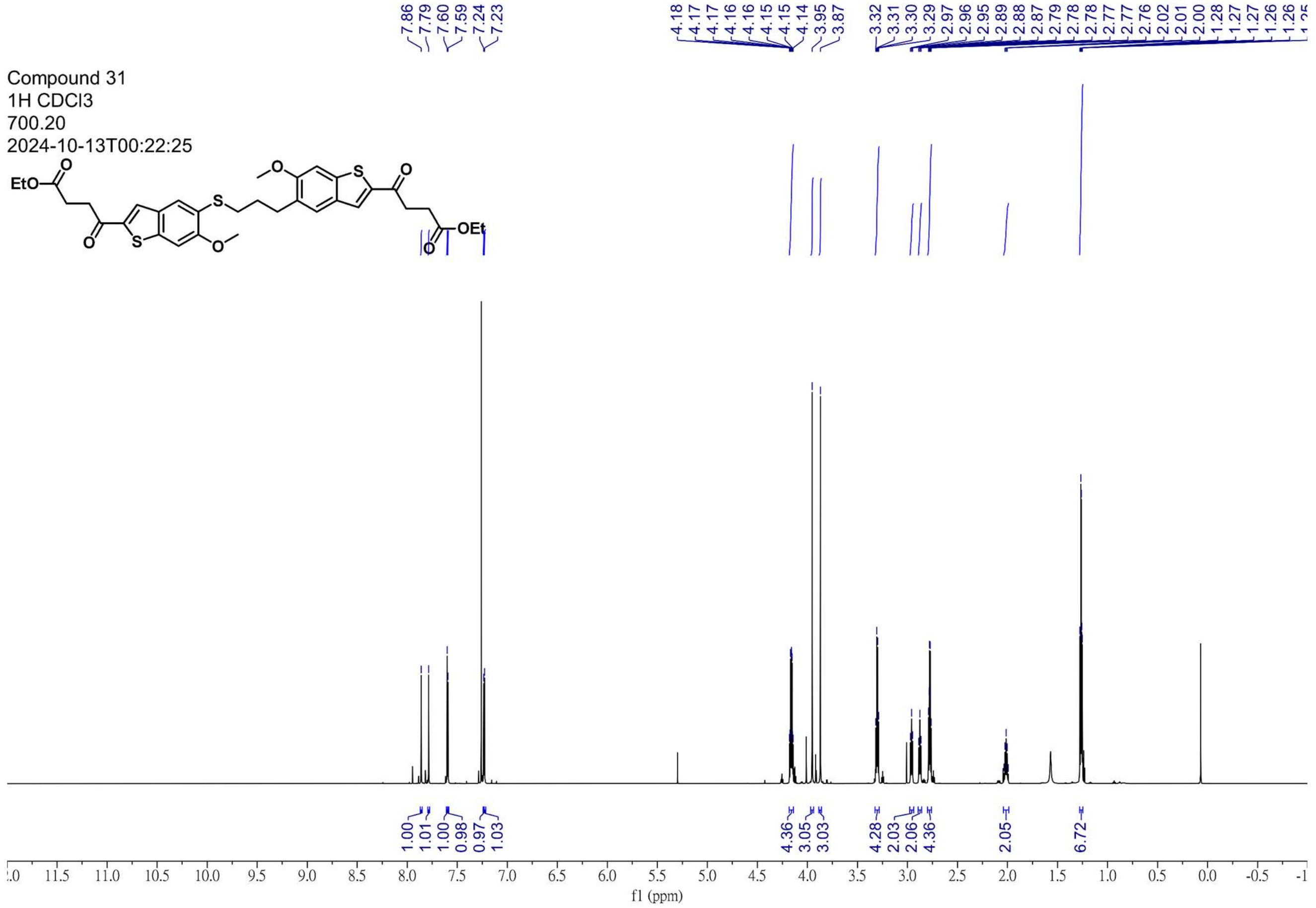
Compound 31
1H CDCl3
700.20
2024-10-13T00:22:25
EtO
S
O
OEt
f1 (ppm)

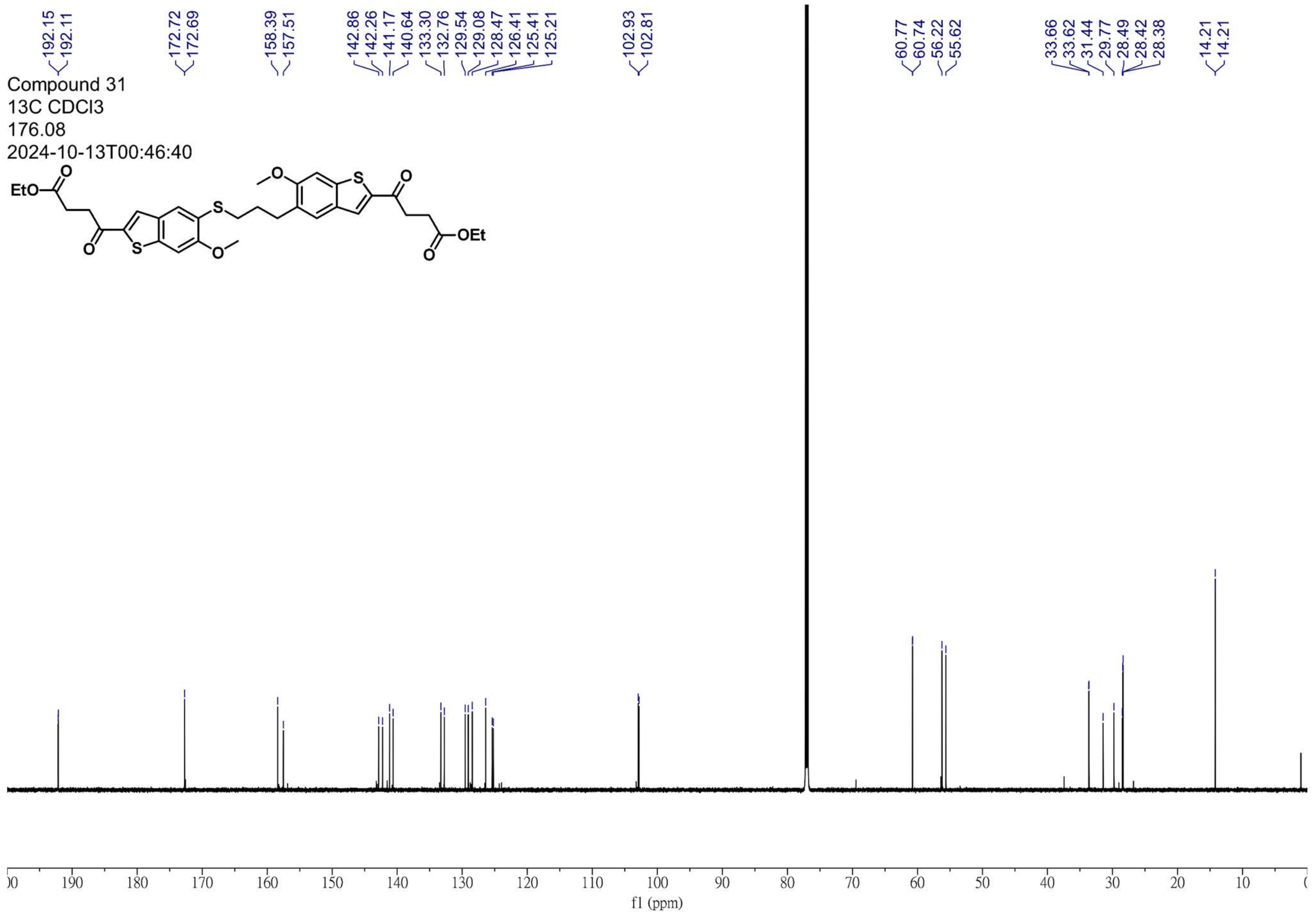
Compound 31
13C CDCl3
176.08
2024-10-13T00:46:40
EtO
S
O
OEt
f1 (ppm)

Compound 32
1H CDCl3
700.20
2024-10-16T00:21:18

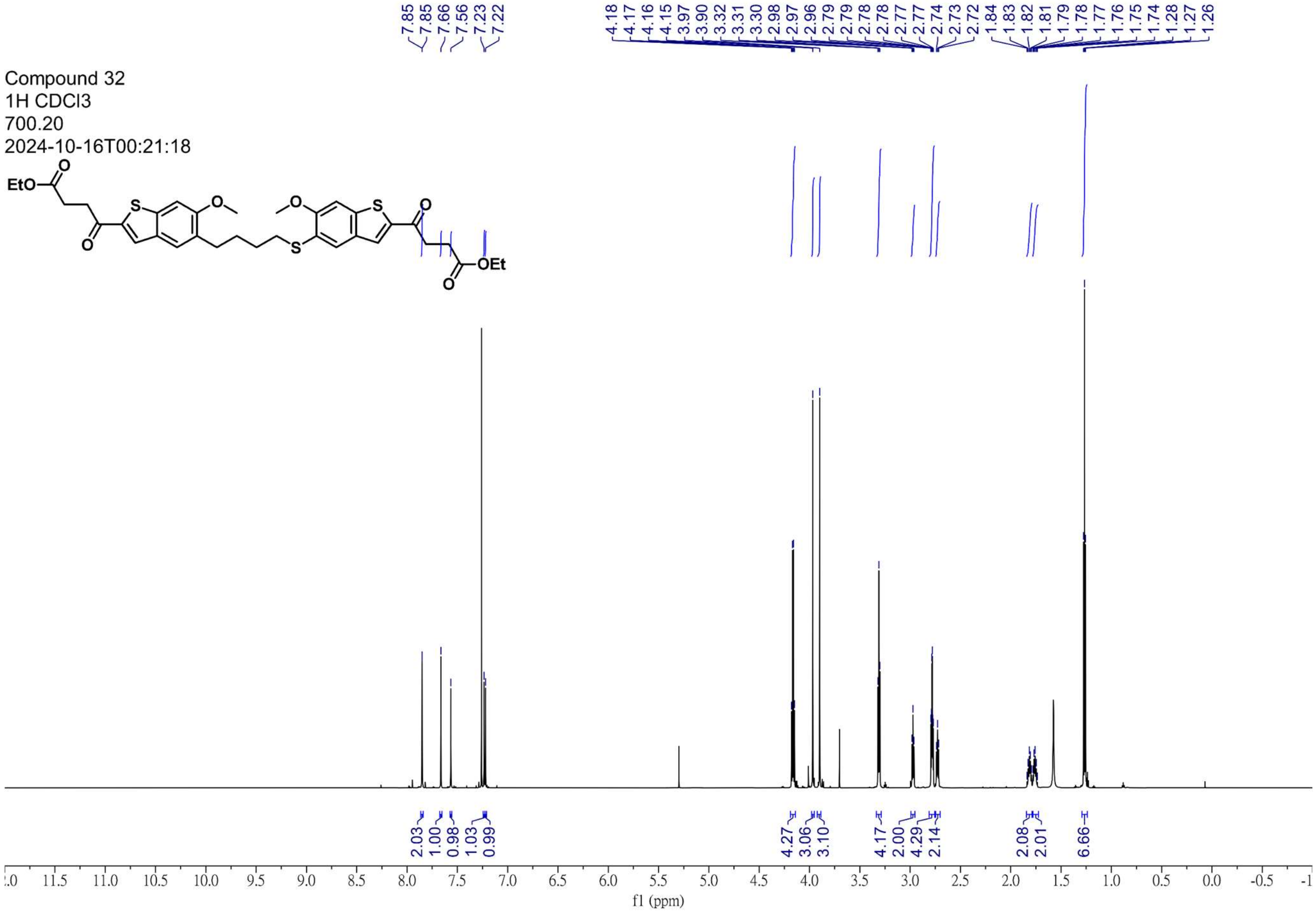

Compound 32
13C CDCl3
176.08
2024-10-16T00:45:34

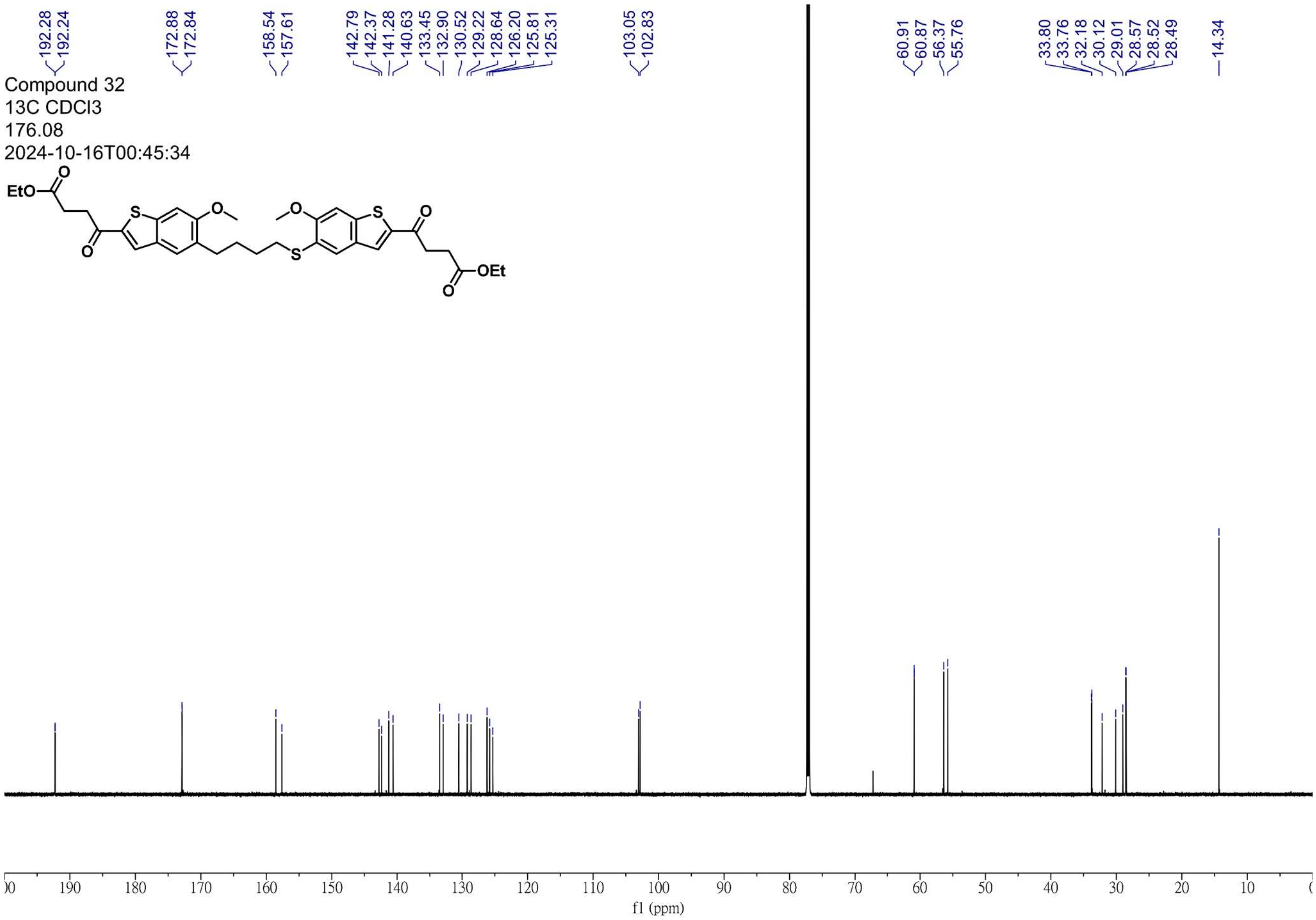

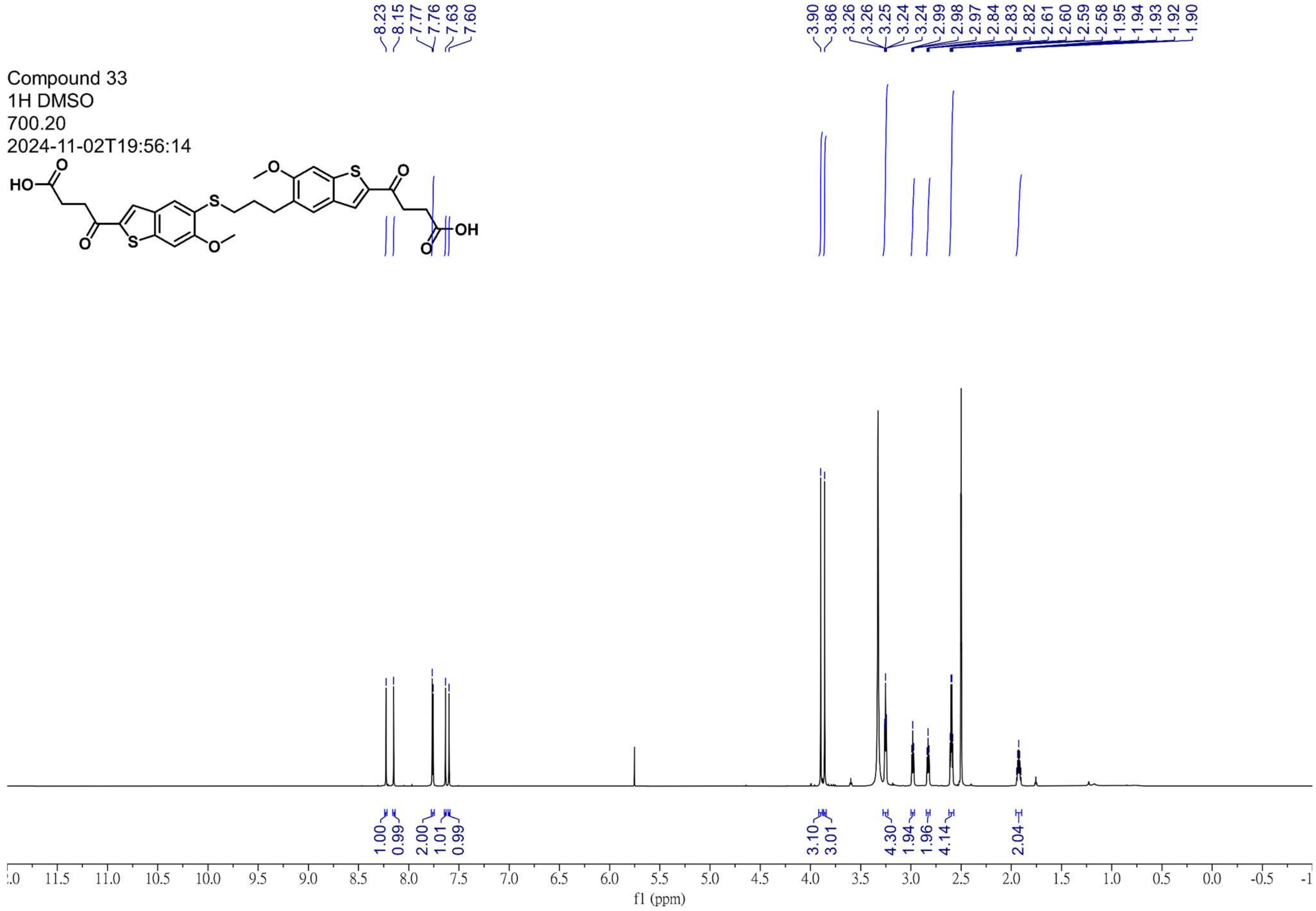
Compound 33
1H DMSO
700.20
2024-11-02T19:56:14
8.23
8.15
7.77
7.76
7.63
7.60
3.90
3.86
3.26
3.25
3.24
3.24
2.99
2.98
2.97
2.84
2.83
2.82
2.61
2.60
2.59
2.58
1.95
1.94
1.93
1.92
1.90
1.00
0.99
2.00
1.01
0.99
3.10
3.01
4.30
1.94
1.96
4.14
2.04
f1 (ppm)

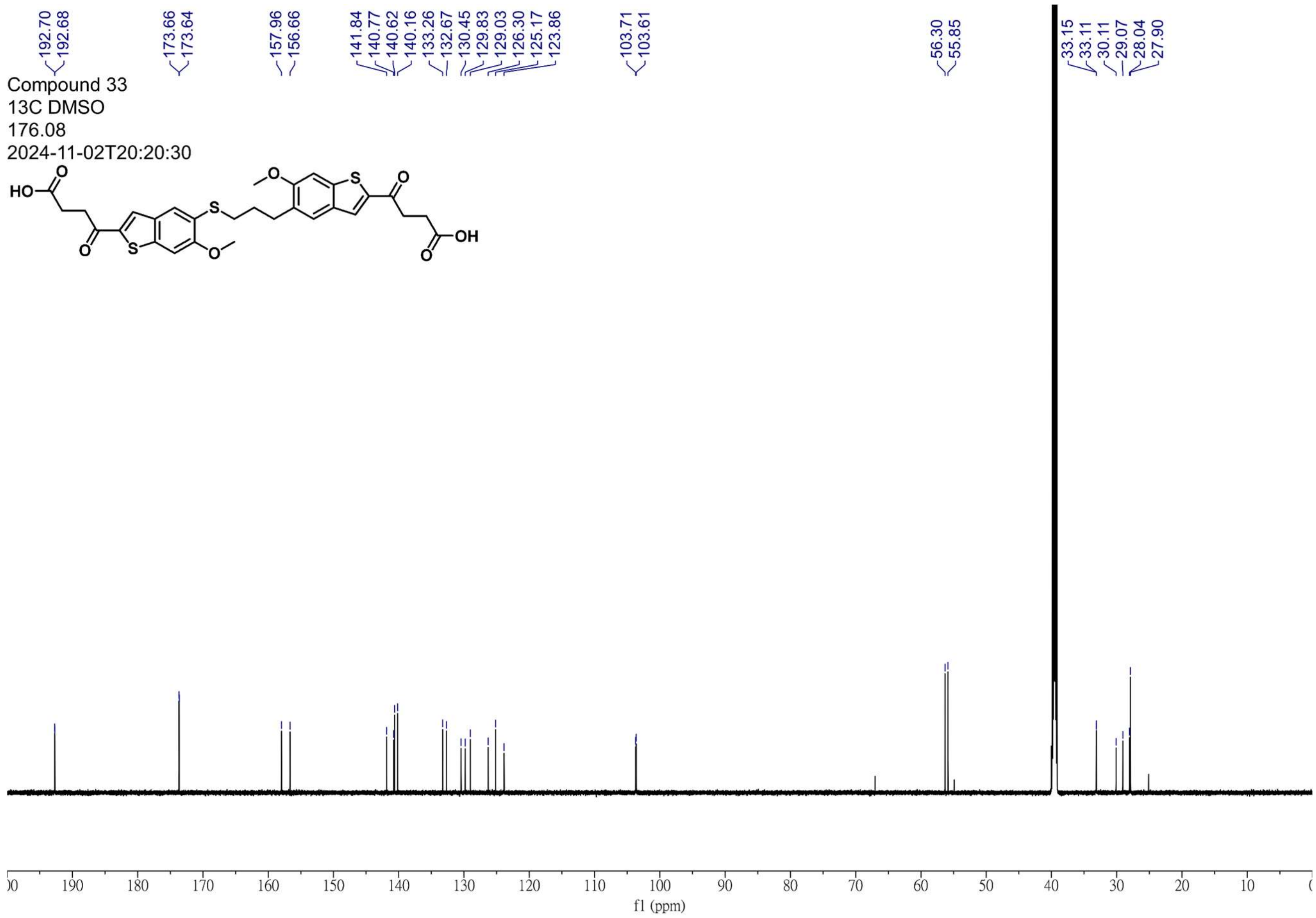
192.70
192.68
173.66
173.64
157.96
156.66
141.84
140.77
140.62
140.16
133.26
132.67
130.45
129.83
129.03
126.30
125.17
123.86
103.71
103.61
56.30
55.85
33.15
33.11
30.11
29.07
28.04
27.90
Compound 33
13C DMSO
176.08
2024-11-02T20:20:30
f1 (ppm)

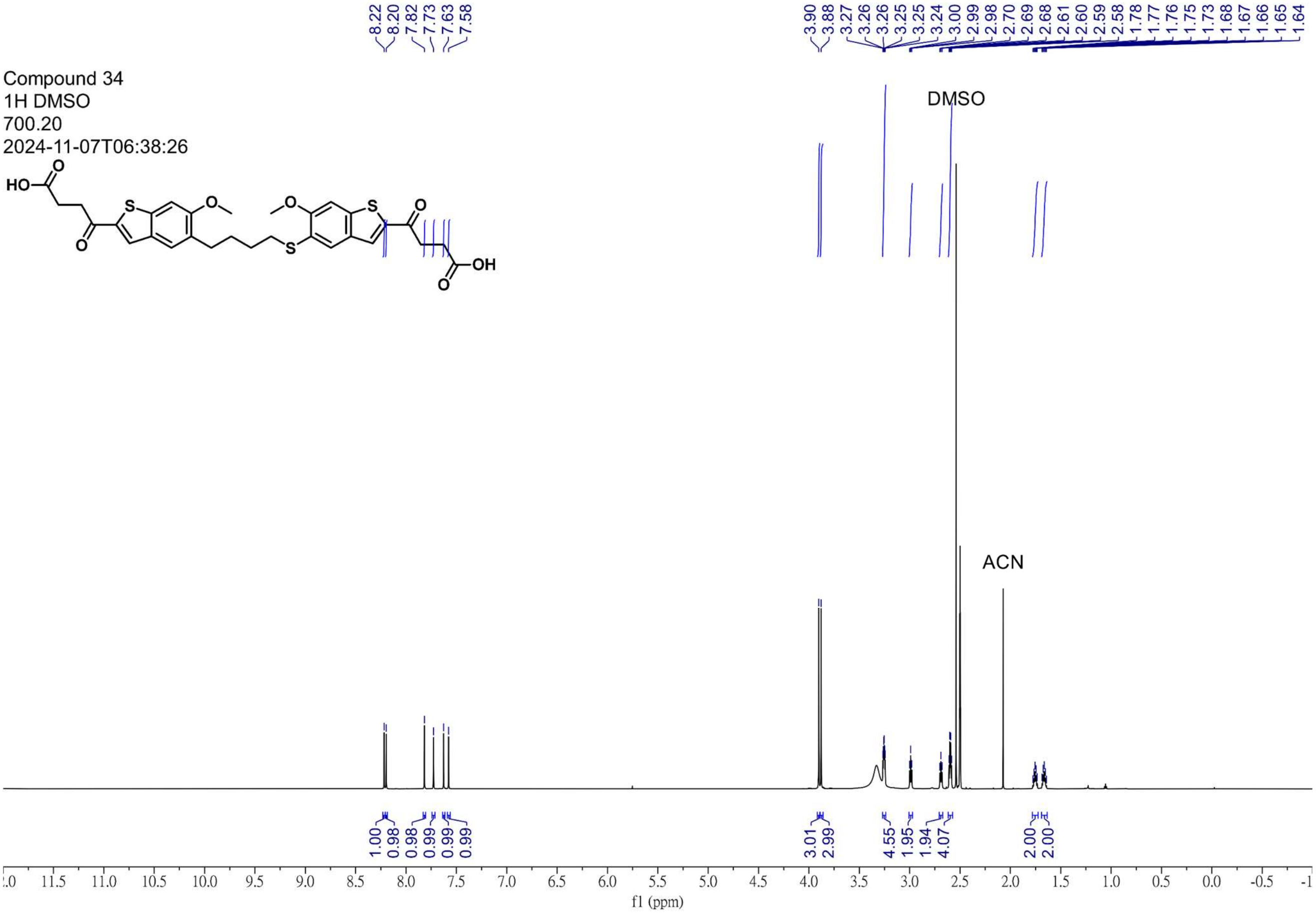
Compound 34
1H DMSO
700.20
2024-11-07T06:38:26
HO
O
S
O
S
S
O
O
OH
8.22
8.20
7.82
7.73
7.63
7.58
3.90
3.88
3.27
3.26
3.26
3.25
3.25
3.24
3.00
2.99
2.98
2.70
2.69
2.68
2.61
2.60
2.59
2.58
1.78
1.77
1.76
1.75
1.73
1.68
1.67
1.66
1.65
1.64
DMSO
ACN
1.00
0.98
0.98
0.99
0.99
0.99
3.01
2.99
4.55
1.95
1.94
4.07
2.00
2.00
f1 (ppm)

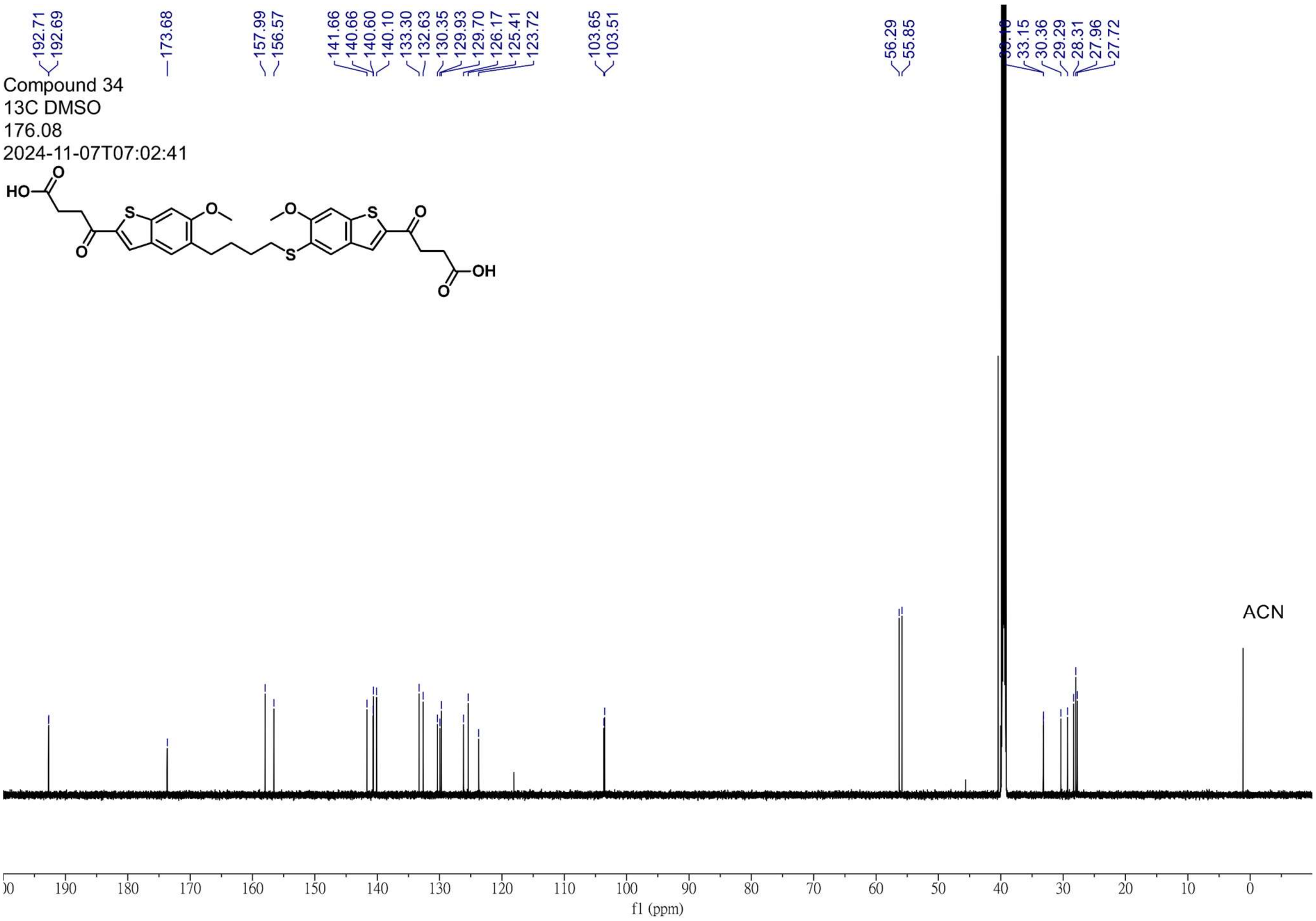
Compound 34
13C DMSO
176.08
2024-11-07T07:02:41
HO
O
S
O
S
S
O
O
OH
192.71
192.69
173.68
157.99
156.57
141.66
140.66
140.60
140.10
133.30
132.63
130.35
129.93
129.70
126.17
125.41
123.72
103.65
103.51
56.29
55.85
[illegible]
33.15
30.36
29.29
28.31
27.96
27.72
ACN
f1 (ppm)

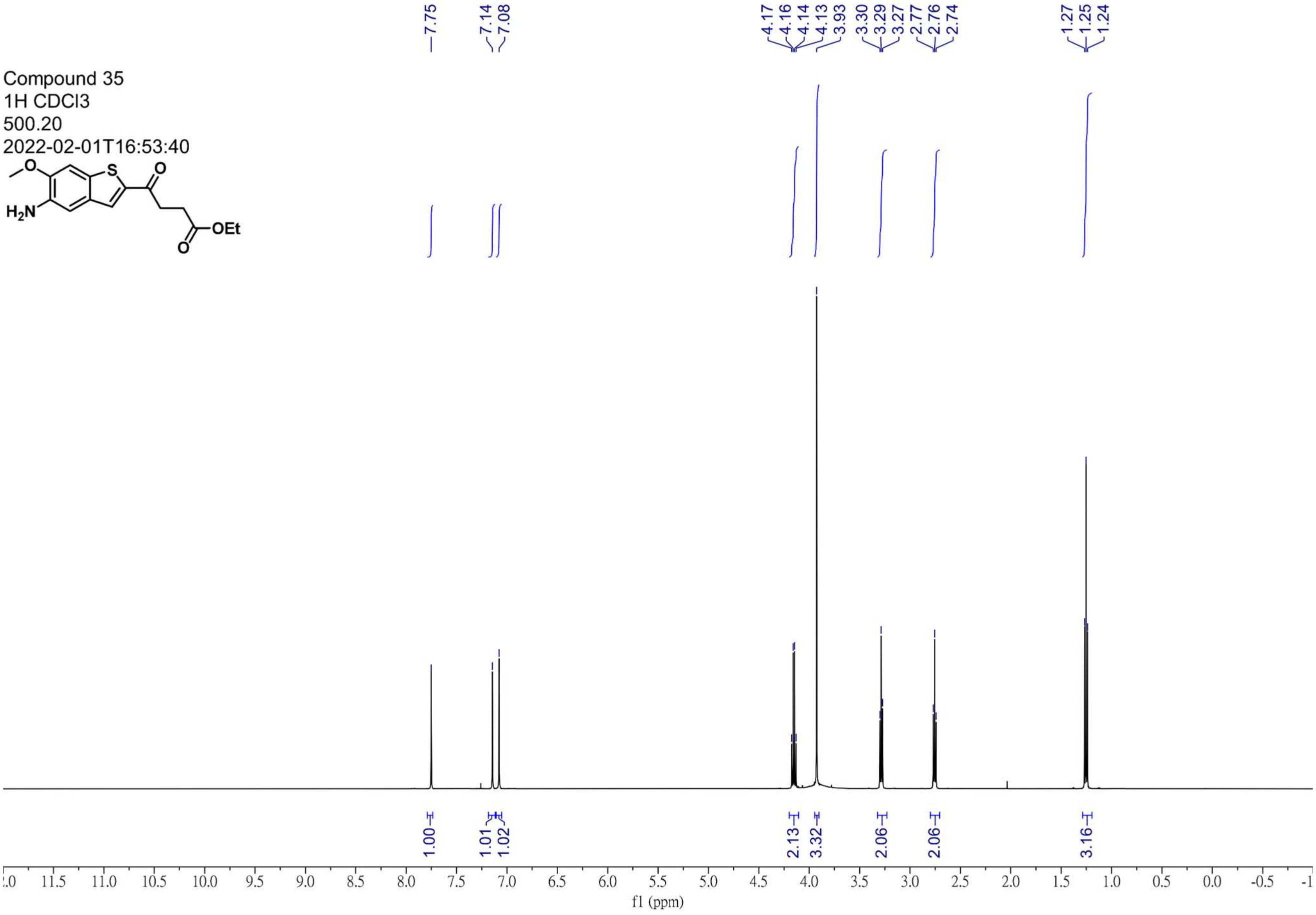
Compound 35
1H CDCl3
500.20
2022-02-01T16:53:40
O
S
O
H2N
OEt
O
7.75
7.14
7.08
4.17
4.16
4.14
4.13
3.93
3.30
3.29
3.27
2.77
2.76
2.74
1.27
1.25
1.24
1.00
1.01
1.02
2.13
3.32
2.06
2.06
3.16
f1 (ppm)

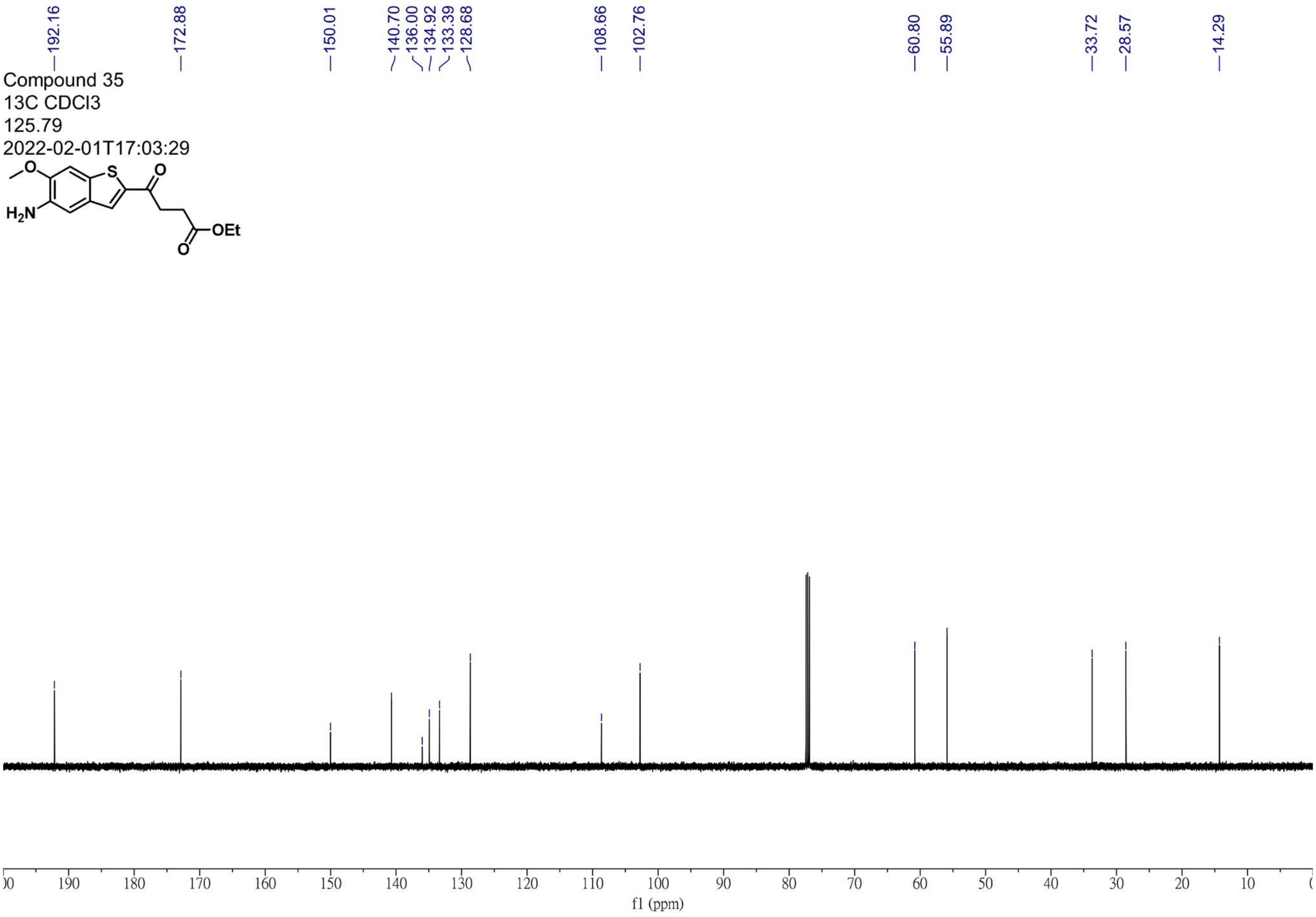
Compound 35
13C CDCl3
125.79
2022-02-01T17:03:29
O
S
O
H2N
OEt
O
192.16
172.88
150.01
140.70
136.00
134.92
133.39
128.68
108.66
102.76
60.80
55.89
33.72
28.57
14.29
f1 (ppm)

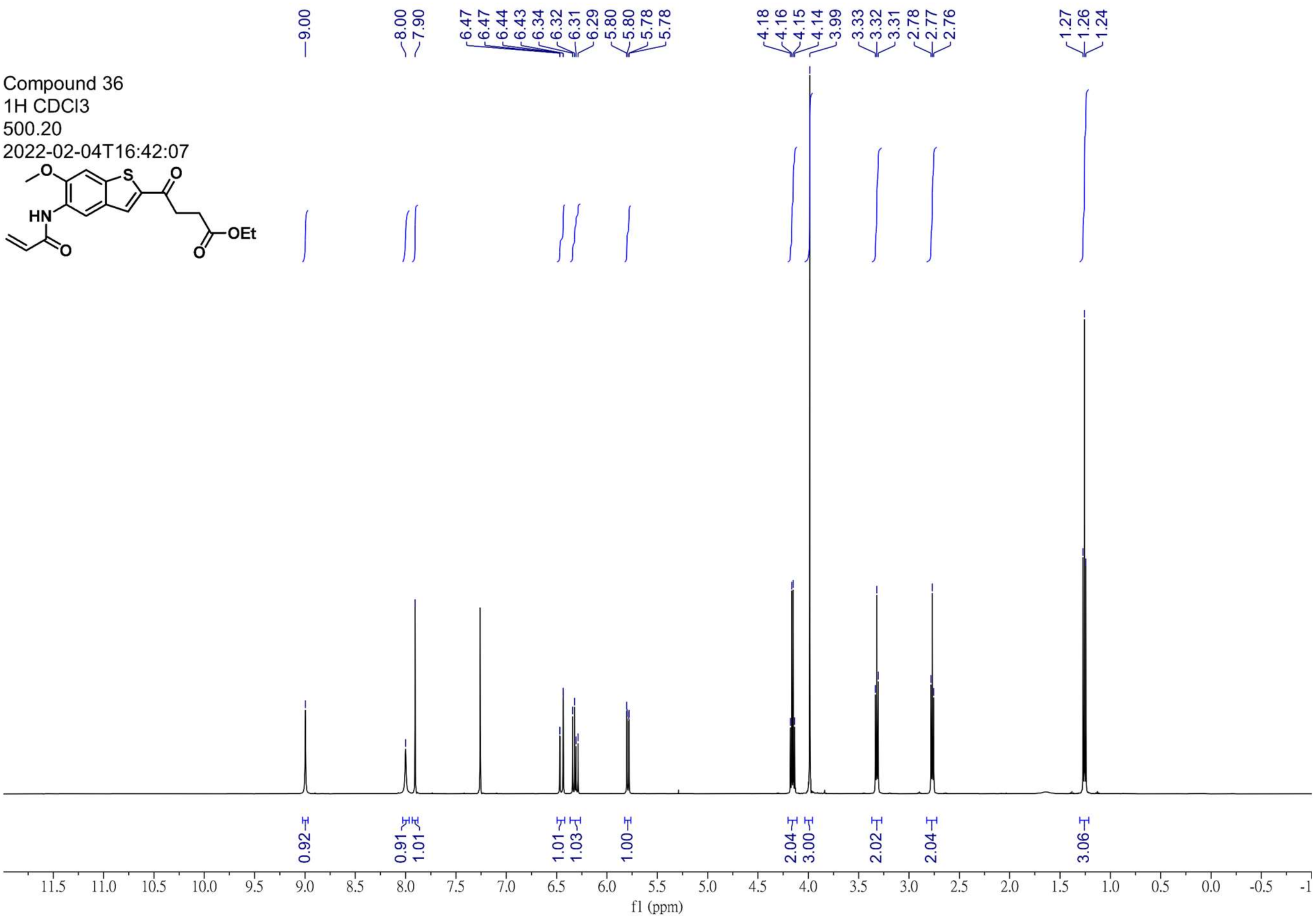

Compound 36
1H CDCl3
500.20
2022-02-04T16:42:07
f1 (ppm)

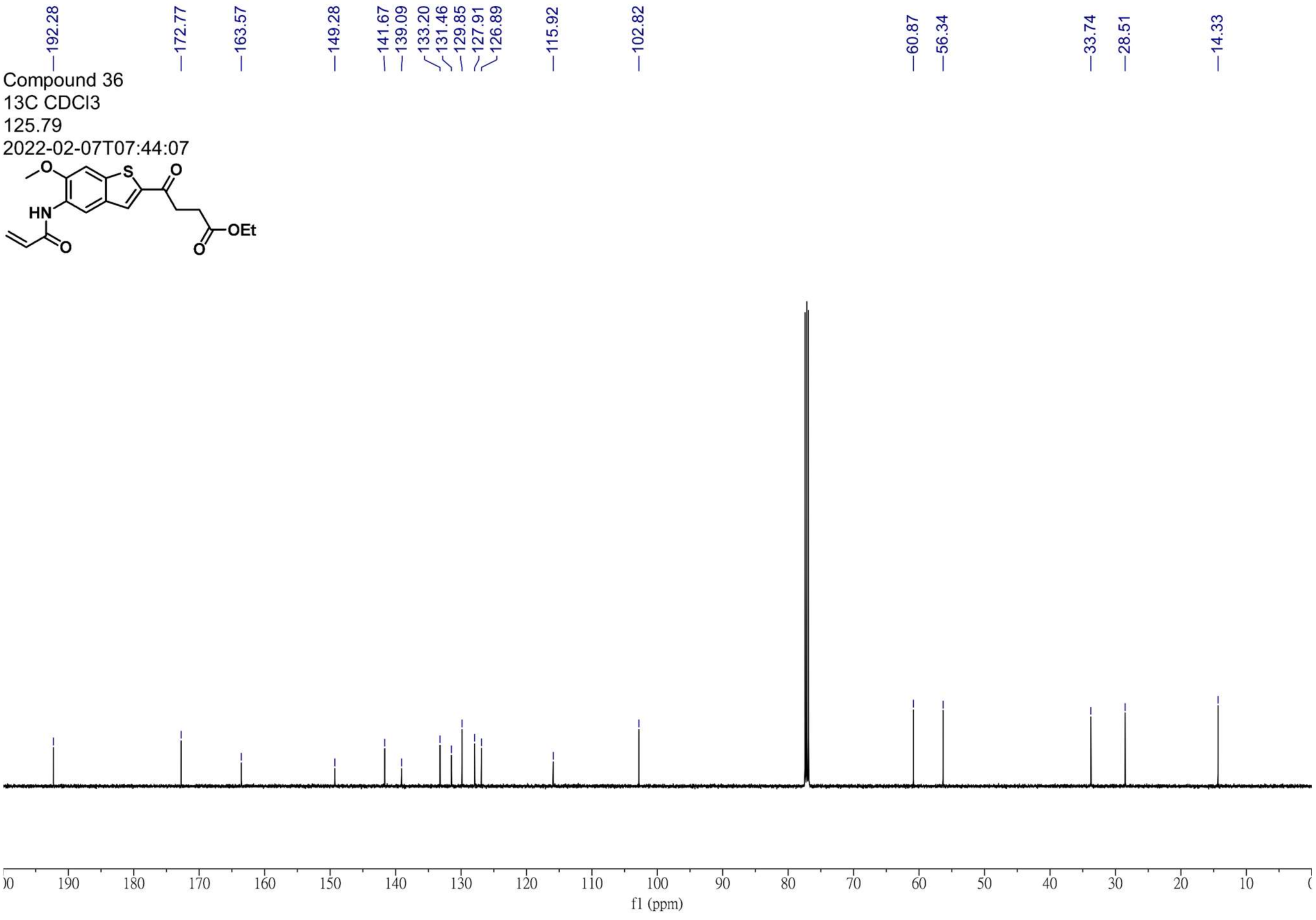

Compound 36
13C CDCl3
125.79
2022-02-07T07:44:07
f1 (ppm)

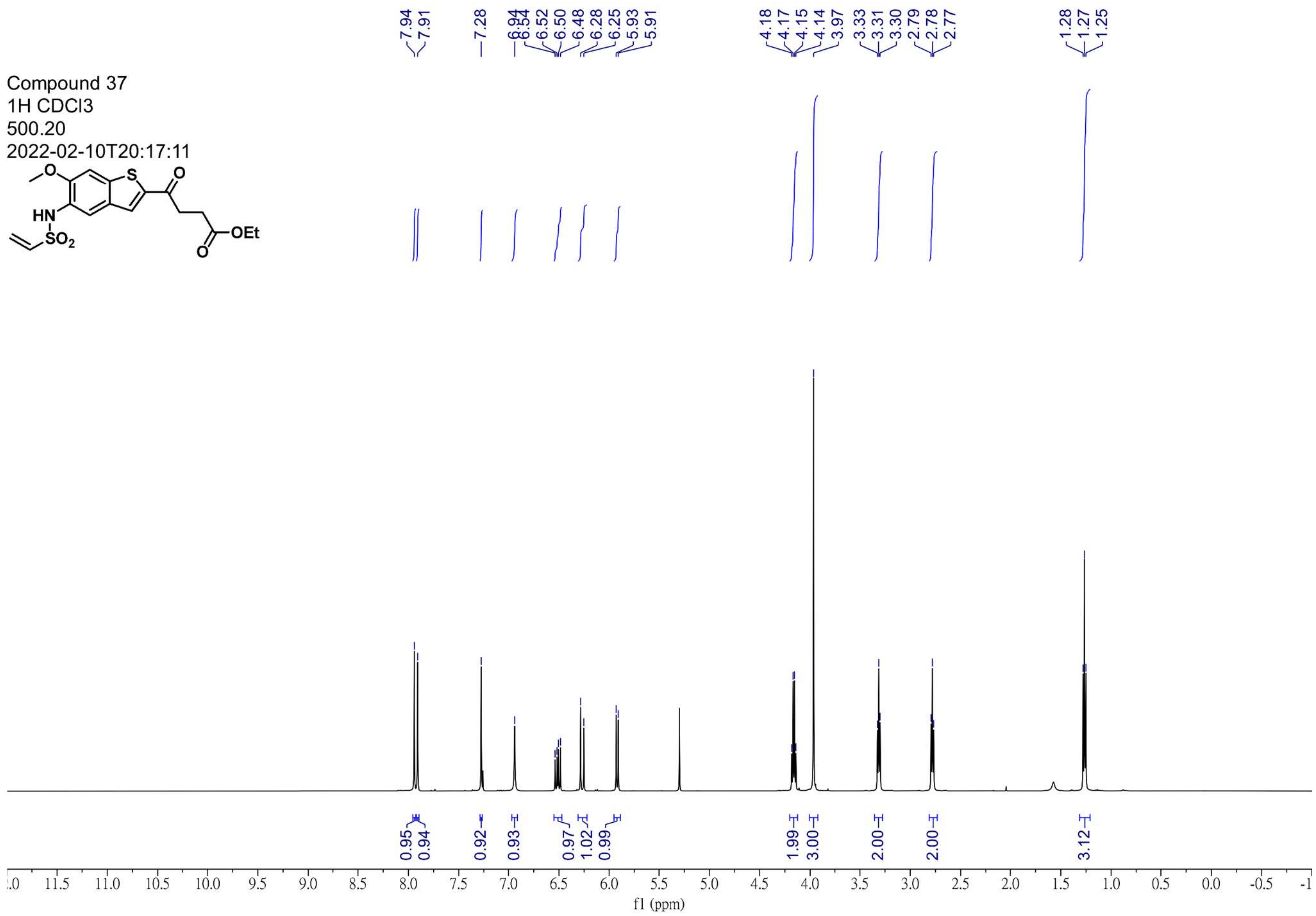
Compound 37
1H CDCl3
500.20
2022-02-10T20:17:11
7.94
7.91
7.28
6.94
6.54
6.52
6.50
6.48
6.28
6.25
5.93
5.91
4.18
4.17
4.15
4.14
3.97
3.33
3.31
3.30
2.79
2.78
2.77
1.28
1.27
1.25
0.95
0.94
0.92
0.93
0.97
1.02
0.99
1.99
3.00
2.00
2.00
3.12
f1 (ppm)

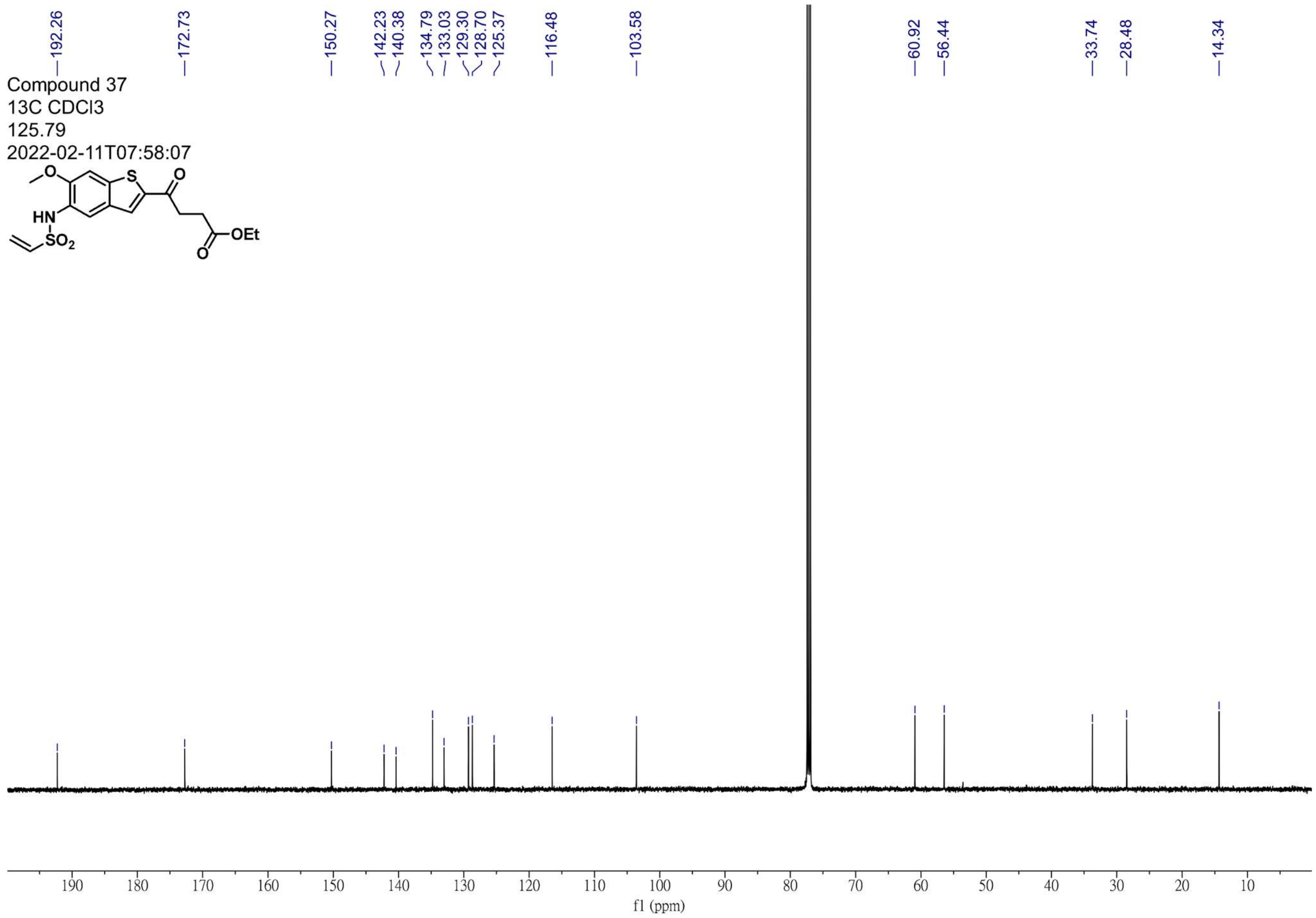
Compound 37
13C CDCl3
125.79
2022-02-11T07:58:07
192.26
172.73
150.27
142.23
140.38
134.79
133.03
129.30
128.70
125.37
116.48
103.58
60.92
56.44
33.74
28.48
14.34
f1 (ppm)

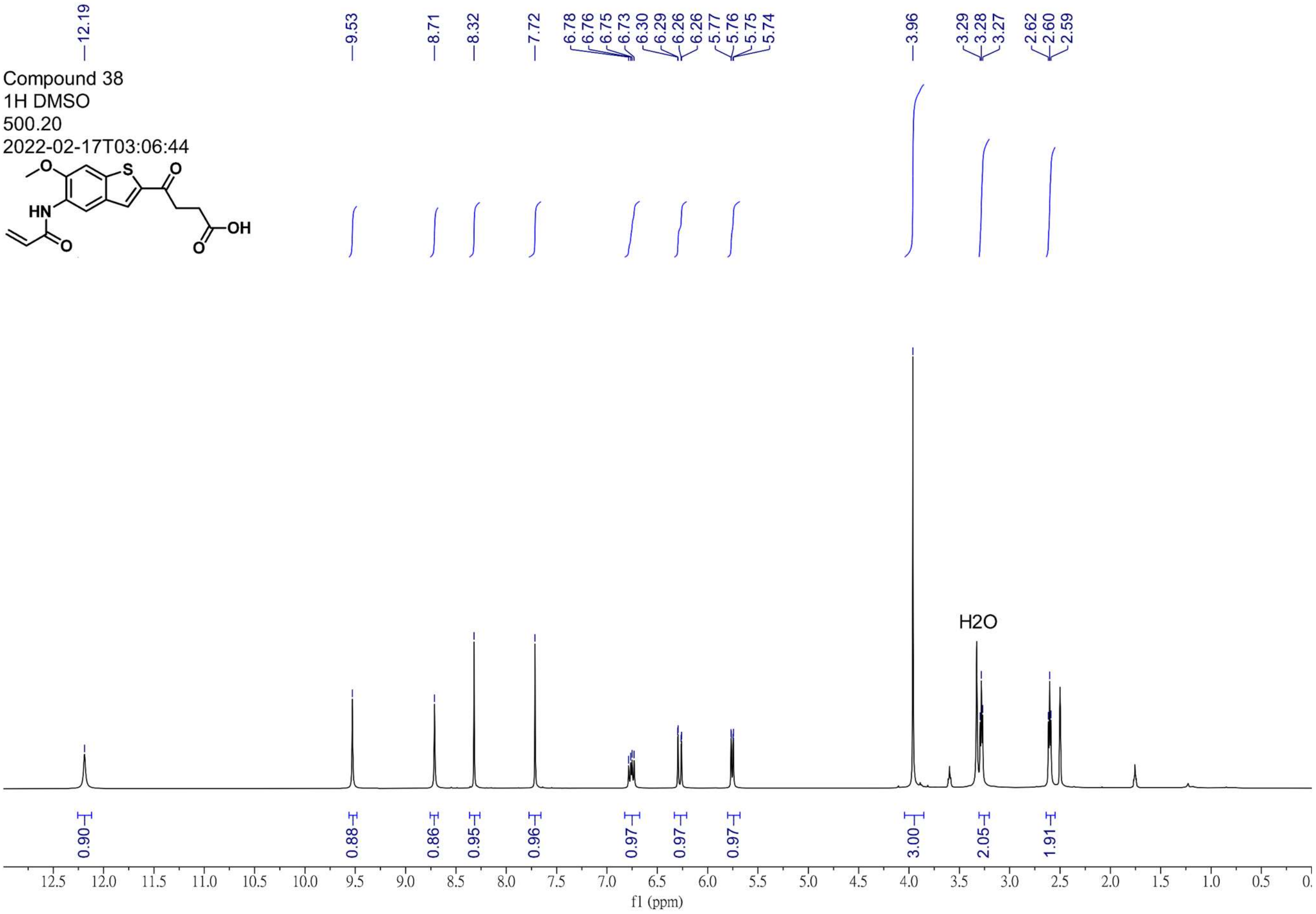
12.19
9.53
8.71
8.32
7.72
6.78
6.76
6.75
6.73
6.30
6.29
6.26
6.26
5.77
5.76
5.75
5.74
3.96
3.29
3.28
3.27
2.62
2.60
2.59
Compound 38
1H DMSO
500.20
2022-02-17T03:06:44
HN
O
S
OH
H2O
0.90
0.88
0.86
0.95
0.96
0.97
0.97
0.97
3.00
2.05
1.91
f1 (ppm)

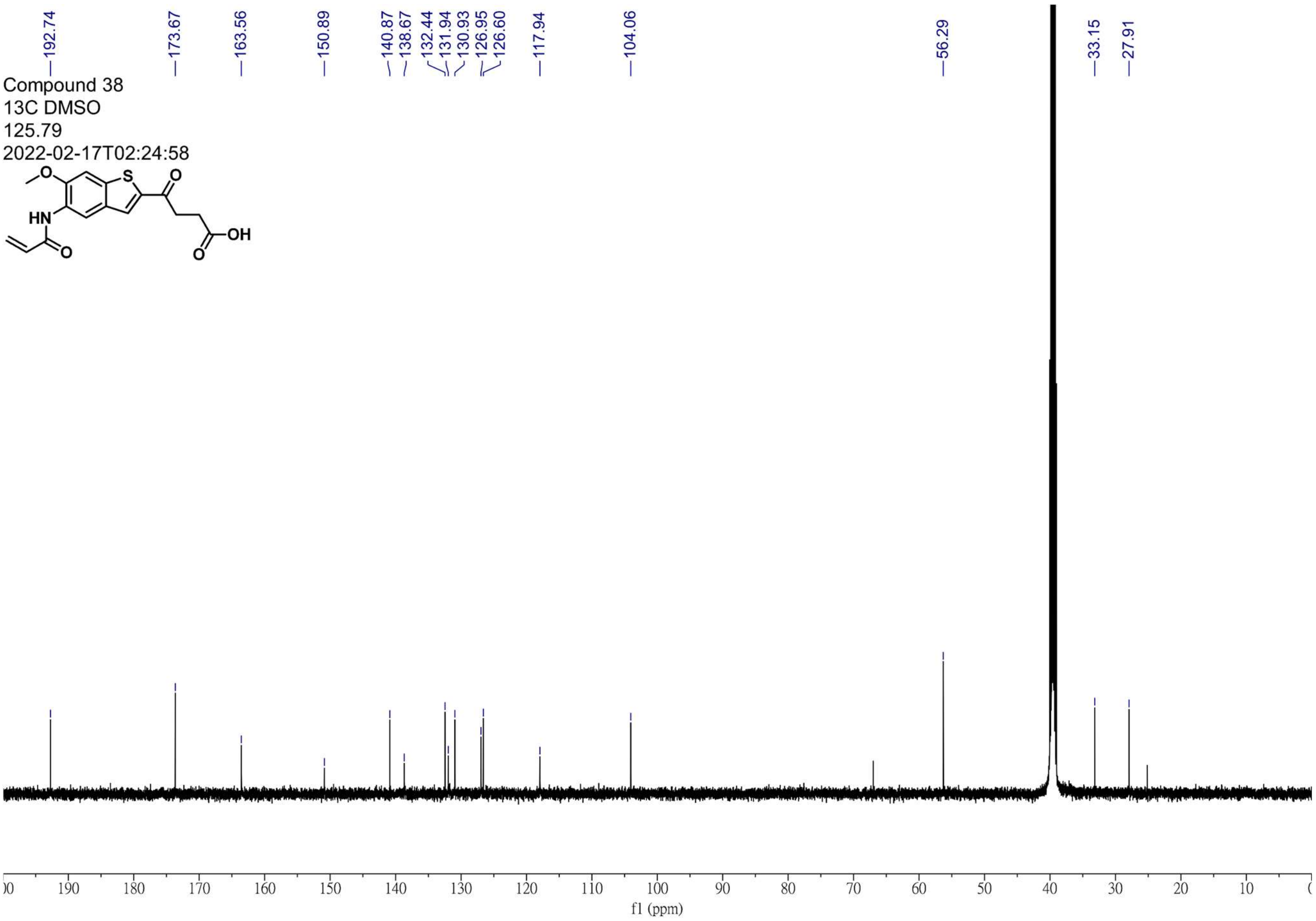
192.74
173.67
163.56
150.89
140.87
138.67
132.44
131.94
130.93
126.95
126.60
117.94
104.06
56.29
33.15
27.91
Compound 38
13C DMSO
125.79
2022-02-17T02:24:58
HN
O
S
OH
f1 (ppm)

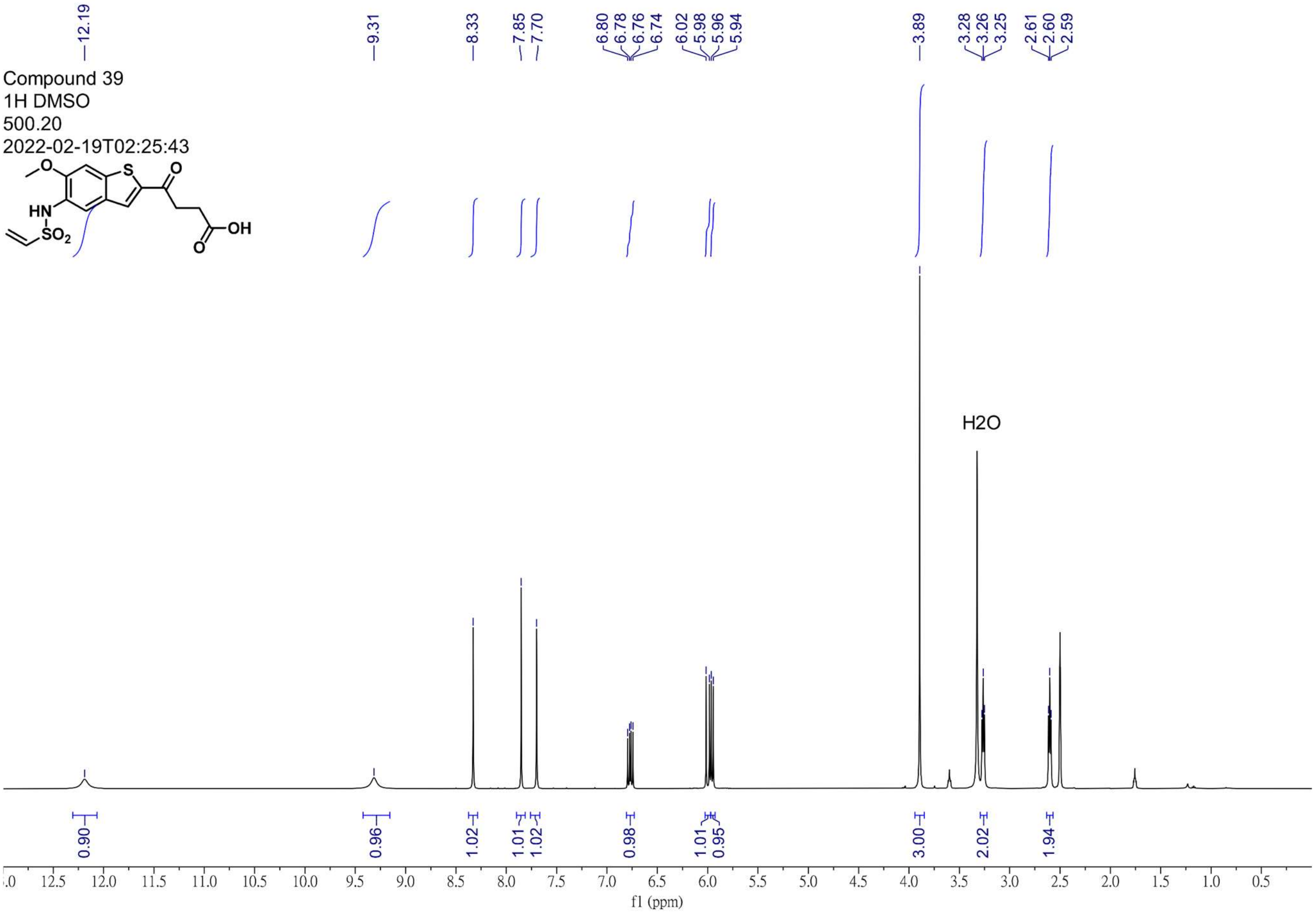
Compound 39
1H DMSO
500.20
2022-02-19T02:25:43
HN
SO2
S
O
OH
O
H2O
12.19
9.31
8.33
7.85
7.70
6.80
6.78
6.76
6.74
6.02
5.98
5.96
5.94
3.89
3.28
3.26
3.25
2.61
2.60
2.59
0.90
0.96
1.02
1.01
1.02
0.98
1.01
0.95
3.00
2.02
1.94
f1 (ppm)

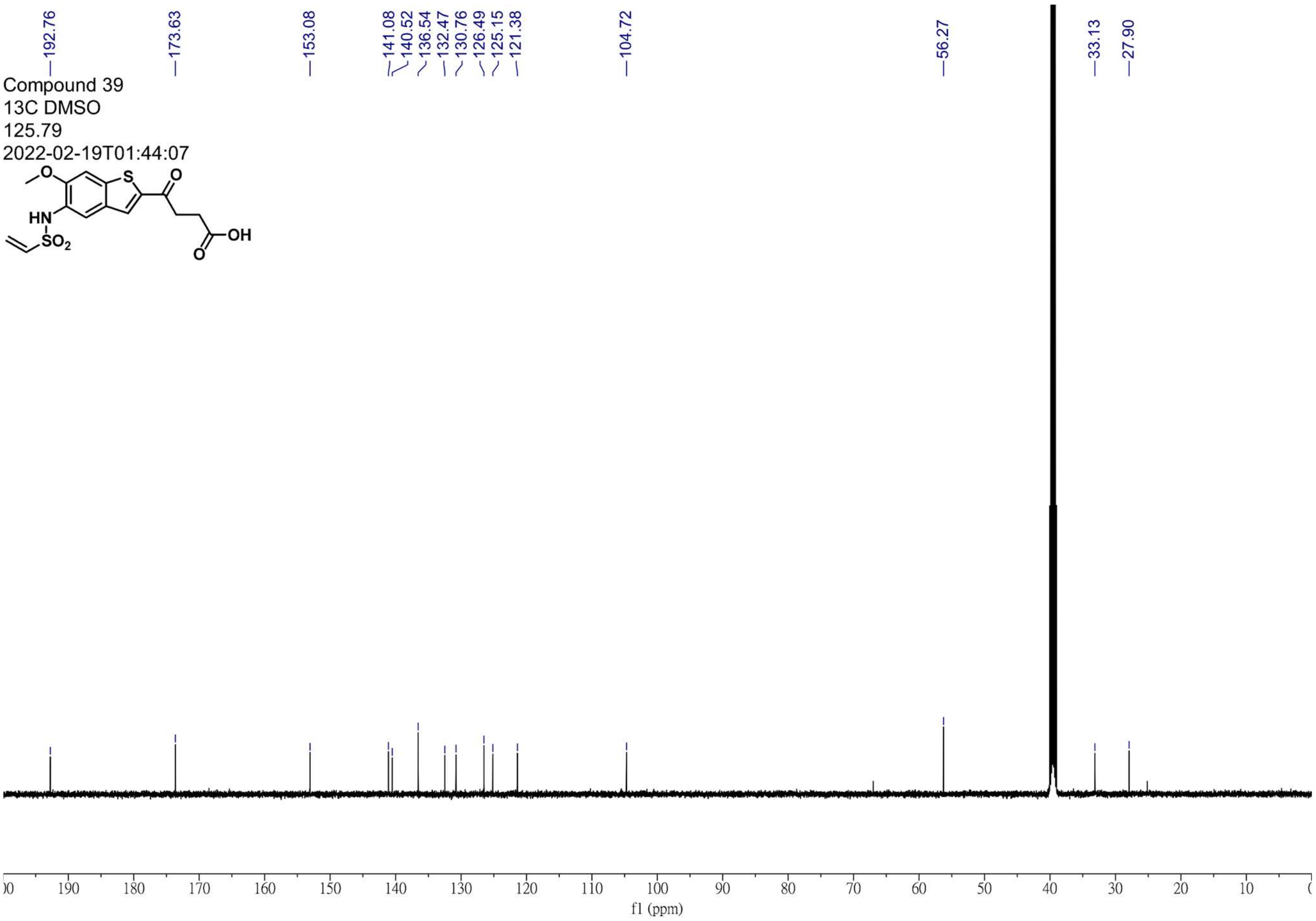
Compound 39
13C DMSO
125.79
2022-02-19T01:44:07
HN
SO2
S
O
OH
O
192.76
173.63
153.08
141.08
140.52
136.54
132.47
130.76
126.49
125.15
121.38
104.72
56.27
33.13
27.90
f1 (ppm)

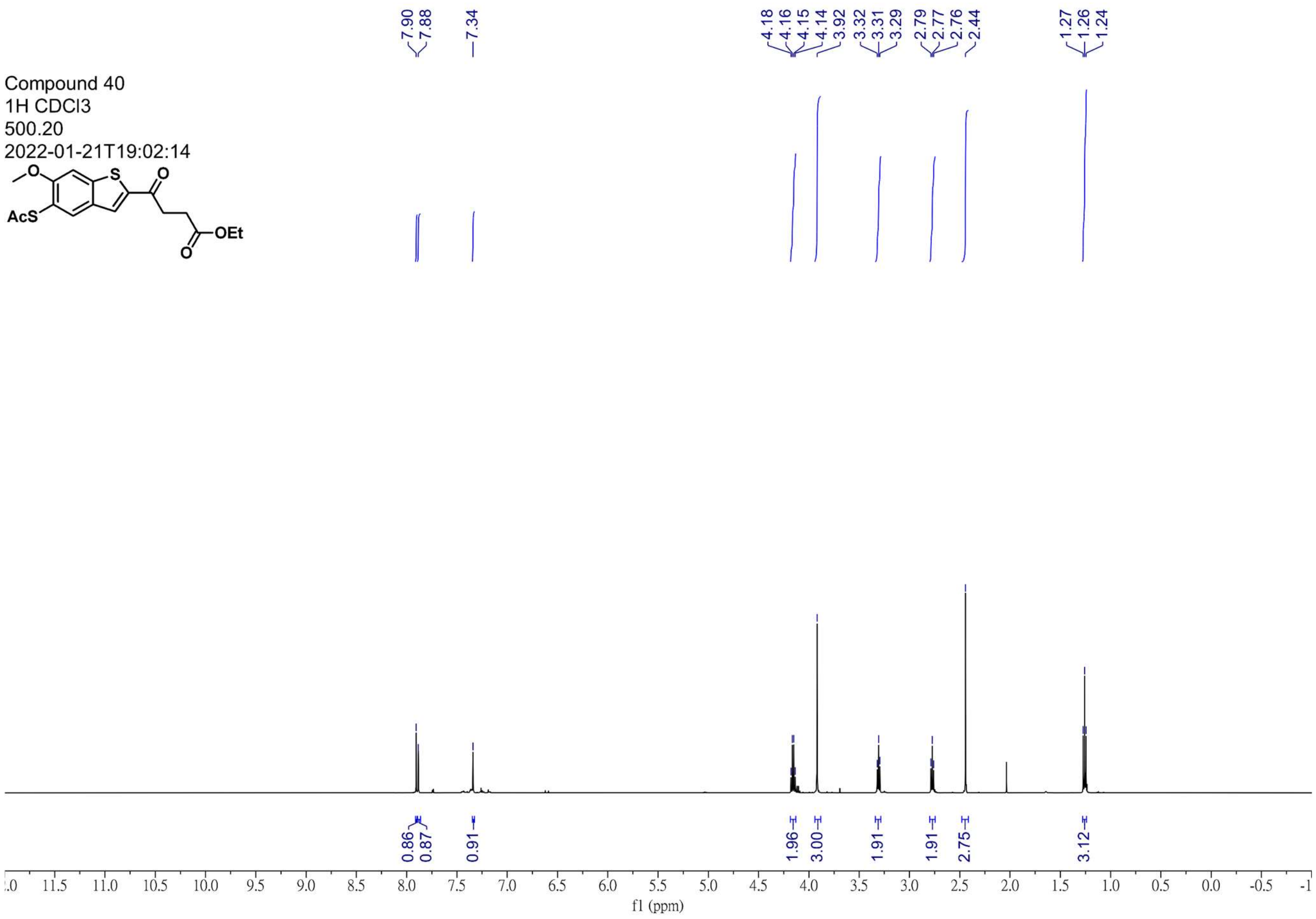
Compound 40
1H CDCl3
500.20
2022-01-21T19:02:14
AcS
OEt
7.90
7.88
7.34
4.18
4.16
4.15
4.14
3.92
3.32
3.31
3.29
2.79
2.77
2.76
2.44
1.27
1.26
1.24
0.86
0.87
0.91
1.96
3.00
1.91
1.91
2.75
3.12
f1 (ppm)

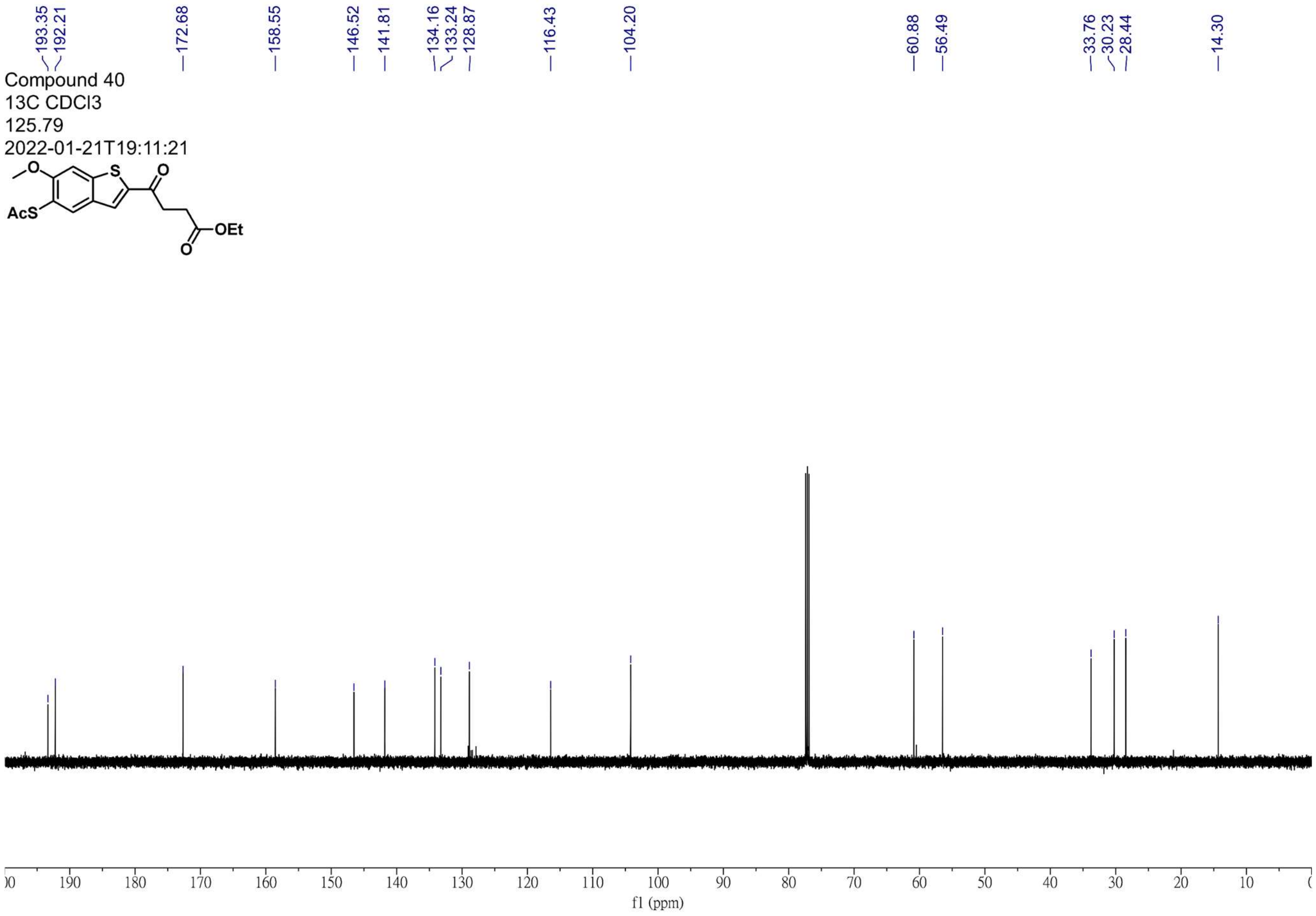
Compound 40
13C CDCl3
125.79
2022-01-21T19:11:21
AcS
OEt
193.35
192.21
172.68
158.55
146.52
141.81
134.16
133.24
128.87
116.43
104.20
60.88
56.49
33.76
30.23
28.44
14.30
f1 (ppm)

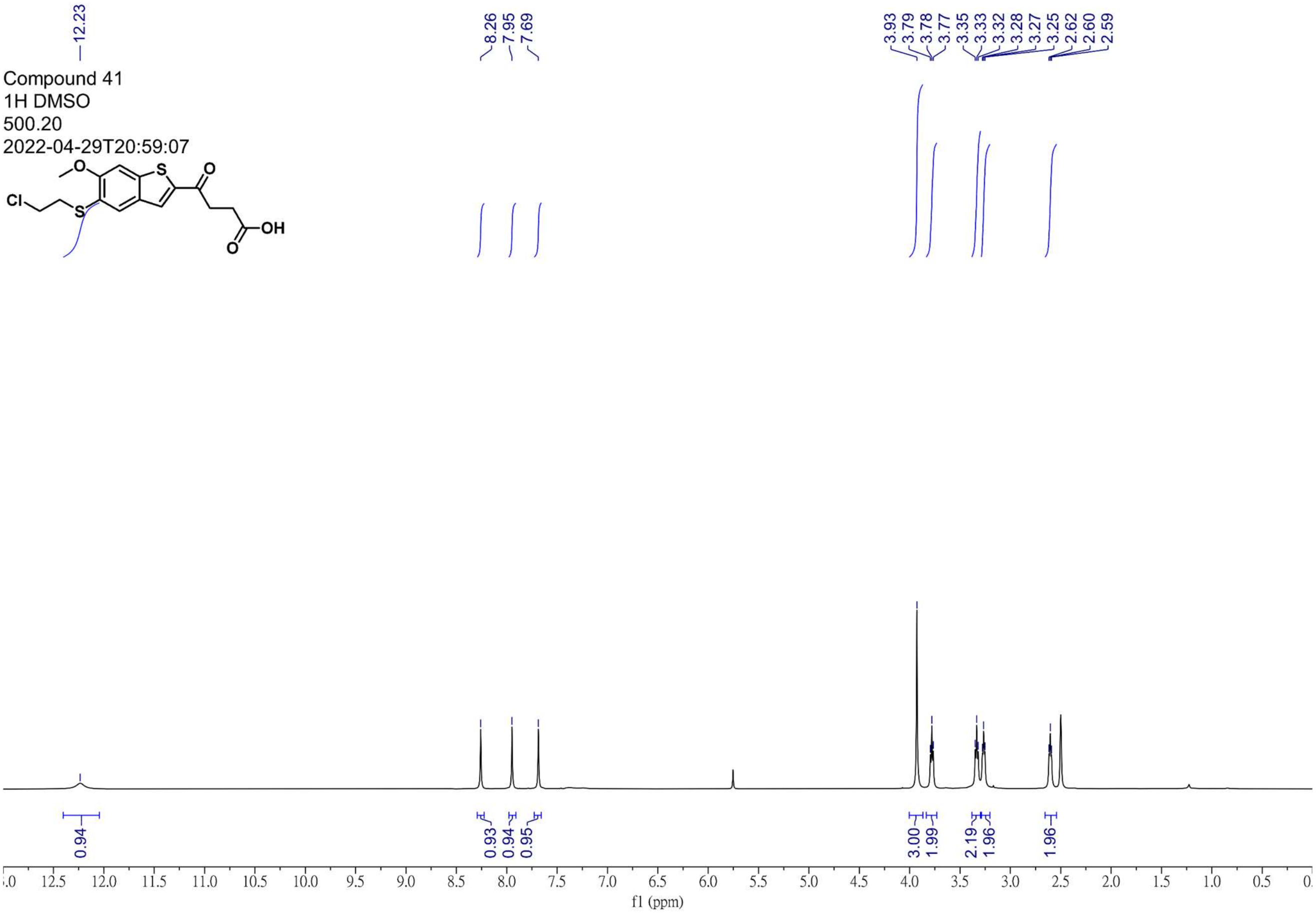
12.23
8.26
7.95
7.69
3.93
3.79
3.78
3.77
3.35
3.33
3.32
3.28
3.27
3.25
2.62
2.60
2.59
Compound 41
1H DMSO
500.20
2022-04-29T20:59:07
Cl
S
O
S
O
OH
O
0.94
0.93
0.94
0.95
3.00
1.99
2.19
1.96
1.96
f1 (ppm)

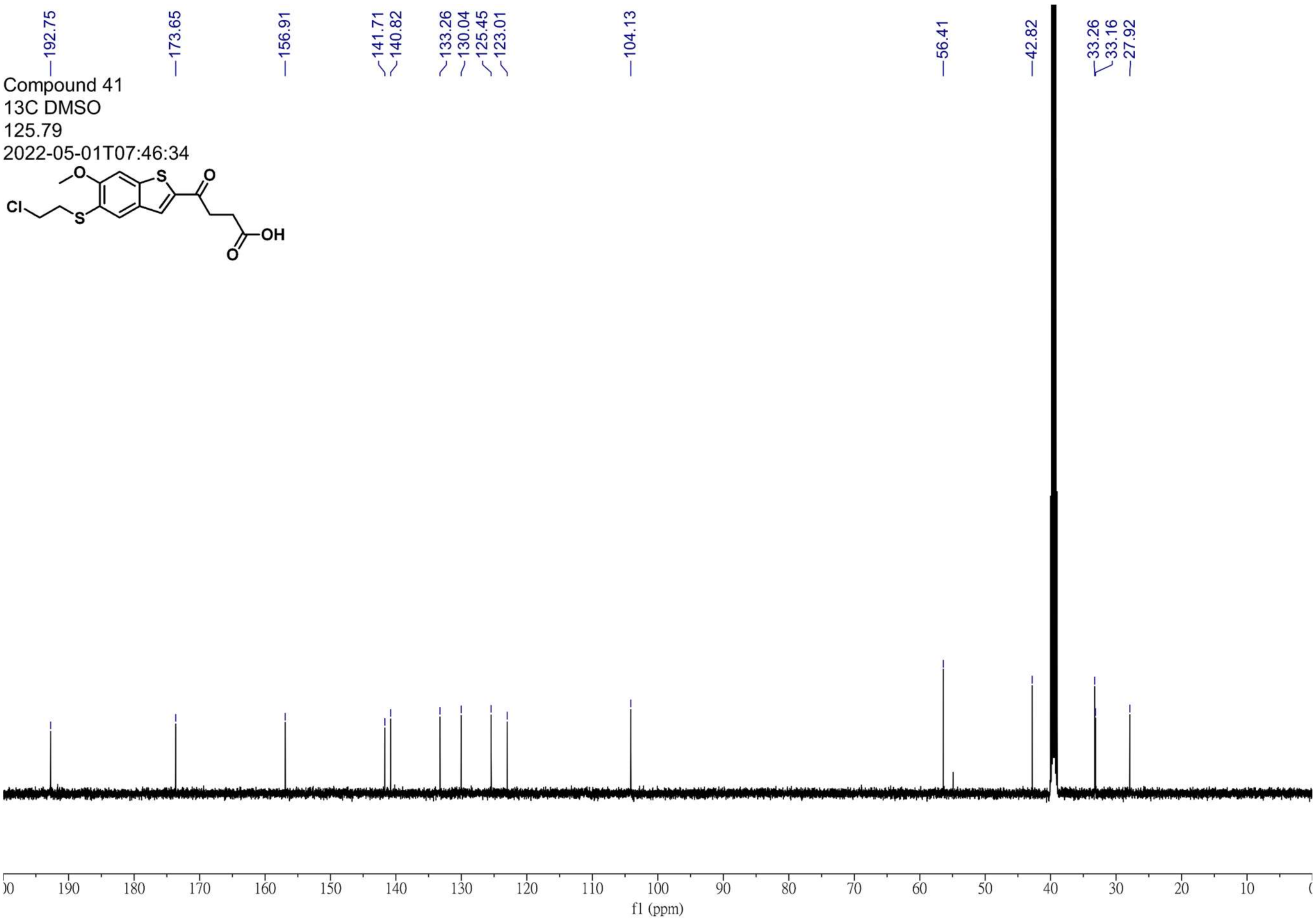
192.75
173.65
156.91
141.71
140.82
133.26
130.04
125.45
123.01
104.13
56.41
42.82
33.26
33.16
27.92
Compound 41
13C DMSO
125.79
2022-05-01T07:46:34
Cl
S
O
S
O
OH
O
f1 (ppm)

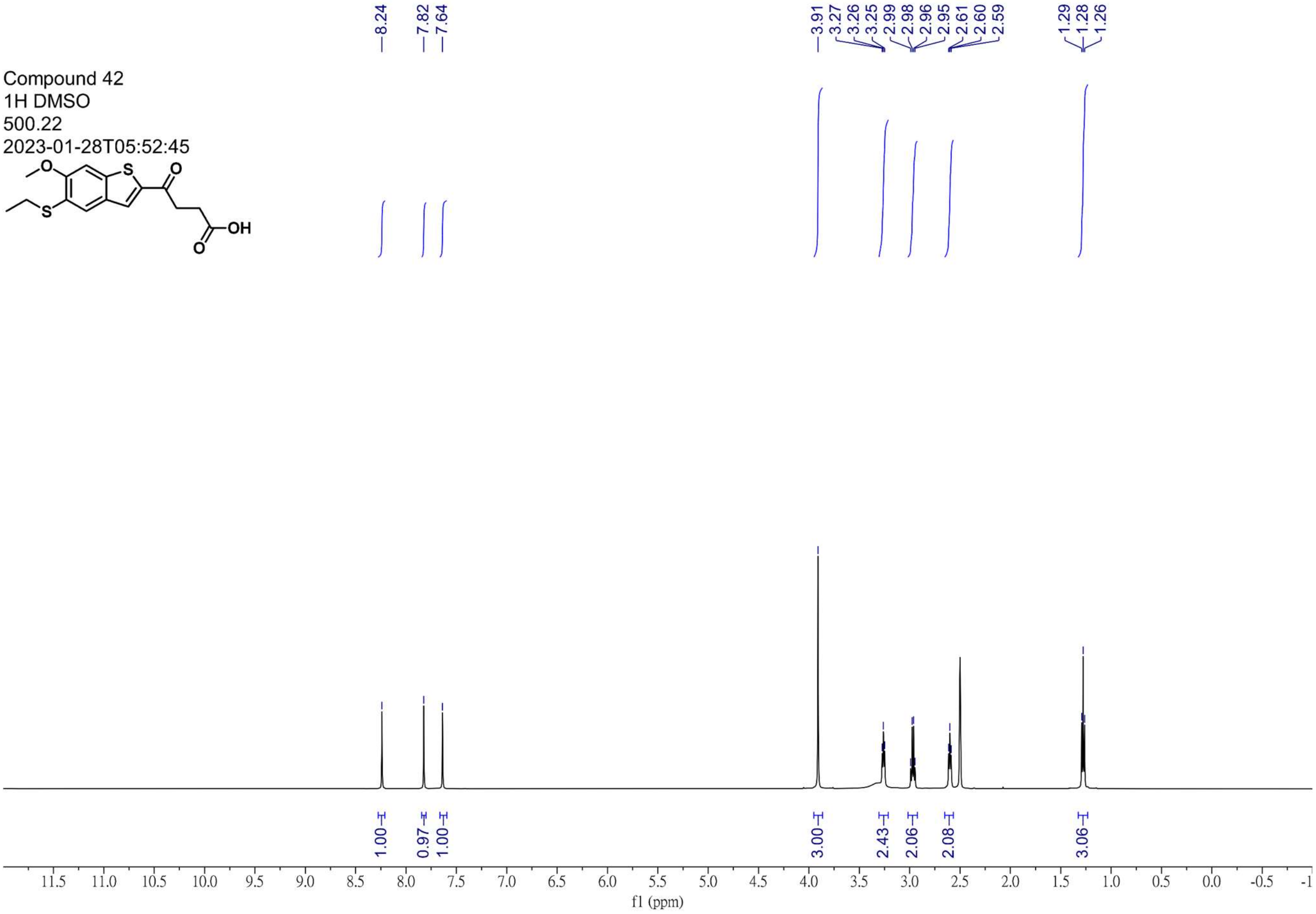
Compound 42
1H DMSO
500.22
2023-01-28T05:52:45
S
O
OH
8.24
7.82
7.64
3.91
3.27
3.26
3.25
2.99
2.98
2.96
2.95
2.61
2.60
2.59
1.29
1.28
1.26
1.00
0.97
1.00
3.00
2.43
2.06
2.08
3.06
f1 (ppm)

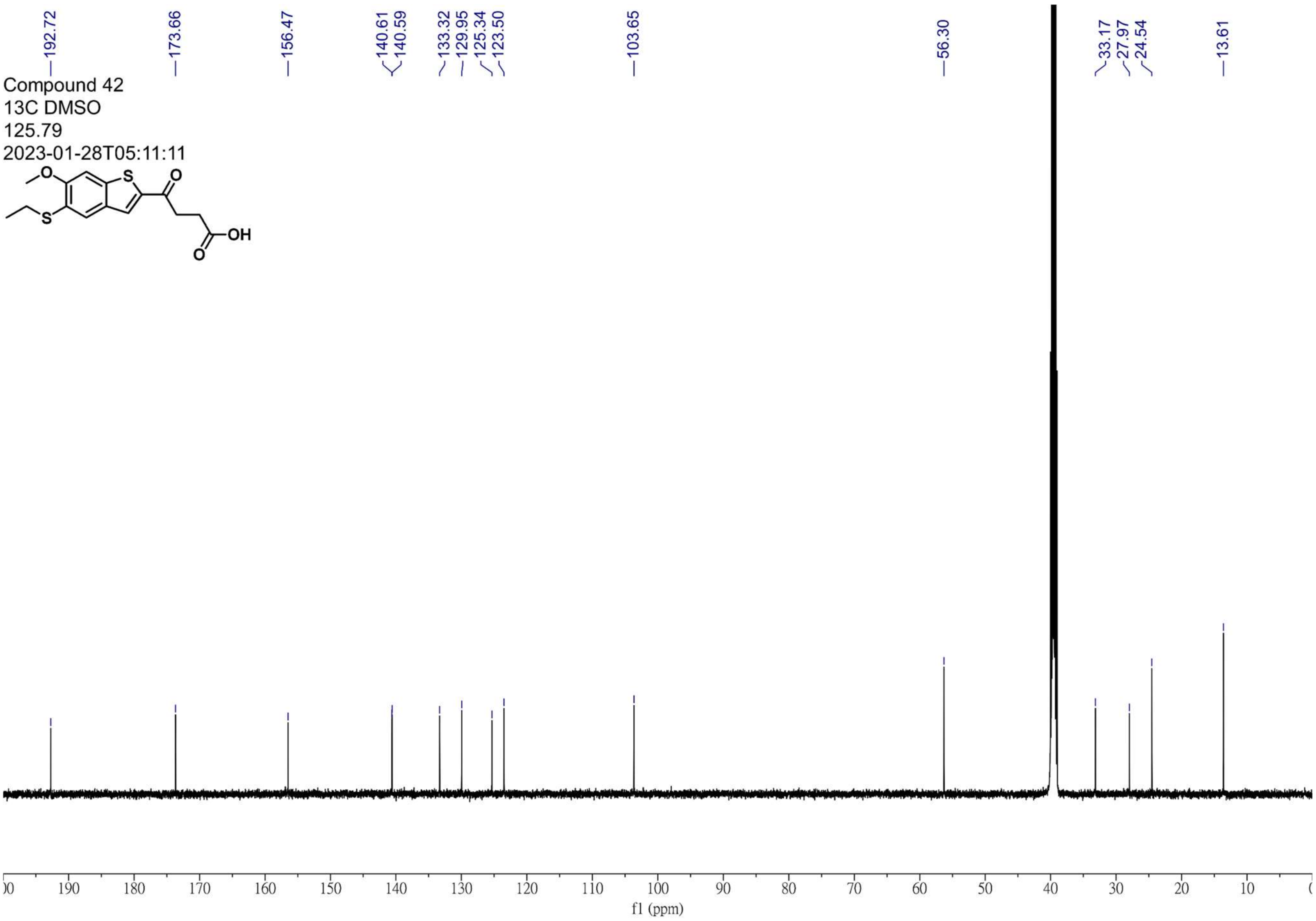
Compound 42
13C DMSO
125.79
2023-01-28T05:11:11
S
O
OH
192.72
173.66
156.47
140.61
140.59
133.32
129.95
125.34
123.50
103.65
56.30
33.17
27.97
24.54
13.61
f1 (ppm)

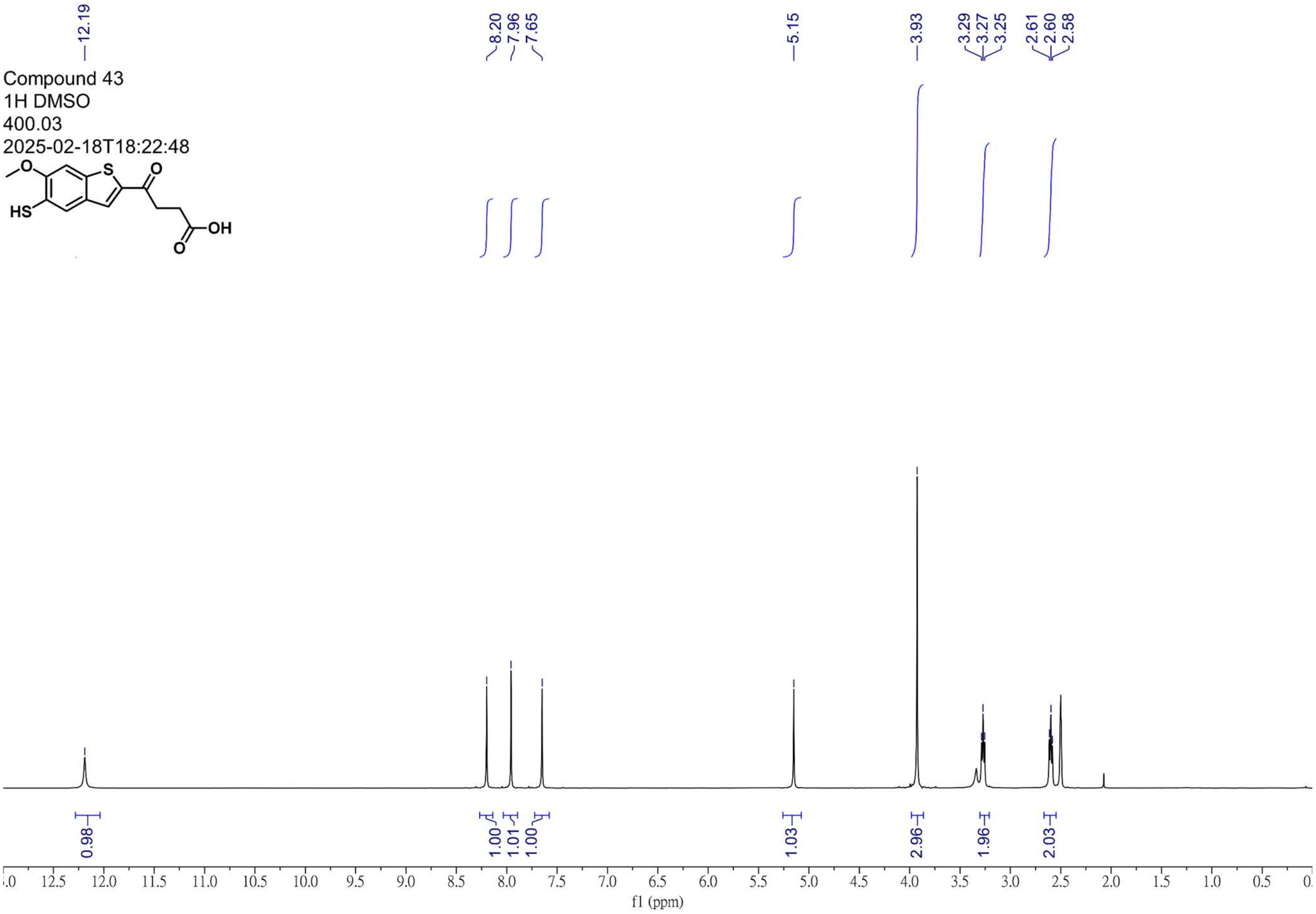
Compound 43
1H DMSO
400.03
2025-02-18T18:22:48
12.19
8.20
7.96
7.65
5.15
3.93
3.29
3.27
3.25
2.61
2.60
2.58
0.98
1.00
1.01
1.00
1.03
2.96
1.96
2.03
f1 (ppm)

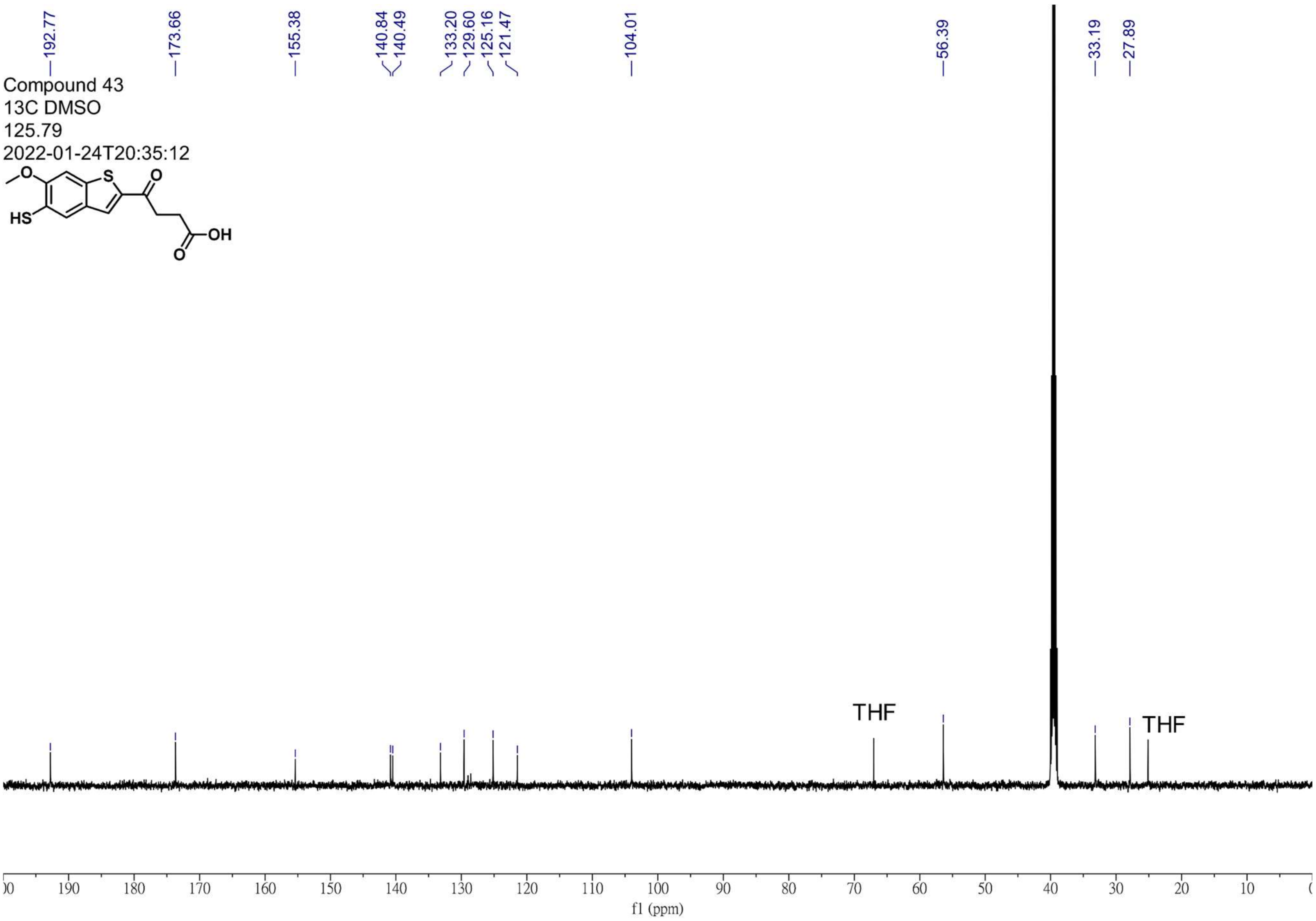
Compound 43
13C DMSO
125.79
2022-01-24T20:35:12
192.77
173.66
155.38
140.84
140.49
133.20
129.60
125.16
121.47
104.01
56.39
33.19
27.89
THF
THF
f1 (ppm)

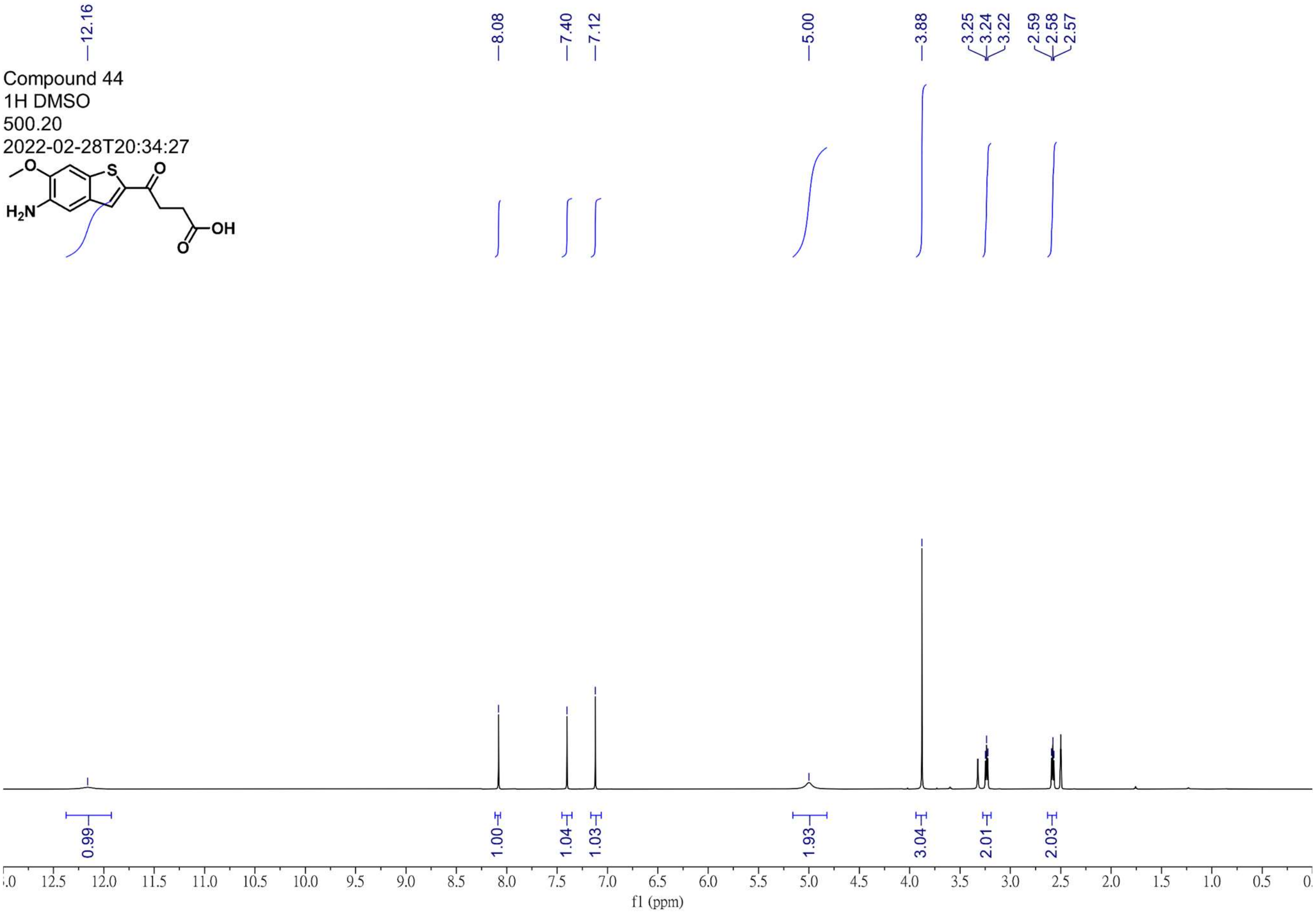
12.16
8.08
7.40
7.12
5.00
3.88
3.25
3.24
3.22
2.59
2.58
2.57
Compound 44
1H DMSO
500.20
2022-02-28T20:34:27
S
O
H2N
OH
0.99
1.00
1.04
1.03
1.93
3.04
2.01
2.03
f1 (ppm)

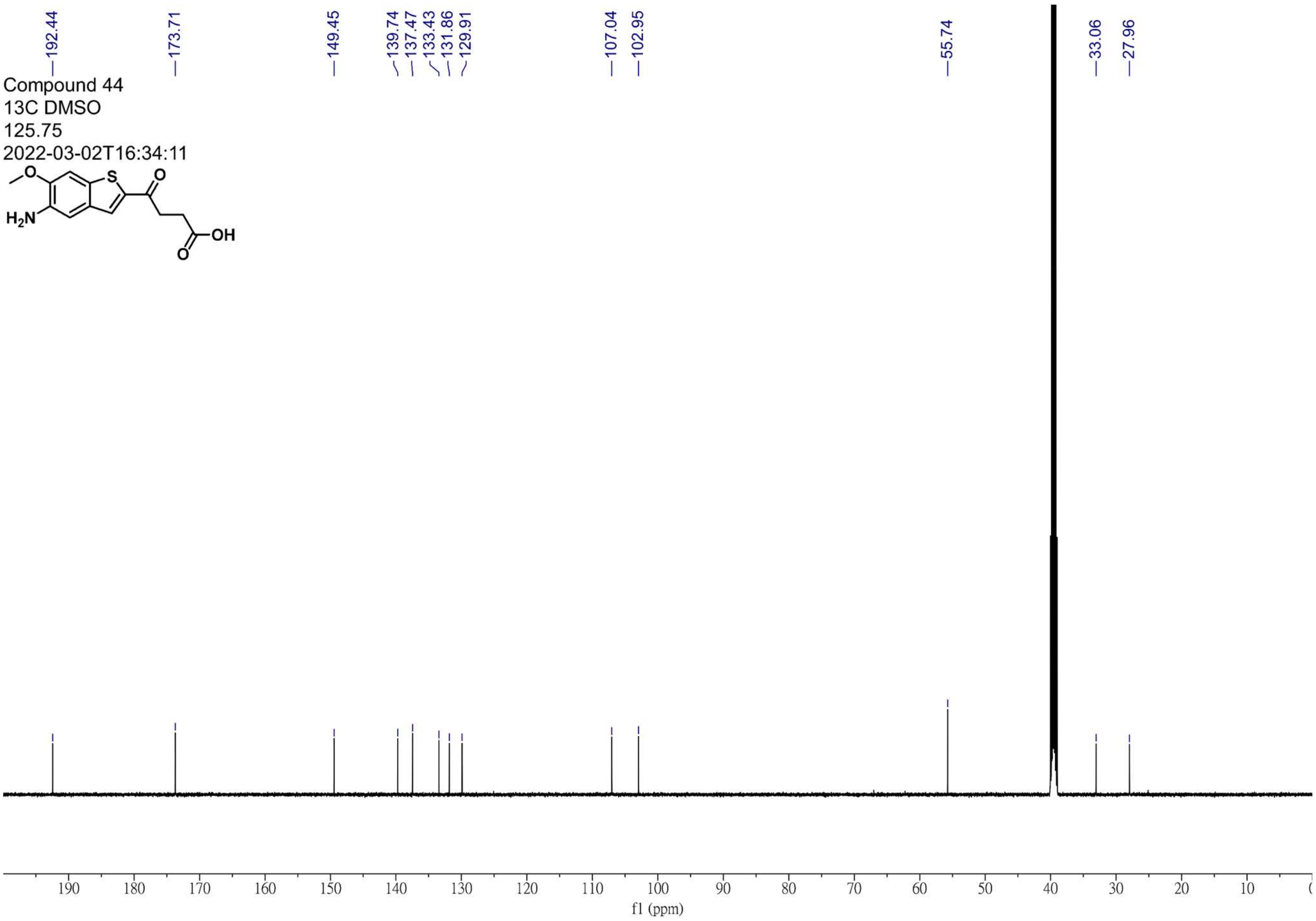
192.44
173.71
149.45
139.74
137.47
133.43
131.86
129.91
107.04
102.95
55.74
33.06
27.96
Compound 44
13C DMSO
125.75
2022-03-02T16:34:11
S
O
H2N
OH
f1 (ppm)

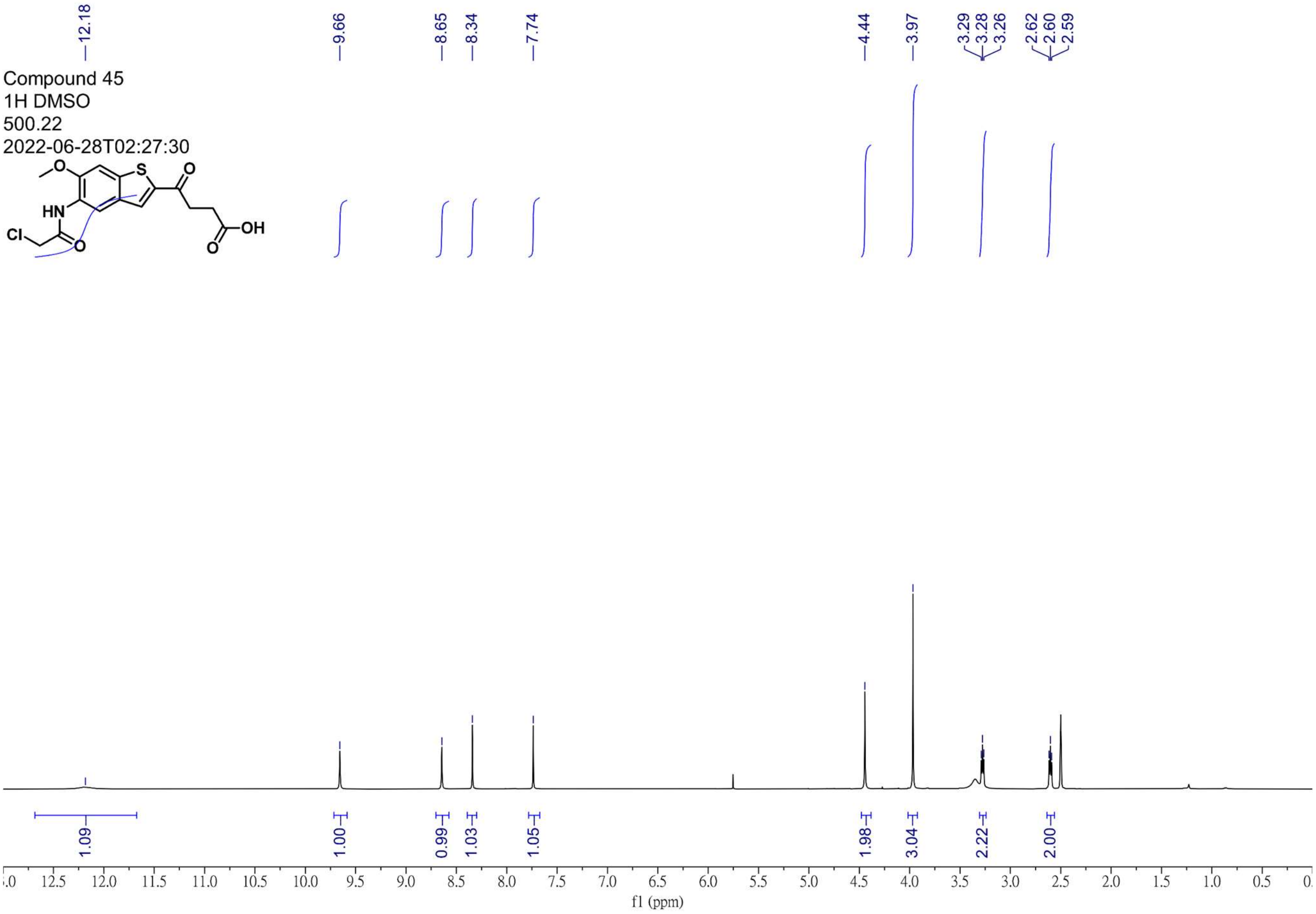

12.18
9.66
8.65
8.34
7.74
4.44
3.97
3.29
3.28
3.26
2.62
2.60
2.59
Compound 45
1H DMSO
500.22
2022-06-28T02:27:30
S
O
OH
HN
Cl
1.09
1.00
0.99
1.03
1.05
1.98
3.04
2.22
2.00
12.5 12.0 11.5 11.0 10.5 10.0 9.5 9.0 8.5 8.0 7.5 7.0 6.5 6.0 5.5 5.0 4.5 4.0 3.5 3.0 2.5 2.0 1.5 1.0 0.5
f1 (ppm)

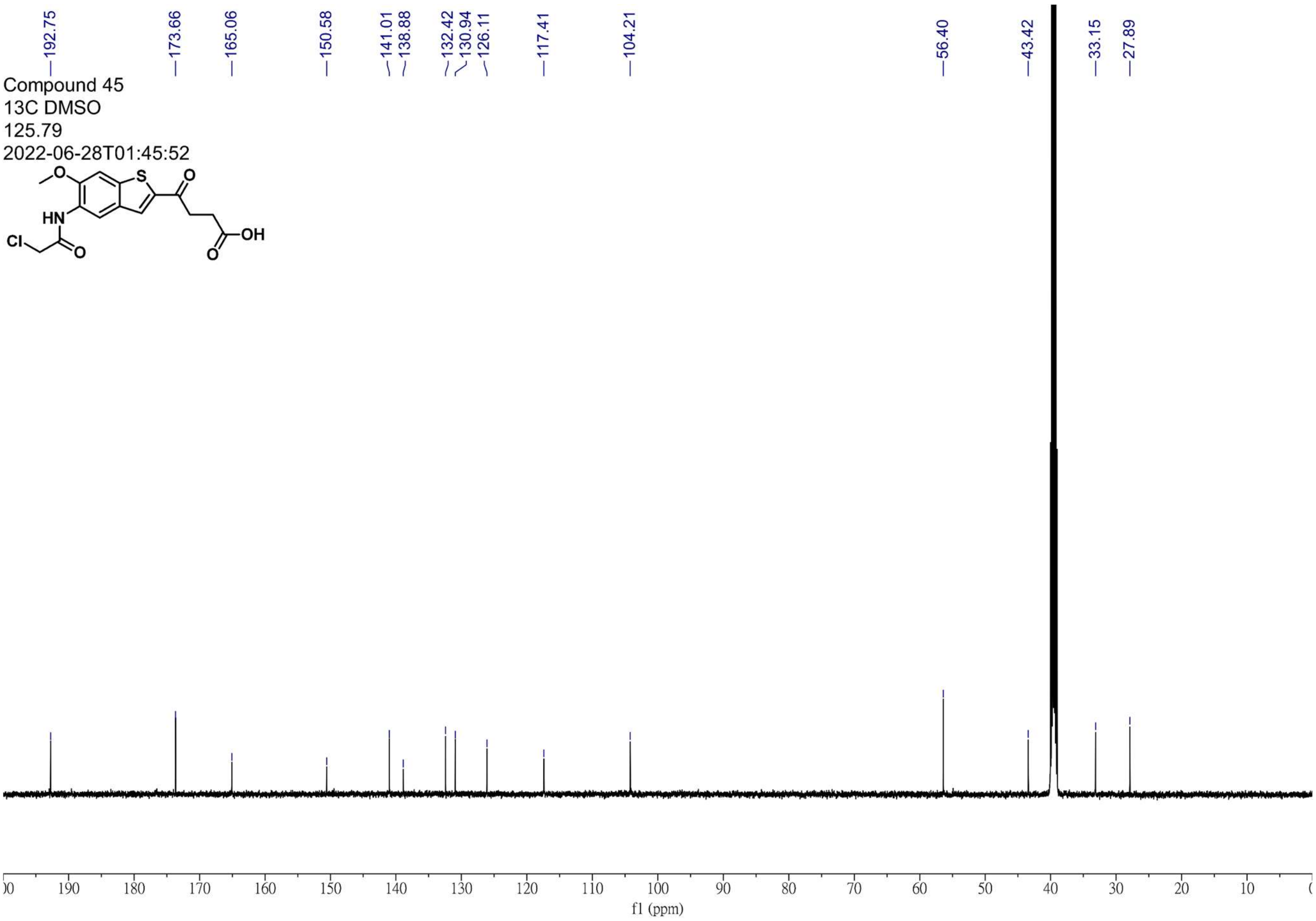

192.75
173.66
165.06
150.58
141.01
138.88
132.42
130.94
126.11
117.41
104.21
56.40
43.42
33.15
27.89
Compound 45
13C DMSO
125.79
2022-06-28T01:45:52
S
O
OH
HN
Cl
190 180 170 160 150 140 130 120 110 100 90 80 70 60 50 40 30 20 10
f1 (ppm)

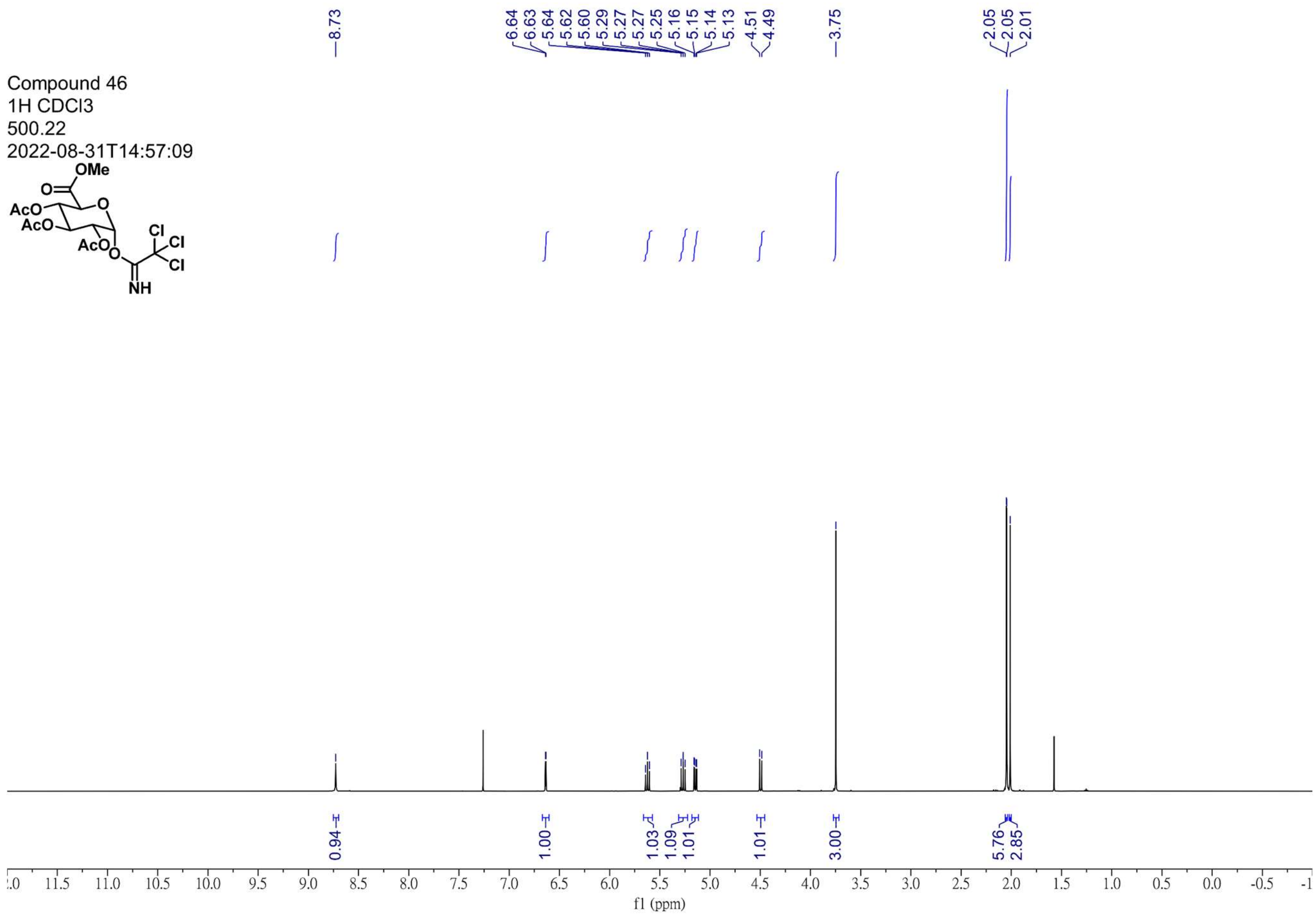
Compound 46
1H CDCl3
500.22
2022-08-31T14:57:09
OMe
O
AcO
AcO
AcO
O
Cl
Cl
Cl
NH
8.73
6.64
6.63
5.64
5.62
5.60
5.29
5.27
5.27
5.25
5.16
5.15
5.14
5.13
4.51
4.49
3.75
2.05
2.05
2.01
0.94
1.00
1.03
1.09
1.01
1.01
3.00
5.76
2.85
f1 (ppm)

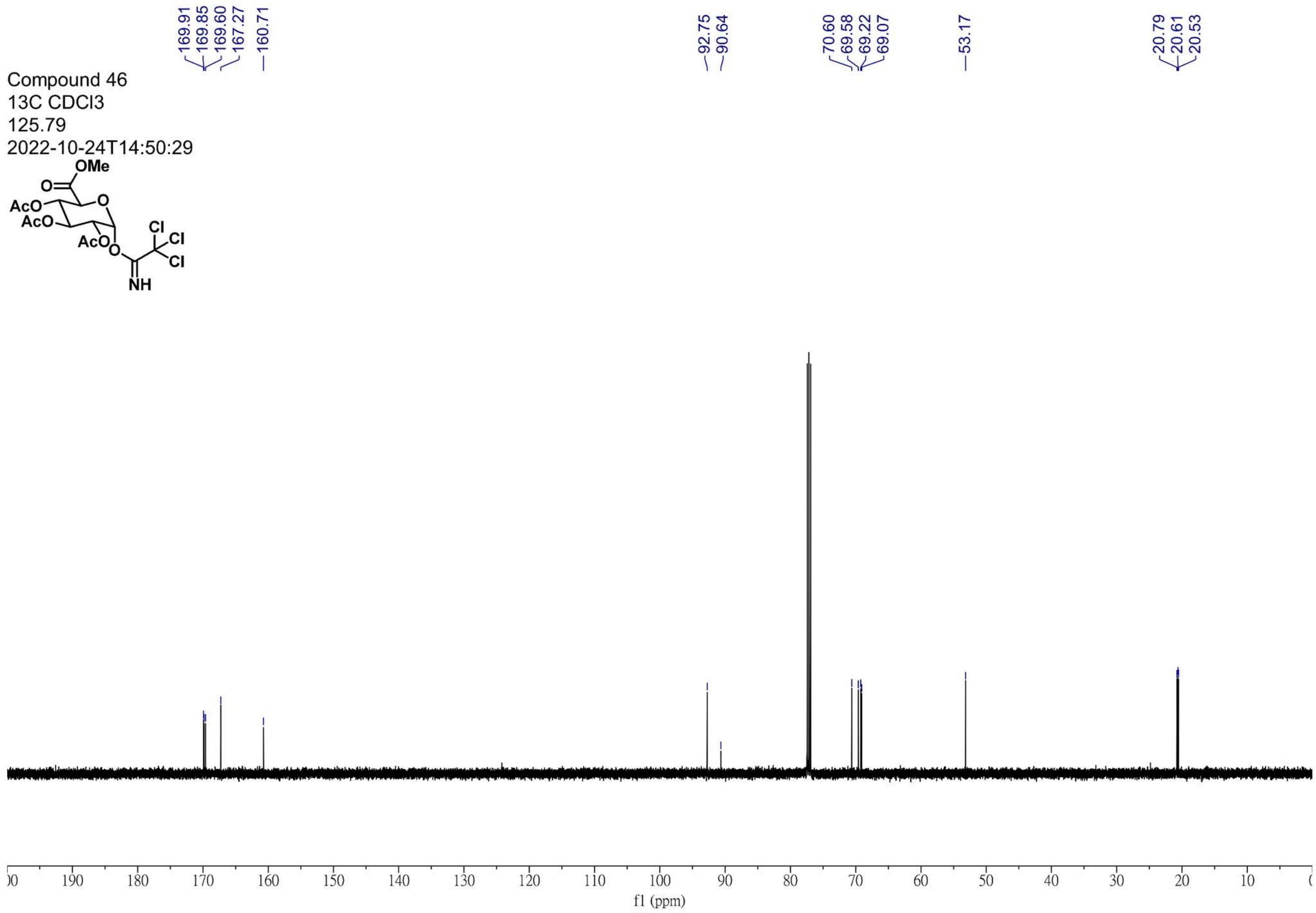
Compound 46
13C CDCl3
125.79
2022-10-24T14:50:29
OMe
O
AcO
AcO
AcO
O
Cl
Cl
Cl
NH
169.91
169.85
169.60
167.27
160.71
92.75
90.64
70.60
69.58
69.22
69.07
53.17
20.79
20.61
20.53
f1 (ppm)

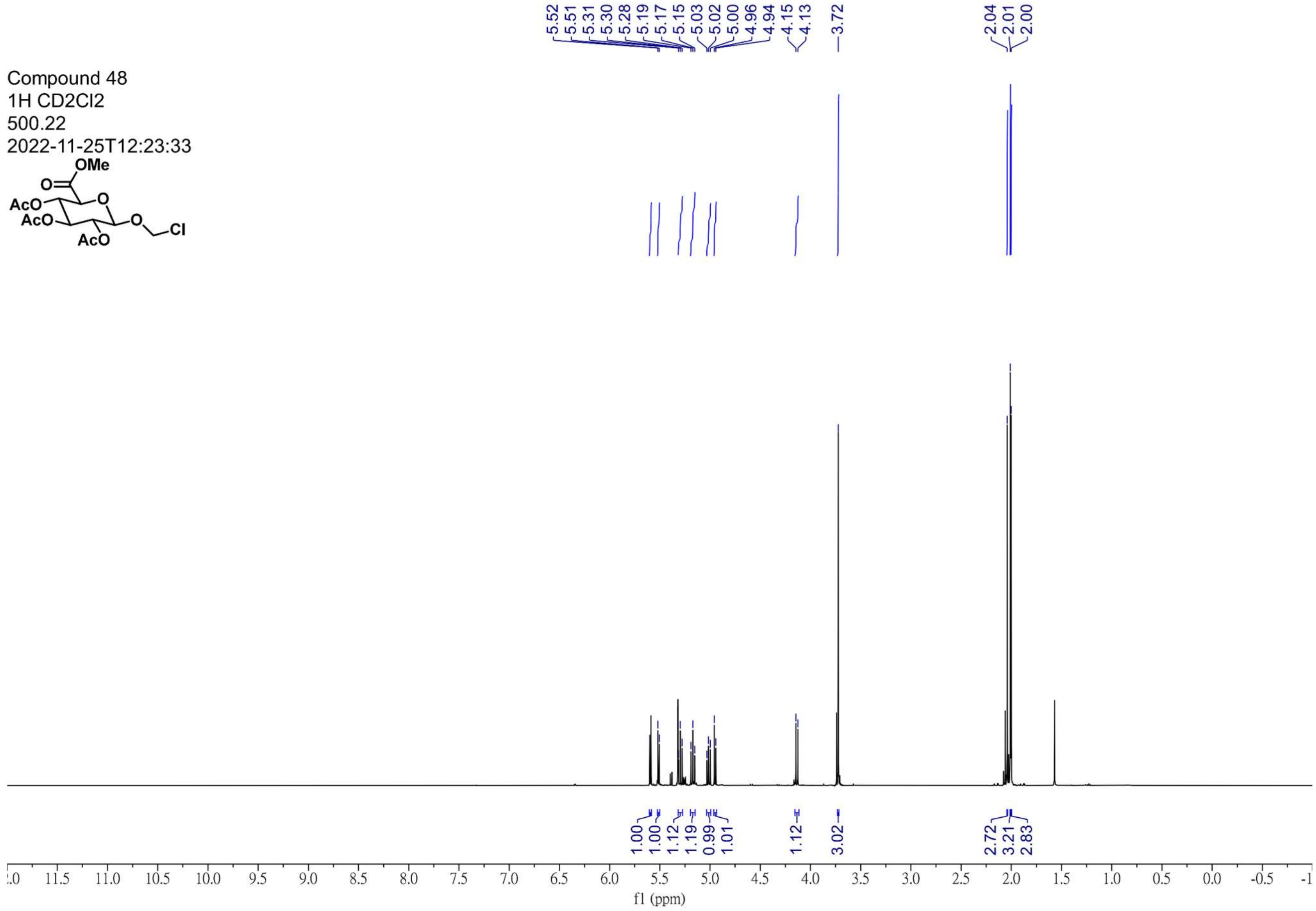

5.52 5.51 5.31 5.30 5.28 5.19 5.17 5.15 5.03 5.02 5.00 4.96 4.94 4.15 4.13 3.72
2.04 2.01 2.00
Compound 48
1H CD2Cl2
500.22
2022-11-25T12:23:33
OMe
O
AcO
AcO
O
O
Cl
AcO
1.00 1.00 1.12 1.19 0.99 1.01
1.12
3.02
2.72 3.21 2.83
11.5 11.0 10.5 10.0 9.5 9.0 8.5 8.0 7.5 7.0 6.5 6.0 5.5 5.0 4.5 4.0 3.5 3.0 2.5 2.0 1.5 1.0 0.5 0.0 -0.5
f1 (ppm)

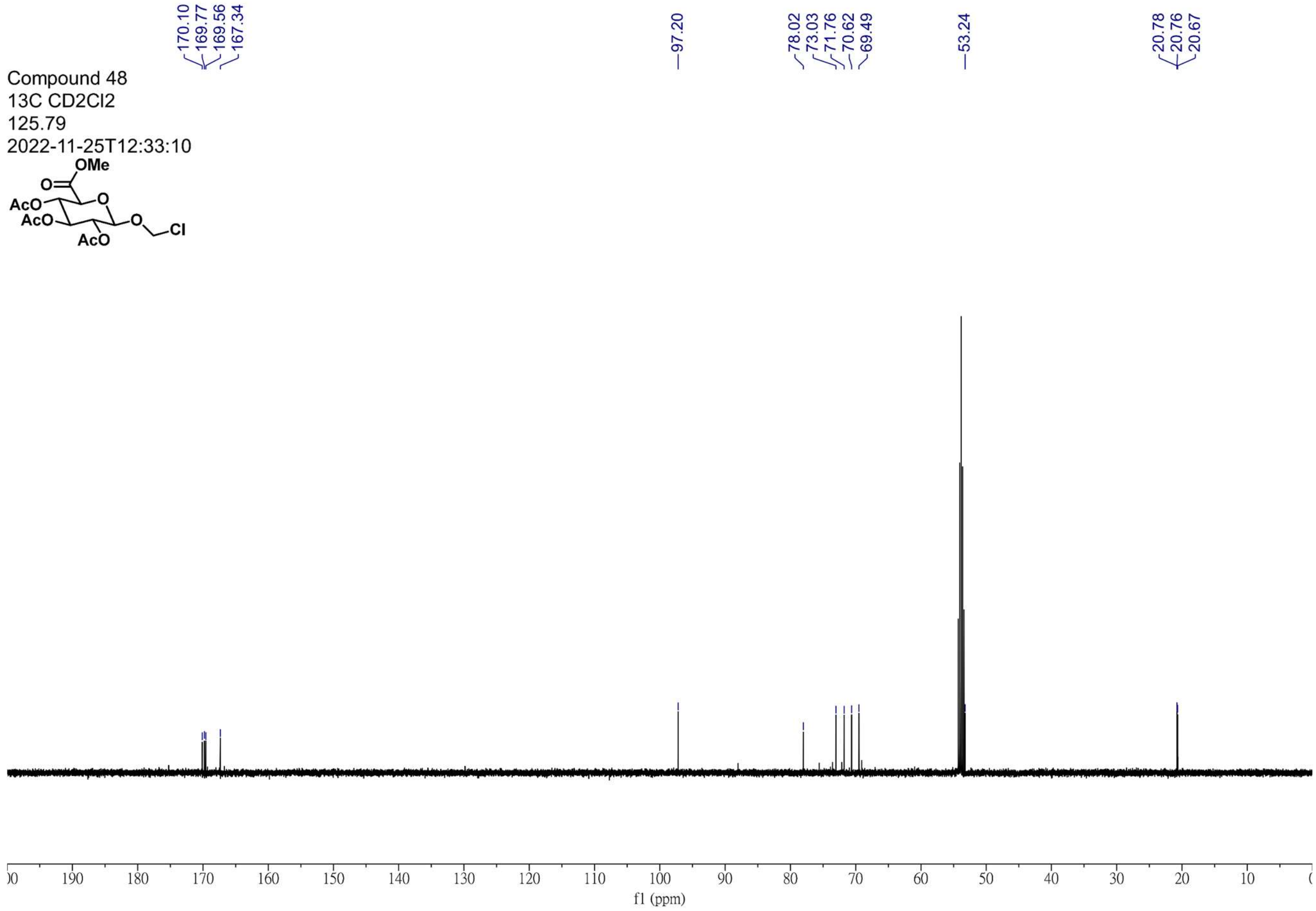

170.10 169.77 169.56 167.34
97.20
78.02 73.03 71.76 70.62 69.49
53.24
20.78 20.76 20.67
Compound 48
13C CD2Cl2
125.79
2022-11-25T12:33:10
OMe
O
AcO
AcO
O
O
Cl
AcO
190 180 170 160 150 140 130 120 110 100 90 80 70 60 50 40 30 20 10
f1 (ppm)

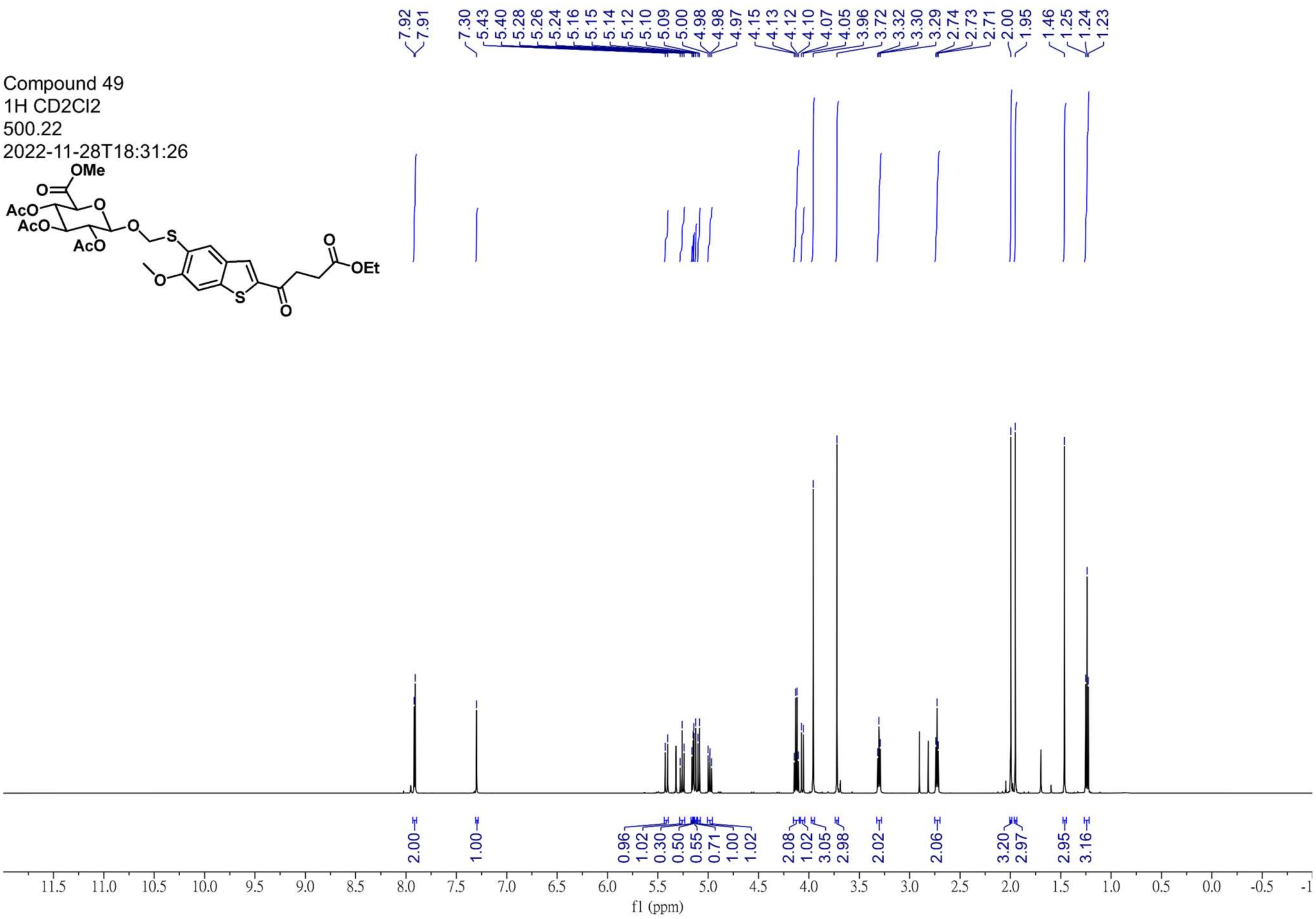
Compound 49
1H CD2Cl2
500.22
2022-11-28T18:31:26
OMe
AcO
AcO
AcO
O
S
OEt
7.92 7.91 7.30 5.43 5.40 5.28 5.26 5.24 5.16 5.15 5.14 5.12 5.10 5.09 5.00 4.98 4.98 4.97 4.15 4.13 4.12 4.10 4.07 4.05 3.96 3.72 3.32 3.30 3.29 2.74 2.73 2.71 2.00 1.95 1.46 1.25 1.24 1.23
2.00 1.00 0.96 1.02 0.30 0.50 0.55 0.71 1.00 1.02 2.08 1.02 3.05 2.98 2.02 2.06 3.20 2.97 2.95 3.16
f1 (ppm)

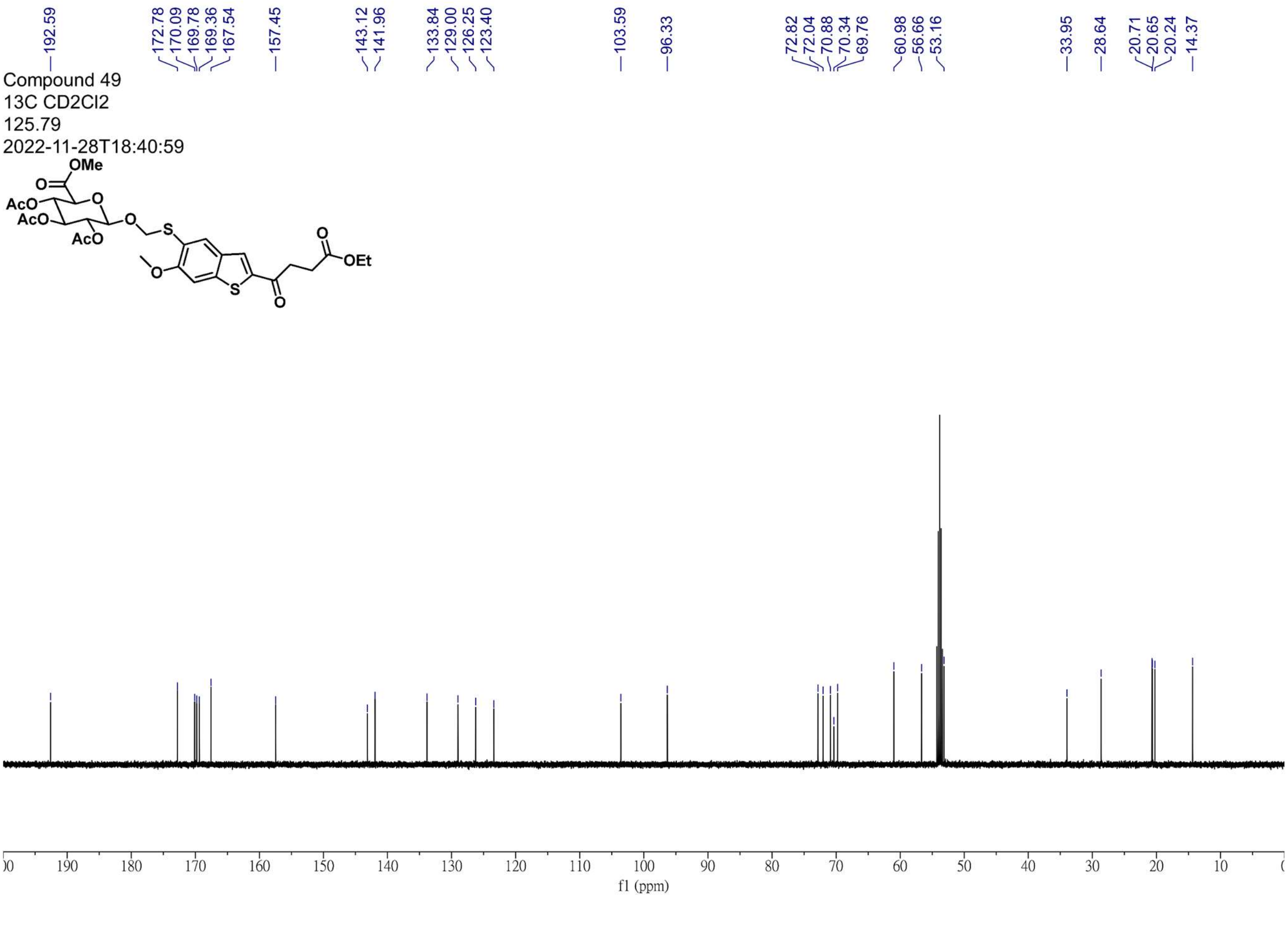
Compound 49
13C CD2Cl2
125.79
2022-11-28T18:40:59
OMe
AcO
AcO
AcO
O
S
OEt
192.59 172.78 170.09 169.78 169.36 167.54 157.45 143.12 141.96 133.84 129.00 126.25 123.40 103.59 96.33 72.82 72.04 70.88 70.34 69.76 60.98 56.66 53.16 33.95 28.64 20.71 20.65 20.24 14.37
f1 (ppm)

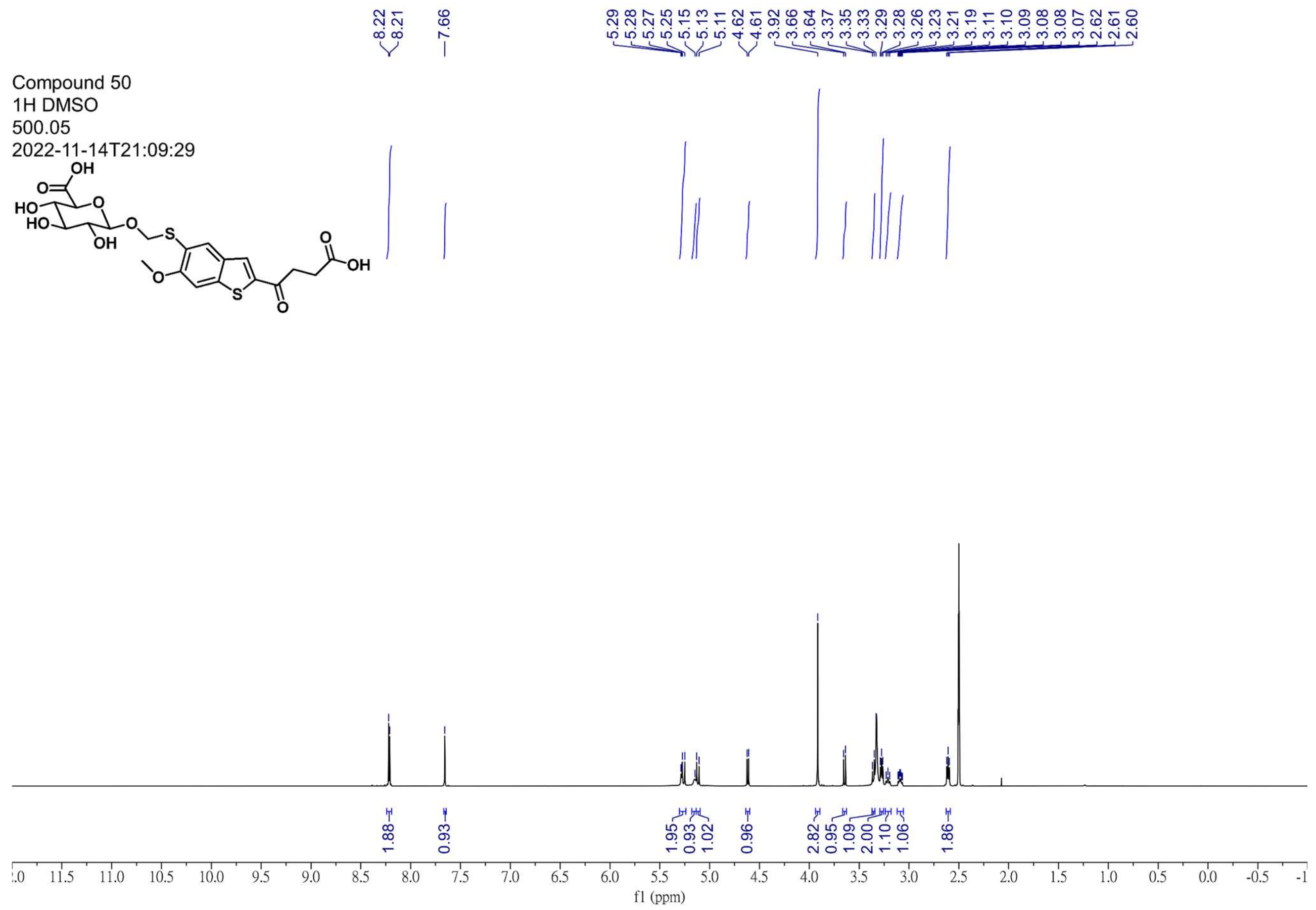
Compound 50
1H DMSO
500.05
2022-11-14T21:09:29
8.22
8.21
7.66
5.29
5.28
5.27
5.25
5.15
5.13
5.11
4.62
4.61
3.92
3.66
3.64
3.37
3.35
3.33
3.29
3.28
3.26
3.23
3.21
3.19
3.11
3.10
3.09
3.08
3.07
2.62
2.61
2.60
1.88
0.93
1.95
0.93
1.02
0.96
2.82
0.95
1.09
2.00
1.10
1.06
1.86
f1 (ppm)

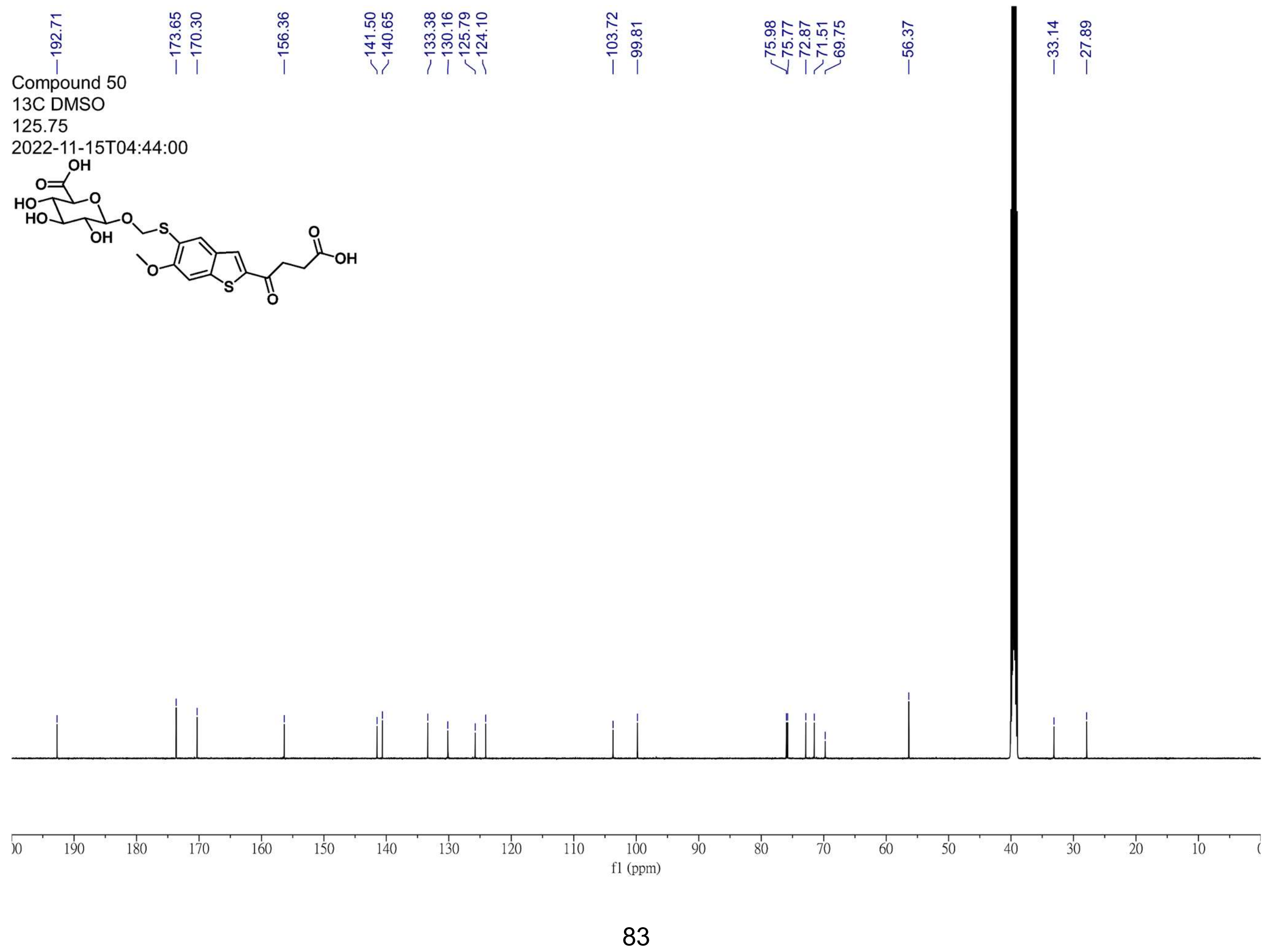
Compound 50
13C DMSO
125.75
2022-11-15T04:44:00
192.71
173.65
170.30
156.36
141.50
140.65
133.38
130.16
125.79
124.10
103.72
99.81
75.98
75.77
72.87
71.51
69.75
56.37
33.14
27.89
f1 (ppm)

HRMS Spectra

Spectrum Plot Report

Name	2F-4MeO-5Br	Rack Pos.		Instrument	Instrument 1	Operator	Nai-Shu Hsu
Inj. Vol. (ul)	2	Plate Pos.		IRM Status	Success		
Data File	2F-4MeO-5Br0489.d	Method (Acq)	Walkup Positive SM.m	Comment	MBAG/461-RG97836-Task1	Acq. Time (Local)	1/4/2022 6:21:43 PM (UTC+01:00)

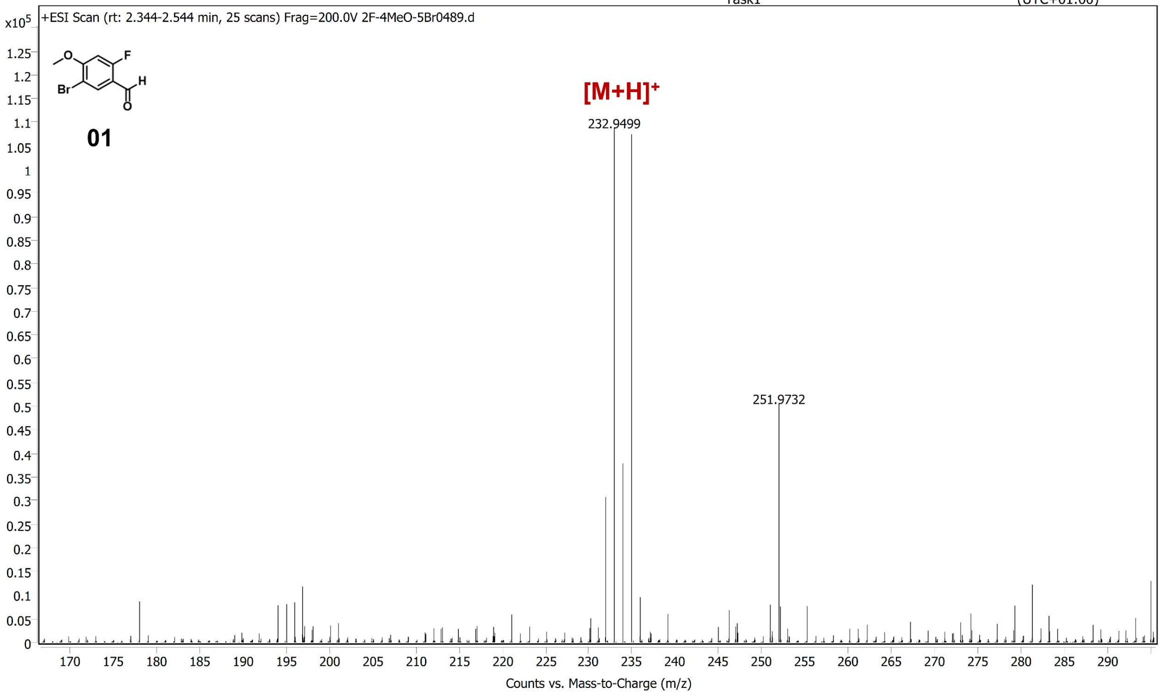

Page 1 of 1 Generated at 7:53 PM on 4/6/2022

Spectrum Plot Report

Name	thiophene ethyl ester	Rack Pos.		Instrument	Instrument 1	Operator	Nai-Shu Hsu
Inj. Vol. (ul)	2	Plate Pos.		IRM Status	Success		
Data File	thiophene ethyl ester0491.d	Method (Acq)	Walkup Positive SM.m	Comment	MBAG/461-RG97836-Task1	Acq. Time (Local)	1/6/2022 8:45:04 PM (UTC+01:00)

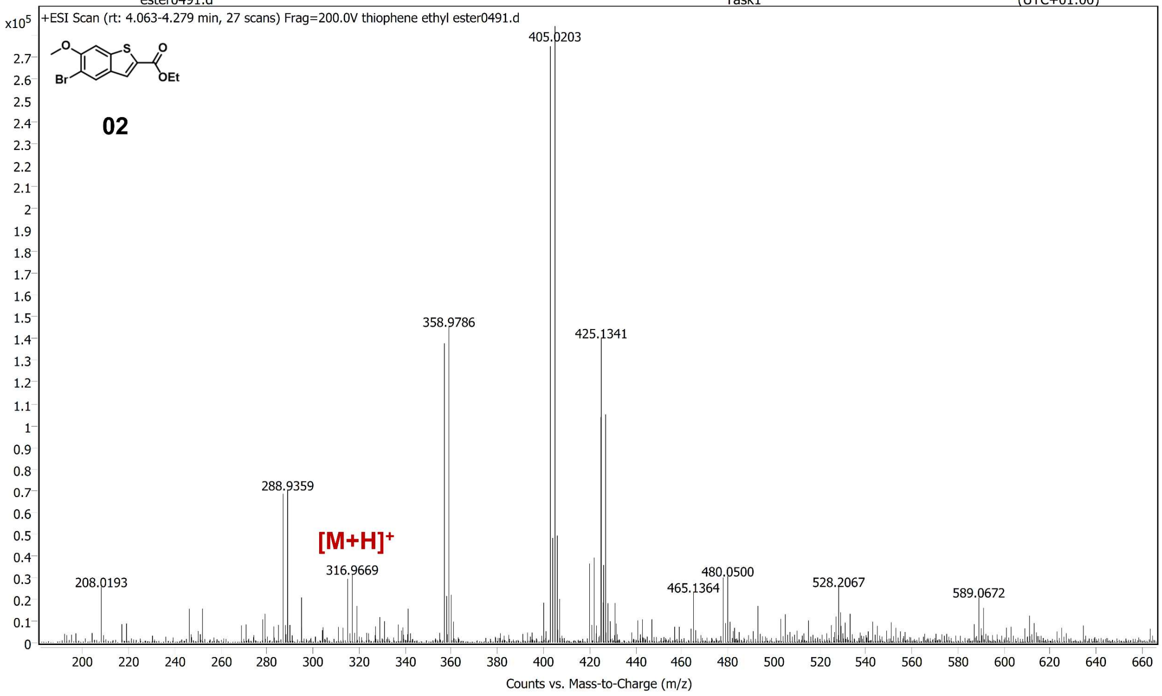

Page 1 of 1 Generated at 8:20 PM on 4/6/2022

Spectrum Plot Report

Agilent | Trusted Answers

Name	5BrThiopheneAcid	Rack Pos.		Instrument	Instrument 1	Operator	Nai-Shu Hsu
Inj. Vol. (ul)	2	Plate Pos.		IRM Status	Success		
Data File	5BrThiopheneAcid0495 .d	Method (Acq)	Walkup Negative SM.m	Comment	MBAG/461-RG97836-Task1	Acq. Time (Local)	1/8/2022 6:54:04 PM (UTC+01:00)

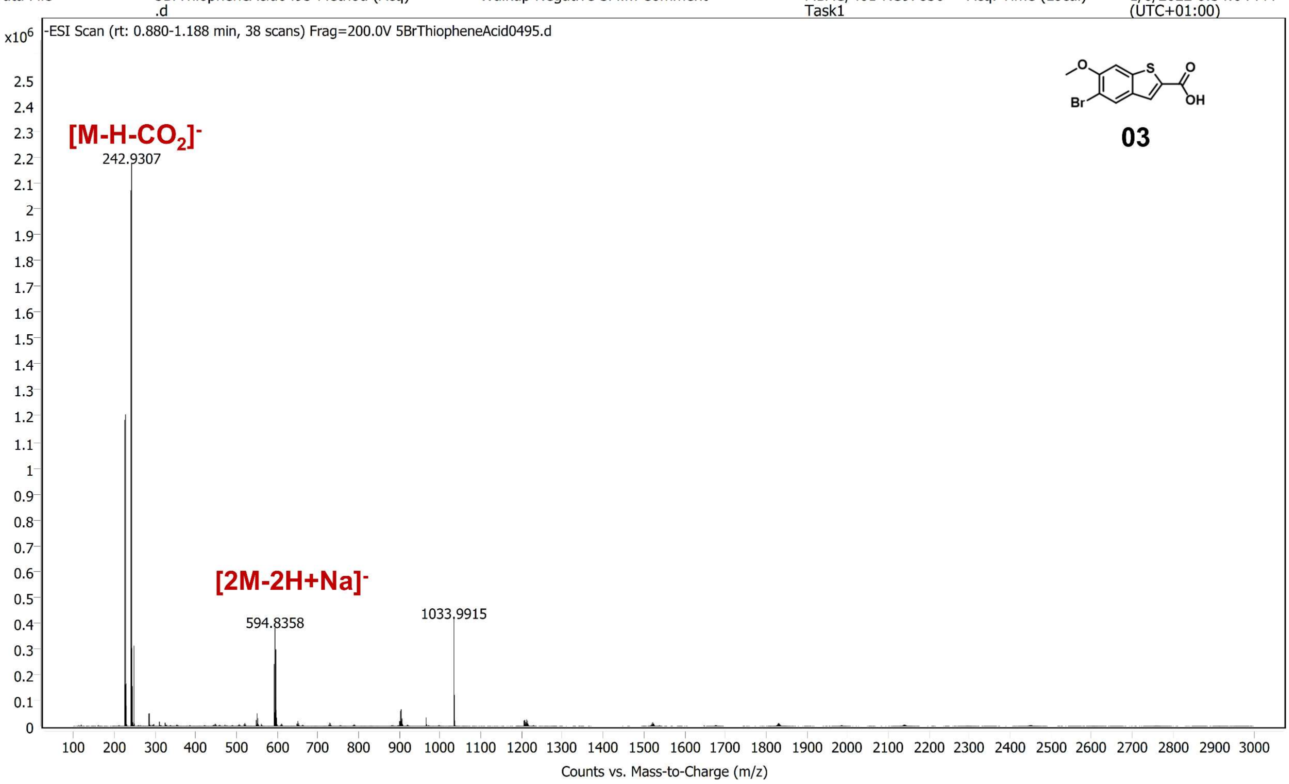

 Generated at 8:16 PM on 4/6/2022

Spectrum Plot Report

Agilent | Trusted Answers

Name	Zn coupling pure jan15	Rack Pos.		Instrument	Instrument 1	Operator	Nai-Shu Hsu
Inj. Vol. (ul)	2	Plate Pos.		IRM Status	Success		
Data File	Zn coupling pure jan150545.d	Method (Acq)	Walkup Positive SM.m	Comment	MBAG/461-RG97836-Task1	Acq. Time (Local)	1/15/2022 7:07:07 PM (UTC+00:00)

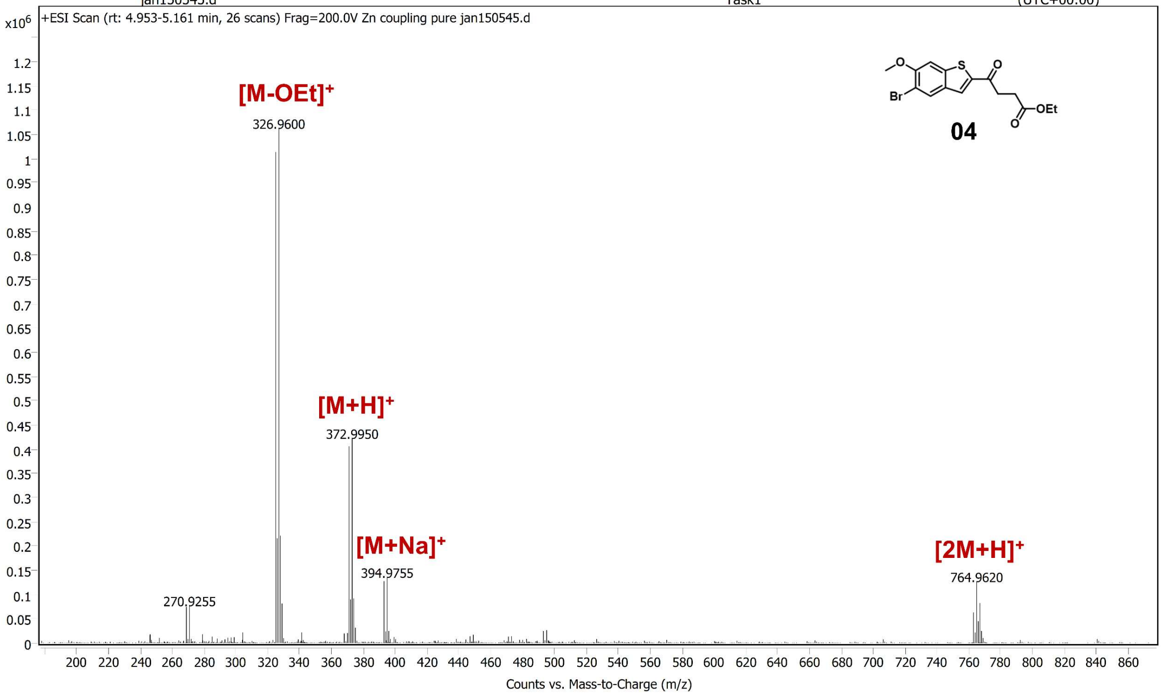

 Generated at 9:04 PM on 2/18/2022

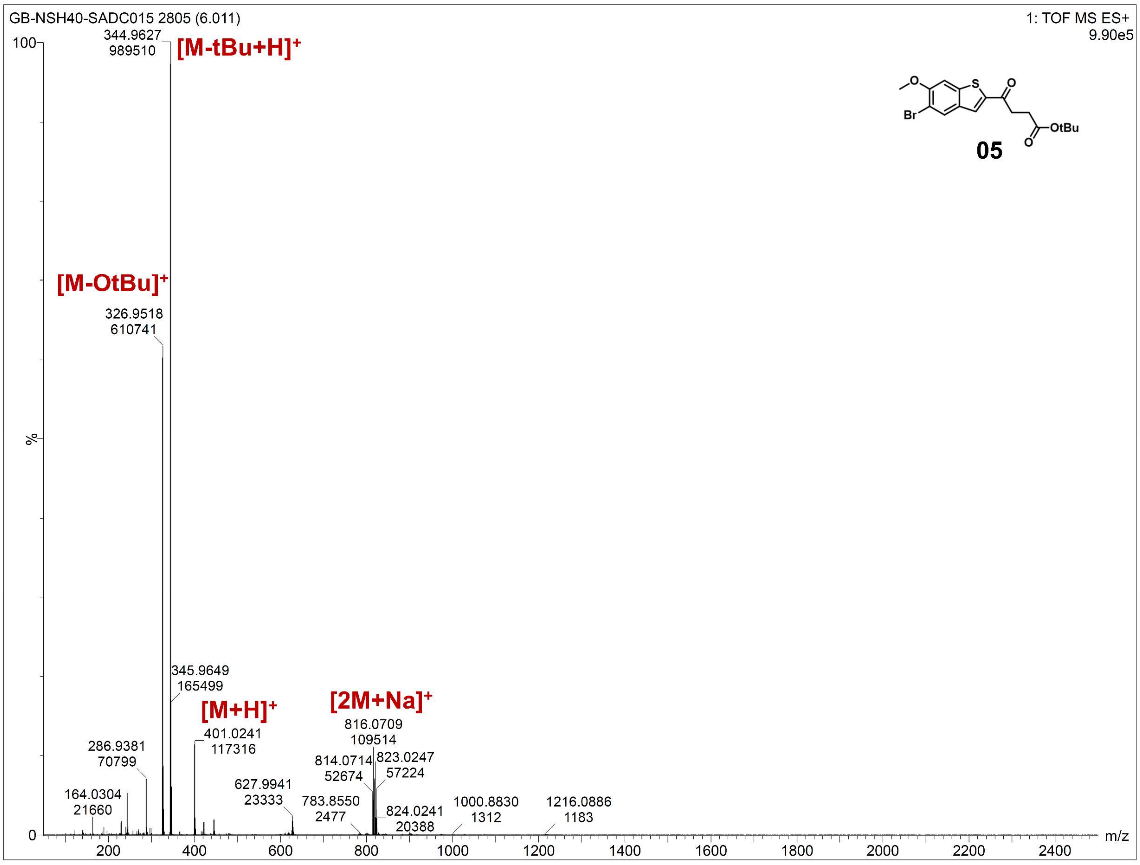
GB-NSH40-SADC015 2805 (6.011)
1: TOF MS ES+
9.90e5
344.9627
989510
[M-tBu+H]+
[M-OtBu]+
326.9518
610741
345.9649
165499
[M+H]+
401.0241
117316
[2M+Na]+
816.0709
109514
286.9381
70799
814.0714
52674
823.0247
57224
627.9941
23333
164.0304
21660
783.8550
2477
824.0241
20388
1000.8830
1312
1216.0886
1183
Br
S
OtBu
05
%
m/z

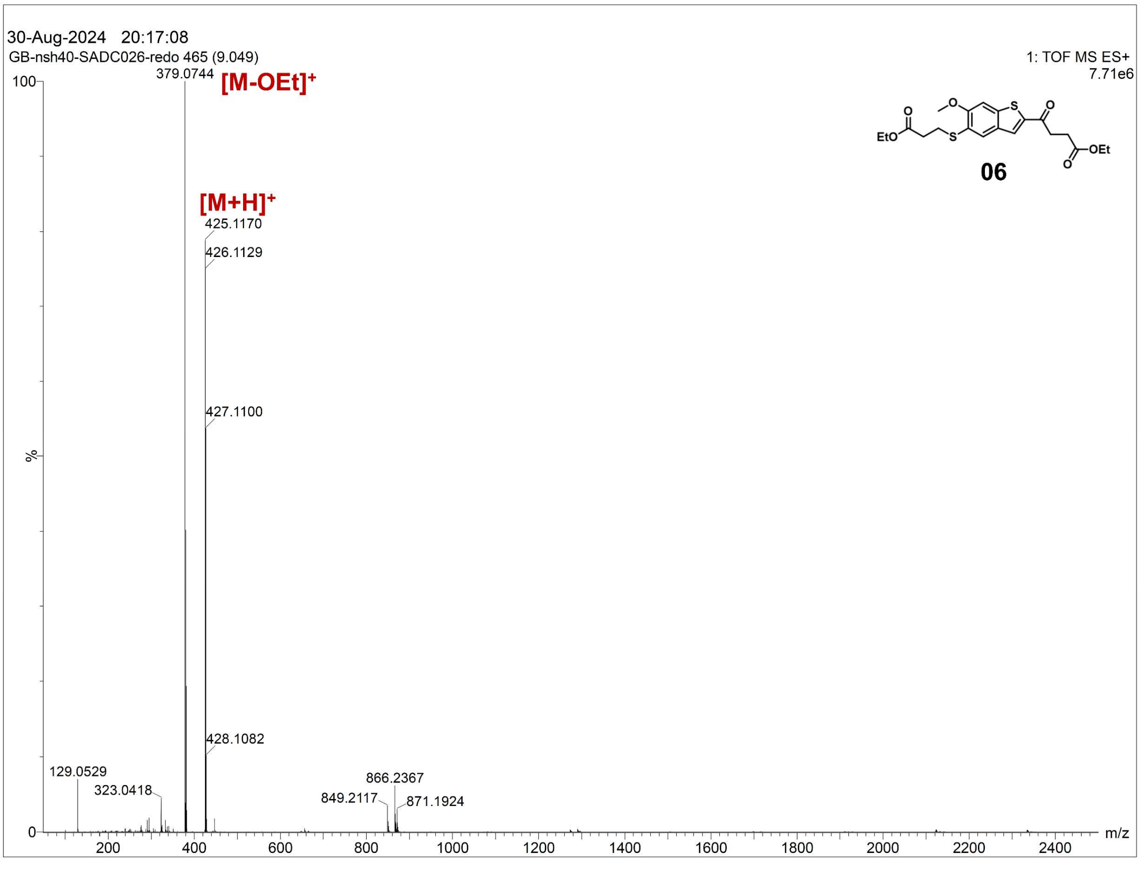
30-Aug-2024 20:17:08
GB-nsh40-SADC026-redo 465 (9.049)
1: TOF MS ES+
7.71e6
379.0744
[M-OEt]+
[M+H]+
425.1170
426.1129
427.1100
428.1082
129.0529
323.0418
866.2367
849.2117
871.1924
EtO
S
OEt
06
%
m/z

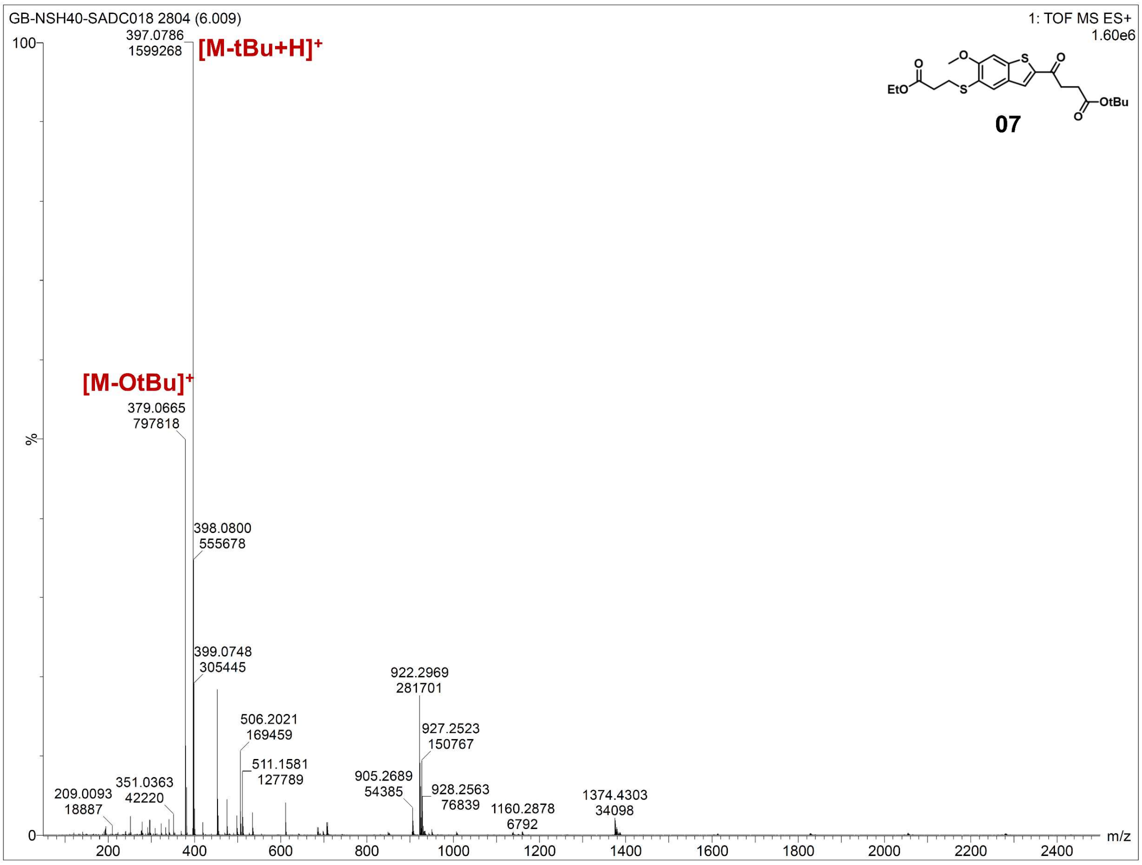

Compound Chromatogram(s)

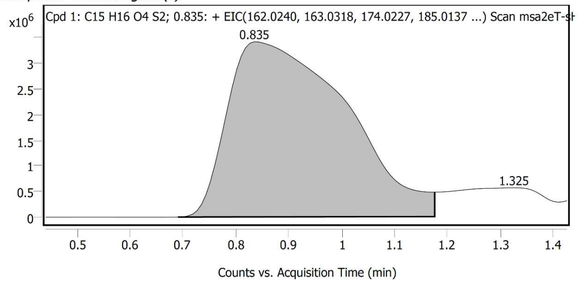

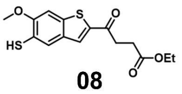

08

Compound Spectra (overlaid)

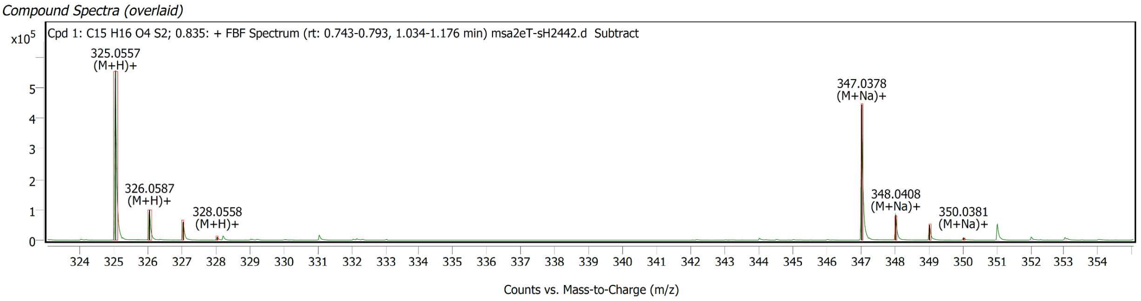

Spectrum Peaks

m/z	Z	Abund	Diff (ppm)	Height %	Height % (Calc)	Ion Species	Formula
325.0557	1	556711	-1.73	100.00	100.00	(M+H)+	C15H16O4S2
326.0587	1	97544	-1.83	17.52	18.15	(M+H)+	C15H16O4S2
327.0538	1	60452	-1.01	10.86	11.32	(M+H)+	C15H16O4S2
328.0558	1	9801	-1.83	1.76	1.79	(M+H)+	C15H16O4S2
347.0378	1	444840	-1.32	100.00	100.00	(M+Na)+	C15H16O4S2
348.0408	1	82673	-1.44	18.58	18.14	(M+Na)+	C15H16O4S2

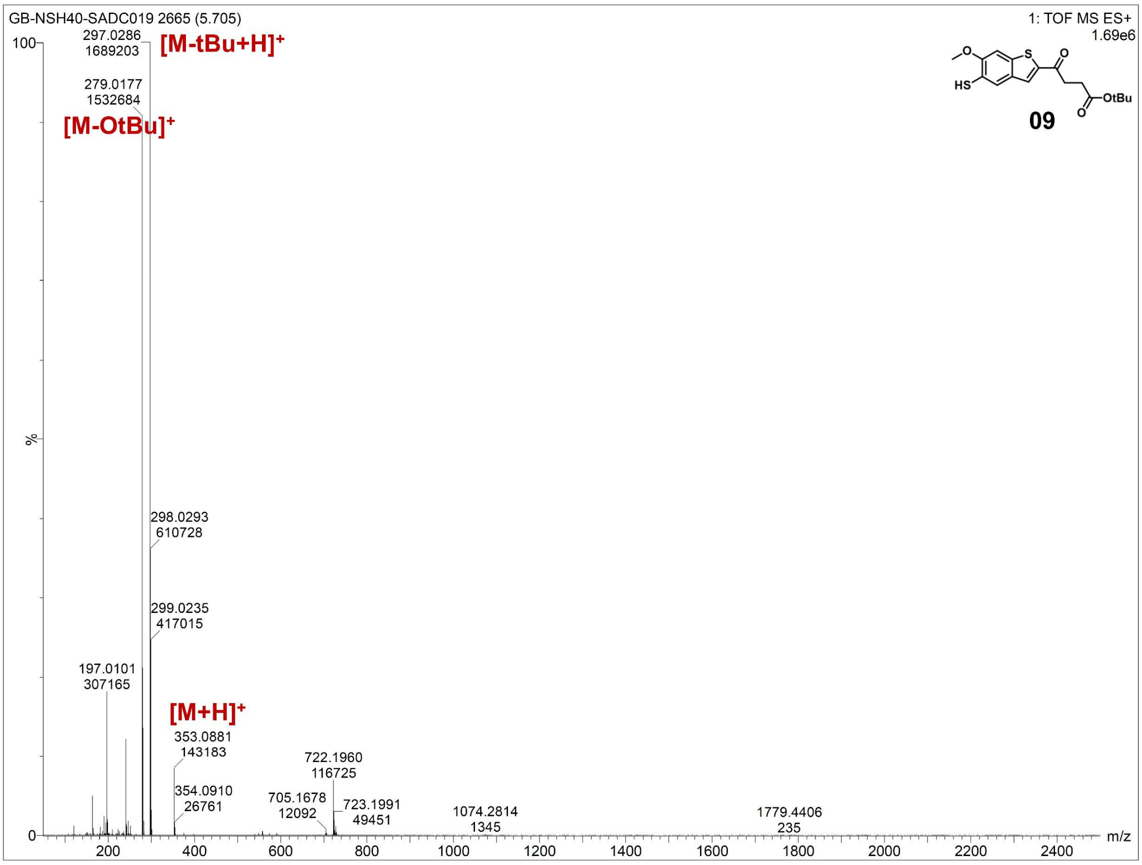
GB-NSH40-SADC019 2665 (5.705)
1: TOF MS ES+
1.69e6
297.0286
1689203
[M-tBu+H]+
279.0177
1532684
[M-OtBu]+
298.0293
610728
299.0235
417015
197.0101
307165
[M+H]+
353.0881
143183
722.1960
116725
354.0910
26761
705.1678
12092
723.1991
49451
1074.2814
1345
1779.4406
235
m/z
%
09
HS
S
O
OtBu

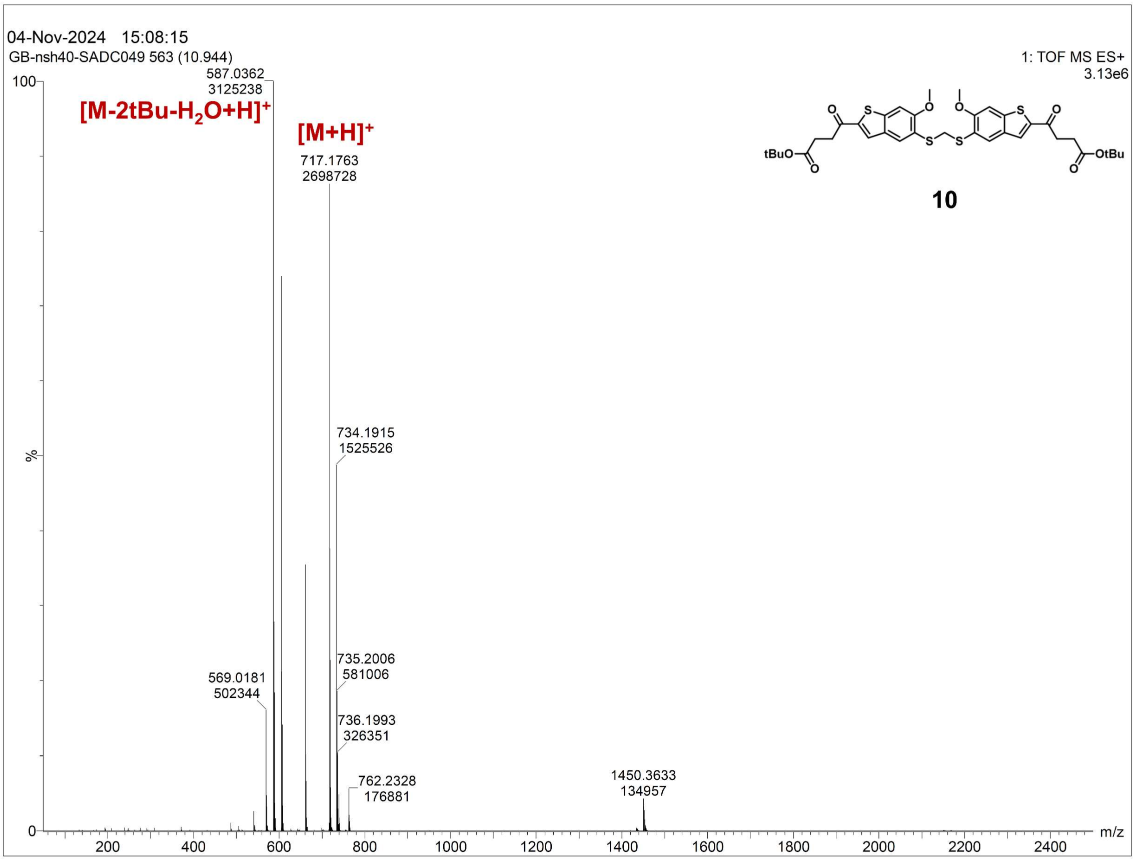
04-Nov-2024 15:08:15
GB-nsh40-SADC049 563 (10.944)
1: TOF MS ES+
3.13e6
587.0362
3125238
[M-2tBu-H2O+H]+
[M+H]+
717.1763
2698728
734.1915
1525526
735.2006
581006
569.0181
502344
736.1993
326351
762.2328
176881
1450.3633
134957
m/z
%
10
tBuO
OtBu
S
O

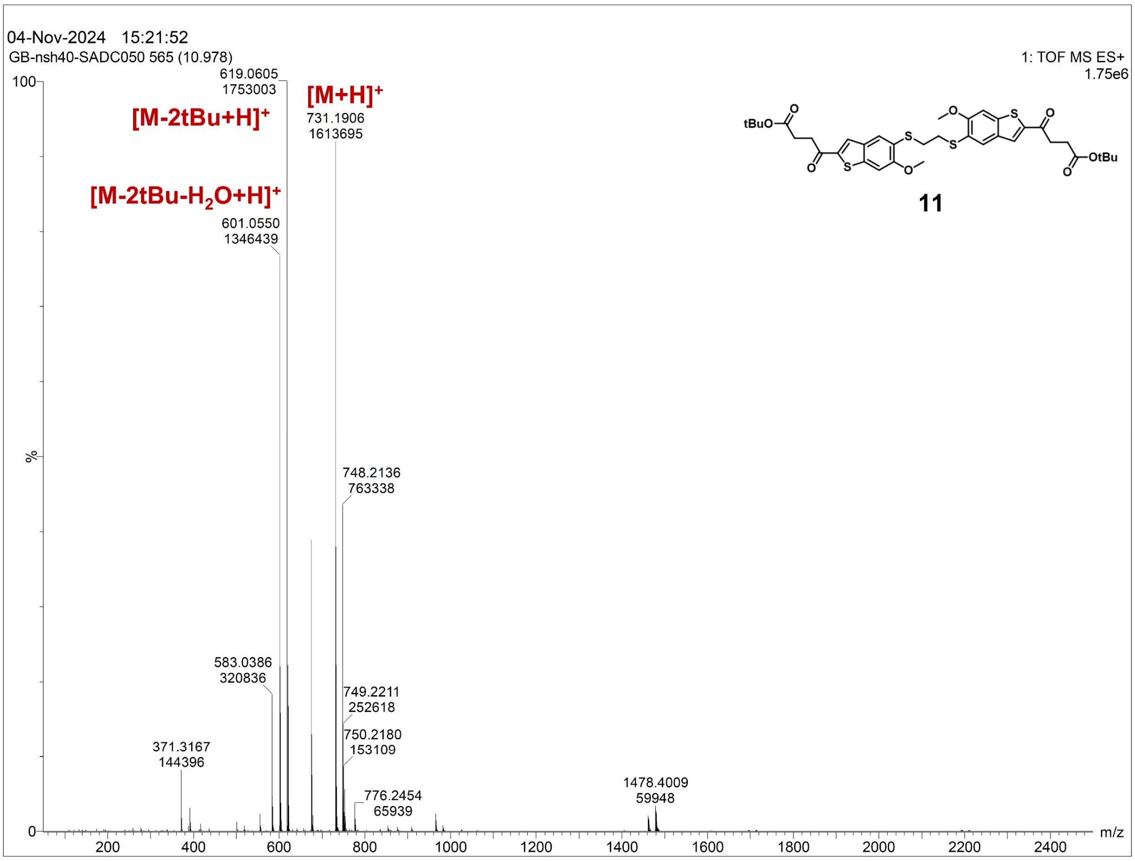
04-Nov-2024 15:21:52
GB-nsh40-SADC050 565 (10.978)
1: TOF MS ES+
1.75e6
[M-2tBu+H]+
619.0605
1753003
[M+H]+
731.1906
1613695
[M-2tBu-H2O+H]+
601.0550
1346439
748.2136
763338
583.0386
320836
749.2211
252618
750.2180
153109
371.3167
144396
776.2454
65939
1478.4009
59948
tBuO
OtBu
11
%
m/z

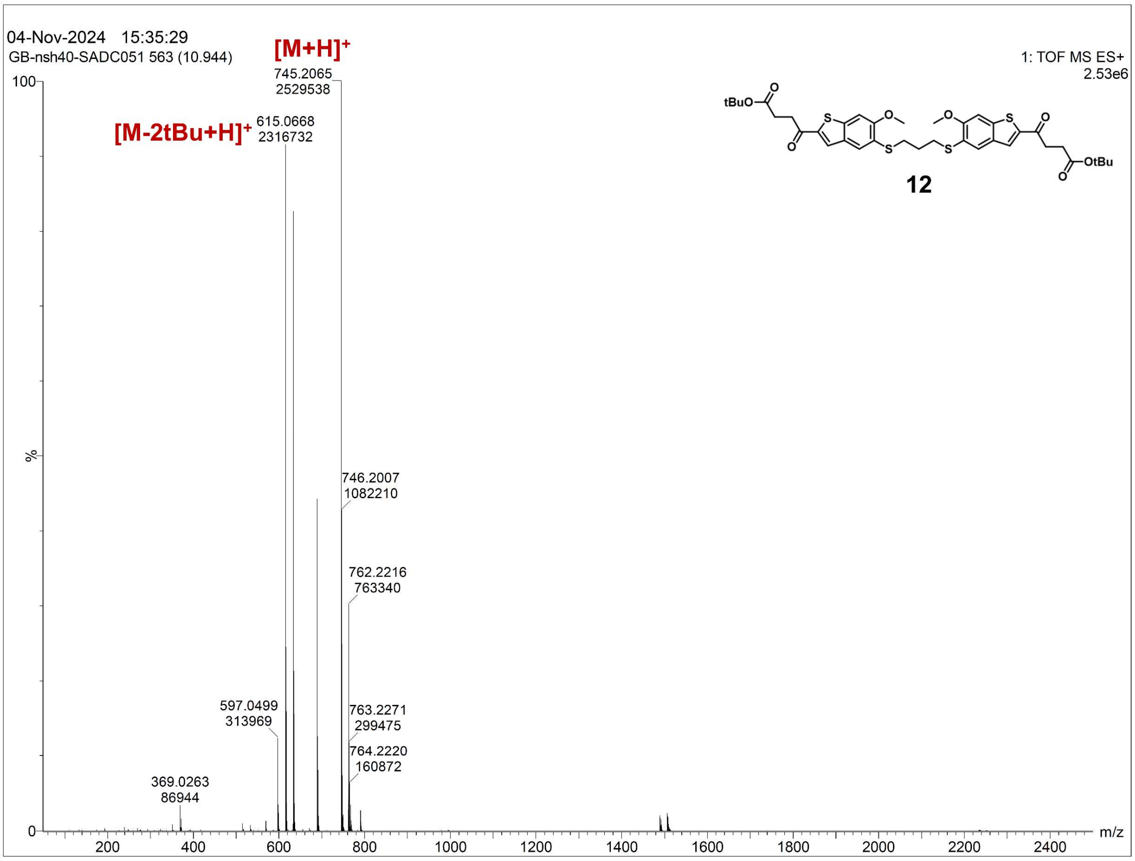
04-Nov-2024 15:35:29
GB-nsh40-SADC051 563 (10.944)
1: TOF MS ES+
2.53e6
[M+H]+
745.2065
2529538
[M-2tBu+H]+
615.0668
2316732
746.2007
1082210
762.2216
763340
597.0499
313969
763.2271
299475
764.2220
160872
369.0263
86944
tBuO
OtBu
12
%
m/z

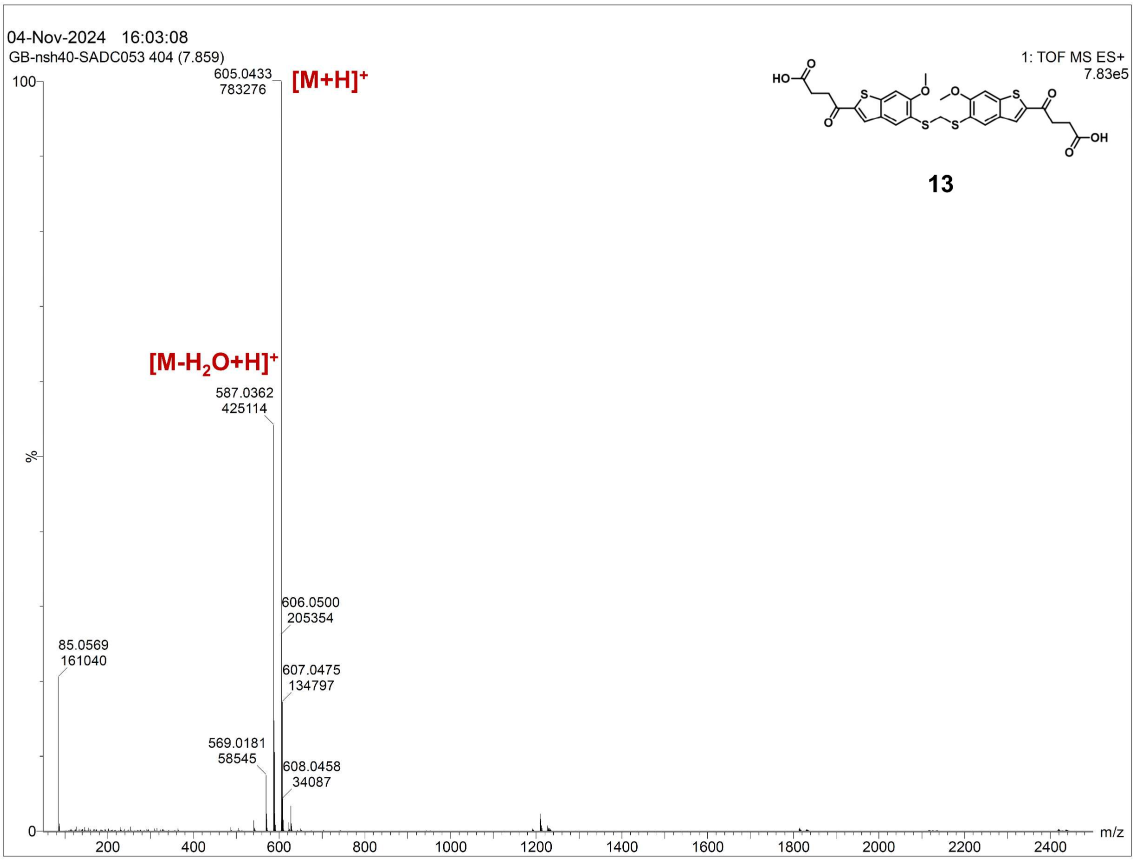

Spectrum Plot Report

Name	S2Cl dimer HPLC purified	Rack Pos.		Instrument	Instrument 1	Operator	Nai-Shu Hsu
Inj. Vol. (ul)	2	Plate Pos.		IRM Status	All ions missed		
Data File	S2Cl dimer HPLC purified1243.d	Method (Acq)	Walkup Positive SM.m	Comment	MBAG/461-RG97836-Task1	Acq. Time (Local)	6/1/2022 7:15:11 PM (UTC+01:00)

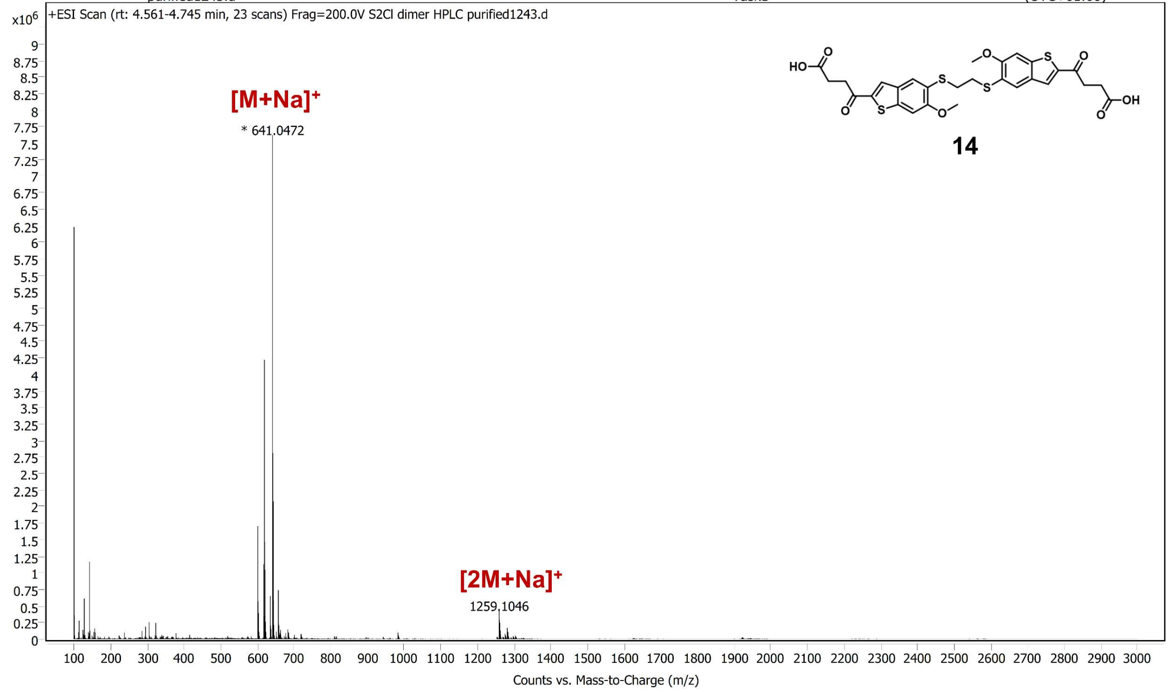

Generated at 8:10 PM on 6/1/2022

Spectrum Plot Report

Name	27 Compound	Rack Pos.		Instrument	Instrument 1	Operator	Nai-Shu Hsu
Inj. Vol. (ul)	10	Plate Pos.		IRM Status	All ions missed		
Data File	27 Compound3796.d	Method (Acq)	Walkup Positive SM.m	Comment	MBAG/546-G100088-Task2	Acq. Time (Local)	10/4/2023 2:54:11 PM (UTC+01:00)

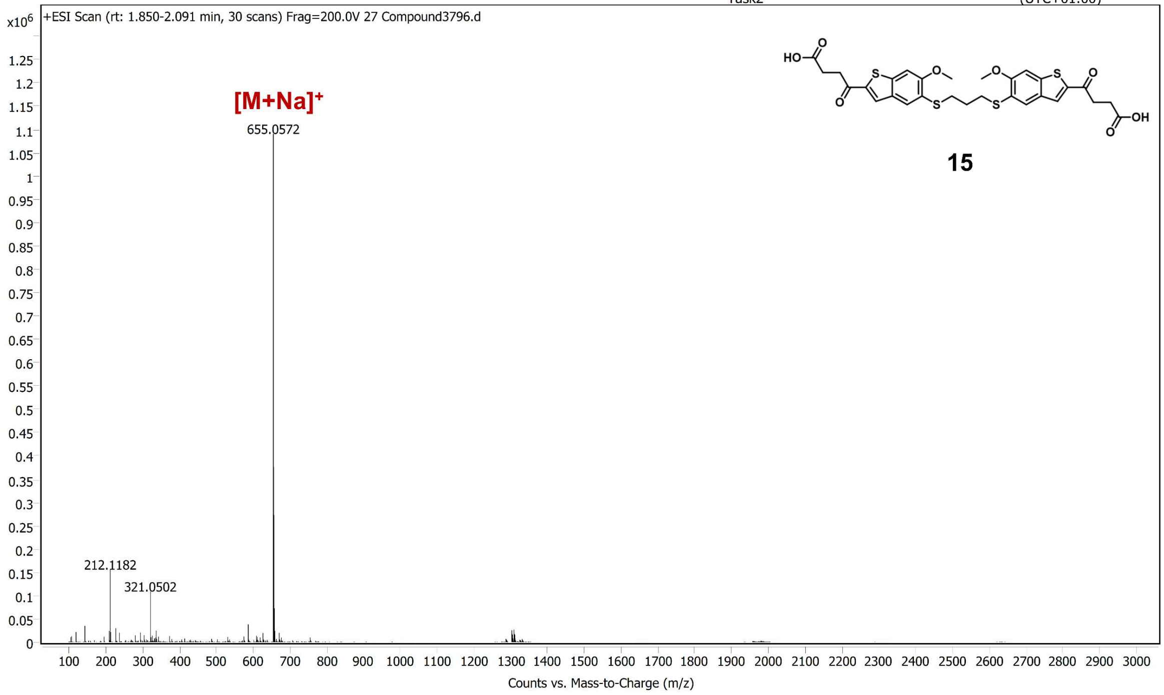

Page 1 of 1

Generated at 3:07 PM on 10/4/2023

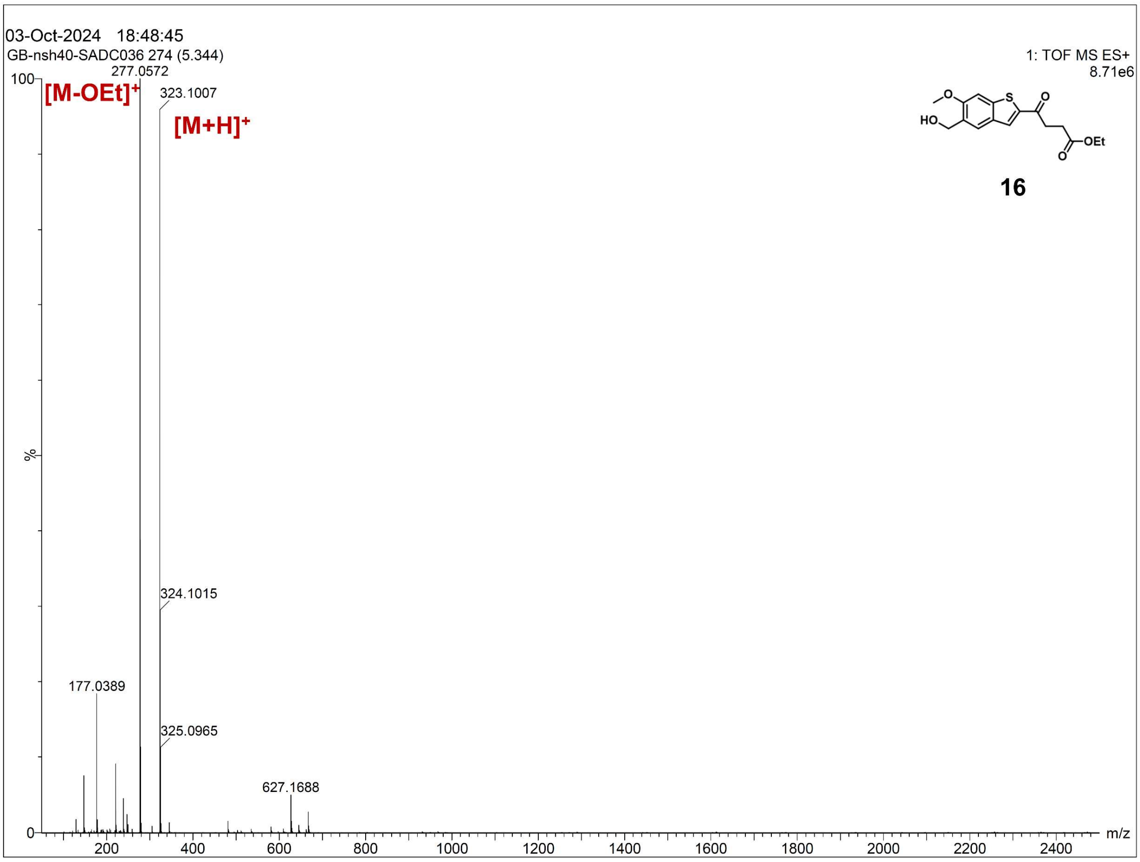

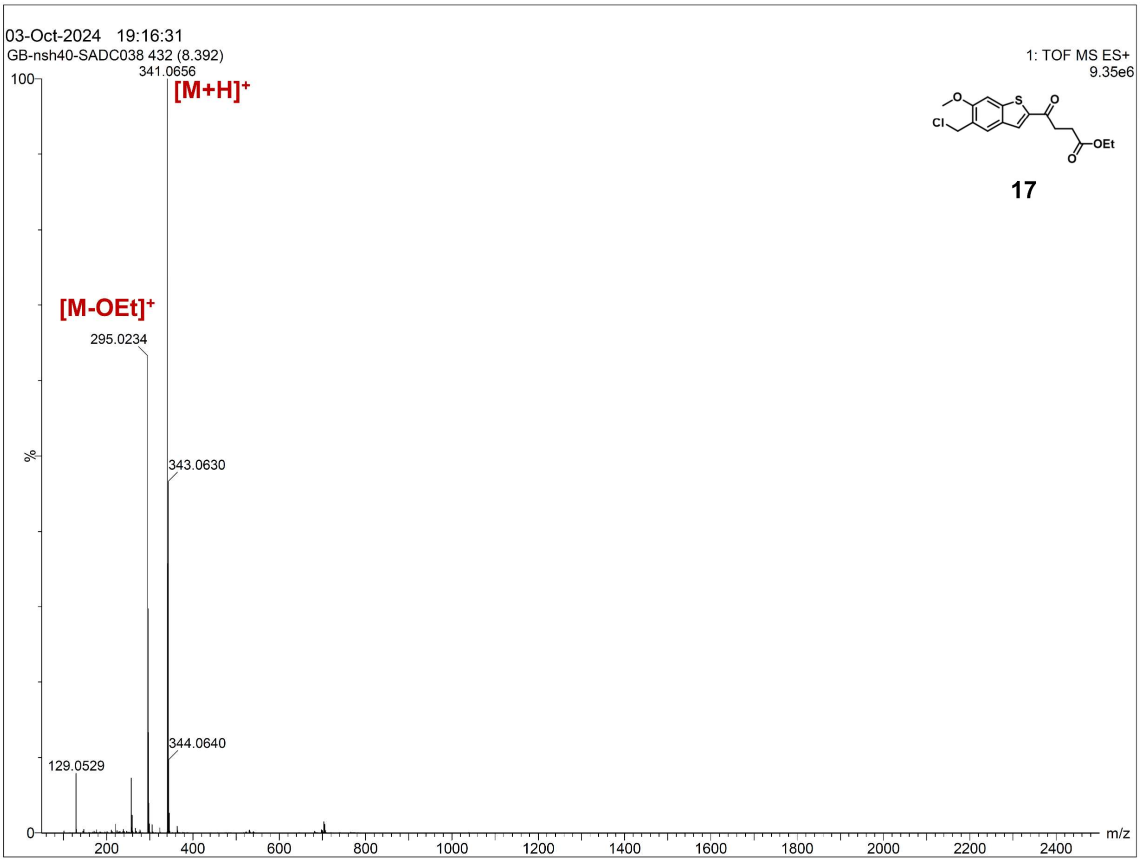
03-Oct-2024 19:16:31
GB-nsh40-SADC038 432 (8.392)
1: TOF MS ES+
9.35e6
341.0656
[M+H]+
[M-OEt]+
295.0234
343.0630
344.0640
129.0529
%
100
0
m/z
200
400
600
800
1000
1200
1400
1600
1800
2000
2200
2400
Cl
O
S
O
O
OEt
17

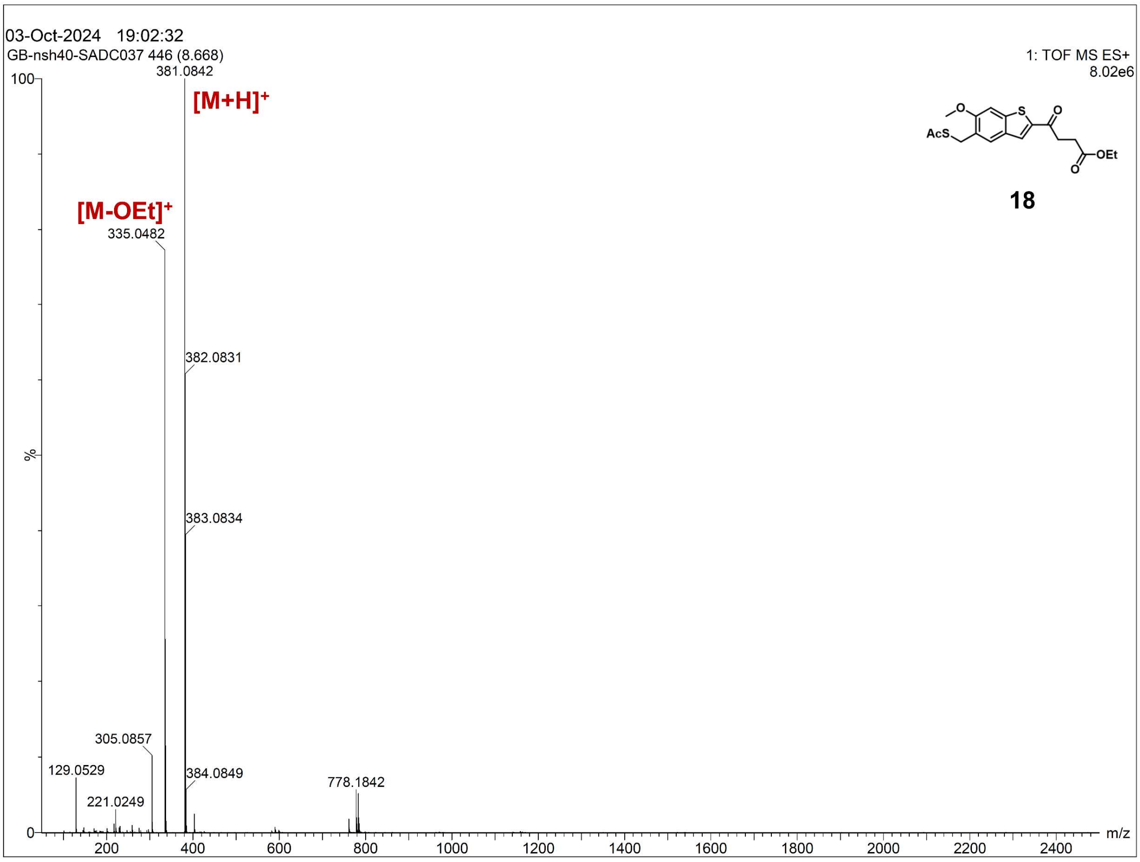
03-Oct-2024 19:02:32
GB-nsh40-SADC037 446 (8.668)
1: TOF MS ES+
8.02e6
381.0842
[M+H]+
[M-OEt]+
335.0482
382.0831
383.0834
305.0857
129.0529
221.0249
384.0849
778.1842
%
100
0
m/z
200
400
600
800
1000
1200
1400
1600
1800
2000
2200
2400
AcS
O
S
O
O
OEt
18

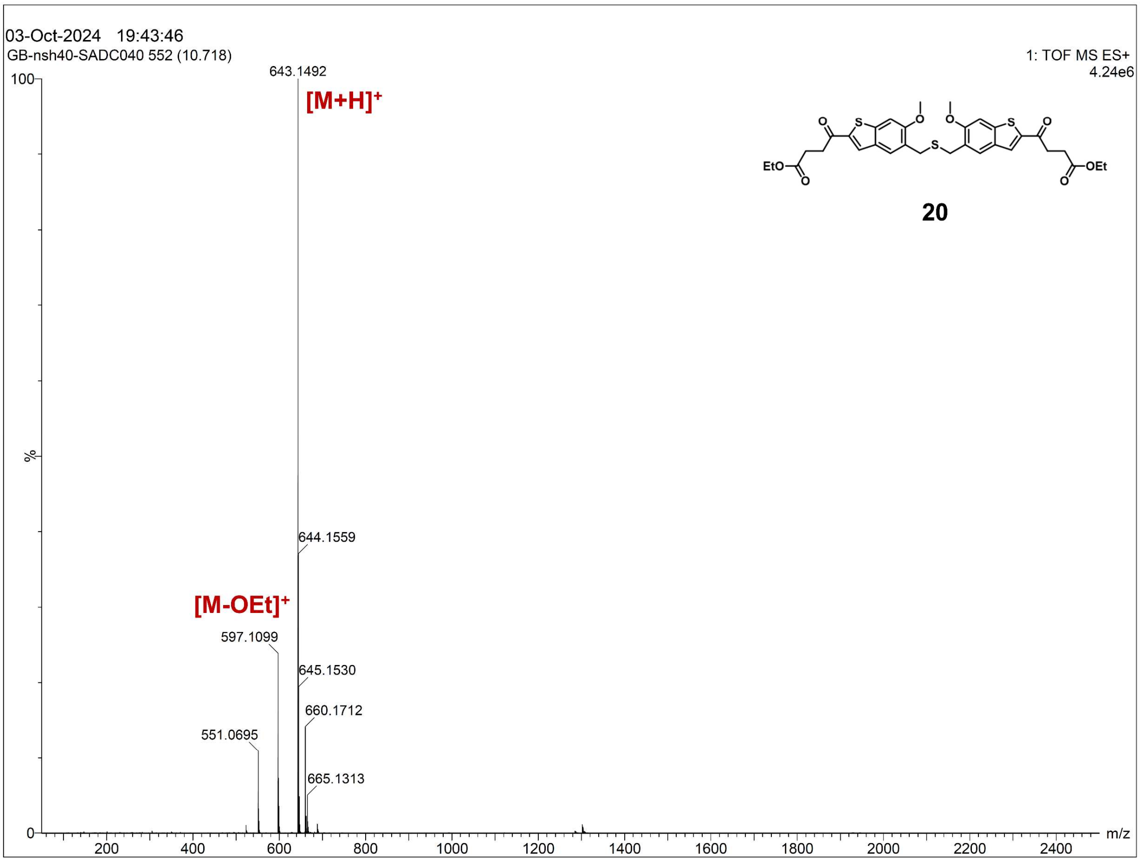
03-Oct-2024 19:43:46
GB-nsh40-SADC040 552 (10.718)
1: TOF MS ES+
4.24e6
643.1492
[M+H]+
644.1559
[M-OEt]+
597.1099
645.1530
660.1712
551.0695
665.1313
100
%
0
m/z
200
400
600
800
1000
1200
1400
1600
1800
2000
2200
2400
EtO
OEt
S
O
20

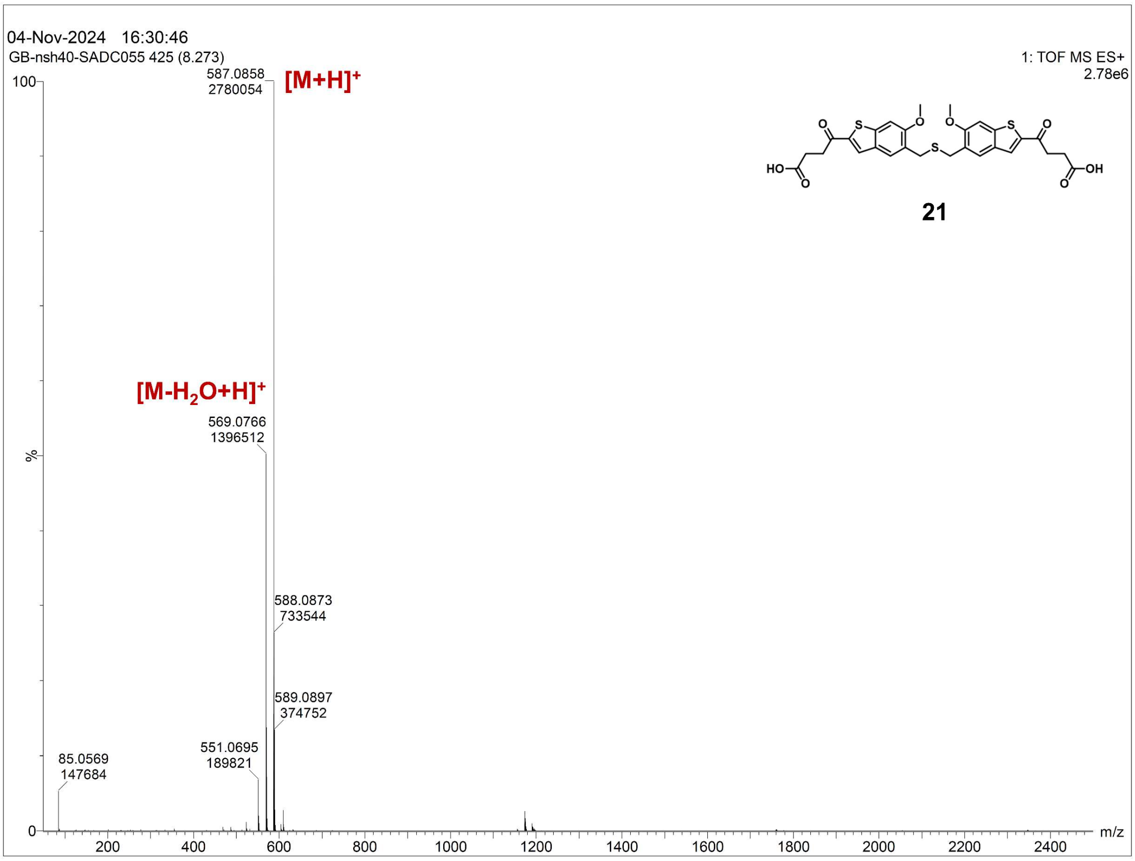
04-Nov-2024 16:30:46
GB-nsh40-SADC055 425 (8.273)
1: TOF MS ES+
2.78e6
587.0858
2780054
[M+H]+
[M-H2O+H]+
569.0766
1396512
588.0873
733544
589.0897
374752
85.0569
147684
551.0695
189821
100
%
0
m/z
200
400
600
800
1000
1200
1400
1600
1800
2000
2200
2400
HO
OH
S
O
21

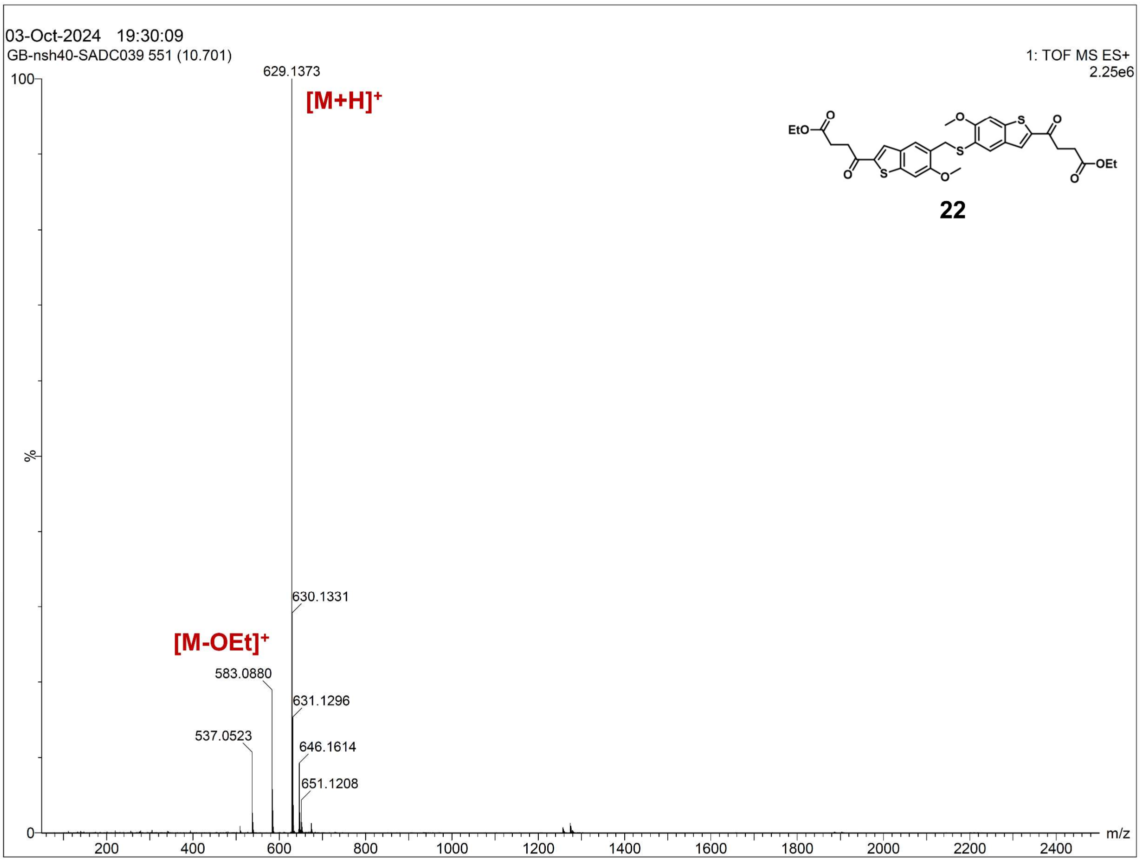
03-Oct-2024 19:30:09
GB-nsh40-SADC039 551 (10.701)
1: TOF MS ES+
2.25e6
629.1373
[M+H]+
630.1331
[M-OEt]+
583.0880
631.1296
537.0523
646.1614
651.1208
%
m/z
EtO
OEt
S
22

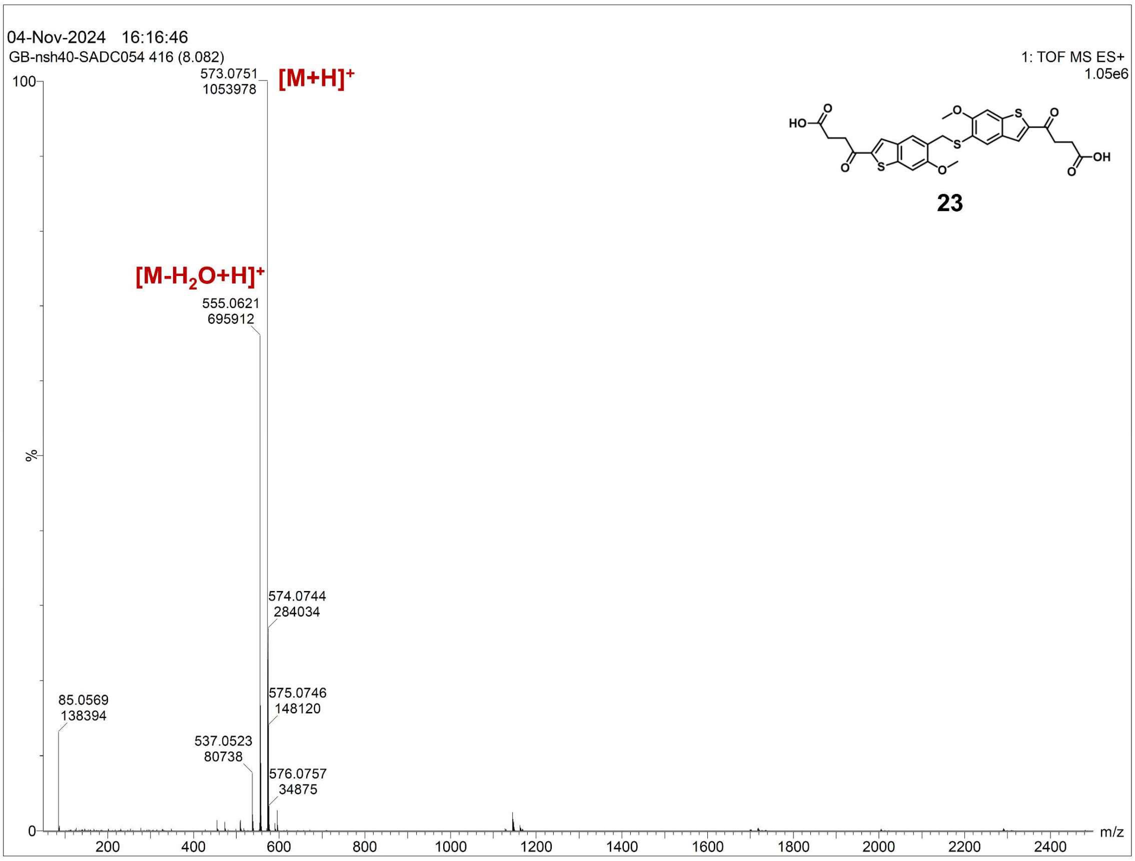
04-Nov-2024 16:16:46
GB-nsh40-SADC054 416 (8.082)
1: TOF MS ES+
1.05e6
573.0751
1053978
[M+H]+
[M-H2O+H]+
555.0621
695912
574.0744
284034
85.0569
138394
575.0746
148120
537.0523
80738
576.0757
34875
%
m/z
HO
OH
S
23

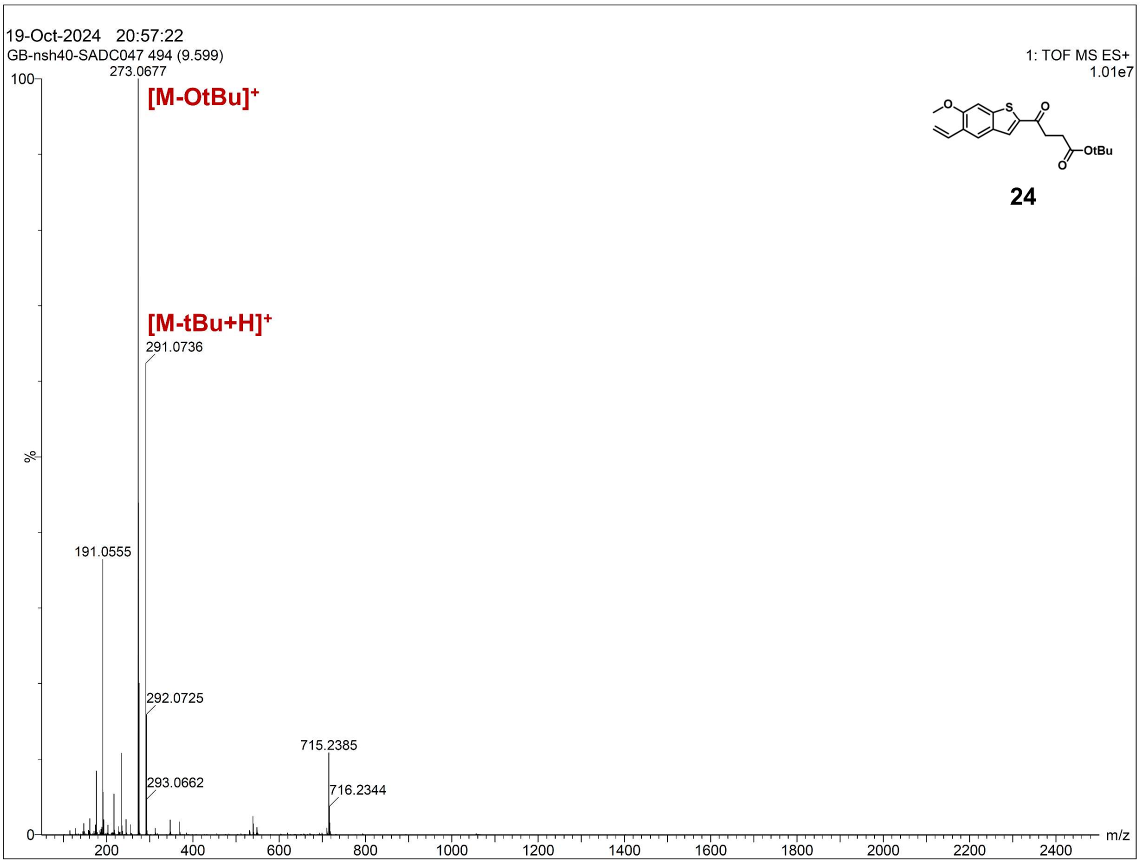
19-Oct-2024 20:57:22
GB-nsh40-SADC047 494 (9.599)
1: TOF MS ES+
1.01e7
273.0677
[M-OtBu]+
[M-tBu+H]+
291.0736
191.0555
292.0725
293.0662
715.2385
716.2344
%
m/z
OtBu
24

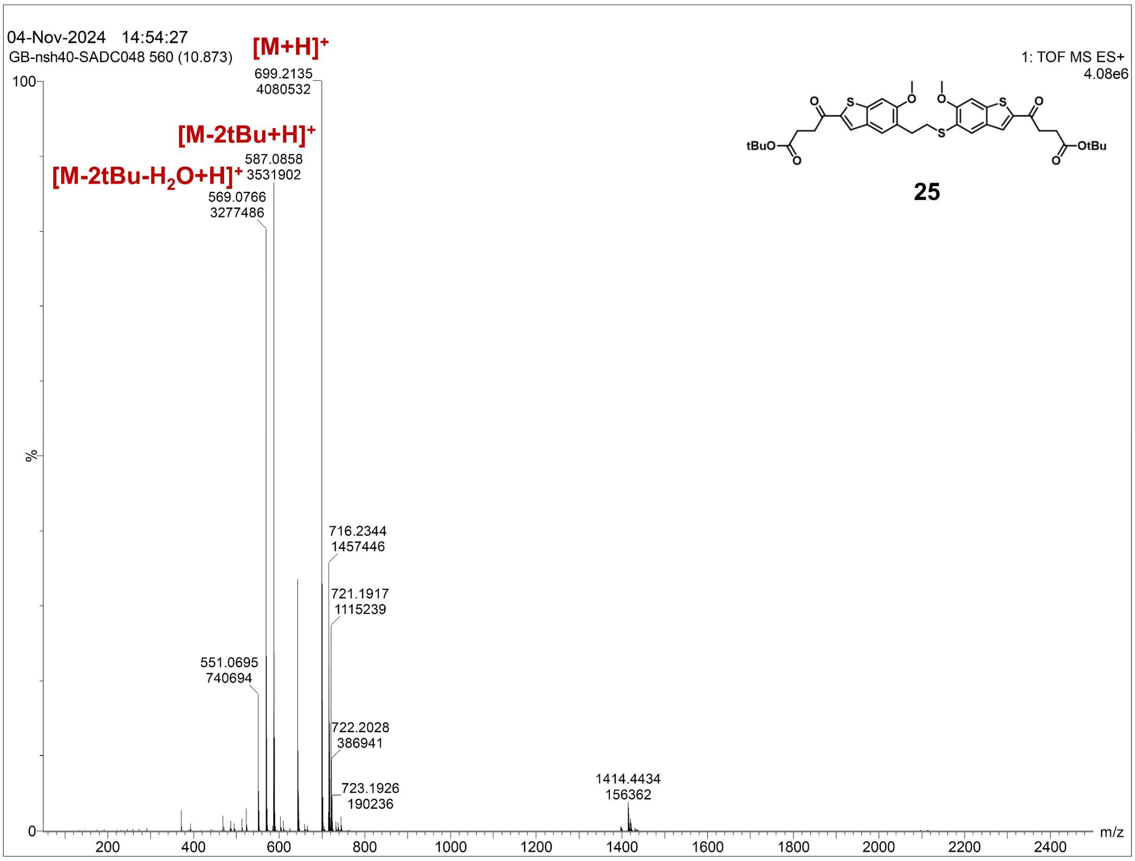
04-Nov-2024 14:54:27
GB-nsh40-SADC048 560 (10.873)
[M+H]+
699.2135
4080532
1: TOF MS ES+
4.08e6
[M-2tBu+H]+
587.0858
3531902
[M-2tBu-H2O+H]+
569.0766
3277486
716.2344
1457446
721.1917
1115239
551.0695
740694
722.2028
386941
723.1926
190236
1414.4434
156362
%
m/z
tBuO
OtBu
25

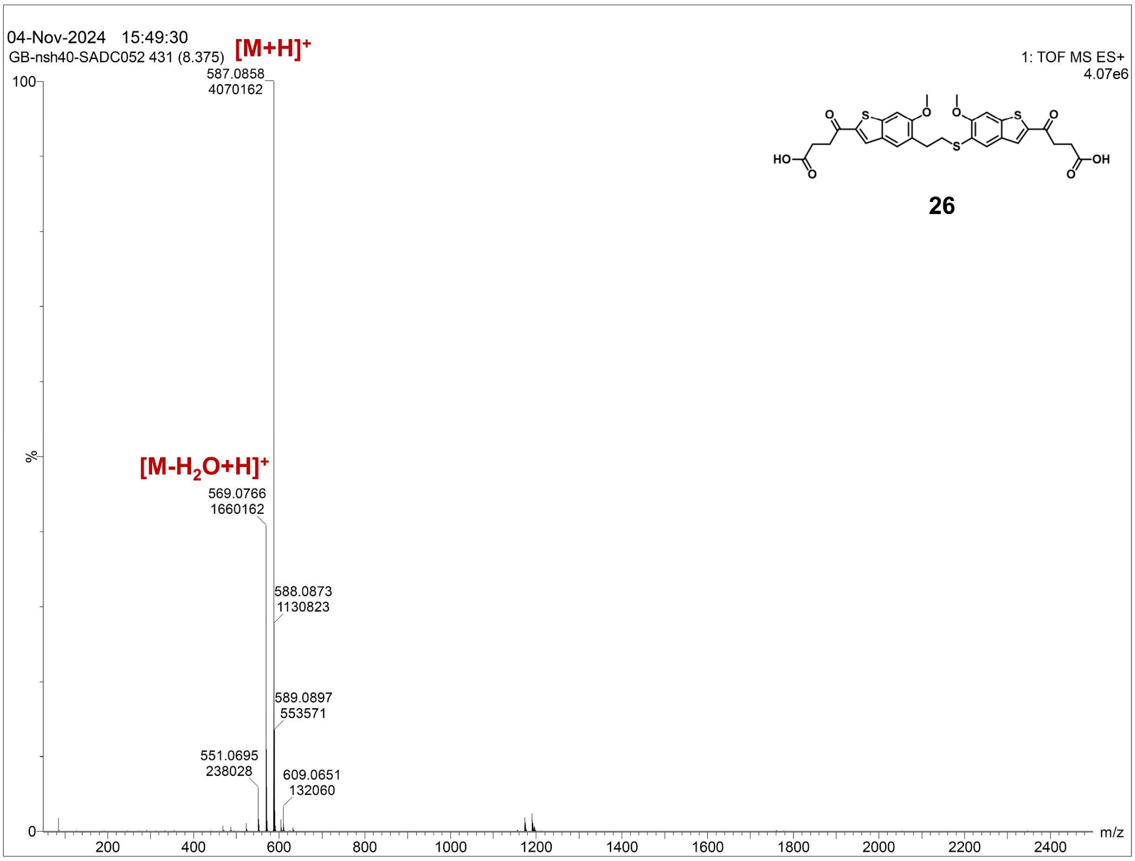
04-Nov-2024 15:49:30
GB-nsh40-SADC052 431 (8.375)
[M+H]+
587.0858
4070162
1: TOF MS ES+
4.07e6
26
[M-H2O+H]+
569.0766
1660162
588.0873
1130823
589.0897
553571
551.0695
238028
609.0651
132060
%
m/z

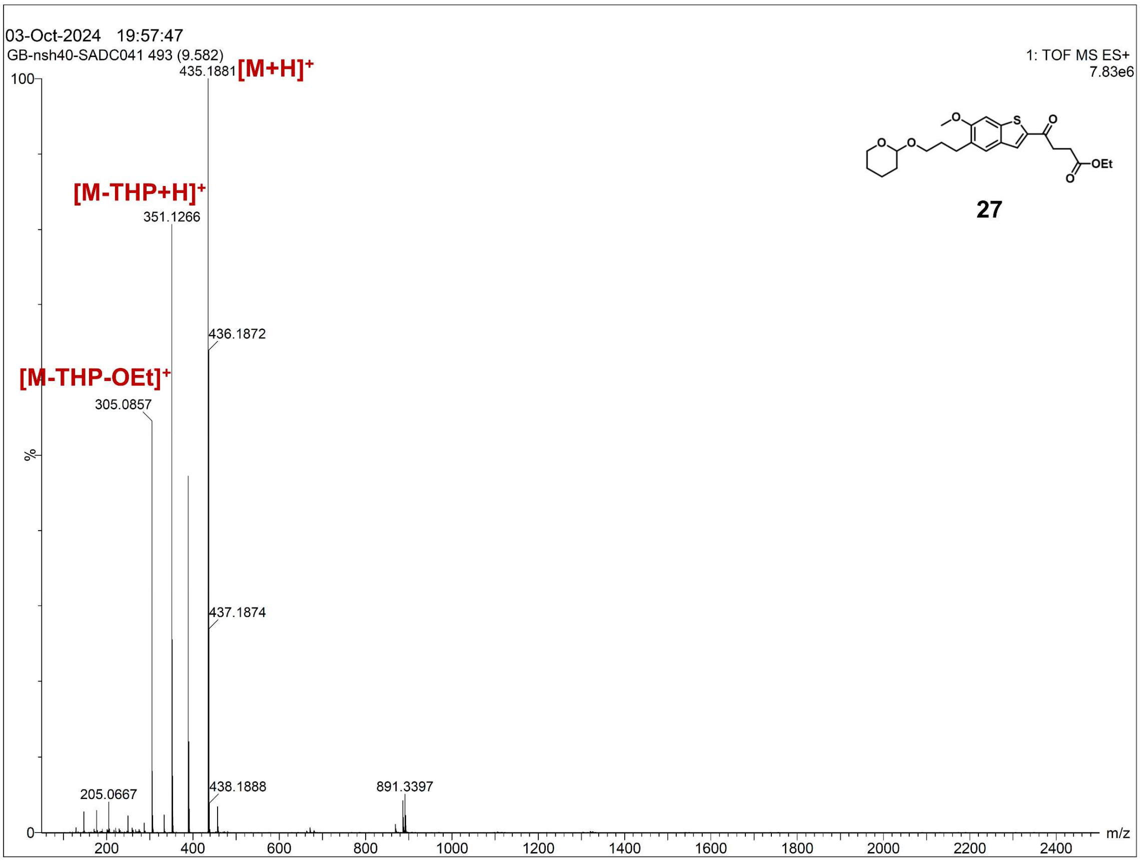
03-Oct-2024 19:57:47
GB-nsh40-SADC041 493 (9.582)
435.1881
[M+H]+
1: TOF MS ES+
7.83e6
27
[M-THP+H]+
351.1266
436.1872
[M-THP-OEt]+
305.0857
437.1874
205.0667
438.1888
891.3397
%
m/z

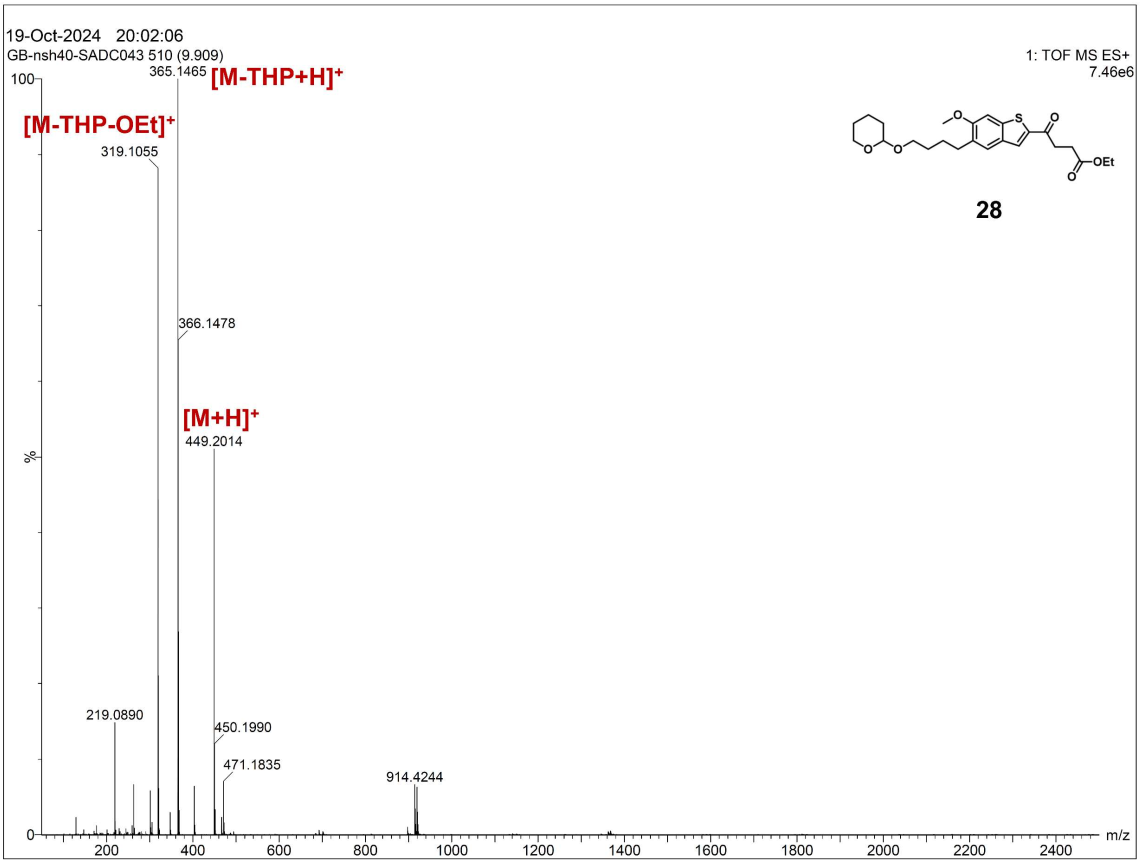
19-Oct-2024 20:02:06
GB-nsh40-SADC043 510 (9.909)
1: TOF MS ES+
7.46e6
365.1465 [M-THP+H]+
[M-THP-OEt]+
319.1055
366.1478
[M+H]+
449.2014
219.0890
450.1990
471.1835
914.4244
OEt
28
m/z

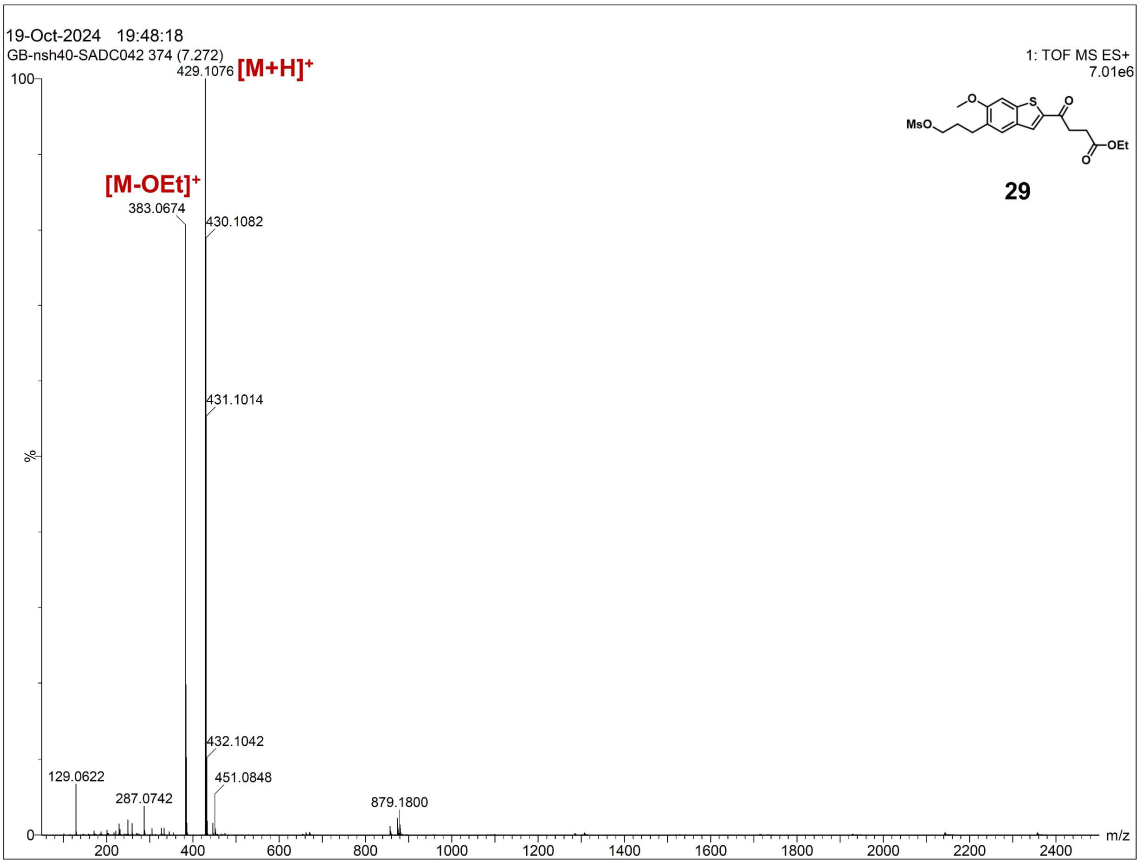
19-Oct-2024 19:48:18
GB-nsh40-SADC042 374 (7.272)
1: TOF MS ES+
7.01e6
429.1076 [M+H]+
[M-OEt]+
383.0674
430.1082
431.1014
432.1042
129.0622
451.0848
287.0742
879.1800
MsO
OEt
29
m/z

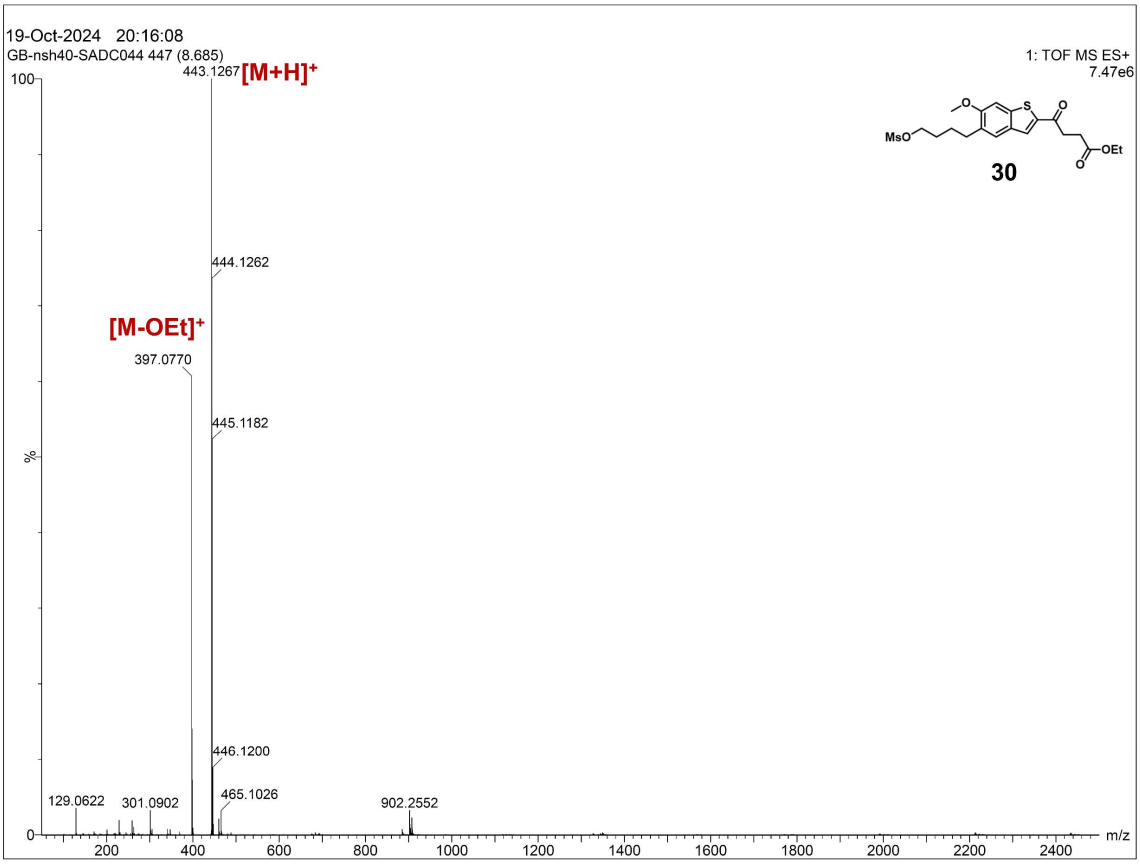
19-Oct-2024 20:16:08
GB-nsh40-SADC044 447 (8.685)
1: TOF MS ES+
7.47e6
100
%
0
443.1267 [M+H]+
444.1262
[M-OEt]+
397.0770
445.1182
446.1200
129.0622
301.0902
465.1026
902.2552
200
400
600
800
1000
1200
1400
1600
1800
2000
2200
2400
m/z
MsO
S
O
OEt
30

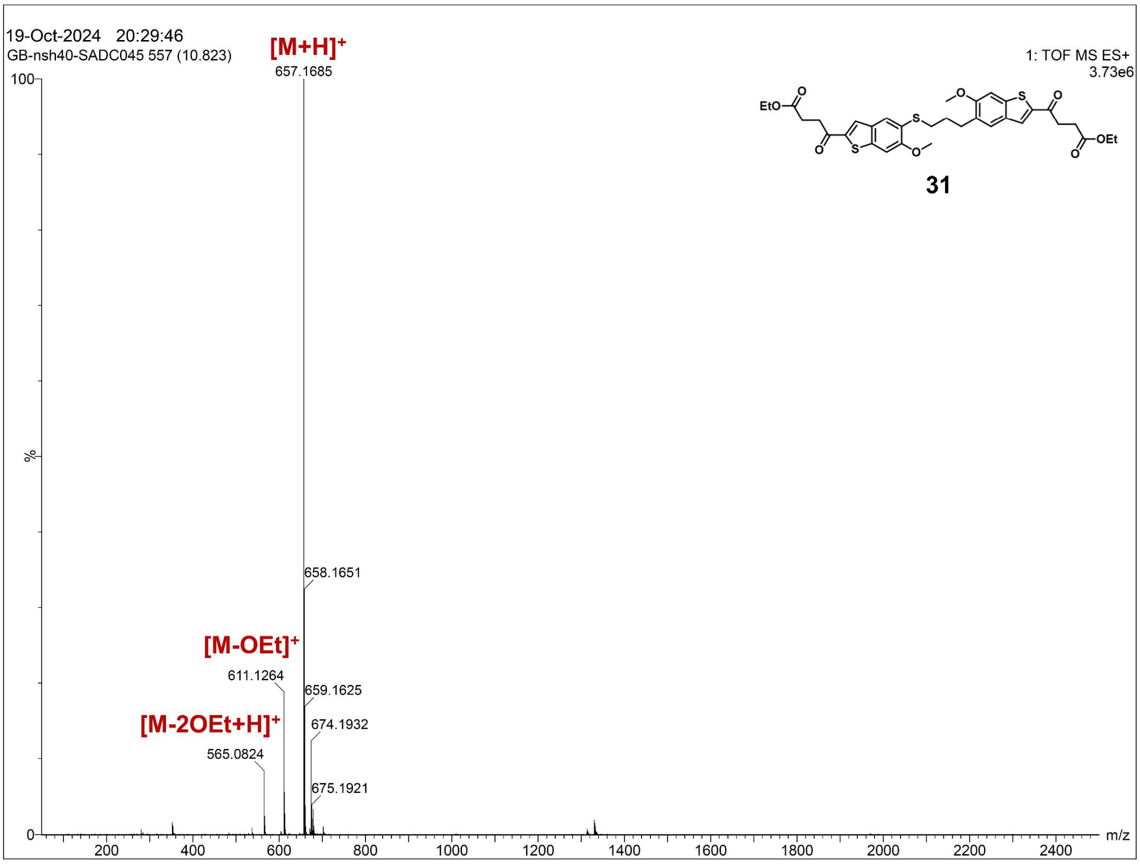
19-Oct-2024 20:29:46
GB-nsh40-SADC045 557 (10.823)
[M+H]+
657.1685
1: TOF MS ES+
3.73e6
100
%
0
658.1651
[M-OEt]+
611.1264
659.1625
[M-2OEt+H]+
674.1932
565.0824
675.1921
200
400
600
800
1000
1200
1400
1600
1800
2000
2200
2400
m/z
EtO
S
O
OEt
31

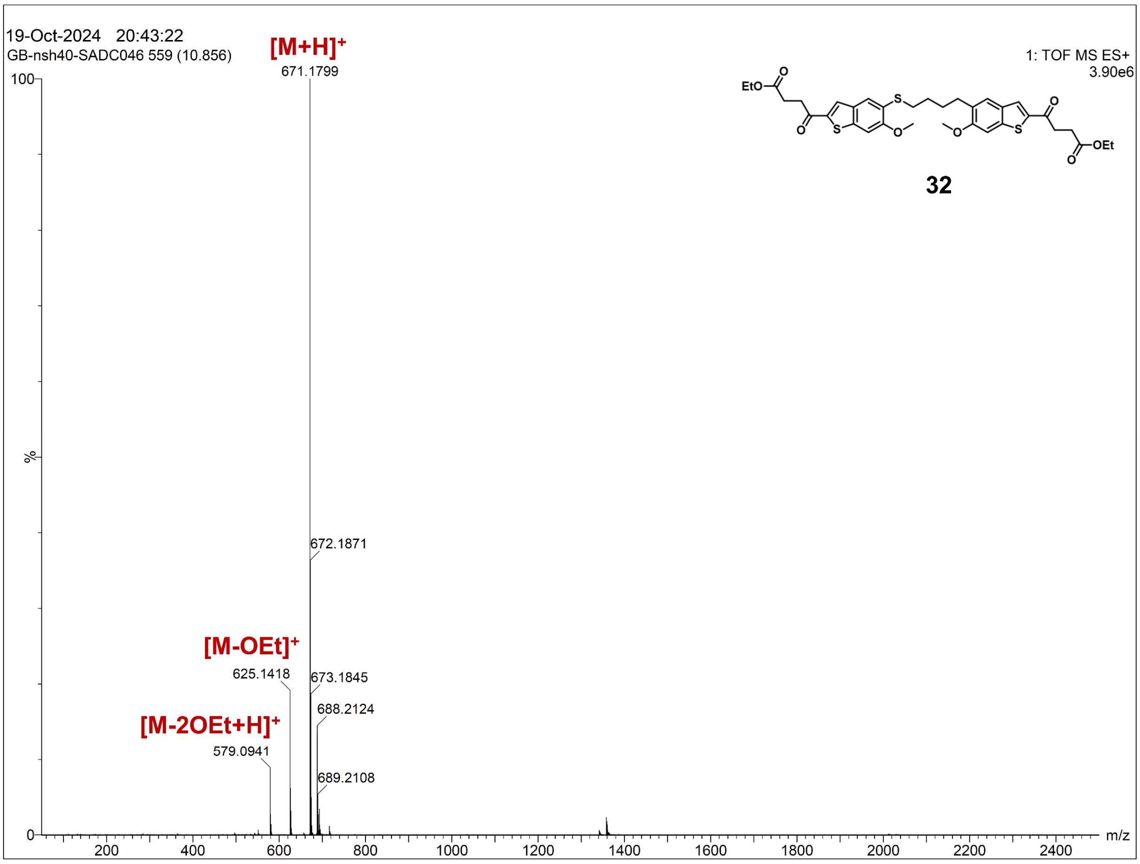
19-Oct-2024 20:43:22
GB-nsh40-SADC046 559 (10.856)
[M+H]+
671.1799
1: TOF MS ES+
3.90e6
EtO
OEt
32
100
%
672.1871
[M-OEt]+
625.1418
673.1845
688.2124
[M-2OEt+H]+
579.0941
689.2108
0
200
400
600
800
1000
1200
1400
1600
1800
2000
2200
2400
m/z

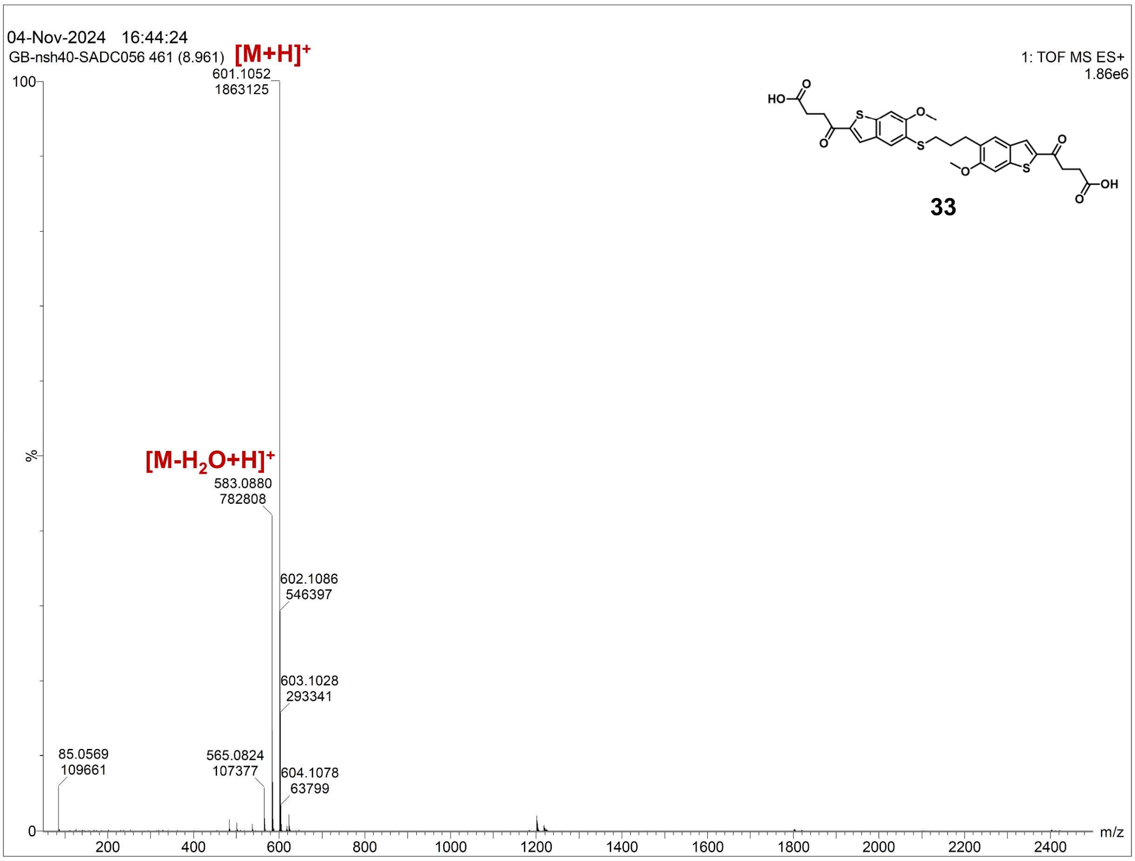
04-Nov-2024 16:44:24
GB-nsh40-SADC056 461 (8.961)
[M+H]+
601.1052
1863125
1: TOF MS ES+
1.86e6
HO
OH
33
100
%
[M-H2O+H]+
583.0880
782808
602.1086
546397
603.1028
293341
85.0569
109661
565.0824
107377
604.1078
63799
0
200
400
600
800
1000
1200
1400
1600
1800
2000
2200
2400
m/z

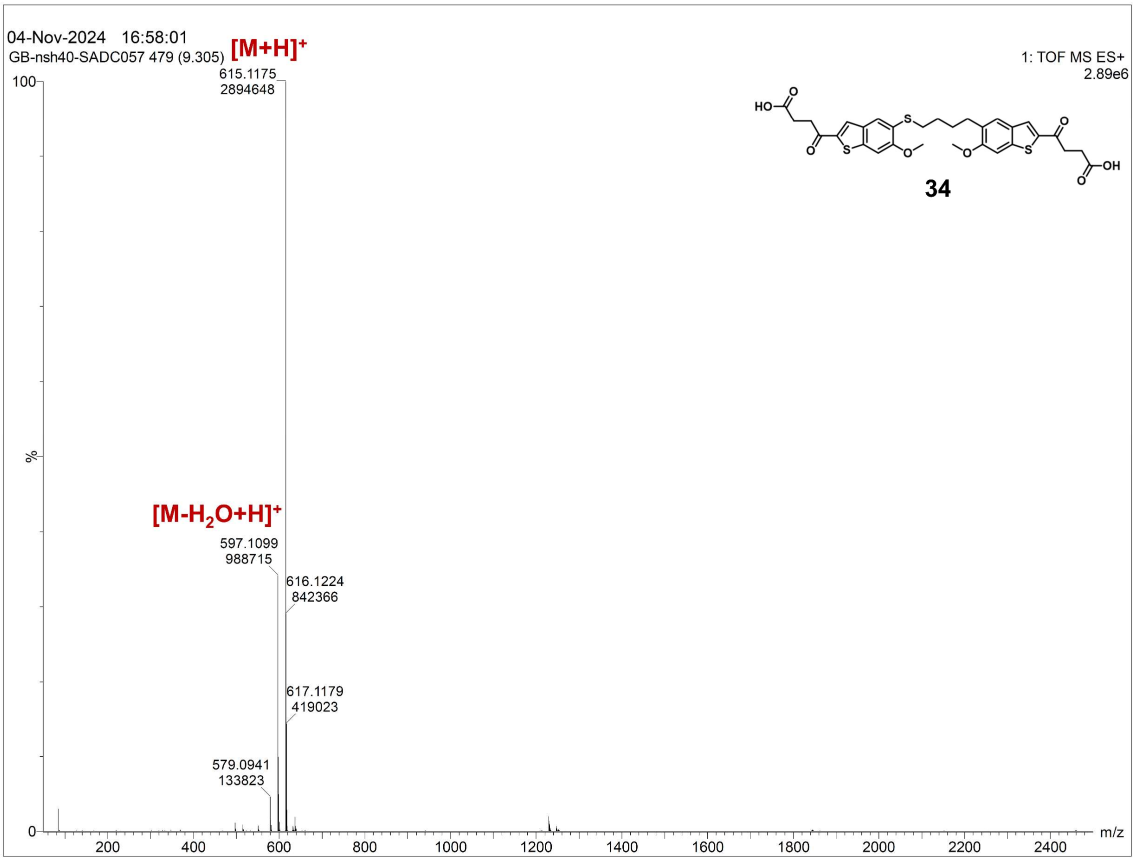

Spectrum Plot Report

Agilent | Trusted Answers

Name	az to aniline purified	Rack Pos.		Instrument	Instrument 1	Operator	Nai-Shu Hsu
Inj. Vol. (ul)	2	Plate Pos.		IRM Status	All ions missed		
Data File	az to aniline purified0618.d	Method (Acq)	Walkup Positive SM.m	Comment	MBAG/461-RG97836-Task1	Acq. Time (Local)	1/31/2022 9:27:18 PM (UTC+00:00)

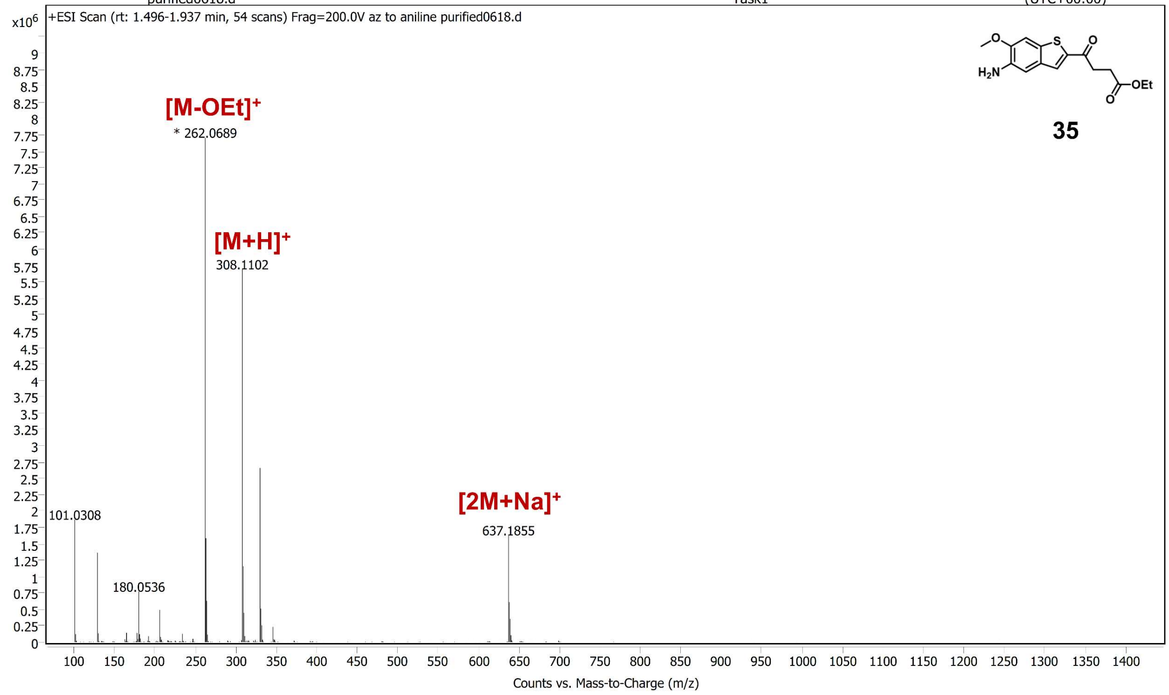

Spectrum Plot Report

Name	acryl amide Et esterMSA2	Rack Pos.		Instrument	Instrument 1	Operator	Nai-Shu Hsu
Inj. Vol. (ul)	2	Plate Pos.		IRM Status	All ions missed		
Data File	acryl amide Et esterMSA20622.d	Method (Acq)	Walkup Positive SM.m	Comment	MBAG/461-RG97836-Task1	Acq. Time (Local)	2/3/2022 10:30:56 PM (UTC+00:00)

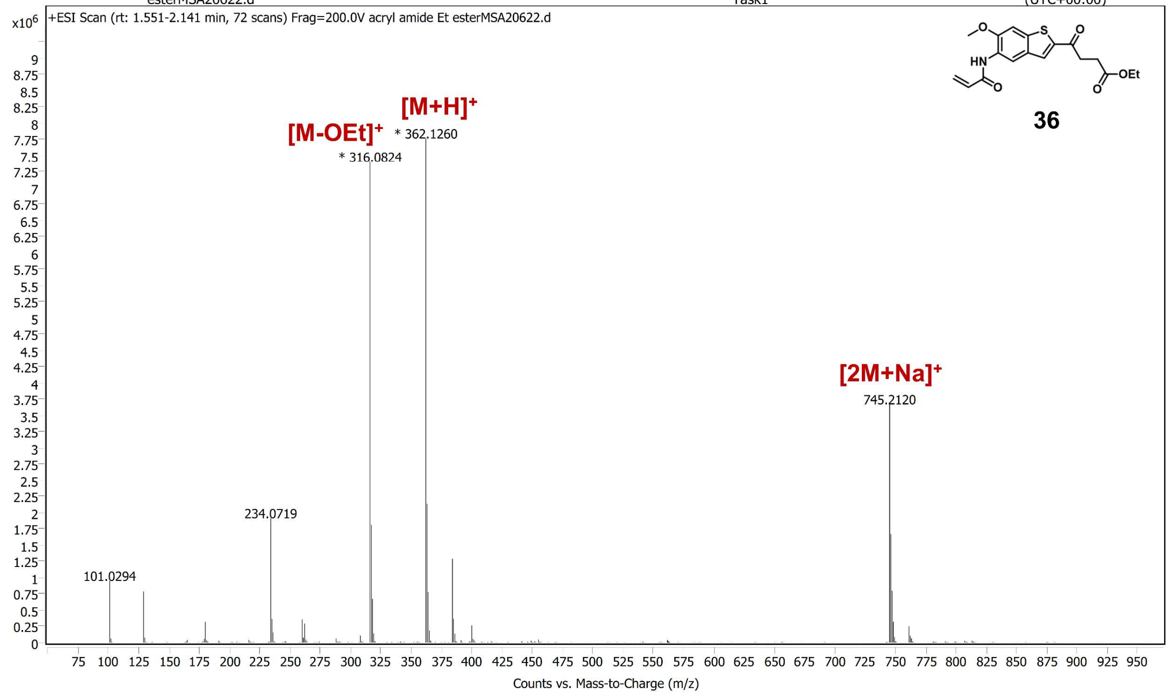

Page 1 of 1 Generated at 4:49 PM on 2/5/2022

Spectrum Plot Report

Name	MSA2EtSulfonamide	Rack Pos.		Instrument	Instrument 1	Operator	Nai-Shu Hsu
Inj. Vol. (ul)	2	Plate Pos.		IRM Status	Success		
Data File	MSA2EtSulfonamide0641.d	Method (Acq)	Walkup Positive SM.m	Comment	MBAG/461-RG97836-Task1	Acq. Time (Local)	2/9/2022 9:56:35 PM (UTC+00:00)

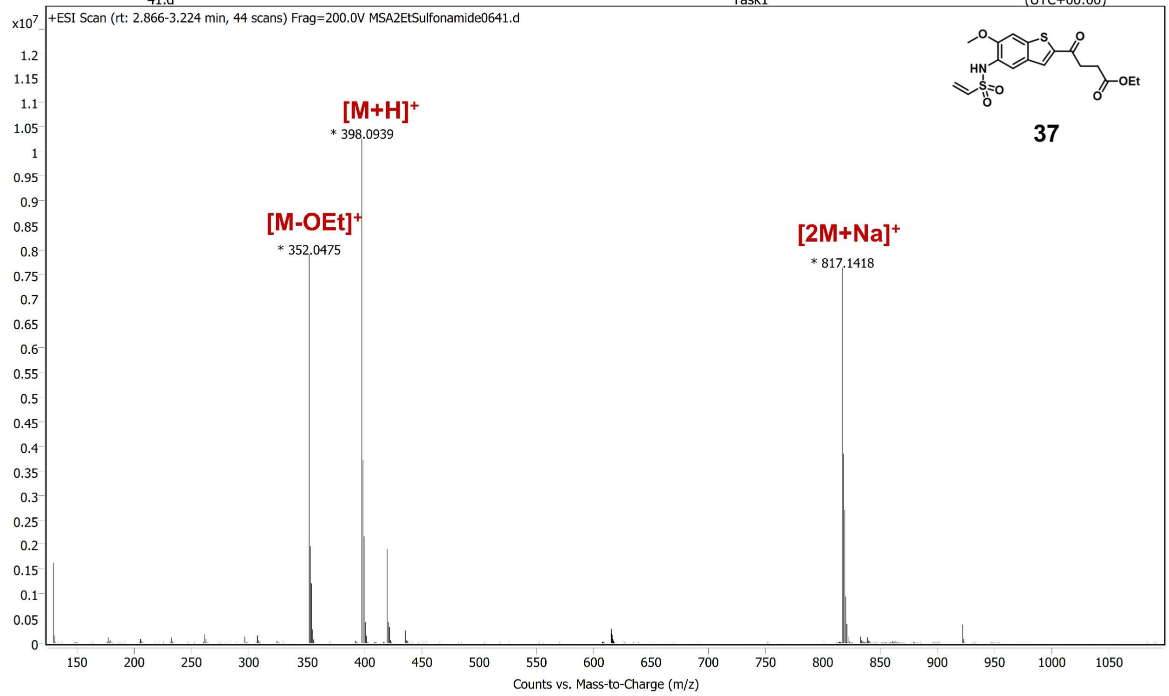

Page 1 of 1 Generated at 8:58 PM on 2/18/2022

Spectrum Plot Report

Name	MSA2-N-acryl	Rack Pos.		Instrument	Instrument 1	Operator	Nai-Shu Hsu
Inj. Vol. (ul)	2	Plate Pos.		IRM Status	All ions missed		
Data File	MSA2-N-acryl0634.d	Method (Acq)	Walkup Positive SM.m	Comment	MBAG/461-RG97836-Task1	Acq. Time (Local)	2/5/2022 4:41:23 PM (UTC+00:00)

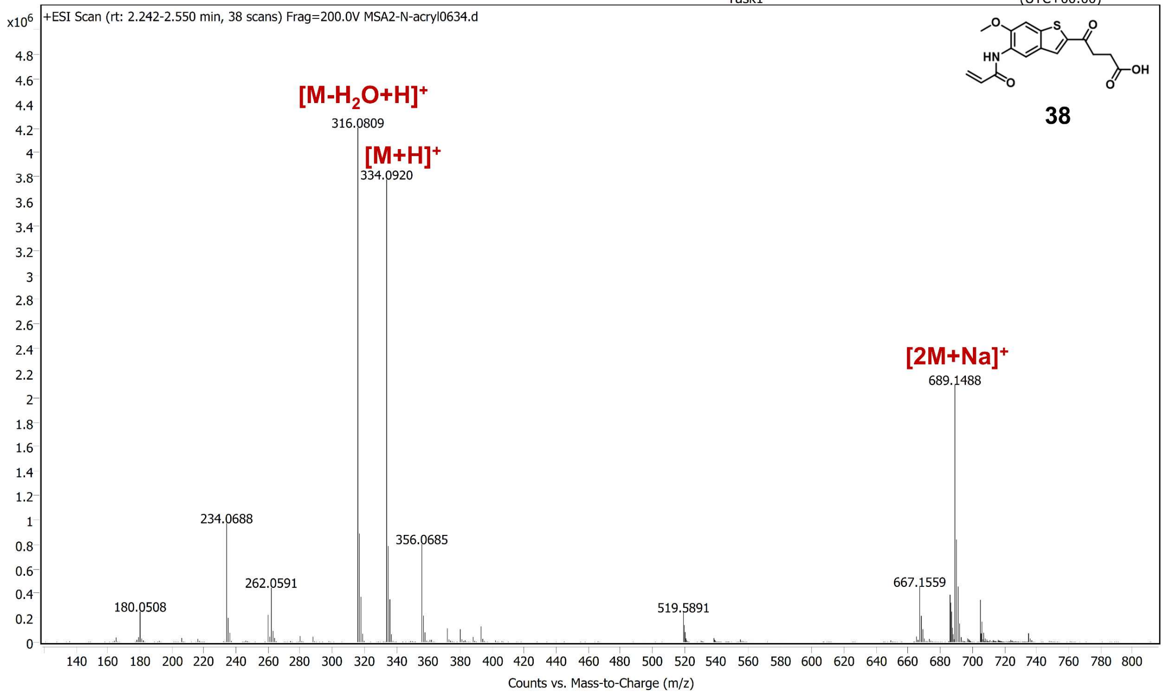

Generated at 4:58 PM on 2/5/2022

Spectrum Plot Report

Name	msa2-sulfonamide	Rack Pos.		Instrument	Instrument 1	Operator	Nai-Shu Hsu
Inj. Vol. (ul)	2	Plate Pos.		IRM Status	Success		
Data File	msa2-sulfonamide0657.d	Method (Acq)	Walkup Positive SM.m	Comment	MBAG/461-RG97836-Task1	Acq. Time (Local)	2/11/2022 10:53:58 PM (UTC+00:00)

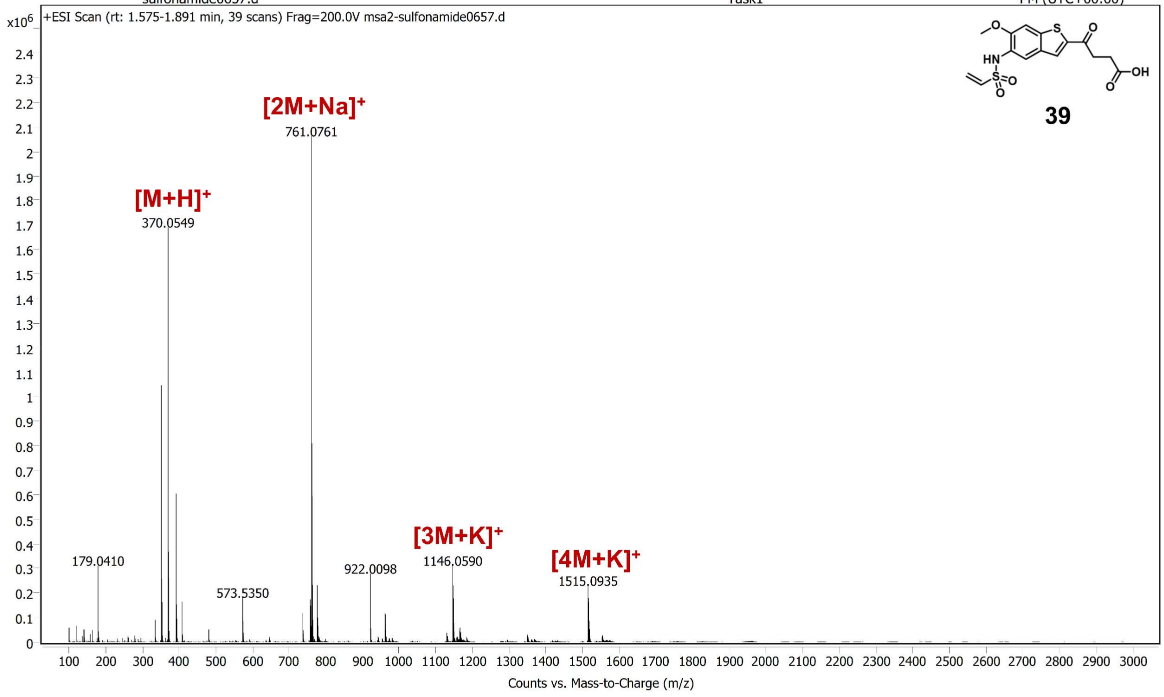

Generated at 8:39 PM on 2/18/2022

Spectrum Plot Report

Name	SAc Purified 0121	Rack Pos.		Instrument	Instrument 1	Operator	Nai-Shu Hsu
Inj. Vol. (ul)	2	Plate Pos.		IRM Status	All ions missed		
Data File	SAc Purified 01210574.d	Method (Acq)	Walkup Positive SM.m	Comment	MBAG/461-RG97836-Task1	Acq. Time (Local)	1/21/2022 5:22:12 PM (UTC+00:00)

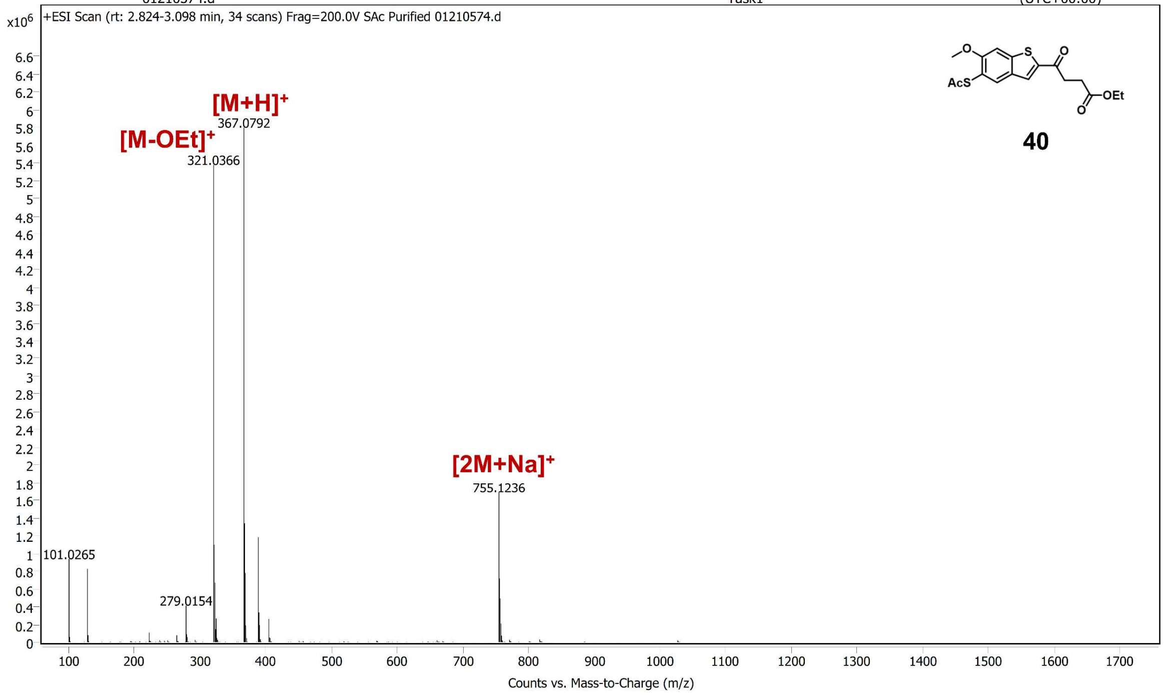

Generated at 5:09 PM on 2/5/2022

Spectrum Plot Report

Name	MSA2-S-Cl	Rack Pos.		Instrument	Instrument 1	Operator	Nai-Shu Hsu
Inj. Vol. (ul)	2	Plate Pos.		IRM Status	Success		
Data File	MSA2-S-Cl0989.d	Method (Acq)	Walkup Positive SM.m	Comment	MBAG/461-RG97836-Task1	Acq. Time (Local)	4/29/2022 10:36:18 AM (UTC+01:00)

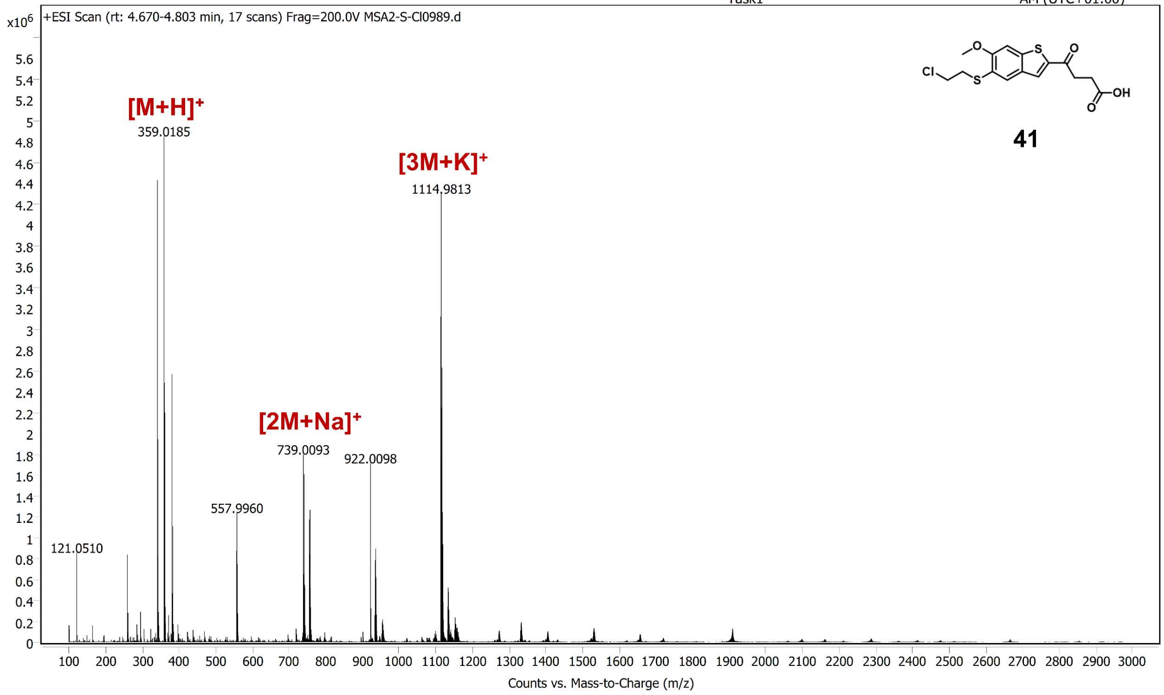

Generated at 6:59 PM on 4/29/2022

Spectrum Plot Report

Agilent Trusted Answers

Name	25-Compound	Rack Pos.		Instrument	Instrument 1	Operator	Nai-Shu Hsu
Inj. Vol. (ul)	3	Plate Pos.		IRM Status	All ions missed		
Data File	25-Compound3801.d	Method (Acq)	Walkup Positive SM.m	Comment	MBAG/546-G100088-Task2	Acq. Time (Local)	10/4/2023 6:11:41 PM (UTC+01:00)

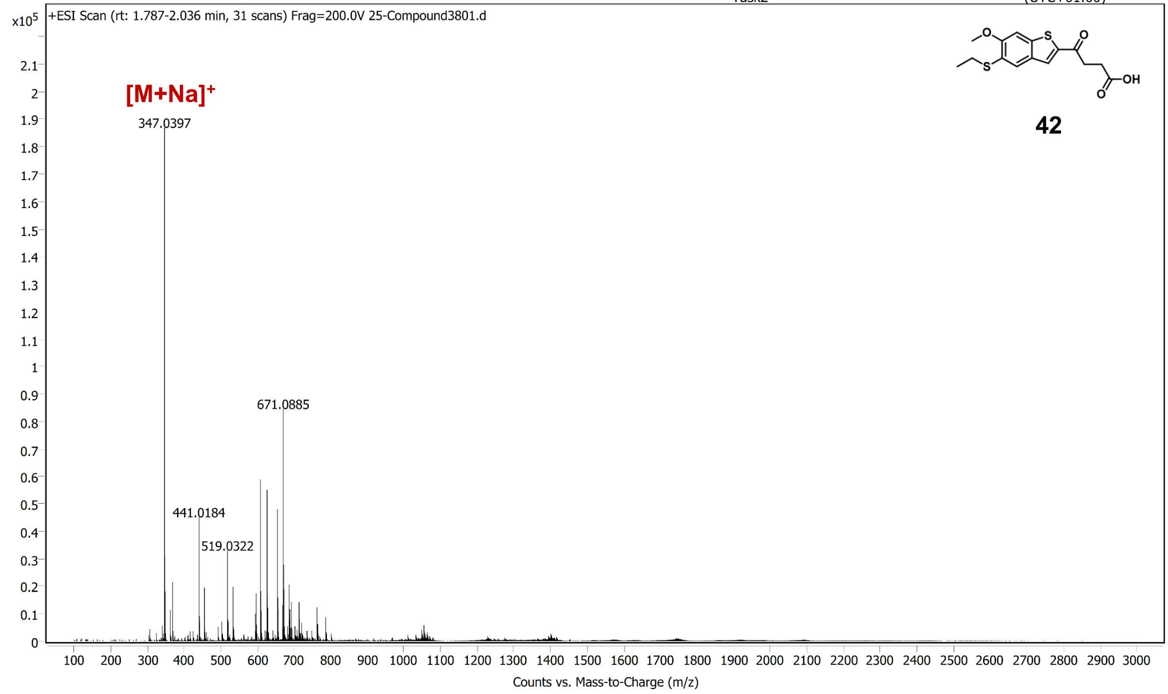

 Generated at 6:35 PM on 10/5/2023

Spectrum Plot Report

Agilent Trusted Answers

Name	Thio MSA-2	Rack Pos.		Instrument	Instrument 1	Operator	Nai-Shu Hsu
Inj. Vol. (ul)	2	Plate Pos.		IRM Status	All ions missed		
Data File	Thio MSA-20576.d	Method (Acq)	Walkup Positive SM.m	Comment	MBAG/461-RG97836-Task1	Acq. Time (Local)	1/22/2022 8:30:47 PM (UTC+00:00)

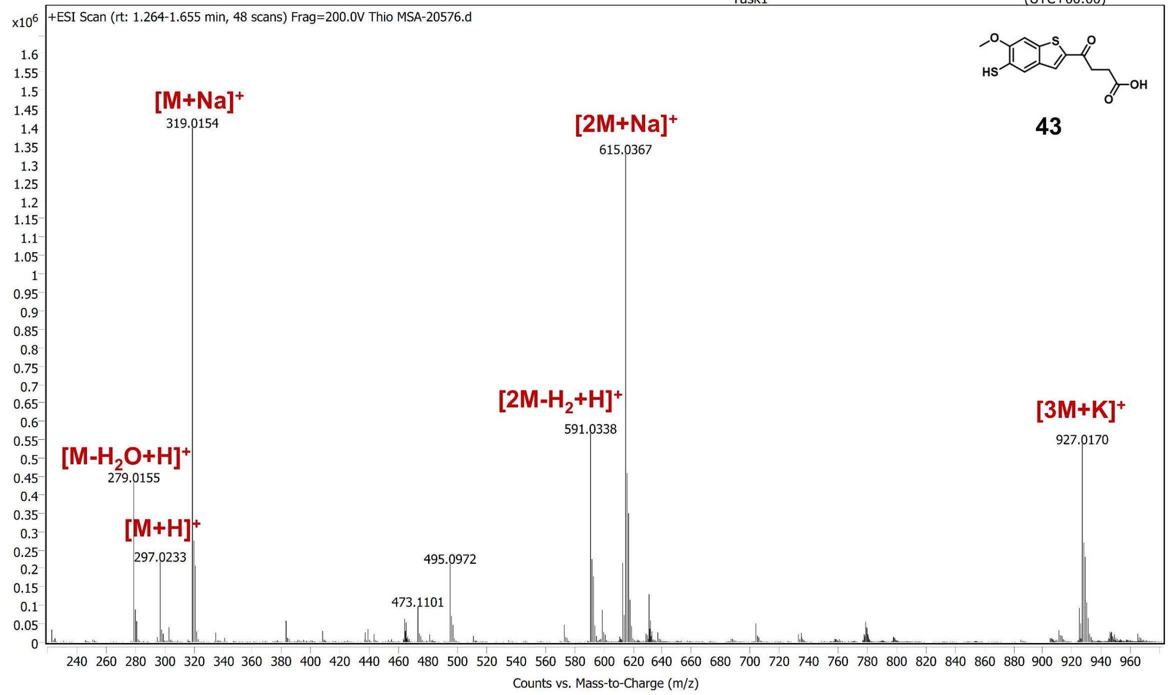

 Generated at 5:06 PM on 2/5/2022

Spectrum Plot Report

Agilent | Trusted Answers

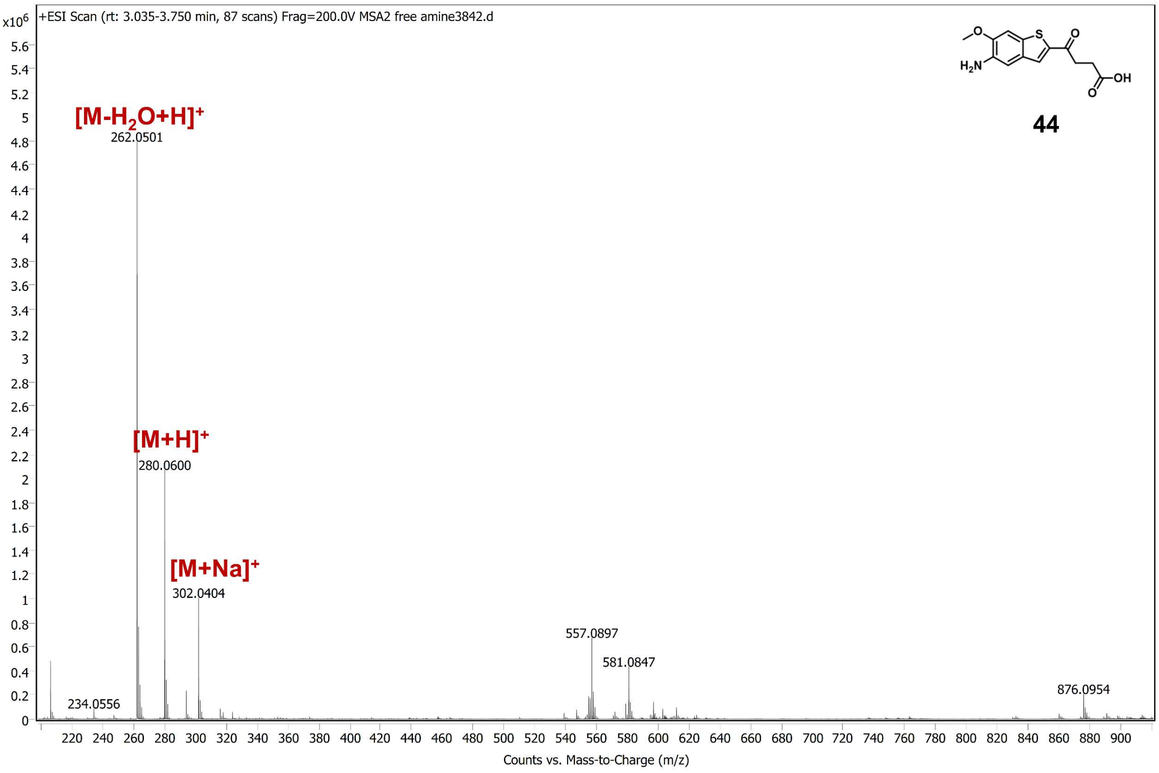

 Generated at 3:09 PM on 10/17/2023

Spectrum Plot Report

Agilent | Trusted Answers

Name	13 Compound	Rack Pos.		Instrument	Instrument 1	Operator	Nai-Shu Hsu
Inj. Vol. (ul)	10	Plate Pos.		IRM Status	All ions missed		
Data File	13 Compound3795.d	Method (Acq)	Walkup Positive SM.m	Comment	MBAG/546-G100088-Task2	Acq. Time (Local)	10/4/2023 2:46:51 PM (UTC+01:00)

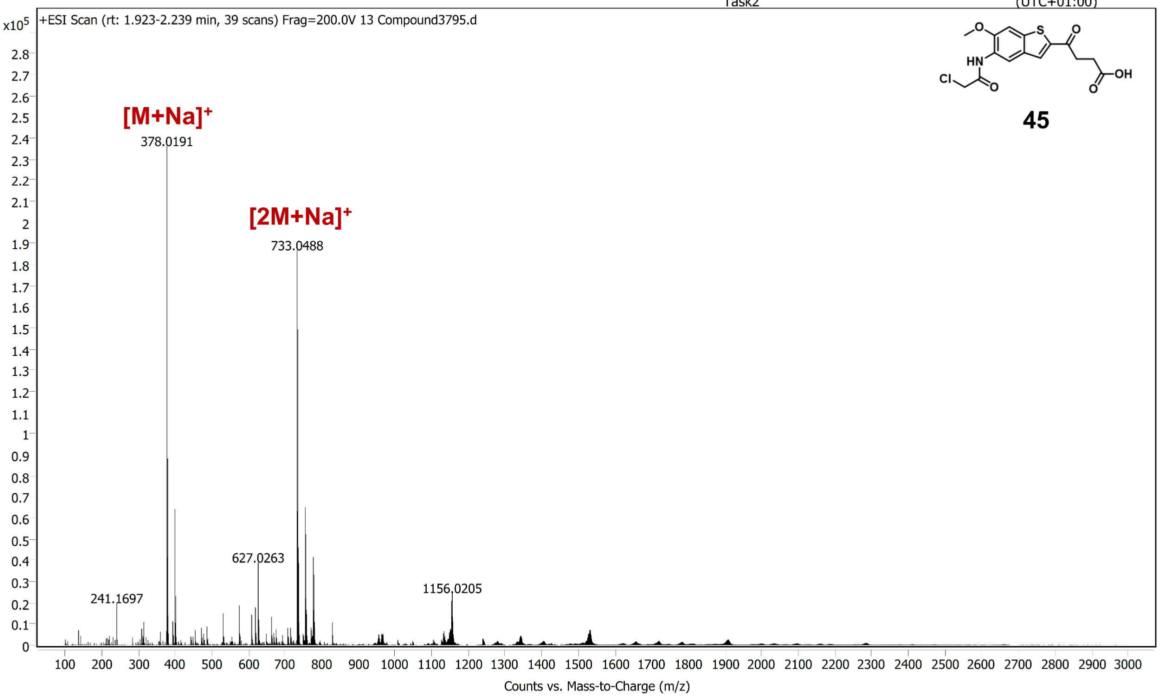

 Generated at 3:04 PM on 10/4/2023

Spectrum Plot Report

Name	Ac-beta-amidate	Rack Pos.		Instrument	Instrument 1	Operator	Nai-Shu Hsu
Inj. Vol. (ul)	1	Plate Pos.		IRM Status	Success		
Data File	Ac-beta-amidate2144.d	Method (Acq)	Walkup Positive SM.m	Comment	MBAG/546-G100088-Task2	Acq. Time (Local)	12/27/2022 5:49:20 PM (UTC+00:00)

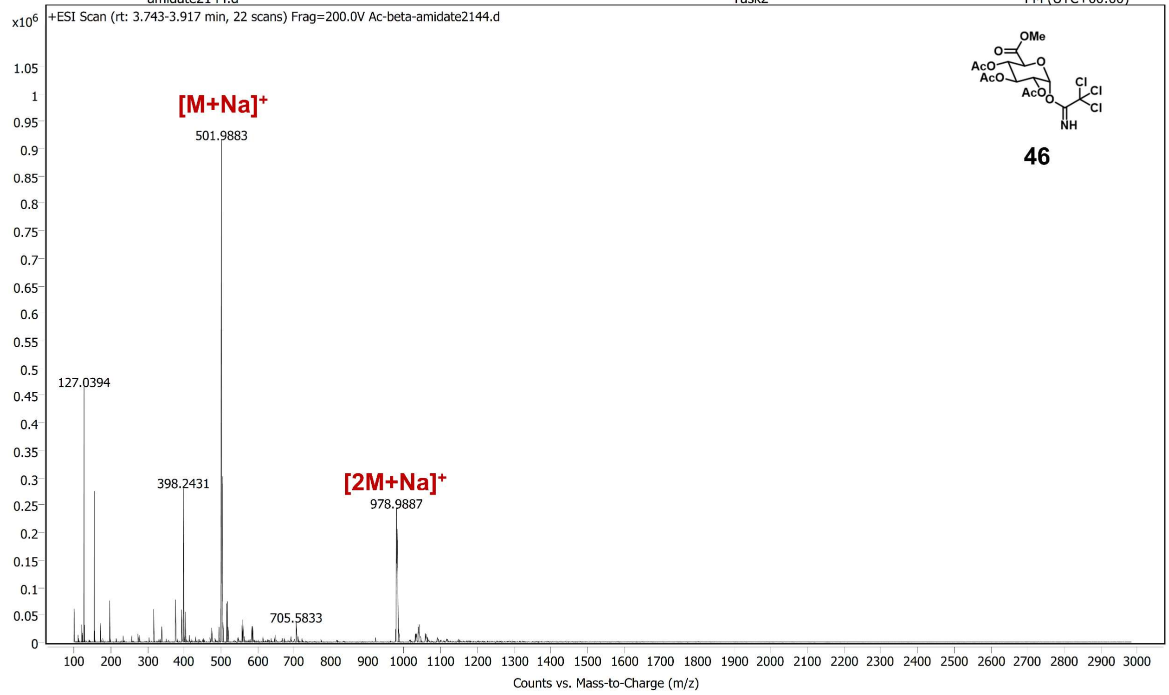

 Generated at 7:08 PM on 12/27/2022

Spectrum Plot Report

Name	Ac-Beta-O-Me-Sph mix	Rack Pos.		Instrument	Instrument 1	Operator	Nai-Shu Hsu
Inj. Vol. (ul)	1	Plate Pos.		IRM Status	Success		
Data File	Ac-Beta-O-Me-Sph mix2086.d	Method (Acq)	Walkup Positive SM.m	Comment	MBAG/546-G100088-task2	Acq. Time (Local)	12/17/2022 8:10:54 PM (UTC+00:00)

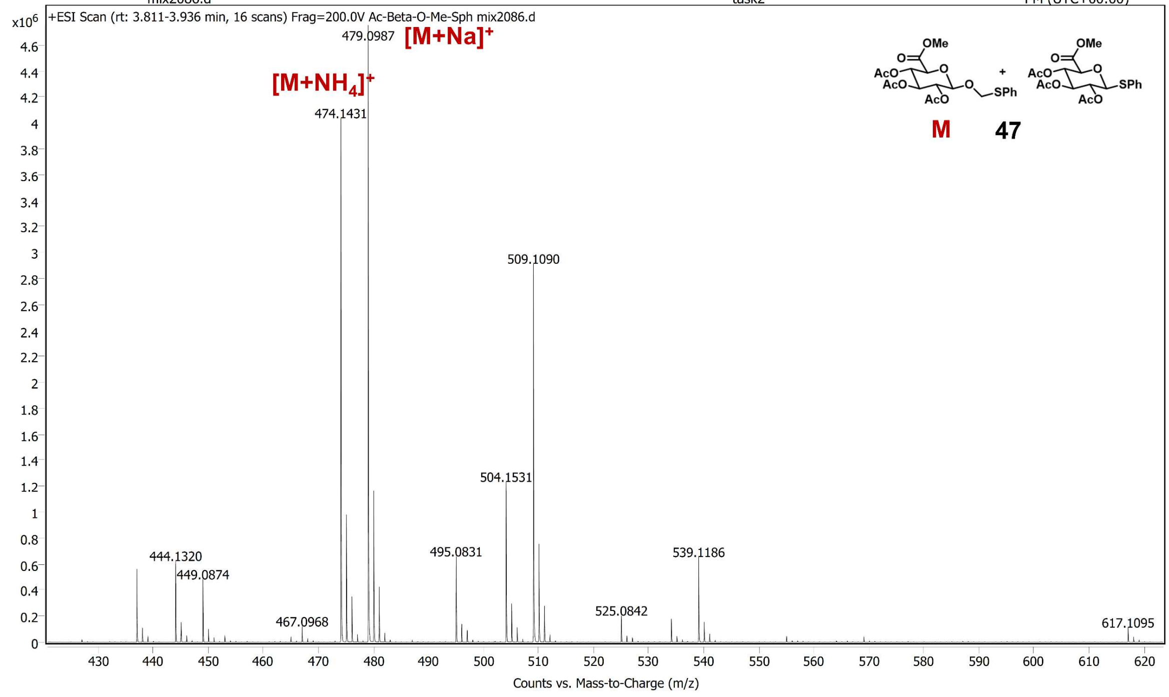

 Generated at 3:33 PM on 12/23/2022

Spectrum Plot Report

Name	beta-O-me-Cl direct	Rack Pos.		Instrument	Instrument 1	Operator	Nai-Shu Hsu
Inj. Vol. (ul)	10	Plate Pos.		IRM Status	Success		
Data File	beta-O-me-Cl direct1856.d	Method (Acq)	Walkup POSITIVE BYPASS.m	Comment	MBAG/461-RG97836-Task1	Acq. Time (Local)	10/27/2022 9:06:48 PM (UTC+01:00)

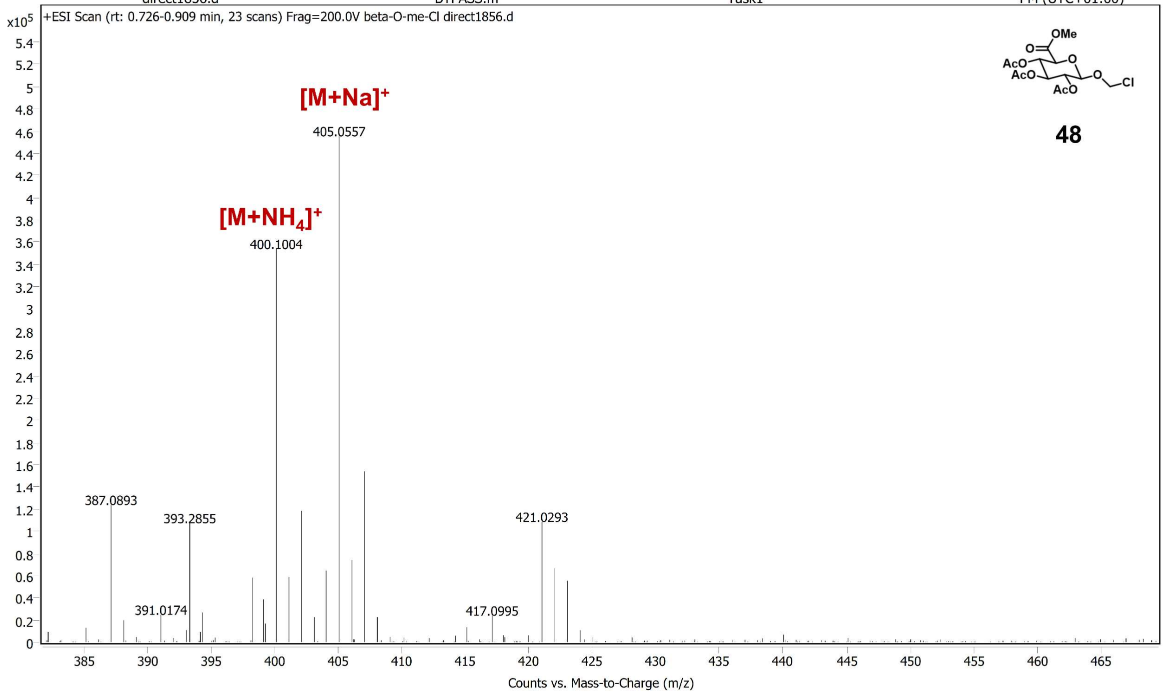

Spectrum Plot Report

Name	Ac-beta-glu-MSA2Et	Rack Pos.		Instrument	Instrument 1	Operator	Nai-Shu Hsu
Inj. Vol. (ul)	5	Plate Pos.		IRM Status	Some ions missed		
Data File	Ac-beta-glu-MSA2Et2084.d	Method (Acq)	Walkup Positive SM.m	Comment	MBAG/546-G100088-task2	Acq. Time (Local)	12/17/2022 3:41:41 PM (UTC+00:00)

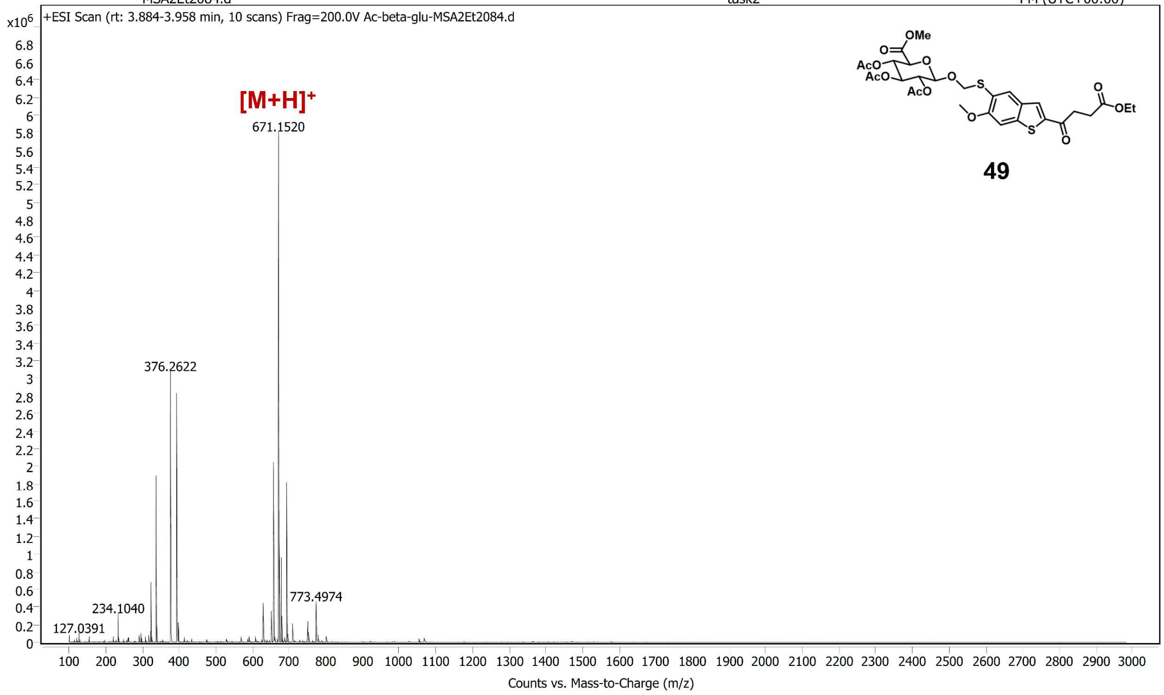

Spectrum Plot Report

Name	beta-glu-MSA2	Rack Pos.		Instrument	Instrument 1	Operator	Nai-Shu Hsu
Inj. Vol. (ul)	2	Plate Pos.		IRM Status	Some ions missed		
Data File	beta-glu-MSA22163.d	Method (Acq)	Walkup Positive SM.m	Comment	MBAG/546-G100088-Task2	Acq. Time (Local)	1/6/2023 7:59:55 PM (UTC+00:00)

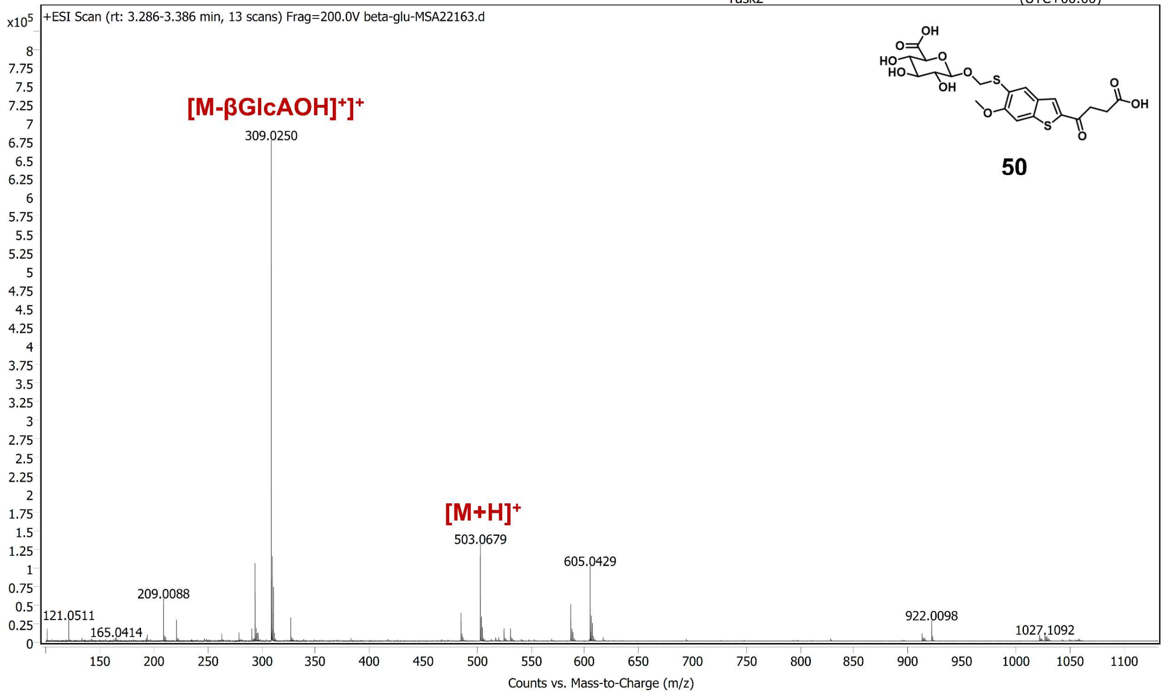

Page 1 of 1 Generated at 8:32 PM on 1/6/2023

References

1. Pan B-S, Perera SA, Piesvaux JA, Presland JP, Schroeder GK, Cumming JN*, et al.* An orally available non-nucleotide STING agonist with antitumor activity. *Science* 2020, **369**(6506)**:** eaba6098.

2. Itoh T, Mase T. A general palladium-catalyzed coupling of aryl bromides/triflates and thiols. *Org. Lett.* 2004, **6**(24)**:** 4587-4590.

3. Cooper P, Crisenza GEM, Feron LJ, Bower JF. Iridium-catalyzed α-selective arylation of styrenes by dual C−H functionalization. *Angew. Chem., Int. Ed.* 2018, **57**(43)**:** 14198-14202.

4. Meguro T, Yoshida S, Hosoya T. Aromatic azido-selective reduction via the Staudinger reaction using tri-n-butylphosphonium tetrafluoroborate with triethylamine. *Chem. Lett.* 2017, **46**(4)**:** 473-476.

5. Andersen J, Madsen U, Björkling F, Liang X. Rapid synthesis of aryl azides from aryl halides under mild conditions. *Synlett* 2005, **2005**(14)**:** 2209-2213.

6. Elferink H, Titulaer WHC, Derks MGN, Veeneman GH, Rutjes FPJT, Boltje TJ. Chloromethyl glycosides as versatile synthons to prepare glycosyloxymethyl-prodrugs. *Chem. - Eur. J.* 2022, **28**(9)**:** e202103910.

Supplementary Figure Source Data

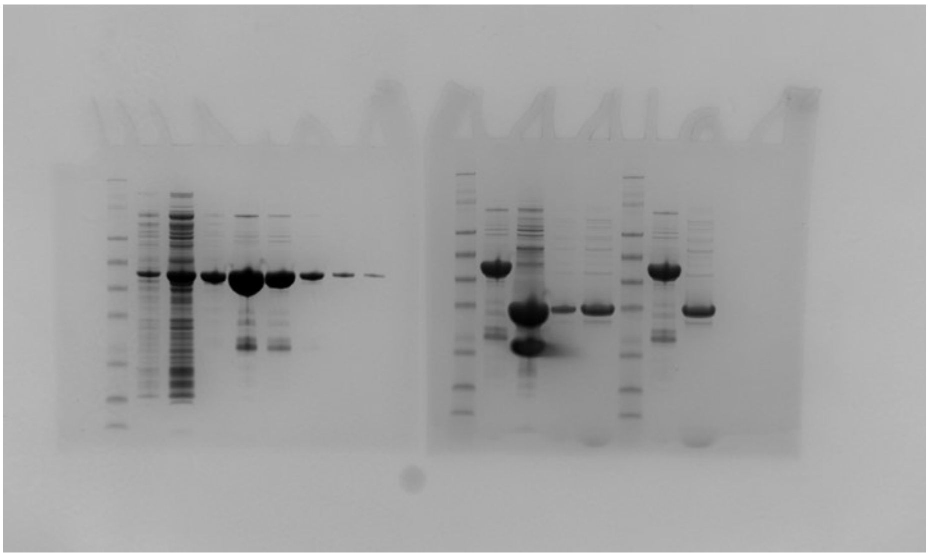

Source data for Supplementary Figure 5.
