# Supplementary material for: Tumour-specific STING agonist synthesis via a two-component prodrug system
Source: Nat Chem. 2025 Sep 16;17(12):1941–51. doi: 10.1038/s41557-025-01930-9 (PMC12669040; doi:10.1038/s41557-025-01930-9)
Supplement: Supplementary file 2 — Reporting Summary [file 41557_2025_1930_MOESM2_ESM.pdf]

nature portfolio

Corresponding author(s): Gonçalo J. L. Bernardes

Last updated by author(s): Jul 13, 2025

Reporting Summary

Nature Portfolio wishes to improve the reproducibility of the work that we publish. This form provides structure for consistency and transparency in reporting. For further information on Nature Portfolio policies, see our Editorial Policies and the Editorial Policy Checklist.

Statistics

For all statistical analyses, confirm that the following items are present in the figure legend, table legend, main text, or Methods section.

n/a	Confirmed	
☐	☒	The exact sample size (*n*) for each experimental group/condition, given as a discrete number and unit of measurement
☐	☒	A statement on whether measurements were taken from distinct samples or whether the same sample was measured repeatedly
☐	☒	The statistical test(s) used AND whether they are one- or two-sided<br>*Only common tests should be described solely by name; describe more complex techniques in the Methods section.*
☒	☐	A description of all covariates tested
☐	☒	A description of any assumptions or corrections, such as tests of normality and adjustment for multiple comparisons
☐	☒	A full description of the statistical parameters including central tendency (e.g. means) or other basic estimates (e.g. regression coefficient) AND variation (e.g. standard deviation) or associated estimates of uncertainty (e.g. confidence intervals)
☐	☒	For null hypothesis testing, the test statistic (e.g. *F*, *t*, *r*) with confidence intervals, effect sizes, degrees of freedom and *P* value noted<br>*Give P values as exact values whenever suitable.*
☒	☐	For Bayesian analysis, information on the choice of priors and Markov chain Monte Carlo settings
☒	☐	For hierarchical and complex designs, identification of the appropriate level for tests and full reporting of outcomes
☐	☒	Estimates of effect sizes (e.g. Cohen's *d*, Pearson's *r*), indicating how they were calculated

Our web collection on statistics for biologists contains articles on many of the points above.

Software and code

Policy information about availability of computer code

Data collection	MassLynx V4.1 for acquisition of LC-MS traces; Agilent Mass Hunter V4.1 for high-resolution mass-spectroscopy characterization of compounds; SoftMax Pro 7.0 for data acquisition of plate assays; MicroCal Analysis for acquisition of ITC data; TopSpin 3.6.5 and 3.6.4 on 700 Mhz and 500 Mhz spectometer, respectively, for NMR spectra acquisition; Molecular dynamics (MD) simulations were conducted using the AMBER 22 software suite (Antechamber and Xleap modules for input files; PMEMD.CUDA module for trajectory calculations; CPPTRAJ module for subsequent analysis of the trajectories) for Moleculr Dynamics simulations. Zeiss Zen (Zen blue edition) on Zeiss LSM 980 and Fusion software 1.1.1.17 on BC43 Andor confocal microscopes for confocal microscopy. X-ray crystals were obtained at DIAMOND LIGHT SOURCE using beamline i04.
Data analysis	GraphPad Prism 10 for bar diagrams/heatmap plotting and statistical analysis; Origin 2025 for plotting mass spectra; Agilent Qualitative Analysis 10.0; MicroCal Analysis for fitting the ITC binding curve; MestReNova V14.2.1 for NMR spectra analysis; FIJI ImageJ 2.14 for analysis of confocal fluorescence microscopy images; PyMOL 3.0 for creating the molecular movies; Biorender, Adobe Illustrator, and Microsoft Powerpoint for making figures; X-ray crystallography-related: BUSTER 2.10.4 for phasing and refinement; Aimless 0.8.2 for data scaling; autoPROC 1.0.5 for data reduction.

For manuscripts utilizing custom algorithms or software that are central to the research but not yet described in published literature, software must be made available to editors and reviewers. We strongly encourage code deposition in a community repository (e.g. GitHub). See the Nature Portfolio guidelines for submitting code & software for further information.

Data

Policy information about availability of data

All manuscripts must include a data availability statement. This statement should provide the following information, where applicable:

- Accession codes, unique identifiers, or web links for publicly available datasets
- A description of any restrictions on data availability
- For clinical datasets or third party data, please ensure that the statement adheres to our policy

The co-crystal structure of Compound D5 and STING LBD has been deposited in the RCSB Protein Data Bank with the access code 9QVT. Detailed synthetic procedures and compound characterization data such as NMR and HRMS spectra are available in Supplementary Information. Numerical source data of the figures are provided with the paper.

Human research participants

Policy information about studies involving human research participants and Sex and Gender in Research.

Reporting on sex and gender	N/A
Population characteristics	N/A
Recruitment	N/A
Ethics oversight	N/A

Note that full information on the approval of the study protocol must also be provided in the manuscript.

Field-specific reporting

Please select the one below that is the best fit for your research. If you are not sure, read the appropriate sections before making your selection.

☒ Life sciences ☐ Behavioural & social sciences ☐ Ecological, evolutionary & environmental sciences

For a reference copy of the document with all sections, see nature.com/documents/nr-reporting-summary-flat.pdf

Life sciences study design

All studies must disclose on these points even when the disclosure is negative.

Sample size	In all other zebrafish xenografts experiments using cell lines we prepared always tumor cells in excess and tried to inject as much as possible zf embryos, limiting factor is the no of the embryos that were layed and then the xenografts that died or acquired cardiac edema. Also sometimes the limiting is the penetrance of the transgenes (mpeg/tnf)– that in some cases gives low positive transgenics. We found empirically that with a minimal of 6-7 xenografts we can do the stats, but always aimed at having ~or more 10 xenografts analyzed per condition.For the quantification of implantation rates, tumor size, and % of apoptosis, confocal images acquired with the Zeiss LSM 980 were individually and manually quantified. The quantity of cells obtained for each experiment and the number of zebrafish embryos analyzed in the end are variable. This variability depends not only on the initial sample size but also on intrinsic characteristics of the tumor, such as cellularity. A sample size of 6 mice is common practice for preclinical efficacy evaluation in mouse study.
Data exclusions	To enable statistical analysis, we established a minimum requirement of 10 zebrafish embryos analyzed for each experimental condition. We excluded 2 values - statistical outliers, determined by GraphPad QuickCalcs: outlier calculator, 2 in Fig. 5h-i - M1/M2 macrophages (1 at 2dpi and another at 4dpi), which would otherwise complicate the statistical analysis performed. Outlier data has been included in the source data file.
Replication	To verify the reproducibility of the experimental findings most experiments were repeated at least two times. The number of independent experiments realized for each cell line is indicated in the corresponding image/subtitle. For mouse tumor models, the experiments have been repeated once (unpublished data during the first revision of the manuscript) with similar results.
Randomization	Before injection, we randomly mix a pool of zebrafish larvae and anesthetize them, in the moment of injection we randomly take some larvae for injection. 1day post injection we sort xenografts according to their tumor size and then randomly distributed in each experimental group.
Blinding	The zebrafish experimental work, quantifications and analysis were performed by Raquel Mendes without blinding.

Reporting for specific materials, systems and methods

We require information from authors about some types of materials, experimental systems and methods used in many studies. Here, indicate whether each material, system or method listed is relevant to your study. If you are not sure if a list item applies to your research, read the appropriate section before selecting a response.

Materials & experimental systems

n/a	Involved in the study
☐	☒ Antibodies
☐	☒ Eukaryotic cell lines
☒	☐ Palaeontology and archaeology
☐	☒ Animals and other organisms
☒	☐ Clinical data
☒	☐ Dual use research of concern

Methods

n/a	Involved in the study
☒	☐ ChIP-seq
☒	☐ Flow cytometry
☒	☐ MRI-based neuroimaging

Antibodies

Antibodies used

Cell Signaling Technologies 50907 (anti-phospho STING IgG, 1:1000), 37829 (rabbit anti-IRF3 IgG, 1:1000), 4970 (rabbit anti-beta actin IgG, 1:1000), 7074 (Ggoat anti-rabbit IgG, HRP-linked, 1:1000), 5483 (rabbit anti-phosphoTBK1 IgG, 1:1000), cat#9661 (rabbit anti-Cleaved Caspase3, 1:100), #18799 (rabit anti-Vinculin,1:1000). Roche cat#11814460001 (mouse anti-GFP, 1:100). Abcam cat#ab167453 (rabbit anti-mcherry, 1:100), ab205719 (goat anti-mouse, 1:10000). Thermo Fisher Scientific cat#10688674 (goat anti-mouse DyLight 488), cat#10108403 (goat anti-rabbit DyLight 594, 1:400). Proteintech cat#16332-1-AP (rabbit anti-GUSB, 1:1000).

Validation

Validations of antibodies are provided on the respective manufacturer's website.

Eukaryotic cell lines

Policy information about cell lines and Sex and Gender in Research

Cell line source(s)

THP-1 cells were from Cytion (then Cell Line Service, Cat. 300356). Engineered THP-1 Lucia ISG cells were from InvivoGen (cat. thpl-isg). Human breast cancer cell lines Hs578T, were derived from American Type Culture Collection and kindly provided by Monica Bettencourt Dias'Lab (Instituto Gulbenkian de Ciência). MC38 was purchased from Kerafast (cat. ENH204-FP). CT26mbGUS was received as a gift from Dr. Steve R. Roffler's laboratory (Institute of Biomedical Sciences, Academia Sinica, Taiwan).

Authentication

THP-1, THP-1 Lucia ISG, MC38, and CT26mbGUS were not further authenticated. Hs578T were authenthicated through short tandem repeat (STR) profile analysis.

Mycoplasma contamination

THP-1 and THP-1 Lucia ISG cells were not further tested for mycoplasma; their growth media contained the Normocin (InVivoGen cat. ant-nr-05) antibiotic formulation for preventing mycoplasma contamination. Hs578T were tested and were negative for mycoplasma.

Commonly misidentified lines
(See ICLAC register)

No commonly misidentified lines were used.

Animals and other research organisms

Policy information about studies involving animals; ARRIVE guidelines recommended for reporting animal research, and Sex and Gender in Research

Laboratory animals

8-week-old male and female C57BL/6J or BALB/c mice (purchased from Charles River) were used in the study. The light/dark cycle was 14h light/10h dark (lights on at 07:00; lights off at 21:00). The temperature was 20–24°C and the relative humidity was 55±10%, with controlled supply of HEPA-filtered air provided to individually ventilated cages. Maximum number of animals per cage was five. Social isolation was avoided whenever possible. The type of food was autoclaved diet pellets RM3A (P), from SDS Special Diets Services (801030). Food was placed in a grid inside the cage and provided ad libitum to animals. The type of water was sterile water treated by reverse osmosis. Water was provided ad libitum to animals through bottles with a capillary hole.

Wild animals

Study did not involve wild animals.

Reporting on sex

Sex is not relevant in this study, and most animals used were female mice.

Field-collected samples

Study did not involve field-collected samples.

Ethics oversight

All mice experiments were conducted at the Instituto de Medicina Molecular João Lobo Antunes (IMM, Lisbon). Animal work was performed with strict adherence to the Portuguese Law (Portaria 1005/92) and the European Guideline 86/609/EEC. The Federation of European Laboratory Animal Science Associations guidelines and recommendations concerning laboratory animal welfare were followed. All animal experiments were approved by the Portuguese official veterinary department for welfare licensing – Direção Geral de Alimentação e Veterinária (DGAV) and the IMM Animal Ethics Committee (authorization AWB_2021_03_GB_TargCancerDrugs). Mice were observed every 1-2 days and euthanized when either the tumor volume reached ~1000 mm3, the body weight loss exceeded 20%, or ulceration started to appear.
