# Supplementary material for: Tumour-specific STING agonist synthesis via a two-component prodrug system
Source: Nat Chem. 2025 Sep 16;17(12):1941–51. doi: 10.1038/s41557-025-01930-9 (PMC12669040; doi:10.1038/s41557-025-01930-9)
Supplement: Supplementary file 4 — Statistical source data. [file 41557_2025_1930_MOESM4_ESM.pdf]

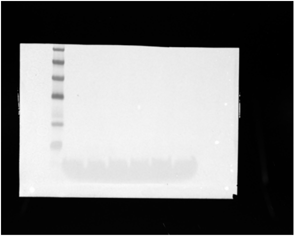

β-actin ladder

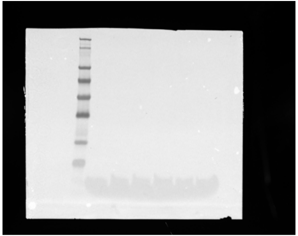

pSTING ladder

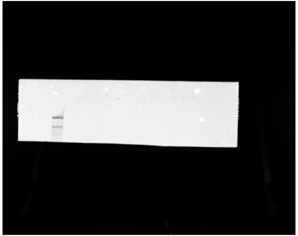

pTBK-1 ladder

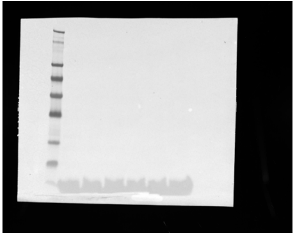

pIRF3 ladder

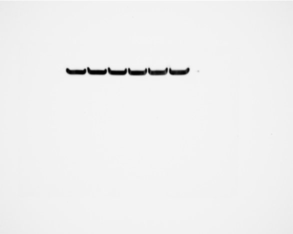

β-actin chemiluminescence

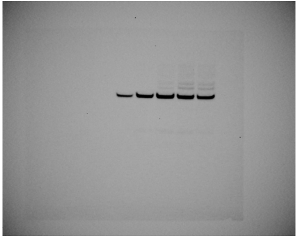

pSTING chemiluminescence

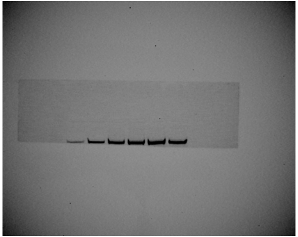

pTBK-1 chemiluminescence

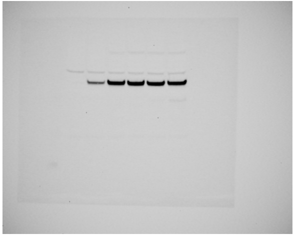

pIRF3 chemiluminescence

Blots for β-actin and TBK-1 were from the same gel. The membrane was cut after transferring the bands. The rest are loaded with the same amounts of samples in each well.
