# Supplementary figures and images for: Tumour-specific STING agonist synthesis via a two-component prodrug system
Source: Nat Chem. 2025 Sep 16;17(12):1941–51. doi: 10.1038/s41557-025-01930-9 (PMC12669040; doi:10.1038/s41557-025-01930-9)

Vinculin

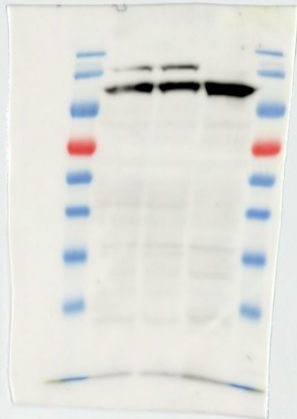

$\beta$ GUS

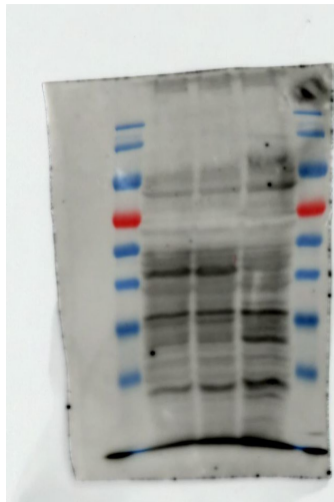

Supplement: Supplementary file 12 — Original blot. [file 41557_2025_1930_MOESM12_ESM.pdf]
